# A contorted nanographene shelter

**DOI:** 10.1038/s41467-021-25255-6

**Published:** 2021-08-31

**Authors:** Huang Wu, Yu Wang, Bo Song, Hui-Juan Wang, Jiawang Zhou, Yixun Sun, Leighton O. Jones, Wenqi Liu, Long Zhang, Xuan Zhang, Kang Cai, Xiao-Yang Chen, Charlotte L. Stern, Junfa Wei, Omar K. Farha, Jessica M. Anna, George C. Schatz, Yu Liu, J. Fraser Stoddart

**Affiliations:** 1 Department of Chemistry, Northwestern University, Evanston, IL USA; 2 College of Chemistry, State Key Laboratory of Elemento-Organic Chemistry, Nankai University, Nankai District, Tianjin China; 3 Department of Chemistry, University of Pennsylvania, Philadelphia, PA USA; 4 Key Laboratory of Applied Surface and Colloid Chemistry (Ministry of Education), Key Laboratory for Macromolecular Science of Shaanxi Province, School of Chemistry and Chemical Engineering, Shaanxi Normal University, Xi’an, China; 5 Collaborative Innovation Center of Chemical Science and Engineering (Tianjin), Nankai District, Tianjin China; 6 School of Chemistry, University of New South Wales, Sydney, NSW Australia; 7 Stoddart Institute of Molecular Science, Department of Chemistry, Zhejiang University, Hangzhou, China; 8 ZJU-Hangzhou Global Scientific and Technological Innovation Center, Hangzhou, China

**Keywords:** Supramolecular chemistry, Interlocked molecules, Self-assembly

## Abstract

Nanographenes have kindled considerable interest in the fields of materials science and supramolecular chemistry as a result of their unique self-assembling and optoelectronic properties. Encapsulating the contorted nanographenes inside artificial receptors, however, remains challenging. Herein, we report the design and synthesis of a trigonal prismatic hexacationic cage, which has a large cavity and adopts a relatively flexible conformation. It serves as a receptor, not only for planar coronene, but also for contorted nanographene derivatives with diameters of approximately 15 Å and thicknesses of 7 Å. A comprehensive investigation of the host-guest interactions in the solid, solution and gaseous states by experimentation and theoretical calculations reveals collectively an induced-fit binding mechanism with high binding affinities between the cage and the nanographenes. Notably, the photostability of the nanographenes is improved significantly by the ultrafast deactivation of their excited states within the cage. Encapsulating the contorted nanographenes inside the cage provides a noncovalent strategy for regulating their photoreactivity.

## Abstract (web summary)

Encapsulating large and contorted nanographenes inside artificial receptors remain challenging. This work reports the synthesis, characterization and binding properties of a trigonal prismatic cage compound that can serve as a receptor for contorted nanographene derivatives.

## Introduction

**Fig. 1:**
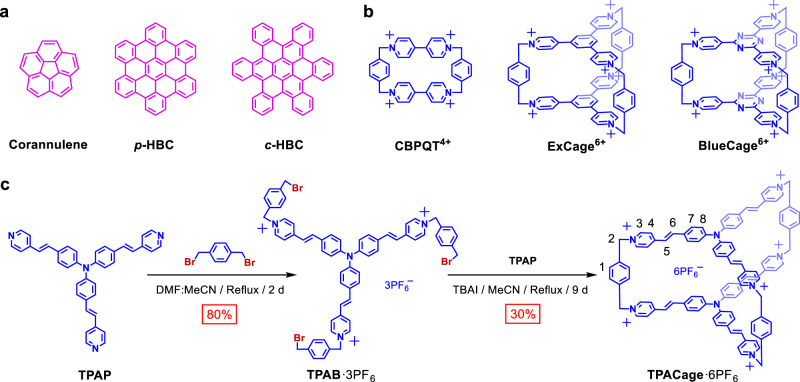
Structures and synthesis. **a** Structural formulas of corannulene, ***p*-HBC** and ***c*-HBC**. **b** Structural formulas of **CBPQT**^4+^, **ExCage**^6+^, and **BlueCage**^6+^. **c** Synthesis and labeling of protons on **TPACage**^6+^.

Nanographenes^1–4^ (**NGs**), a class of large polycyclic aromatic hydrocarbons (**PAHs**) that extend over 1.0 nm^5^, have attracted considerable attention both in the scientific community and in technological spheres on account of their unique self-assembling^6,7^, redox^8^, and optoelectronic properties^9–11^. Of particular interest are the coronene (**COR**) homologous molecules (Fig. 1a), such as the bowl-like corannulene^12^, planar hexa-*peri*-hexabenzocoronene^13–15^ (***p*-HBC**) and contorted hexa-*cata*-hexabenzocoronene^16,17^ (***c*-HBC**), which have been widely applied in field-effect transistors^18^, light-emitting diodes^19^, and nonlinear optical materials^20^. Along with the advent of supramolecular chemistry^21–26^, investigations^27–29^ on artificial receptors for **PAHs**, on the basis of noncovalent bonding interactions, have become an active research area in recent decades. To date, a series of receptors, including, macrocycles^30^, tweezers^31^, covalent^32^, and coordination^33,34^ cages, as well as carbon nanotubes^35^, have been synthesized as hosts for **COR**. Several receptors^36–40^ are even able to bind bowl-like corannulene, despite the fact that they show lower binding affinities compared to that for **COR**. Well-crafted receptors for the larger and contorted ***c*-HBC**, however, are still few and far between, for the simple reason that their contorted conformations lead to (i) weaker [π···π] interactions between the hosts and guests, and (ii) less shape complementarity in order to fit within the cavity of the host^36,40^. In addition, large nanographenes often suffer from instability upon exposure to light^41,42^, reducing considerably the stability and life of graphene-based materials. Typical examples are the acenes^43,44^ larger than pentacene, an important class of one-dimensional nanographenes, which have been found to be unstable to light. This deficiency limits their utility severely. Hence, designing an artificial receptor, with the property of high binding affinities toward nanographenes and protecting them from photo-degradation, is a challenging, yet worthwhile, objective in noncovalent synthesis.

Macrocyclic arenes, such as calix[n]arenes^45^, calixpyrroles^46,47^, cyanostars^48,49^, and pillar[n]arenes^50,51^, represent a rapidly growing family of molecular receptors that play a crucial role in supramolecular chemistry by virtue of their properties of molecular recognition towards various guests. As a representative cationic cyclophane, cyclobis(paraquat-*p*-phenylene)^52,53^ (**CBPQT**^4+^, Fig. 1b), not only shows excellent molecular recognition properties^54^ but also serves as a vital building block for constructing mechanically interlocked molecules^55^ (MIMs). In attempts to modulate the dimensions of **CBPQT**^4+^, the 1,4-disubstituted phenylene rings have been replaced by 1,3,5-trisubstituted benzenoid ones in order to obtain cage-like cyclophanes^56^, such as, **ExCage**^6+^ (Fig. 1b), which exhibits higher complexation strengths towards a series of neutral **PAHs**. Upon changing the central benzenoid rings within **ExCage**^6+^ into π-electron-deficient triazine rings, a more electron-poor cyclophane^57^, so-called **BlueCage**^6+^ (Fig. 1b), was obtained. Since it acts as a receptor for both planar aromatic guests and [PF_6_]^−^ anions, binding affinities for the aromatic guests can be controlled by anion–π interactions. These two cage-like receptors, however, can only bind planar and relatively small aromatic guests because of their rigid conformations and the limited space within their cavities.

In this investigation, we introduced two triphenylamine units into a hexacationic cage, **TPACage**^6+^ (Fig. 1c). Its solid-state structure reveals that the cage adopts a relatively flexible conformation and has a large cavity with a diameter of 20.8 Å and a volume of 368 Å^3^. As a result, **TPACage**^6+^ can form 1:1 complexes, not only with planar **COR** but also with large contorted ***c*-HBC** derivatives with diameters of 15.3 Å, as evidenced by single-crystal X-ray diffraction, NMR spectroscopy, and high-resolution mass spectrometry (HRMS). Benefiting from ideal size-matching, **TPACage**^6+^ shows higher binding affinities towards ***c*-HBC** derivatives than that with **COR** in the solution state. Gradient tandem mass spectrometry revealed that as the electron density of guests increases, the stability of the host-guest complexes are enhanced in the gas phase. As a consequence of the ultrafast energy transfer between the host and guests, the photostability of ***c*-HBC** guests is significantly improved within the cavity of **TPACage**^6+^, as confirmed by femtosecond transient absorption spectroscopy.

## Results

### Synthesis and characterization of TPACage^**6**+^

The **TPACage**^6+^ was synthesized (Fig. 1c and Supplementary Figs. 1–3) in three steps from commercially available starting materials. A coupling reaction between tris(4-bromophenyl)amine and 4-vinylpyridine in triethylamine was carried out under reflux for 6 h to obtain **TPAP** in a 58% yield. Thereafter, an S_N_2 reaction between an excess of *p*-xylylene dibromide and **TPAP** in a MeCN/DMF mixture under reflux for 2 days led to the formation of **TPAB**•3PF_6_ after counterion exchange in a yield of 80%. Finally, equimolar amounts of **TPAB**•3PF_6_ and **TPAP** in the presence of 0.2 equiv. of tetrabutylammonium iodide (TBAI) as a catalyst were heated under reflux in MeCN for 9 days, resulting in the isolation of the crude chloride salt as a red solid after precipitating with tetrabutylammonium chloride (TBACl). The desired product **TPACage**•6PF_6_ was obtained in 30% yield after purification by reverse-phase column chromatography, followed by counterion exchange (NH_4_PF_6_/H_2_O). Furthermore, another two salts—namely **TPACage**•6Cl and **TPACage**•6AsF_6_—were obtained from **TPACage**•6PF_6_ by counterion exchange in yields of 95% and 96%, respectively.

**TPACage**^6+^ was fully characterized by one-dimensional (1D, Supplementary Figs. 9–15) and two-dimensional (2D, Supplementary Figs. 22–26) ^1^H/^13^C NMR spectroscopies, as well as by high-resolution mass spectrometry (HRMS). In the HRMS of **TPACage**•6PF_6_, peaks with *m/z* values of 313.1297 ([**TPACage**•PF_6_]^5+^), 427.6539 ([**TPACage**•2PF_6_]^4+^), and 618.5258 ([**TPACage**•3PF_6_]^3+^) were observed (Supplementary Fig. 36), and were shown to be consistent with the calculated values. The UV-Vis absorption spectrum of **TPACage**•6PF_6_ shows (Supplementary Fig. 46) a strong absorption at 487 nm, which originates from the 4-vinylpyridine-modified triphenylamine units in **TPACage**^6+^.

**Fig. 2:**
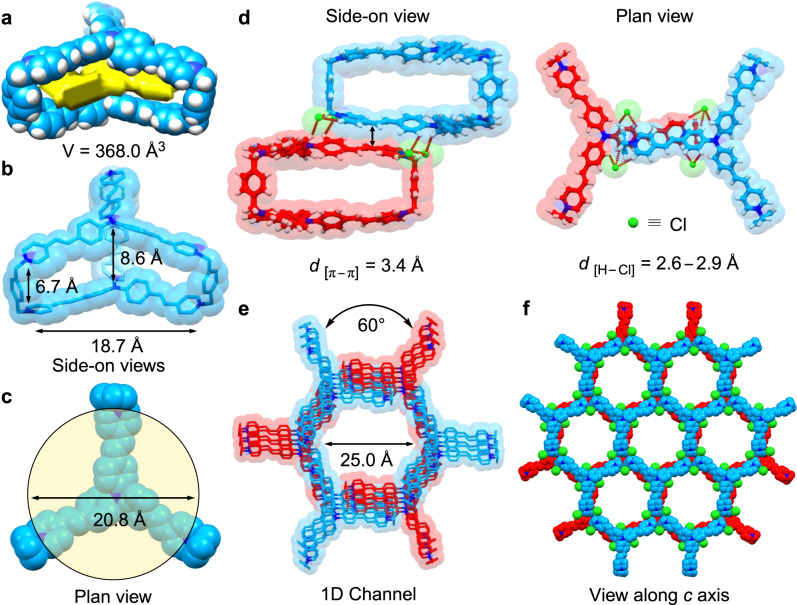
Solid-state (super)structure of **TPACage•6Cl**. **a**–**b** Capped-stick and space-filling representations of side-on views, showing the volume and dimensions of the cavity inside **TPACage**^6+^. **c** A cut-away space-filling plan view of the cavity inside **TPACage**^6+^, showing that its diameter is 20.8 Å. **d** Capped-stick and space-filling representations of different views, showing the [π···π] stacking interaction between two adjacent cages and the [C−H···Cl^−^] hydrogen bonding between **TPACage**^6+^ and Cl^−^ anions. **e–f** The solid-state superstructure of **TPACage**•6Cl, revealing how **TPACage**^6+^ assembles into a 3D framework with relatively large 1D channels with diameter of 25.0 Å. Solvent molecules have been omitted for the sake of clarity. C skyblue and red, H white, N blue, Cl green.

The solid-state (super)structure of **TPACage**^6+^ was determined unambiguously by single-crystal X-ray diffraction analysis of a dark red crystal, which was obtained by slow vapor diffusion of *i*Pr_2_O into a MeOH solution of **TPACage**•6Cl after four days. The cage displays (Fig. 2a) *D*_3h_ symmetry, and possesses (Fig. 2b) three identical large rectangular windows with average dimensions of 18.7 × 6.7 Å^2^. These rectangular windows allow potential guests to undergo association/dissociation with the internal cavity of the cage. The distance between the two central nitrogen atoms in the two TPAP propellers is (Fig. 2b) 8.6 Å, which is larger than that (6.7 Å) between the two nitrogen atoms bridged by the *p*-xylylene linkers. The volume of the resulting slot-like cavity is estimated (Fig. 2a) to be 368 Å^3^, indicating that **TPACage**^6+^ may serve as a receptor for some large **PAHs**. The solid-state superstructure of **TPACage**^6+^ reveals that it crystallizes in a hexagonal space group *P*6_3_/m and forms (Fig. 2f) a porous network with interconnected 1D channels. The diameters (Fig. 2e) of these hexagonal channels are 25 Å. Each channel is comprised of two styrene-pyridinium arms from three **TPACage**^6+^ molecules that are positioned in a *C*_3_-symmetric manner. Every styrene-pyridinium arm within the cage establishes (Fig. 2d) four sets of short contacts with its neighbors through [C−H···Cl] hydrogen bonding interactions with distances in the range of 2.6−2.9 Å. Combined with the intermolecular [π···π] stacking (Fig. 2d) between adjacent styrene-pyridinium arms with a plane-to-plane distance of 3.4 Å, two sets of three **TPACage**^6+^ are stacked with each other in a coaxial manner with a perpendicular rotation angle of 60°, forming (Fig. 2e) an infinite hexagonal channel. The third styrene-pyridinium arm within each cage is involved directly in the formation of adjacent channels. Consequently, a three-dimensional (3D) supramolecular organic framework with interconnected 1D channels along the *c*-axis is formed. This observation indicates that the **TPACage**^6+^ has the potential for constructing organic porous materials^58–60^.

### Solution-phase characterizations of the host-guest complexes

**Fig. 3:**
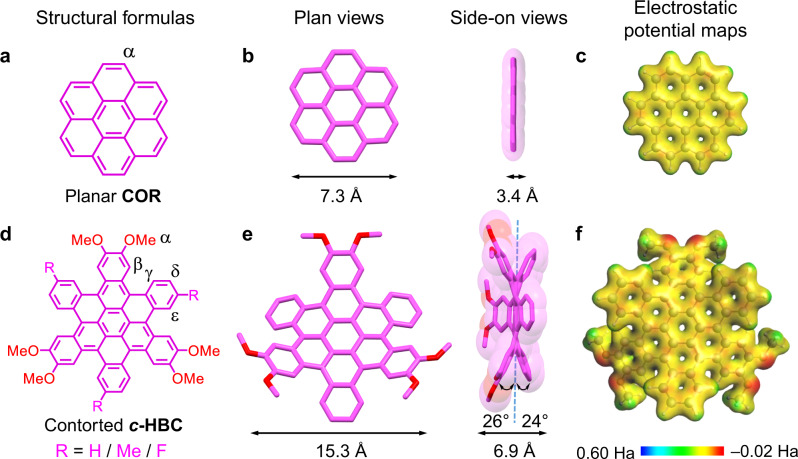
Structure formulas and solid-state structures of guest molecules. **a** Structural formula and labeling of the proton on the planar **COR**. **b**, **c** Capped-stick and space-filling representations of the solid-state structure (obtained from CCDC:1129883) and the electrostatic potential map of **COR**. **d** Structural formulas and labeling of protons on the contorted ***c*-HBC** guest molecules, namely, **3H-HBC**, **3Me-HBC**, and **3F-HBC**. **e**, **f** Capped-stick and space-filling representations of the solid-state structure and electrostatic potential map of **3H-HBC**, showing its characteristic dimensions, tilt angles, and electron density distribution.

**Fig. 4:**
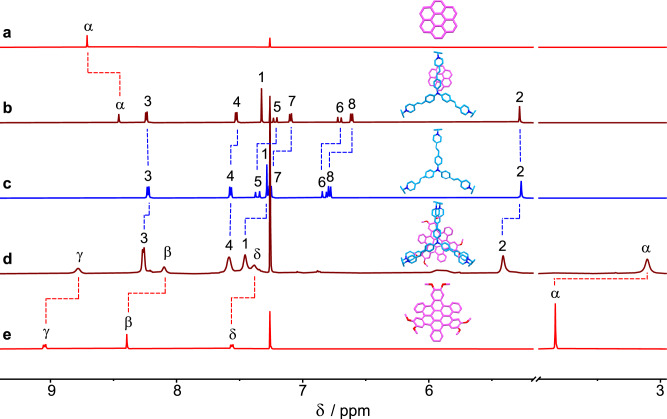
^1^H NMR Spectroscopic characterization. ^1^H NMR Spectra (500 MHz, CD_3_CN:CDCl_3_ = 4:1, [**TPACage**•6PF_6_] = [**COR**] = [**3H-HBC**] = o5.0 × 10^−4^ M, 298 K) of (**a**) **COR**, (**b**) **COR⊂TPACage**•6PF_6_, (**c**) **TPACage**•6PF_6_, (**d**) **3H-HBC⊂TPACage**•6PF_6_, (**e**) **3H-HBC**. The structural formula and the labeling of protons on **TPACage**^6+^ are defined in Fig. 1, while those for **COR** and **3H-HBC** are defined in Fig. 3.

Since the constitution of **TPACage**^6+^ is based on the well-known family^61^ of “extended viologen cyclophanes”, we can anticipate that **TPACage**^6+^ will be able to encapsulate **PAHs** as a result of primarily intermolecular [π···π] interactions. The planar **COR** (Figs. 3a–c), with a diameter of 7.3 Å and a thickness of 3.4 Å, was selected as a representative guest. In the ^1^H NMR spectrum of an equimolar mixture of **TPACage**^6+^ and **COR**, the chemical shifts of both the protons on **COR** (Δδ = −0.25 ppm for H-α) and on **TPACage**^6+^ (Δδ = −0.05, −0.14, −0.12, −0.17, and −0.17 ppm for H-4, H-5, H-6, H-7, and H-8, respectively) show (Fig. 4b) significant upfield shifts compared with the chemical shifts of the free **COR** and the free **TPACage**^6+^, indicating the presence of aromatic [π···π] stacking interactions between **COR** and the styrene-pyridinium units. A 2D ^1^H−^1^H ROESY spectrum confirmed (Supplementary Fig. 30) the binding mode with through-space correlations between H-α on **COR** and H-7 and H-8 on **TPACage**^6+^. A Job plot showed (Supplementary Fig. 29) a maximum at a mole fraction of 0.5, confirming the existence of a 1:1 stoichiometry between **TPACage**^6+^ and **COR** in solution. Upon adding dropwise 8 equiv. of **TPACage**^6+^ to a CD_3_CN / CDCl_3_ (4:1) solution of **COR**, the resonance for H-α on **COR** moved gradually upfield in the ^1^H NMR spectra (Supplementary Fig. 27), indicating that the association and disassociation of **COR⊂TPACage**^6+^ are undergoing fast exchange on the ^1^H NMR timescale. The binding constant (*K*_a_) was determined (Supplementary Fig. 28) to be 1.3 × 10^3^ M^−1^, according to the chemical shift changes undergone by H-α.

In order to explore the binding ability of **TPACage**^6+^ towards some larger and thicker **PAHs**, three *C*_3_-symmetrical contorted hexa-*cata*-hexabenzocoronene derivatives (Fig. 3d)—namely, **3H-HBC**, **3Me-HBC**, and **3F-HBC**—were synthesized (Supplementary Fig. 4) according to previously reported^62^ protocols. The single-crystal structure of **3H-HBC** reveals (Fig. 3e) that its diameter reaches 15.3 Å, and that it adopts a contorted conformation with a thickness of 6.9 Å. Upon adding an equimolar amount of **TPACage**^6+^ into a CD_3_CN / CDCl_3_ (4:1) solution of **3H-HBC**, the chemical shifts for protons on **TPACage**^6+^ and **3H-HBC** all show (Fig. 4d) marked changes with some of the peaks undergoing severe broadening in the ^1^H NMR spectrum, suggesting the formation of a host-guest complex. The peaks in the spectrum were assigned in so far as possible on the basis of in-depth analyses of their 2D ^1^H−^1^H COSY (Supplementary Fig. 31) and ROESY (Supplementary Fig. 32) spectra. The chemical shifts of protons on the 1,2-dimethoxybenzene rings (Δδ = −0.72 and −0.29 ppm for H-α and H-β, respectively) in **3H-HBC** exhibited (Fig. 4d) larger upfield shifts than those of H-γ and H-δ (Δδ = −0.27 and −0.18 ppm for H-γ and H-δ, respectively). This observation may result from the fact that the 1,2-dimethoxybenzene rings are located in the cavity formed by pairs of styrene-pyridinium units. The resonances for protons (H-5, H-6, H-7, and H-8) on the styrene units in **TPACage**^6+^ display (Fig. 4d) severe broadening, possibly on account of the free pedaling motion about the C=C double bonds within the **3H-HBC⊂TPACage**^6+^ complex. Protons H-1 and H-2, associated with the *p*-xylylene linkers, exhibit (Fig. 4d) downfield shifts (Δδ = 0.18 and 0.14 ppm for H-1 and H-2, respectively). In the ^1^H NMR spectra (Supplementary Fig. 33) of **3Me-HBC⊂TPACage**^6+^ and **3F-HBC⊂TPACage**^6+^, a new set of resonances also appears, accompanied by severe broadening. The resonances for protons H-1 and H-2 attached to the *p*-xylylene linkers show (Supplementary Fig. 33) characteristic downfield shifts, confirming that **3Me-HBC** and **3F-HBC** are encapsulated by **TPACage**^6+^. Because of the contorted conformations of the ***c*-HBC** guests, the association and dissociation of their complexes with **TPACage**^6+^ undergo (Supplementary Fig. 33) slow exchange on the ^1^H NMR timescale, in contrast with the fast exchange process observed for **COR⊂TPACage**^6+^. The cage serves as a receptor for both planar **COR** and contorted ***c*-HBC** derivatives in the solution state, on account of the relatively flexible conformation and the extensive cavity present in **TPACage**^6+^ .

High-resolution electrospray ionization mass spectrometry (HR-ESI-MS) also provided strong evidence for the formation of these four host-guest complexes. The monoisotopic masses of the four complexes (Supplementary Figs. 42−45), after deconvolution, were calculated as 2590.56 Da (**COR⊂TPACage**•6PF_6_), 3070.71 Da (**3H-HBC⊂TPACage**•6PF_6_), 3112.77 Da (**3Me-HBC⊂TPACage**•6PF_6_), and 3124.70 Da (**3F-HBC⊂TPACage**•6PF_6_), respectively. These values match well with the calculated ones.

**Fig. 5:**
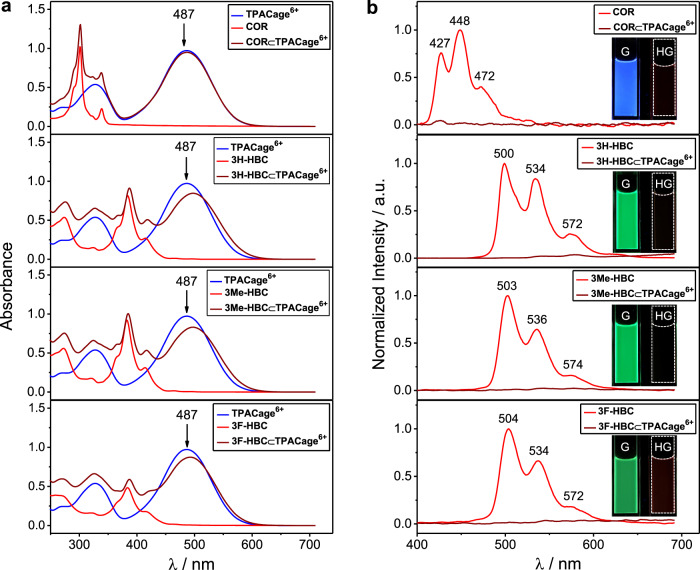
Photophysical characterization. **a** UV-Vis Absorption spectra (3 × 10^−5^ M, MeCN:CHCl_3_ = 4:1, 298 K, optical path: 2 mm) of **COR**, **3H-HBC**, **3Me-HBC**, **3F-HBC**, and their equimolar mixtures with **TPACage**•6PF_6_. **b** Emission spectra and fluorescent photographs (insets) (5 × 10^−6^ M, λ_ex_ = 302 nm for **COR**/384 nm for ***c*-HBC**, MeCN:CHCl_3_ = 4:1, 298 K) of **COR**, **3H-HBC**, **3Me-HBC**, and **3F-HBC** before (red) and after (brown) adding 4 equiv. of **TPACage**•6PF_6_.

UV-Vis absorption and fluorescence spectra of both the cage and guests exhibit marked changes in the formation of host-guest complexes. Upon addition of 1 equiv. of ***c-*HBC** guests to a MeCN/CHCl_3_ (4:1) solution of **TPACage**^6+^, the characteristic absorption peak (Fig. 5a) of **TPACage**^6+^ centered on 487 nm decreases and is accompanied by a redshift. The bright green fluorescence of ***c*-HBC** guests is almost fully quenched (Fig. 5b), while the fluorescence quantum yields for **3H-HBC**, **3Me-HBC**, and **3F-HBC** change (Supplementary Table 1) from 3.8% to 0.6%, 3.7% to 0.9%, and 3.7% to 0.3%, respectively. Meanwhile, the emission of ***c*-HBC** overlaps well (Fig. 5) with the absorption band of **TPACage**^6+^, enabling potential energy transfer from ***c*-HBC** to **TPACage**^6+^. In the UV-Vis absorption of **COR⊂TPACage**^6+^, the absorbance at 487 nm does not change much (Fig. 5a) compared with that of the free **TPACage**^6+^, because of the low binding affinity (*K*_a_ = 1.3 × 10^3^ M^−1^) between **COR** and **TPACage**^6+^. By contrast, on adding 4 equiv. of **TPACage**^6+^ to a solution of **COR**, the blue fluorescence of **COR** is almost completely quenched (Fig. 5b) with the fluorescence quantum yield decreasing (Supplementary Table 1) from 1.8 to 0.2%, an observation that can be attributed to (i) the formation of a host-guest complex, and (ii) the strong absorption of the excess of **TPACage**^6+^ present in the solution.

The changes in the absorption spectra induced by the formation of the host-guest complexes afford us an easy way to track the molecular recognition processes. The Job plots for **3H-HBC** (Supplementary Fig. 48), **3Me-HBC** (Supplementary Fig. 51), and **3F-HBC** (Supplementary Fig. 54) with **TPACage**^6+^ all show maxima at mole fractions of 0.5, confirming a stoichiometric ratio of 1:1 for all three complexes. The binding affinities between **TPACage**^6+^ and the different ***c*-HBC** guests were determined by UV-Vis titrations. The *K*_a_ value for the **3H-HBC⊂TPACage**^6+^ complex was determined (Supplementary Fig. 47) to be 1.7 × 10^5^ M^−1^ by following the change in absorbance at 487 nm. The Gibbs free energy (Δ*G*) was calculated (Table 1) to be −7.1 kcal mol^−1^, a value which is more negative than that (−4.2 kcal mol^−1^) for **COR⊂TPACage**^6+^ complex. It follows that the better the size matching between the host and guests, the stronger are the binding affinities. Because of the poor solubility of **3H-HBC** in MeCN/CHCl_3_ (4:1), it is difficult to obtain the *K*_a_ value for the **3H-HBC⊂TPACage**^6+^ complex using isothermal titration calorimetry (ITC). The change in enthalpy (Δ*H*) on complexing **3H-HBC** within **TPACage**^6+^ was estimated (Supplementary Fig. 62) to be −3.2 kcal mol^−1^ from an isotherm obtained from a single injection experiment^63^. The value of *T*Δ*S* was calculated (Table 1) to be 3.9 kcal mol^−1^, while the change in entropy (Δ*S*) is 13.1 cal mol^−1^ K^−1^. The increase in the entropy upon forming the **3H-HBC⊂TPACage**^6+^ complex can be attributed most likely to the desolvation of the guest and the expulsion of solvent molecules originally residing inside the cavity of the **TPACage**^6+^, a phenomenon similar to that observed in previous reports^57,64^. Using the same procedures, the *K*_a_ values for the **3Me-HBC⊂TPACage**^6+^ (Supplementary Fig. 50) and the **3F-HBC⊂TPACage**^6+^ (Supplementary Fig. 53) complexes were determined to be 2.4 × 10^5^ and 8.0 × 10^4^ M^−1^, respectively. The Δ*H* values for the formation of **3Me-HBC⊂TPACage**^6+^ (Supplementary Fig. 63) and **3F-HBC⊂TPACage**^6+^ (Supplementary Fig. 64) were estimated to be −3.5 and −2.4 kcal mol^−1^ by ITC, and the corresponding *T*Δ*S* values were calculated to be 3.8 and 4.3 kcal mol^−1^, respectively. It follows that the formation of the ***c*-HBC⊂TPACage**^6+^ complexes is driven collectively by favorable entropy and enthalpy changes. The binding constants and enthalpy changes between the ***c*-HBC** guests and **TPACage**^6+^ are arranged in the order **3Me-HBC** > **3H-HBC** > **3F-HBC**, indicating that, as the electron density of the guests increases, the binding affinities between the host and guests become stronger in the solution state. The kinetics associated with the encapsulations of the ***c*-HBC** guests by the **TPACage**^6+^ can be obtained^63^ by following the change in absorbance with time. The association rate constants (*k*_on_) for **TPACage**^6+^ with **3H-HBC**, **3Me-HBC**, and **3F-HBC** were found (Supplementary Figs. 59−61) to be 7.2 × 10^6^, 2.6 × 10^6^, and 3.2 × 10^6^ M^−1^ s^−1^, respectively. These *k*_on_ values indicate that, as the size of the guests increases, the rates of association between the **TPACage**^6+^ and the ***c*-HBC** guests decrease.

**Table 1:** *K*_a_ Values and thermodynamic parameters for the 1:1 complexes formed between TPACage·6PF_6_ and four coronene-based guests in MeCN / CHCl_3_ at 25 °C^a^.

Entry	Guest	*K*_a_ (M^−1^)	Δ*G* (kcal mol^−1^)	Δ*H* (kcal mol^−1^)	*T*Δ*S* (kcal mol^−1^)	Δ*S* (cal mol^−1^ K^−1^)
1	COR	1.3 × 10^3^	−4.2^b^	ND^d^	ND^d^	ND^d^
3	3H-HBC	1.7 × 10^5^	−7.1^c^	−3.2^e^	3.9	13.1
2	3Me-HBC	2.4 × 10^5^	−7.3^c^	−3.5^e^	3.8	12.8
4	3F-HBC	8.0 × 10^4^	−6.7^c^	−2.4^e^	4.3	14.4

^a^Standard errors are presented in the Supplementary Information. ^b^Estimated from 1H NMR titrations. ^c^Estimated from UV-Vis titrations. ^d^Not determined. ^e^Directly determined by ITC.

### Solid-state superstructures of the host-guest complexes

**Fig. 6:**
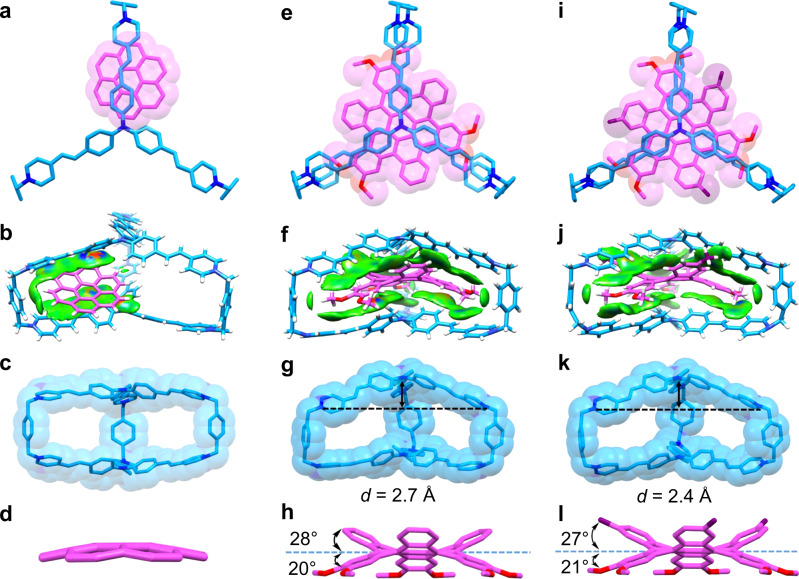
Solid-state superstructures of the host-guest complexes. **a**, **b** Capped-stick and space-filling representations of the solid-state superstructure and the intermolecular binding iso-surface of **COR⊂TPACage**^6+^. **c**, **d** Capped-stick and space-filling representations of the corresponding solid-state structures of individual **TPACage**^6+^ and **COR** molecules in their 1:1 complex. **e, f** Capped-stick and space-filling representations of the solid-state superstructure and intermolecular binding iso-surface of **3H-HBC⊂TPACage**^6+^. **g, h** Capped-stick and space-filling representations of the corresponding solid-state structures of individual **TPACage**^6+^ and **3H-HBC** molecules in their 1:1 complex, showing the characteristic parameters defining the changes in their geometries. **i**, **j** Capped-stick and space-filling representations of the solid-state superstructure and intermolecular binding iso-surface of **3Me-HBC⊂TPACage**^6+^. **k**, **l** Capped-stick and space-filling representations of the corresponding solid-state structures of individual **TPACage**^6+^ and **3Me-HBC** molecules in their 1:1 complex, showing the characteristic parameters defining the changes in their geometries. Solvent molecules and counterions have been omitted for the sake of clarity. C skyblue, pink and purple, H white, O red, N blue.

In order to elucidate the binding modes of **TPACage**^6+^ toward **COR** and ***c*-HBC** guests in the solid state, their complexes were analyzed by single-crystal X-ray diffraction. Dark red single crystals of the 1:1 complex between **TPACage**^6+^ and **COR** were obtained by slow vapor diffusion of *i*Pr_2_O into MeOH / CHCl_3_ (4:1) solution of **TPACage**•6Cl containing an excess of **COR** over a period of three days. Single-crystal X-ray diffraction analysis revealed that the **COR** is positioned (Fig. 6a) closer to one of the *p*-xylylene linkers in **TPACage**^6+^, rather than in the center of the cavity. Independent gradient model (IGM) analysis revealed that (Fig. 6b) the binding mode is sustained by [π···π] interactions between the electron-rich **COR** and the electron-deficient pyridinium units on the side of the cage, as well as by a [C−H···π] interaction between a hydrogen atom on **COR** and the nearby *p*-xylylene linker with a distance of 3.1 Å. Notably, the **COR** guest is disordered (Supplementary Fig. 65) among six different positions with an average occupation of one-sixth in the cavity of the cage. The **COR** guest is not only able to reside (Supplementary Fig. 65a) in any one of three slots formed by three pairs of styrene-pyridinium units, but it also occupies (Supplementary Fig. 65b) two positions in each slot. These observations indicate that **COR** is not large enough to occupy the entire cavity in the cage.

Single crystals of **3H-HBC⊂TPACage**^6+^ complex were obtained after numerous attempts by slow vapor diffusion of *i*Pr_2_O into MeNO_2_ / CHCl_3_ (4:1) solution containing equimolar amounts of **TPACage**•6AsF_6_ and **3H-HBC** over three days. In the solid-state superstructure of **3H-HBC⊂TPACage**^6+^, **3H-HBC** is located (Fig. 6e) in the center of the cavity inside **TPACage**^6+^, forming a *C*_3_-symmetrical host-guest complex. Three 1,2-dimethoxybenzene groups in **3H-HBC** are located in the slots defined by three pairs of styrene-pyridinium units, which are stabilized by intermolecular [π···π] and [C−H···π] interactions with average distances of 3.7 Å and 3.2 Å, respectively. The outer benzenoid rings in **3H-HBC** reside in the spaces between two styrene-pyridinium units in the cage. The reasons for this particular binding mode are (i) the [C−H···π] interaction (Fig. 6f) between the hydrogens attached to methoxy groups on the **3H-HBC** and *p*-xylylene linkers of the cage, and (ii) the fact that 1,2-dimethoxybenzene groups are (Fig. 3f) more electron-rich than the outer benzenoid rings in **3H-HBC**. The conformation of **3H-HBC** undergoes (Fig. 6h) remarkable changes following inclusion inside the cavity of **TPACage**^6+^. The average tilt angle between the 1,2-dimethoxybenzene groups and the central benzene ring is 20°, while that between the outer benzenoid rings and the central benzene ring is 28°. These two tilt angles are different from those (26 and 24°) in the free **3H-HBC** (Fig. 3e), on account of the tight encapsulation of **3H-HBC** by **TPACage**^6+^. The additional strain energy for **3H-HBC** in the host-guest complex is (Supplementary Table 4) 2.1 kcal mol^−1^ according to DFT calculations. The **TPACage**^6+^ also becomes deformed in order to accommodate the twisted geometry of **3H-HBC**. It changes (Fig. 6g) to a yurt-like shape, in which one of the TPAP units bulges outwards. These observations are reminiscent of the induced fit of enzymes with respect to their substrates in biological systems^65,66^.

Using the same procedure, the crystal superstructure of **3Me-HBC⊂TPACage**^6+^ was obtained. The binding mode (Fig. 6i−6 l) of **3Me-HBC** inside the cavity of **TPACage**^6+^ is almost the same as that of **3H-HBC**, indicating that the yurt-like binding mode of complexes is thermodynamically stable in the solid state. The **3Me-HBC** guest also undergoes distortion within the cavity of **TPACage**^6+^. Its strain energy is (Supplementary Table 4) 2.3 kcal mol^−1^, a value which is close to that (2.1 kcal mol^−1^) of **3H-HBC** in its host-guest complex.

### Gas-phase stability of the host-guest complexes

**Fig. 7:**
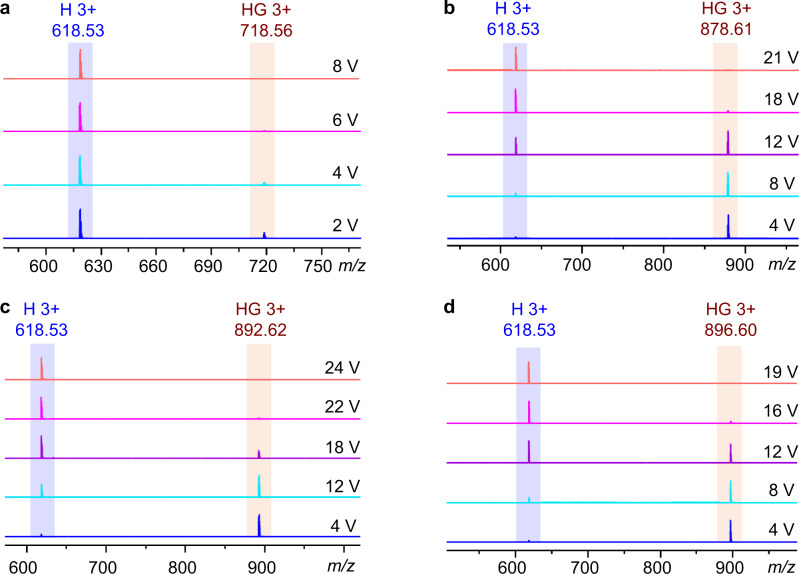
Gradient tandem mass spectra (gMS^2^) of four host-guest complexes. The gMS^2^ of (**a**) [**COR⊂TPACage**•3PF_6_]^3+^, (**b**) [**3H-HBC⊂TPACage**•3PF_6_]^3+^, (**c**) [**3Me-HBC⊂TPACage**•3PF_6_]^3+^, and (**d**) [**3F-HBC⊂TPACage**•3PF_6_]^3+^, showing that the signal intensity for complexes gradually decreases as the voltage is increased. Blue “H 3+” represents the three positively charged states of the free **TPACage**•6PF_6_, while brown “HG 3+” represents the three positively charged states of the host-guest complexes.

In addition, the stability of these four complexes in the gas phase was investigated in-depth by gradient tandem mass spectrometry (gMS^2^). The 3+ peaks for the complexes (Fig. 7), e.g., *m/z* = 718.56 for [**COR⊂TPACage**•3PF_6_]^3+^, 878.61 for [**3H-HBC⊂TPACage**•3PF_6_]^3+^, 892.62 for [**3Me-HBC⊂TPACage**•3PF_6_]^3+^, and 896.60 for [**3F-HBC⊂TPACage**•3PF_6_]^3+^, were selected as parent ions, which were isolated using a quadrupole, followed by disintegration in the trap cell through collision-induced dissociation (CID) while the trap voltage was gradually increased. As the voltages increase, the signal intensities for the complexes gradually decrease (Fig. 7), accompanied by the generation of a peak at *m/z* = 618.53, which can be assigned to the free [**TPACage**•3PF_6_]^3+^. The [**3Me-HBC⊂TPACage**•3PF_6_]^3+^ complex dissociates (Fig. 7c) fully at a trap voltage of 24 V, corresponding to a center-of-mass collision energy of 1.06 eV, which is the highest one among the four complexes. In sharp contrast, the [**COR⊂TPACage**•3PF_6_]^3+^ complex dissociates (Fig. 7a) completely at a low trap voltage of 8 V, corresponding to a center-of-mass collision energy of 0.44 eV. The collision energies for dissociating the [**3H-HBC⊂TPACage**•3PF_6_]^3+^ (Fig. 7b) and [**3F-HBC⊂TPACage**•3PF_6_]^3+^ (Fig. 7d) complexes were calculated to be 0.94 eV and 0.83 eV, respectively. The stability of the four complexes in the gas phase can be ranked **3Me-HBC** > **3H-HBC** > **3F-HBC≫** **COR** according to the collision energies, a trend which is consistent with the order of binding constants (Table 1) obtained in solution.

### DFT calculations

In order to probe the origins of the changes in photophysical properties and gain insights into the electronic properties of these four host-guest complexes, density functional theory (DFT) calculations were performed. The optimized superstructures (Supplementary Figs. 76−77) of **COR⊂TPACage**^6+^, **3H-HBC⊂TPACage**^6+^, and **3Me-HBC⊂TPACage**^6+^ are good matches with those obtained from single-crystal X-ray diffraction. Frontier molecular orbital (FMO) analyses show (Supplementary Fig. 76a) that both the highest occupied molecular orbital (HOMO) and lowest unoccupied molecular orbital (LUMO) of **COR⊂TPACage**^6+^ complex are localized on **TPACage**^6+^, a situation which is different from the FMOs (Supplementary Figs. 76−77) for the complexes between the **TPACage**^6+^ and ***c*-HBC** guests. The HOMOs for the ***c*-HBC⊂TPACage**^6+^ complexes are localized predominantly on the ***c*-HBC** guests, while the LUMOs are confined mainly to **TPACage**^**6+**^. The HOMO−LUMO energy gaps in MeCN (Supplementary Table 3) for the **COR**, **3H-HBC**, **3Me-HBC** and **3F-HBC** guests, and **TPACage**^6+^ host are 4.07, 3.22, 3.20, 3.19, and 2.48 eV, respectively, while their corresponding host-guest complexes possess contracted energy gaps with the values of 2.44, 2.30, 2.24, and 2.32 eV, respectively. These narrowed energy gaps provide the internal reason for the red-shifted absorption spectra in the case of both the host and guests upon forming complexes. Electrostatic potential analyses revealed that the electron density in the original electron-rich guests decreases sharply (Supplementary Figs. 79−80) following complexation by **TPACage**^**6+**^, suggesting the presence of the intermolecular electron delocalization. The host-guest binding energies were also estimated by DFT calculations. The calculated binding energies in vacuum (Supplementary Table 5) between **TPACage**^**6+**^ and four different guests are arranged in the order of |Δ*E*_**3Me-HBC**_| > |Δ*E*_**3H-HBC**_| > |Δ*E*_**3F-HBC**_| ≫ |Δ*E*_**COR**_|, a trend which is consistent with the sequence of Gibbs free energies in Table 1.

### Guest protection within the cage

**Fig. 8:**
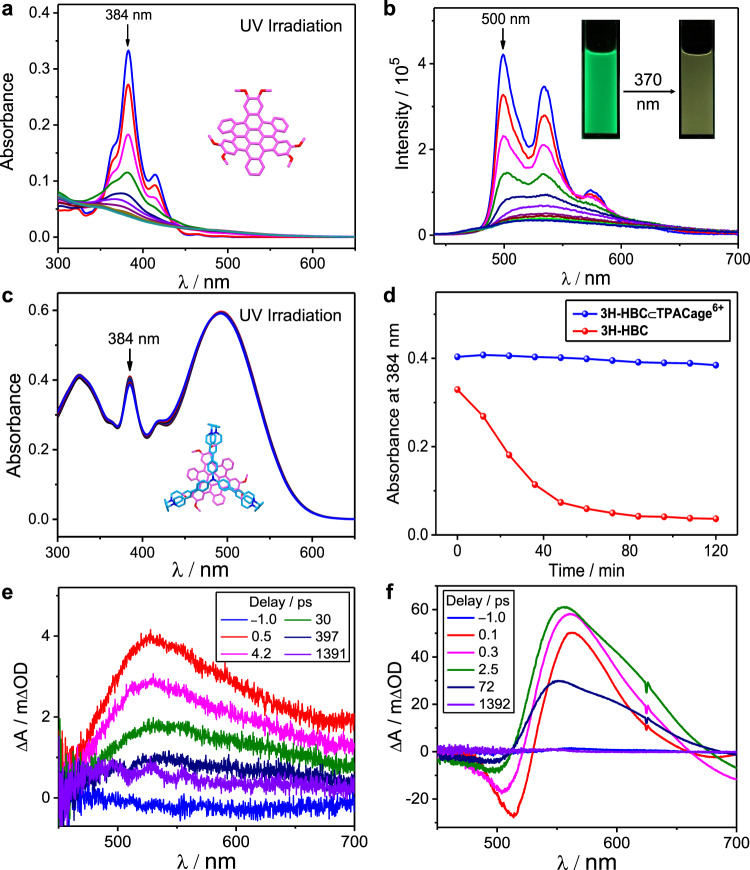
Investigation of photostability. **a** Changes in UV−Vis absorption spectra of **3H-HBC** upon irradiation with UV light (370 nm, 120 min). **b** Changes in emission spectra (λ_ex_ = 384 nm) and fluorescent photographs (*inset*) of **3H-HBC** upon irradiation with UV light (370 nm, 120 min). **c** Changes in UV−Vis absorption spectra of **3H-HBC⊂TPACage**•6PF_6_ upon irradiation with UV light (370 nm, 120 min). **d** Changes in absorbance at 384 nm of **3H-HBC** (red) and **3H-HBC⊂TPACage**•6PF_6_ (blue) upon irradiation with UV light (370 nm, 120 min). Femtosecond transient absorption spectra of (**e**) **3H-HBC** excited at 350 nm, and (**f**) **3H-HBC**⊂**TPACage**•6PF_6_ excited at 350 nm.

Upon exposing a **3H-HBC** suspension to UV light (370 nm), we found, quite accidentally, that the suspension turned into a clear solution. In order to gain a better understanding of this phenomenon, UV-Vis absorption and fluorescence spectroscopic measurements were carried out. Upon irradiation of a 0.5 mM **3H-HBC** solution with UV light (370 nm), the UV-Vis absorption spectra of its dilute solution revealed that the absorption peak centered on 384 nm decreases gradually (Fig. 8a). After irradiation for 120 min, the UV-Vis absorption spectrum no longer undergoes any change, and the absorbance at 384 nm is decreased (Fig. 8d) by 89%. During the irradiation, no obvious new peak appears in the UV-Vis absorption spectra, indicating that the **3H-HBC** may be undergoing degradation when exposed to UV light. The fluorescence of **3H-HBC** also decreases dramatically (Fig. 8b), and its bright green fluorescence turns to a pale yellow when exposed to UV light for 120 min. These observations confirm the photo-degradation process of **3H-HBC**. Upon adding 2 equiv. of **TPACage**^6+^ to a MeCN/CHCl_3_ (4:1) solution containing 0.5 mM **3H-HBC**, we estimate that 99% of the **3H-HBC** guest is encapsulated inside the cavity of the cage according to the association and disassociation equilibria. When this solution is irradiated with UV light (370 nm) for 120 min, the UV-Vis absorption spectra of its dilute solution show (Fig. 8c) slight changes. The characteristic absorption peak of **3H-HBC** at 384 nm decreases (Fig. 8d) by only 4.7%. Similarly, when solutions of **3Me-HBC⊂TPACage**^6+^ and **3F-HBC⊂TPACage**^6+^ are irradiated with UV light (370 nm, 120 min), the characteristic absorption peak of the **3Me-HBC** guest at 384 nm decreases (Supplementary Figs. 83−84) by 8.4%, while the absorbance of the **3F-HBC** guest at 384 nm shows no appreciable decline (Supplementary Figs. 85−86). It follows that the **TPACage**^6+^ acts as a protective shield, reducing the photo-degradation rates of the ***c*-HBC** guests to a considerable degree. When the irradiation times for the host-guest complexes were extended to 240 min, the characteristic absorption peaks of **3H-HBC** and **3Me-HBC** at 384 nm decreased (Supplementary Fig. 87) by 13.7 and 15.7%, respectively, while the absorbance of **3F-HBC** at 384 nm exhibited very little change (Supplementary Fig. 87). The slow photo-degradation of the ***c*-HBC** guests after long periods of irradiation can be attributed to the dynamic and reversible nature of the host-guest complexes and the slight photodamage of the host. Possible reasons for the difference in the photo-degradation rate between the three ***c*-HBC** guests in the host-guest complexes could be the fact that (i) the different guests display (Supplementary Figs. 59−61) different association/dissociation binding kinetics toward the **TPACage**^6+^, and (ii) the intrinsic photostability of the three guests is different (Supplementary Figs. 88−89). The good photostability of the **3F-HBC**⊂**TPACage**^6+^ complex may result from the relatively slow photolysis rate of the **3F-HBC**, and ideal association/dissociation exchange kinetics of the **3F-HBC⊂TPACage**^6+^ complex in solution.

In order to ascertain the underlying mechanism giving rise to the additional photostability of ***c***-**HBC** provided by encapsulation within **TPACage**^6+^, femtosecond transient absorption (fsTA) measurements were carried out to probe the photo-induced dynamics of **3H-HBC** and **3H-HBC⊂TPACage**^6+^ complex. The fsTA spectra of **3H-HBC** exhibit (Fig. 8e) a broad excited-state absorption feature at 525 nm when excited with a 350-nm pump pulse. Global analysis reveals (Supplementary Fig. 91) that the singlet excited-state (S_1_) lifetime of **3H-HBC** is ∼100 ps, preceded by a 6.2-ps relaxation attributed to vibrational relaxation within the initial S_1_ excited state. The fsTA spectra of **TPACage**^6+^ show (Supplementary Fig. 90a) a strong excited-state absorption transition centered at 560 nm upon excitation of its main absorption peak at 500 nm. When exciting the **3H-HBC⊂TPACage**^6+^ at 350 nm, its fsTA spectra (Fig. 8f) do not show the S_1_ excited-state signature of **3H-HBC**, while only the spectral features of the **TPACage**^6+^ are immediately observed. The excited-state dynamics of **3H-HBC⊂TPACage**^6+^ (Fig. 8f and Supplementary Fig. 92) are analogous to those of the free **TPACage**^6+^ (Supplementary Fig. 90) excited at 500 nm, and lacking the 6.2-ps relaxation of **3H-HBC**. These observations suggest the presence of ultrafast energy transfer from **3H-HBC** to **TPACage**^6+^ upon excitation of the **3H-HBC**⊂**TPACage**^6+^ complex, and the cage acts to quench the excited state of **3H-HBC** in this process. The corresponding timescale of the energy transfer is estimated to be <0.3 ps (the fsTA instrument time resolution), a value which is much less than the excited-state lifetime (100 ps) of the free **3H-HBC**. This ultrafast deactivation pathway (Supplementary Fig. 93) of the **3H-HBC** S_1_ excited state can suppress significantly the photo-induced degradation of **3H-HBC** in the excited state. Consequently, the photostability of the ***c*-HBC** guests is enhanced on complexation inside the cavity of the **TPACage**^6+^.

## Discussion

A trigonal prismatic hexacationic cage, **TPACage**^6+^, with a well-defined cavity and a relatively flexible conformation, has been designed and synthesized. The cage is able to encapsulate both planar coronene and contorted hexabenzocoronene guests with favorable changes in both enthalpy and entropy. As a result of the ideal dimensional matching, the binding affinities between the **TPACage**^6+^ and the contorted hexabenzocoronene guests are larger than that in the case of coronene. To the best of our knowledge, the contorted hexabenzocoronene is the largest nanographene guest which has been investigated in the context of host-guest chemistry. Encapsulating the contorted hexabenzocoronene inside the cage, not only enriches host-guest chemistry^67,68^ but also extends the potential applications of synthetic receptors when it comes to the separation and stabilization of nanographenes. It is worthy of note that, benefiting from the ultrafast deactivation of the excited state of the hexabenzocoronene by transferring energy to the **TPACage**^6+^, the photostability of the hexabenzocoronene guests is improved significantly. Enhancing the photostability of the ***c*-HBC⊂TPACage**^6+^ complexes constitutes a good example of regulating the photo-reaction pathway in nanographenes by a non-covalent strategy. We anticipate these complexes will serve as promising building blocks for the construction of nanographene-based mechanically interlocked molecules^69^ and new kinds of photostable synthetic materials^64,70^ that combine the properties of graphene derivatives and wholly synthetic receptors.

## Methods

### Materials

All reagents were purchased from commercial suppliers and used without further purification unless stated otherwise. The synthesis of **TPACage**^**6+**^ is shown in Supplementary Figs. 1−3. Coronene, defined as **COR**, was purchased from Sigma-Aldrich. The hexa-*cata*-hexabenzocoronene guests, defined as ***c*-HBC**, were prepared according to the literature procedure with some modifications as shown in Supplementary Fig. 4.

### NMR measurements

NMR spectra were recorded on a Bruker Avance III 600 MHz, Agilent 500 MHz, and Bruker Avance III 400 MHz spectrometers in CD_3_CN, CDCl_3_, CD_3_OD or their mixed solvents, with working frequencies of 600, 500, and 400 MHz for recording ^1^H NMR spectra, and 150, 125 and 100 MHz for recording ^13^C NMR spectra, respectively. Chemical shifts (δ) are given in ppm with residual solvent signals as references.

### Photophysical characterization

Both UV-Vis absorption and fluorescence spectroscopic experiments were performed at 298 K in MeCN / CHCl_3_ (4:1). UV-Vis Absorption spectra were recorded on a UV-3600 Shimadzu spectrophotometer in three types of rectangular quartz cells with the light paths of 10 mm, 4 mm, and 2 mm, respectively. Fluorescence spectra were measured in two types of rectangular quartz cells, with the light paths of 10 mm and 2 mm, respectively, on JASCO FP-2 750 spectrometer or HORIBA FluoroMax-4 spectrometer, which were equipped with an integrating sphere for absolute fluorescence quantum yields determination.

### High-resolution and gradient tandem mass spectrometry

High-resolution mass spectra (HRMS) for the precursors were recorded on an Agilent 6210 Time-of-Flight (TOF) LC-MS with an ESI source. HRMS and gradient tandem mass spectra for cage and host-guest complexes were recorded on a Waters Synapt G2-Si mass spectrometer equipped with ion-mobility under the following conditions: ESI Capillary voltage, 1.0 kV; sample cone voltage, 0 V; source offset, 1.0 V; source temperature, 90 °C; desolvation temperature, 170 °C; cone gas flow, 10 L h^−1^; desolvation gas flow, 200 L h^−1^ (N_2_).

### UV–Vis titration

A 1 mM solution of ***c*-HBC** guest molecules in CHCl_3_ as the titrating solution was added dropwise to a micromolar solution of **TPACage**•6PF_6_ in MeCN / CHCl_3_ (4:1). Spectra were recorded from 700 to 400 nm in 10 × 10 × 45 mm rectangular quartz cells. Binding constants were obtained by fitting a 1:1 isotherm according to the programs available at http://app.supramolecular.org/bindfit/.

### ITC titration

All microcalorimetric titrations were performed using a thermostated TA Nano Isothermal Titration Calorimeter at atmospheric pressure and 298 K. The samples were dissolved in a solution of MeCN / CHCl_3_ (4:1) and allowed to equilibrate overnight before use. A solution of **TPACage•**6PF_6_ (3 × 10^−3^ M) in a syringe was injected with stirring at 75 rpm into a solution of ***c*-HBC** (3 × 10^−4^ M) guests in the sample cell with an active volume of 185 µL. Hindered by (i) the relatively low solubility of ***c*-HBC** guests in MeCN/CHCl_3_ (4:1) and (ii) the small enthalpy changes upon forming the complexes, we found it is difficult to obtain accurate binding constants using a continuous calorimetric titration protocol after multiple attempts. We explored the independent single-injection experiments to estimate the binding enthalpies for the formation of host-guest complexes. The net reaction heat was obtained by subtracting the dilution heat from the apparent reaction heat.

### Crystallizations and X-ray analyses for all complexes

For **TPACage**•6Cl: Dark red single crystals were obtained by slow vapor diffusion of *i*Pr_2_O into a 1.0 mM solution of **TPACage**•6Cl in MeOH over the course of four days. For **3H-HBC**: Yellow single crystals were obtained by slow evaporation of the PhMe / CHCl_3_ (4:1) solution of **3H-HBC** over the course of five days. For **COR⊂TPACage**•6Cl complex: Dark red single crystals were obtained by slow vapor diffusion of *i*Pr_2_O into a MeOH / CHCl_3_ (4:1) solution of **TPACage**•6Cl containing excess amounts of **COR** over the course of three days after numerous attempts. For ***c*-HBC⊂TPACage**•6AsF_6_ complexes: Dark red single crystals were obtained by slow vapor diffusion of *i*Pr_2_O into a MeCN / CHCl_3_ (4:1) solution of equimolar amounts of **TPACage**•6AsF_6_ and ***c*-HBC** over the course of three days after numerous attempts. The suitable crystals, which appeared in the tubes, were mounted on a MITIGEN holder in Paratone oil on a Bruker Kappa APEX2 CCD or a Rigaku XtaLAB Synergy diffractometer using CuKα radiation (*λ* = 1.5407 Å). Data were collected using the Bruker APEX-II or Rigaku CrysAlis Pro program. The structures were solved with the ShelXT program using intrinsic phasing and refined with the ShelXL refinement package using least-squares minimization in OLEX2 software.

### Irradiation experiments

Two rectangular quartz cells, containing ***c*-HBC** ([***c*-HBC**] = 0.5 mM), and ***c*-HBC** with 2 equiv. of **TPACage**^**6+**^ ([***c*-HBC**] = 0.5 mM, [**TPACage**^**6+**^] = 1 mM), respectively, were exposed simultaneously to UV light (370 nm) in MeCN / CHCl_3_ (4:1) solution. The UV-Vis absorption spectra were recorded by diluting the irradiated solution to 10 μM in a 10 × 2 × 45 mm quartz cell every 12 min. The emission spectra were recorded by diluting the irradiated solution to 0.4 μM in a 10 × 10 × 45 mm quartz cell every 12 min. All the UV-light irradiation experiments were carried out using a Kessil PR160−370 nm LED light source at the power density of 5.7 mW/cm^2^, and a Dewar bottle served as concentrating equipment.

### Transient absorption measurements

For the ultrafast transient absorption measurements, pump and probe pulses were generated from the 800-nm output of a commercial Ti: sapphire laser (800 nm, 100 fs pulse duration, Coherent Libra, 4 W). A portion of the 800-nm output was directed into a commercial optical parametric amplifier (OPA, Coherent TOPAS C) to generate a near-IR signal at either 1400 nm or 1333 nm. Pump pulses at 350 nm were generated from the fourth-harmonic generation of the OPA signal at 1400 nm. Pump pulses at 500 nm were generated through the sum-frequency generation of the OPA signal at 1333 nm with residual 800 nm fundamental. White-light continuum probe pulses were generated by focusing a portion of the 800-nm laser output into a 3-mm thick sapphire plate. For the experiments, the energy of the actinic pump pulse was attenuated to ~0.2 μJ/pulse. The polarization of the pump pulse was set to a magic angle relative to that of the probe to remove rotational contributions from the signal. The delay time between the pump and probe pulse was stepped from −1 ps to 1.3 ns in varying step sizes using a computer-controlled delay stage (Newport ILS250cc, XPS Q8). The probe pulse was spectrally resolved with a spectrometer (Andor Shamrock 500i) and the spectra were collected by a CCD camera (Andor Newton EMCCD: DU970P-FI).

## Supplementary information

Supplementary Information

Description of Additional Supplementary Files

Supplementary Data 1

## Data Availability

All the data supporting the conclusions are included in this article and its Supplementary files, or are available from the authors upon reasonable request. The X-ray crystallographic coordinates (Supplementary Data 1) for **TPACage•6Cl**, **3H-HBC**, **COR⊂TPACage•6Cl**, **3H-HBC⊂TPACage•6AsF**_**6**_, and **3Me-HBC⊂TPACage•6AsF**_**6**_, reported in this study, have all been deposited at the Cambridge Crystallographic Data Centre (CCDC). The deposition numbers are CCDC 2045289, 2045282, 2045290, 2044797, and 2045291, respectively. These data can be obtained free of charge from The Cambridge Crystallographic Data Centre via www.ccdc.cam.ac.uk/data_request/cif. The Cartesian coordinates (Supplementary Data 1) for optimized structures are included in an individual Supplementary PDF file.
