# Supplementary material for: A contorted nanographene shelter
Source: Nat Commun. 2021 Aug 31;12:5191. doi: 10.1038/s41467-021-25255-6 (PMC8408160; doi:10.1038/s41467-021-25255-6)
Supplement: Supplementary file 1 — Supplementary Information [file 41467_2021_25255_MOESM1_ESM.pdf]

Supplementary Information for

A Contorted Nanographene Shelter

Huang Wu[1], Yu Wang[1], Bo Song[1], Hui-Juan Wang[2], Jiawang Zhou[3], Yixun Sun[4], Leighton O. Jones[1], Wenqi Liu[1], Long Zhang[1], Xuan Zhang[1], Kang Cai[2], Xiao-Yang Chen[1], Charlotte L. Stern[1], Junfa Wei[4], Omar K. Farha[1], Jessica M. Anna[3], George C. Schatz[1], Yu Liu[2,6]*, and J. Fraser Stoddart[1,5,7,8]*

[1]*Department of Chemistry, Northwestern University, 2145 Sheridan Road, Evanston, Illinois 60208, United States*

[2]*College of Chemistry, State Key Laboratory of Elemento-Organic Chemistry, Nankai University, 94 Weijin Road, Nankai District, Tianjin 300071, P.R. China*

[3]*Deparment of Chemistry, University of Pennsylvania, 231 South 34th Street, Philadelphia, Pennsylvania 19104, United States.*

[4]*Key Laboratory of Applied Surface and Colloid Chemistry (Ministry of Education), Key Laboratory for Macromolecular Science of Shaanxi Province, School of Chemistry and Chemical Engineering, Shaanxi Normal University, Xi'an 710119, P. R. China*

[5]*School of Chemistry, University of New South Wales, Sydney, NSW 2052, Australia*

[6]*Collaborative Innovation Center of Chemical Science and Engineering (Tianjin), 92 Weijin Road, Nankai District, Tianjin 300072, P. R. China*

[7]*Stoddart Institute of Molecular Science, Department of Chemistry, Zhejiang University, Hangzhou 310027, China.*

[8]*ZJU-Hangzhou Global Scientific and Technological Innovation Center, Hangzhou 311215, China.*

*E-mail: yuliu@nankai.edu.cn * E-mail: stoddart@northwestern.edu

Supplementary Note 1. Synthetic Protocols

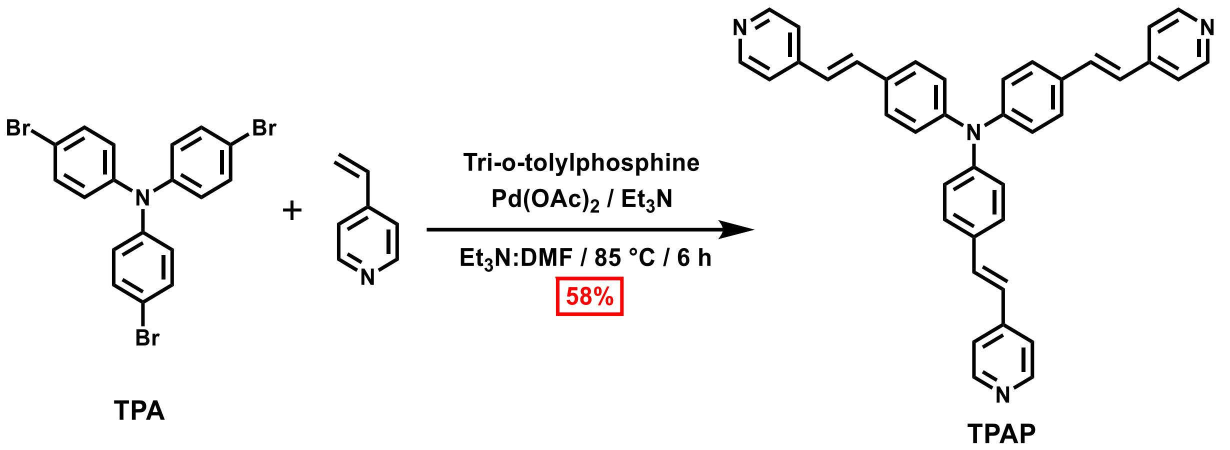

Supplementary Figure 1. Synthesis of **TPAP**

TPAP: Palladium acetate (224 mg, 1 mmol) and tris-o-tolylphosphine (609 mg, 2 mmol) were dissolved in a solution of dry and degassed Et_3N / DMF (300 mL, 2:1, v/v), and the mixture was stirred under N_2 for 15 min. Then, tris(4-bromophenyl)amine (4.82 g, 10 mmol) and 4-vinylpyridine (6.47 mL, 60 mmol) were added under a N_2 atmosphere. The resulting solution was stirred at 85 ºC under N_2 for 6 h. The resulting mixture was cooled to room temperature, and the solvent was removed under vacuum. The residue was dissolved in CH_2Cl_2 and washed with saturated $NaHCO_3$ and H_2O. The organic phase was concentrated and purified by column chromatography using CH_2Cl_2 / MeOH (20:1) as eluent to afford **TPAP** as a yellow solid (yield: 58%). ^{1}H NMR (500 MHz, $CDCl_3$) δ 8.57 (d, J = 5.7 Hz, 6H), 7.47 (d, J = 8.6 Hz, 6H), 7.35 (d, J = 5.9 Hz, 6H), 7.29 (d, J = 16.3 Hz, 3H), 7.14 (d, J = 8.5 Hz, 6H), 6.94 (d, J = 16.3 Hz, 3H). ^{13}C NMR (100 MHz, $CDCl_3$) δ 150.3, 147.4, 144.9, 132.5, 131.5, 128.3, 125.0, 124.5, 120.8. HRMS-ESI for **TPAP**; Calcd for $C_{39}H_{30}N_4$: m/z = 555.2549 $[M + H]^+$; Found: 555.2554 $[M + H]^+$.

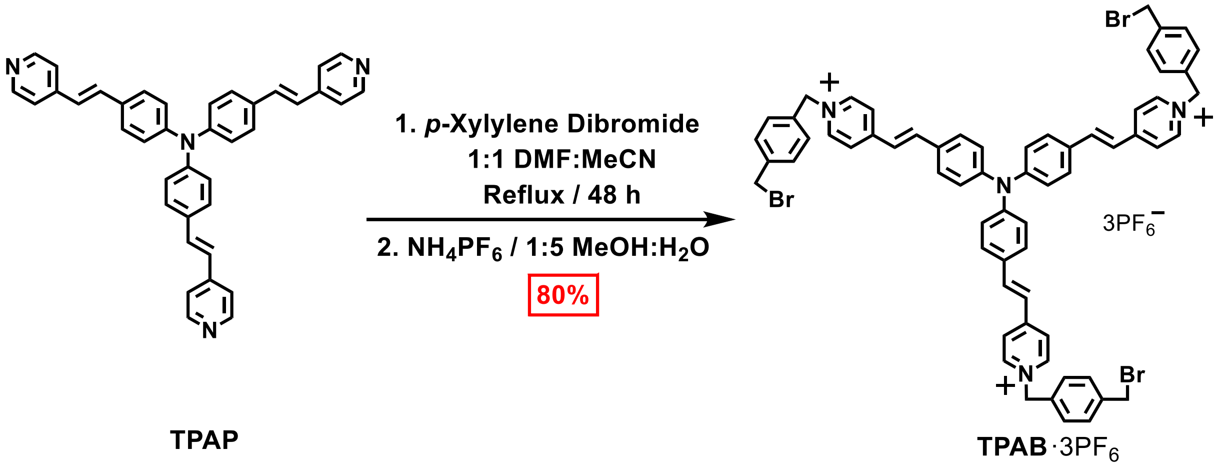

Supplementary Figure 2. Synthesis of **TPAB**·$3PF_6$

TPAB•$3PF_6$: *p*-Xylylene dibromide (4.96 g, 18.9 mmol) and **TPAP** (0.700 g, 1.26 mmol) were dissolved into a 1:1 mixture of DMF / MeCN (100 mL), and refluxed under N_2 atmosphere. After 48 h, the reaction mixture was cooled down to room temperature and poured into Et_2O (500 mL). The red precipitate was collected by filtration and washed with CH_2Cl_2 (300 mL). Subsequently, the red solid was dissolved in MeOH (100 mL), followed by the addition of NH_4PF_6 (1.85 g) and H_2O (1 L). The resulting precipitate was filtered, washed with H_2O (2 × 30 mL) and dried under vacuum to afford pure **TPAB•**$3PF_6$ (yield: 80%). ^{1}H NMR (500 MHz, CD_3CN) δ 8.54 (d, *J* = 6.9 Hz, 6H), 8.00 (d, *J* = 6.9 Hz, 6H), 7.82 (d, *J* = 16.3 Hz, 3H), 7.70 (d, *J* = 8.7 Hz, 6H), 7.55 (d, *J* = 8.2 Hz, 6H), 7.43 (d, *J* = 8.2 Hz, 6H), 7.29 (d, *J* = 16.3 Hz, 3H), 7.22 (d, *J* = 8.7 Hz, 6H), 5.61 (s, 6H), 4.62 (s, 6H). ^{13}C NMR (100 MHz, CD_3CN) δ 155.4, 149.5, 144.7, 142.1, 141.0, 134.5, 131.7, 131.0, 130.8, 130.2, 125.6, 125.0, 122.5, 63.7, 33.6. HRMS-ESI for **TPAB•**$3PF_6$; Calcd for $C_{63}H_{54}Br_3F_{18}N_4P_3$: *m/z* = 367.7291 $[M - 3PF_6]^{3+}$; Found: 367.7297 $[M - 3PF_6]^{3+}$.

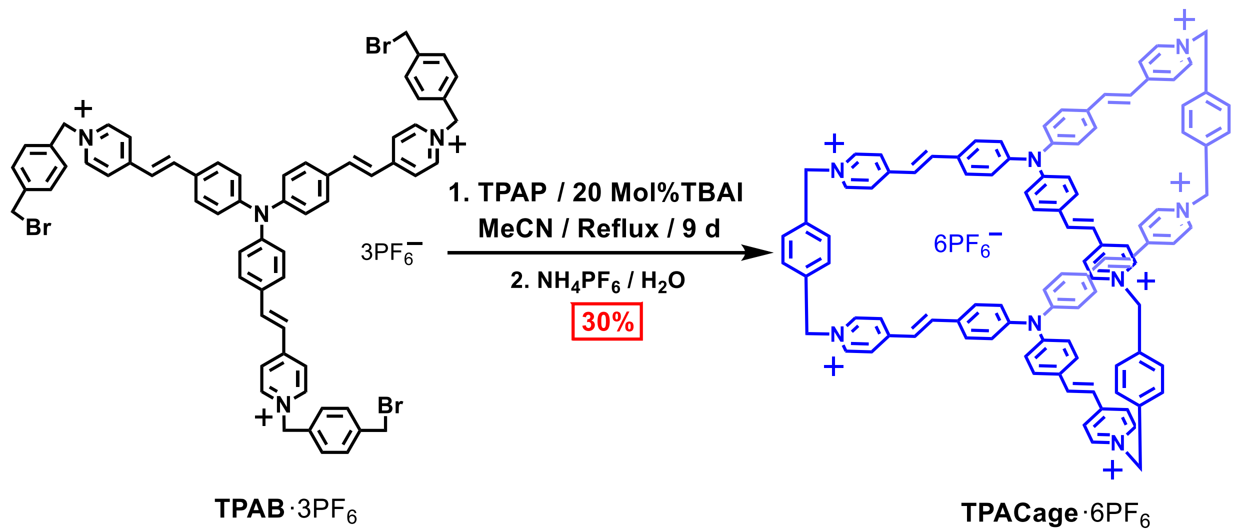

Supplementary Figure 3. Synthesis of **TPACage**·$6PF_6$

TPACage•$6PF_6$: A solution of **TPAB•**$3PF_6$ (2.50 g, 1.62 mmol), **TPAP** (0.900 g, 1.62 mmol), and TBAI (0.120 g, 0.325 mmol) in dry MeCN (810 mL) was heated under reflux at 90 ºC for 9 days. After cooling to room temperature, excess of TBACl was added to quench the reaction and the crude product was collected by filtration. The crude product was subjected to reverse-phase C18 column chromatography, starting with H_2O / 0.1 % TFA as eluent, followed by continuous addition of MeCN up to an eluent mixture of 99.9 % MeCN / 0.1% TFA over the course of 60 min. The fractions containing the desired product were combined, and followed by removal of MeCN by

rotary evaporation under vacuum. The residue was then treated with an excess of NH_4PF_6, resulting in a red precipitate, which was separated by filtration and dried under vacuum yielding **TPACage**•$6PF_6$ (yield: 30%). ^1H NMR (500 MHz, CD_3CN) δ 8.55 (d, J = 6.7 Hz, 12H), 7.89 (d, J = 6.7 Hz, 12H), 7.69 (d, J = 16.3 Hz, 6H), 7.61 (s, 12H), 7.59 (d, J = 8.6 Hz, 12H), 7.17 (d, J = 16.3 Hz, 6H), 7.11 (d, J = 8.8 Hz, 12H), 5.58 (s, 12H). ^{13}C NMR (100 MHz, CD_3CN) δ 155.3, 149.4, 144.2, 142.1, 137.0, 131.6, 131.1, 130.7, 125.4, 125.0, 122.3, 64.1. HRMS-ESI for **TPACage**•$6PF_6$; Calcd for $C_{102}H_{84}F_{36}N_8P_6$: m/z = 618.5248 $[M - 3PF_6]^{3+}$; Found: 618.5258 $[M - 3PF_6]^{3+}$.

TPACage•6 Cl: A solution of tetrabutylammonium chloride (181 mg, 0.65 mmol) in dry MeCN (5 mL) was added to a MeCN solution (5 mL) of **TPACage**•$6PF_6$ (50 mg, 0.02 mmol). The resulting dark red precipitate was collected by centrifugation, washed with Me_2CO (3 × 10 mL), and followed by dry under vacuum to give the desired compound **TPACage**•6Cl (34 mg) as a dark red powder (yield: 95%). ^1H NMR (500 MHz, CD_3OD) δ 8.86 (d, J = 6.5 Hz, 12H), 8.04 (d, J = 6.6 Hz, 12H), 7.80 (d, J = 16.2 Hz, 6H), 7.72 (s, 12H), 7.63 (d, J = 8.5 Hz, 12H), 7.23 (d, J = 16.2 Hz, 6H), 7.09 (d, J = 8.5 Hz, 12H), 5.72 (s, 12H). ^{13}C NMR (125 MHz, CD_3OD) δ 156.1, 150.1, 144.6, 142.9, 137.7, 132.3, 131.4, 131.1, 125.7, 125.4, 122.5, 64.2. HRMS-ESI for **TPACage**•6Cl; Calcd for $C_{102}H_{84}Cl_6N_8$: m/z = 509.1964 $[M - 3Cl]^{3+}$; Found: 509.1955 $[M - 3Cl]^{3+}$.

TPACage•$6AsF_6$: A solution of lithium hexafluoroarsenate (180 mg, 0.92 mmol) in H_2O (5 mL) was added to an aqueous solution (5 mL) of **TPACage**•6Cl (50 mg, 0.03 mmol). The resulting dark red precipitate was collected by centrifugation and washed with H_2O (3 × 10 mL) to remove the excess of lithium hexafluoroarsenate. After drying under vacuum, the desired compound **TPACage**•$6AsF_6$ (69 mg) was obtained as a dark red powder (yield: 96%).^1H NMR (500 MHz, CD_3CN) δ 8.55 (d, J = 6.6 Hz, 12H), 7.88 (d, J = 6.6 Hz, 12H), 7.68 (d, J = 16.5 Hz, 6H), 7.61 (s, 12H), 7.58 (d, J = 8.5 Hz, 12H), 7.16 (d, J = 16.3 Hz, 6H), 7.11 (d, J = 8.3 Hz, 12H), 5.58 (s, 12H). ^{13}C NMR (125 MHz, CD_3CN) δ 155.3, 149.5, 144.2, 142.2, 137.0, 131.6, 131.1, 130.8, 125.4, 125.1, 122.3, 64.1. ^{19}F NMR (564 MHz, CD_3CN) δ –63.3, –65.0, –66.6, –68.3. HRMS-ESI for **TPACage**•$6AsF_6$; Calcd for $C_{102}H_{84}F_{36}N_8As_6$: 662.8071 $[M - 3PF_6]^{3+}$; Found: 662.8069 $[M - 3PF_6]^{3+}$.

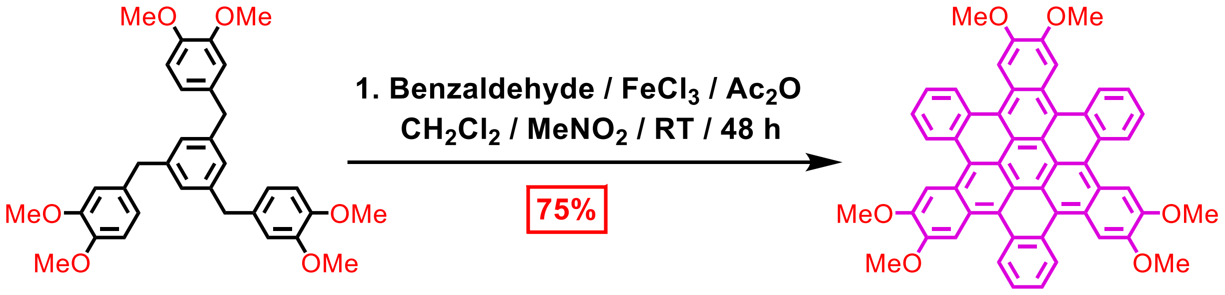

TBB **3H-HBC**

Supplementary Figure 4. Synthesis of **3H-HBC**

3H-HBC: This compound were prepared according to the literature procedure[1] with some modifications. 1 mL $FeCl_3$ (1.18 g, 7.3 mmol) solution in $MeNO_2$ (21 mL) was added dropwise to a solution of benzaldehyde (175 mg, 1.65 mmol) and Ac_2O (0.47 mL, 5 mmol) in CH_2Cl_2 (350 mL) using an injection pump. The resulting solution was followed by the dropwise addition of a solution of 1,3,5-tris(3,4-dimethoxybenzyl)benzene (**TBB**, 0.5 mmol, 264 mg) in CH_2Cl_2 (50 mL) over 1 h. The mixture was stirred at room temperature overnight. The remaining $FeCl_3$ solution in $MeNO_2$ (20 mL) was added dropwise to the reaction mixture over 1 h. After continuing to stir for 12 h under a N_2 atmosphere, cold MeOH (100 mL) was added to quench the reaction and the mixture was poured into cold H_2O (500 mL). The aqueous layer was extracted with CH_2Cl_2 (3 × 100 mL). The combined organic layers were concentrated under vacuum. The residue was purified by column chromatography using CH_2Cl_2 / $MeCO_2Et$ (10:1) as eluent to afford **3H-HBC** as a yellow solid (yield 75%). ^1H NMR (500 MHz, $CDCl_3$) δ 9.32 (dd, *J* = 6.2, 3.4 Hz, 6H), 8.73 (s, 6H), 7.80 (dd, *J* = 6.4, 3.2 Hz, 6H), 4.20 (s, 18H). ^{13}C NMR (125 MHz, $CDCl_3$) δ 148.82, 130.23, 128.42, 126.31, 125.23, 124.96, 120.92, 109.51, 56.31. HRMS-ESI for **3H-HBC**; Calcd for $C_{54}H_{36}O_6$: *m/z* = 780.2512 $[M]^{+\bullet}$; Found: 780.2507 $[M]^{+\bullet}$.

3Me-HBC: This compound was obtained according to a synthetic procedure similar to that employed in the synthesis of **3H-HBC**, except that benzaldehyde (175 mg, 1.65 mmol) was replaced by 4-methylbenzaldehyde (198 mg, 1.65 mmol). **3Me-HBC** was obtained as a yellow powder in 80% yield. ^1H NMR (500 MHz, $CDCl_3$) δ 9.19 (d, *J* = 8.3 Hz, 3H), 9.14 (d, *J* = 4.6 Hz, 3H), 8.71 (d, *J* = 25.2 Hz, 6H), 7.62 (d, *J* = 8.5 Hz, 3H), 4.30 – 4.11 (m, 18H), 2.72 (s, 9H). ^{13}C NMR (125 MHz, $CDCl_3$) δ 148.69, 148.66, 148.64, 148.60, 136.00, 130.37, 128.37, 128.12, 128.10, 127.84, 127.78, 125.23, 125.20, 125.16, 124.99, 124.34, 121.19, 120.64, 109.57, 56.31, 56.28, 56.27, 56.25, 22.26. HRMS-ESI for **3Me-HBC**; Calcd for $C_{57}H_{42}O_6$: *m/z* = 822.2982 $[M]^{+\bullet}$; Found: 822.2974 $[M]^{+\bullet}$.

3F-HBC: This compound was obtained according to a synthetic procedure similar to that employed in the synthesis of **3H-HBC**, except that benzaldehyde (175 mg, 1.65 mmol) was replaced by 4-fluorobenzaldehyde (205 mg, 1.65 mmol). **3F-HBC** was obtained as a brown powder in 82% yield. ^{1}H NMR (500 MHz, $CDCl_3$) δ 9.29 (dd, J = 9.1, 5.9 Hz, 3H), 8.99 (dd, J = 11.3, 2.7 Hz, 3H), 8.65 (d, J = 29.2 Hz, 6H), 7.56 (ddd, J = 9.1, 7.6, 2.6 Hz, 3H), 4.21 (d, J = 13.1 Hz, 18H). ^{13}C NMR (125 MHz, $CDCl_3$) δ 162.21, 160.26, 149.24, 149.10, 131.78, 131.71, 131.13, 131.06, 126.87, 125.44, 125.20, 124.99, 123.96, 121.45, 120.31, 115.18, 115.00, 113.05, 112.86, 109.47, 108.73, 56.37, 56.33. HRMS-ESI for **3F-HBC**; Calcd for $C_{54}H_{33}F_3O_6$: m/z = 834.2229 $[M]^{+\bullet}$; Found: 834.2228 $[M]^{+\bullet}$.

Supplementary Note 2. NMR Spectroscopy

(1) ^{1}H NMR and ^{13}C NMR spectroscopy performed on TPAP, TPAB•3PF_6, TPACage•6PF_6, TPACage•6Cl, TPACage•6AsF_6, and the guest molecules 3H-HBC, 3Me-HBC, 3F-HBC

Chemical shifts are reported in ppm relative to the signals corresponding to the residual non-deuterated solvents (CD_3CN: δ_H = 1.96 ppm and δ_C = 1.32 and 118.26 ppm; CD_3OD: δ_H = 3.31 ppm and δ_C = 49.00 ppm; $CDCl_3$: δ_H = 7.26 ppm and δ_C = 77.16 ppm).

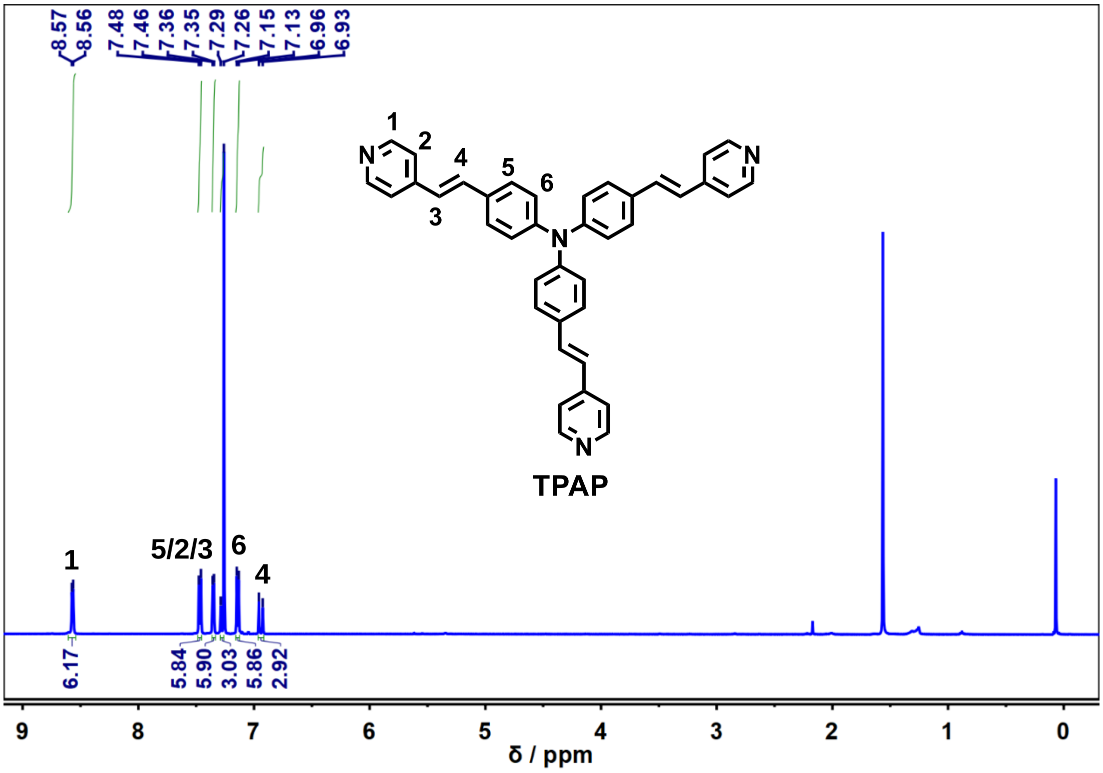

Supplementary Figure 5. ^{1}H NMR spectrum (500 MHz, $CDCl_3$, 298 K) of **TPAP**

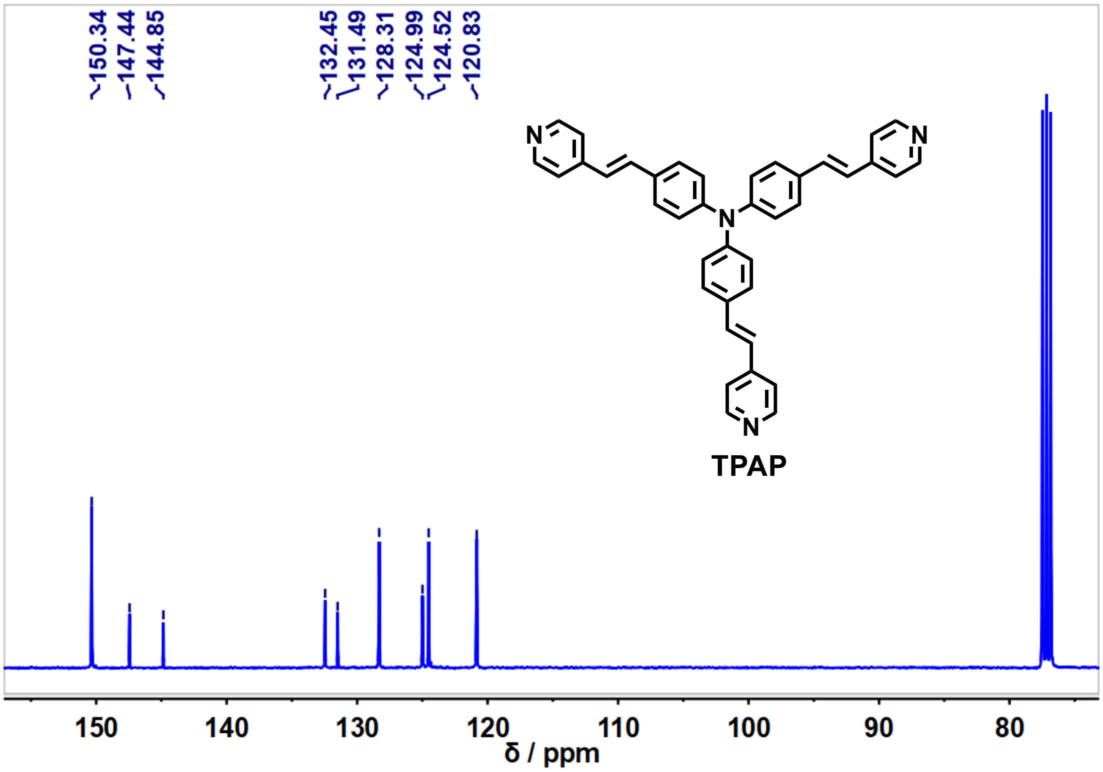

Supplementary Figure 6. ^{13}C NMR spectrum (100 MHz, $CDCl_3$, 298 K) of **TPAP**

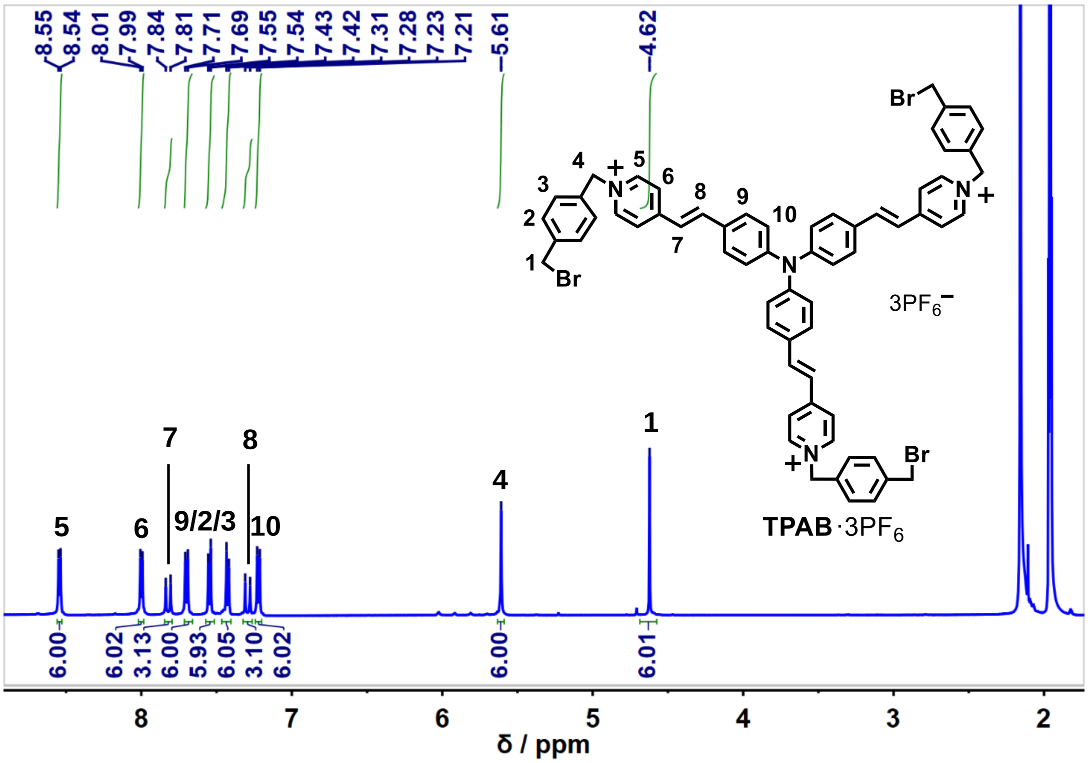

Supplementary Figure 7. ^{1}H NMR spectrum (500 MHz, CD_3CN, 298 K) of **TPAB•**$3PF_6$

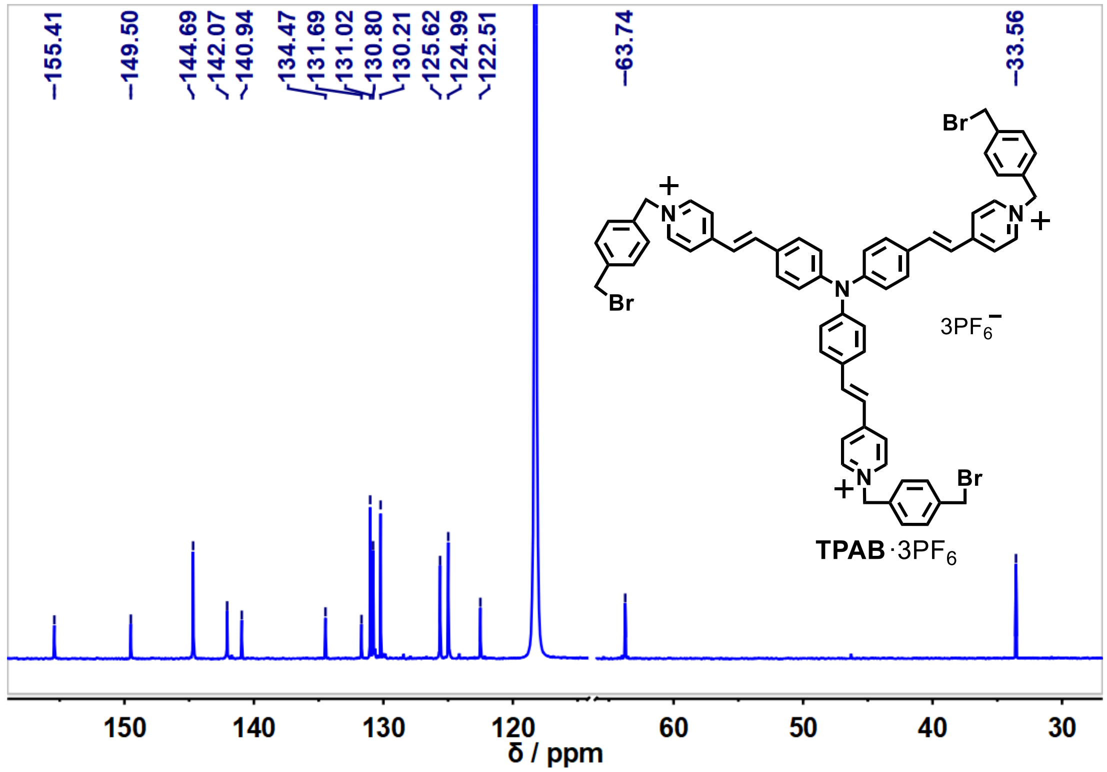

Supplementary Figure 8. ^{13}C NMR spectrum (100 MHz, CD_3CN, 298 K) of **TPAB•**$3PF_6$

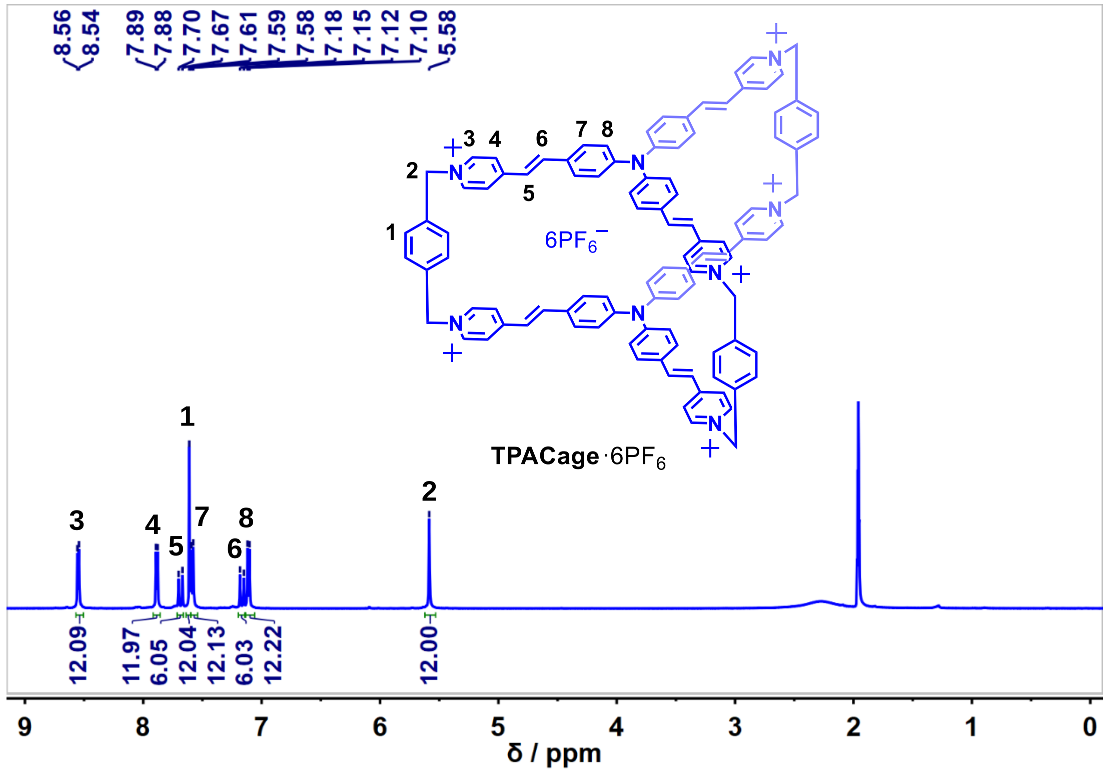

Supplementary Figure 9. ^{1}H NMR spectrum (500 MHz, CD_3CN, 298 K) of **TPACage•**$6PF_6$

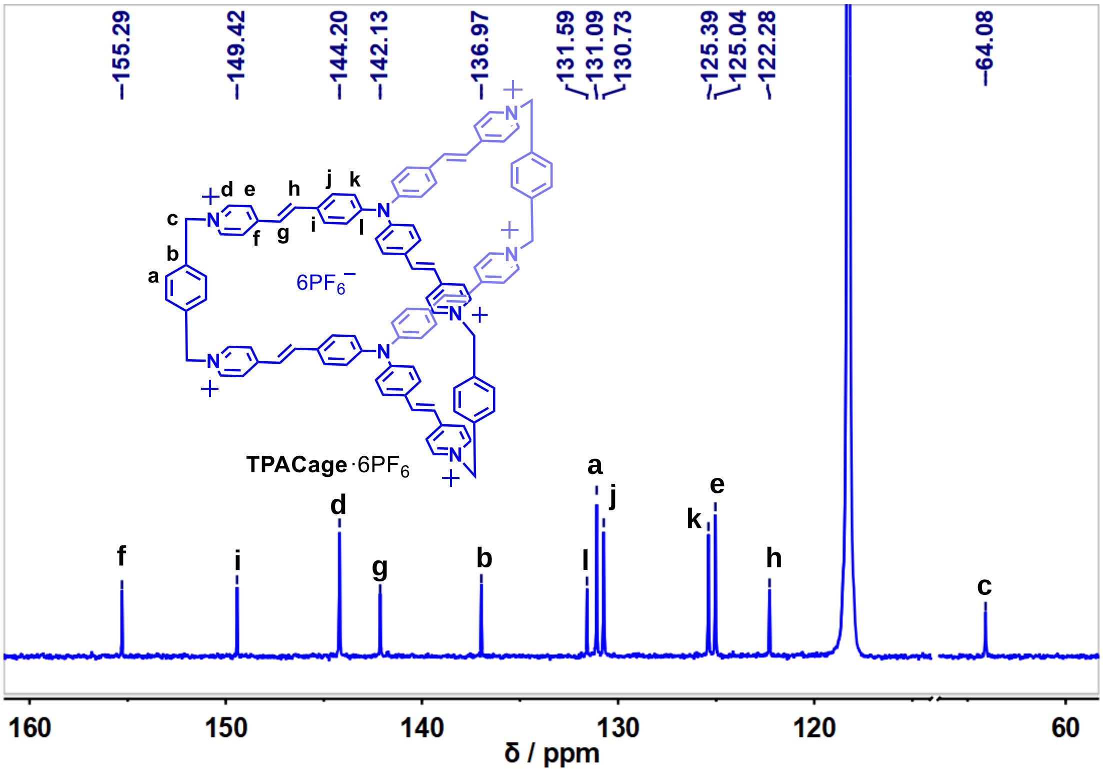

Supplementary Figure 10. ^{13}C NMR spectrum (100 MHz, CD_3CN, 298 K) of **TPACage•**$6PF_6$

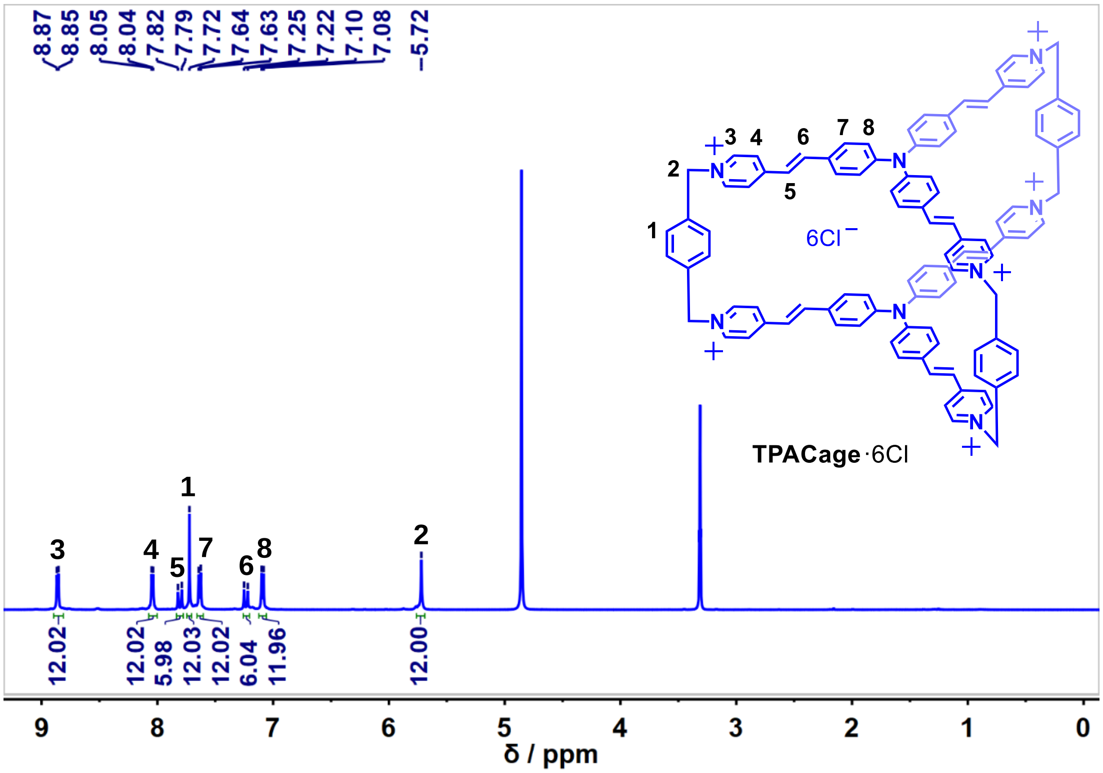

Supplementary Figure 11. ^{1}H NMR spectrum (500 MHz, CD_3OD, 298 K) of **TPACage•**6Cl

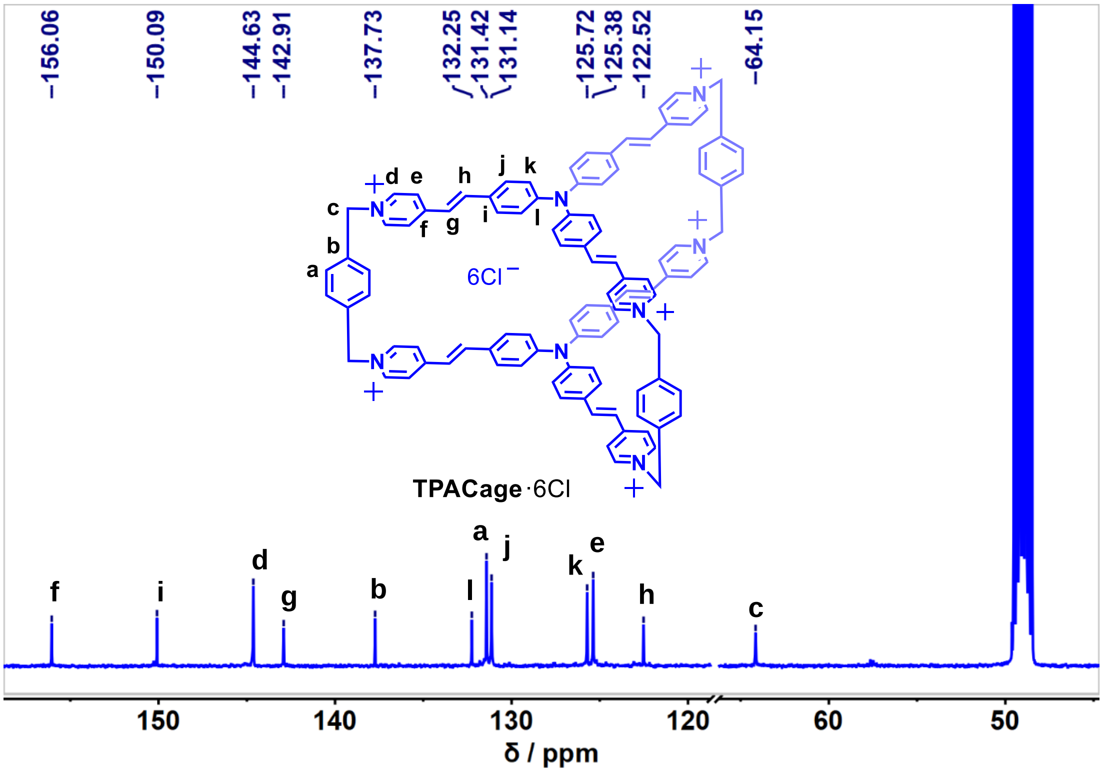

Supplementary Figure 12. ^{13}C NMR spectrum (125 MHz, CD_3OD, 298 K) of **TPACage•**6Cl

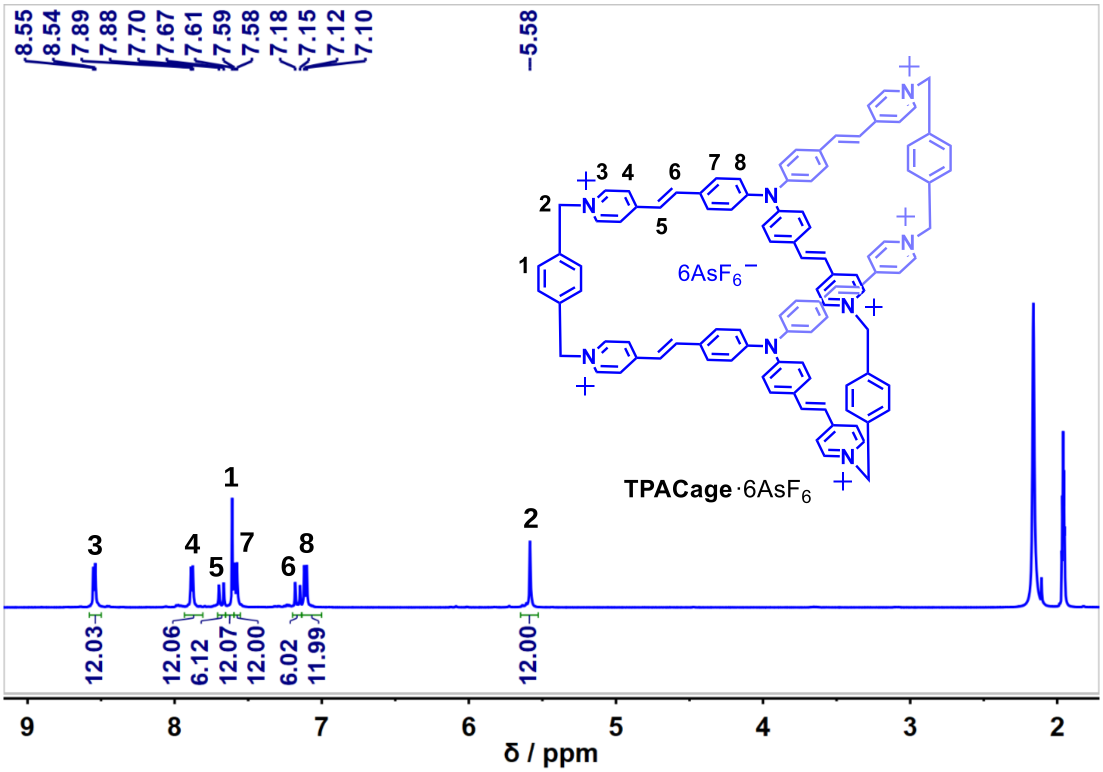

Supplementary Figure 13. ^{1}H NMR spectrum (500 MHz, CD_3CN, 298 K) of **TPACage•**$6AsF_6$

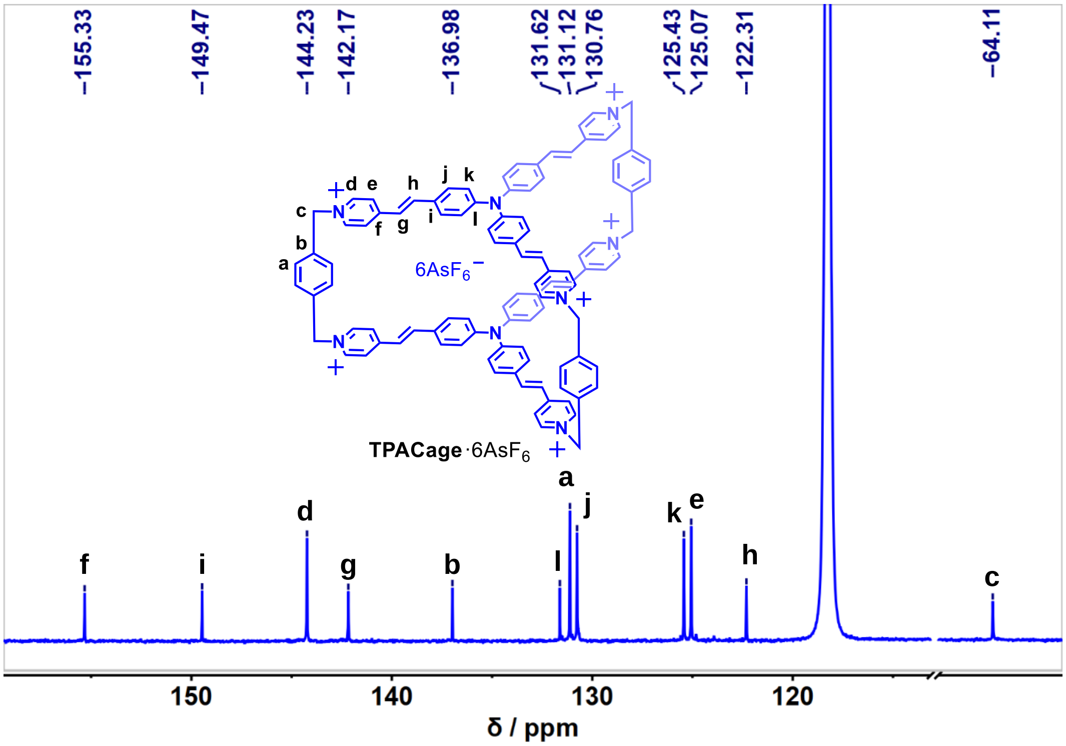

Supplementary Figure 14. ^{13}C NMR spectrum (125 MHz, CD_3CN, 298 K) of **TPACage•**$6AsF_6$

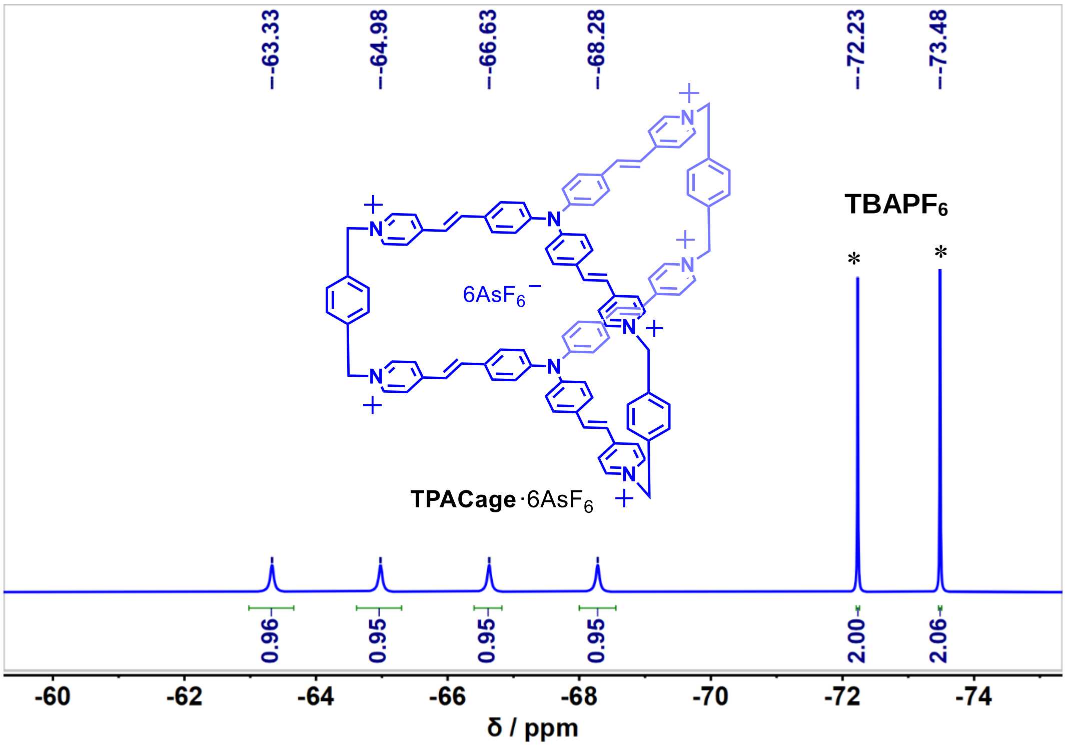

Supplementary Figure 15. ^{19}F NMR spectrum (564 MHz, CD_3CN, 298 K) of **TPACage•**$6AsF_6$. In order to confirm that all of the counteranions have been exchanged for $[AsF_6]^-$, 6 equiv of tetrabutylammonium hexafluorophosphate ($TBAPF_6$), which serves as an internal standard, were added to the CD_3CN solution of **TPACage•**$6AsF_6$. ^{19}F signals for $[PF_6]^-$ anions are indicated by an asterisk (∗).

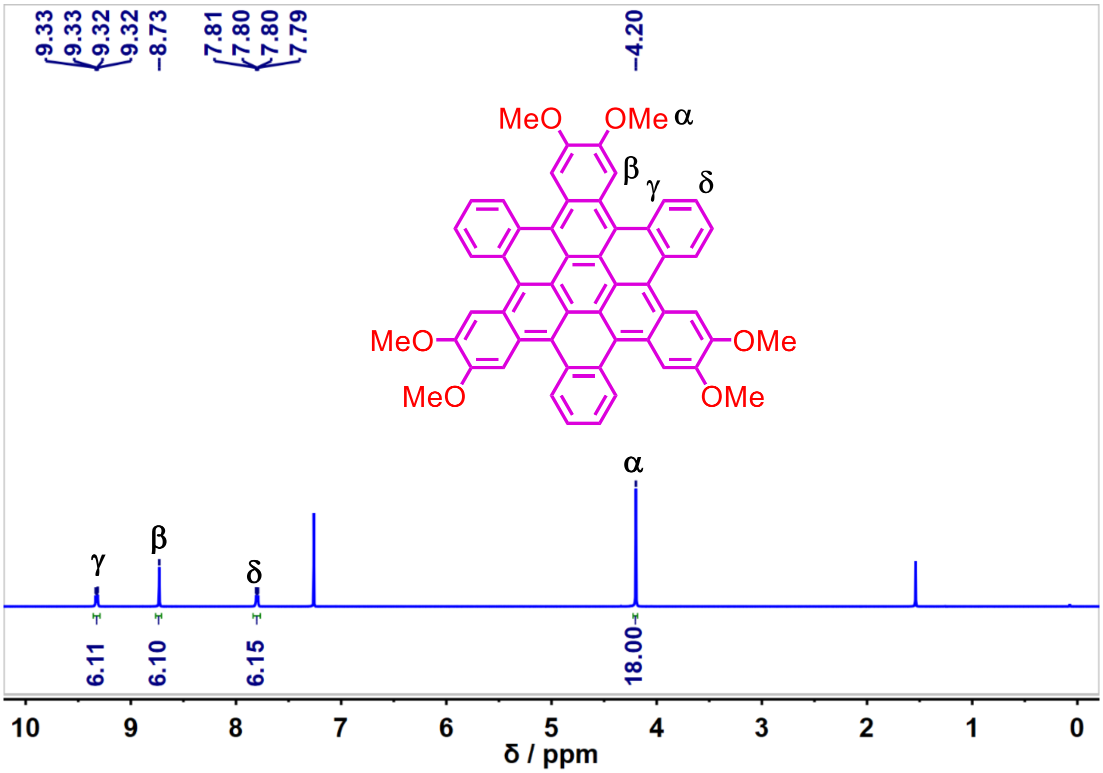

Supplementary Figure 16. ^{1}H NMR spectrum (500 MHz, $CDCl_3$, 298 K) of **3H-HBC**

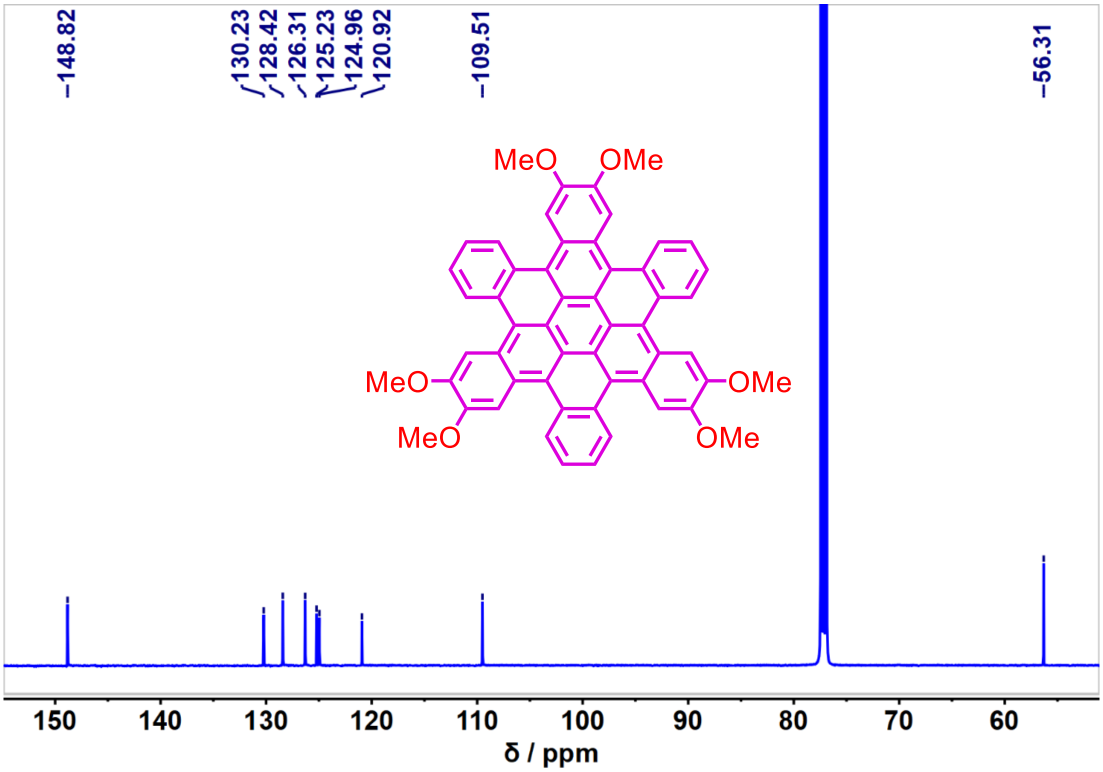

Supplementary Figure 17. ^{13}C NMR spectrum (125 MHz, $CDCl_3$, 298 K) of **3H-HBC**

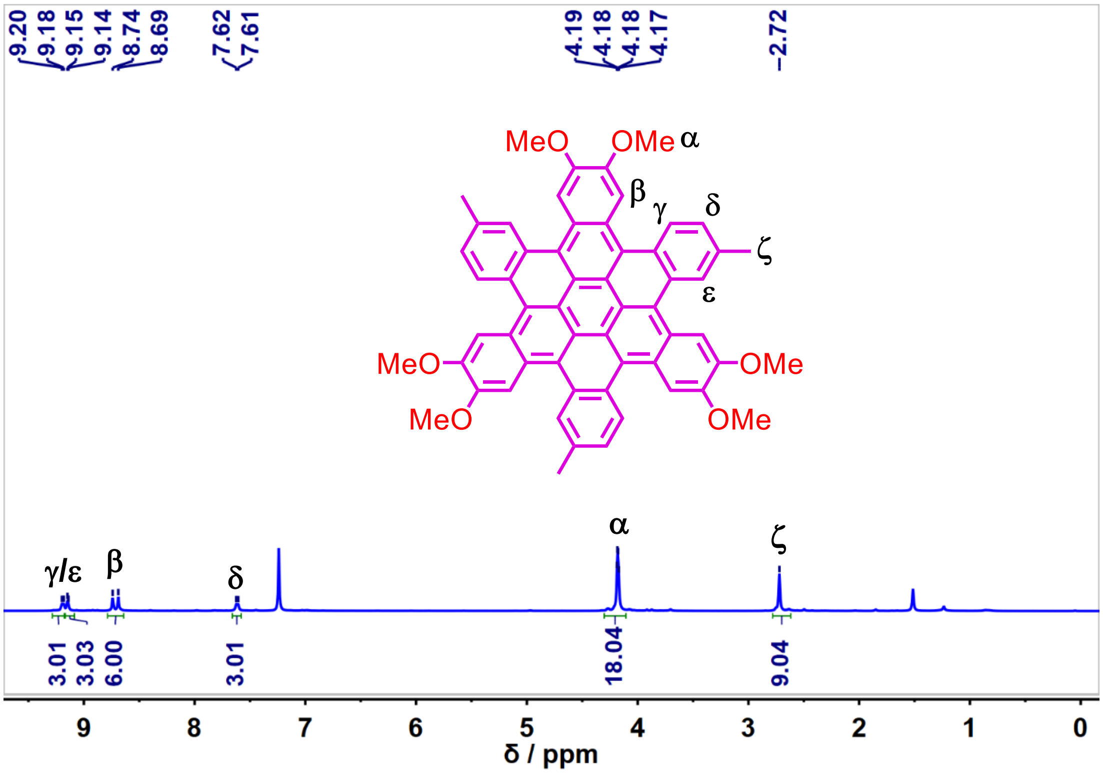

Supplementary Figure 18. ^{1}H NMR spectrum (500 MHz, $CDCl_3$, 298 K) of **3Me-HBC**

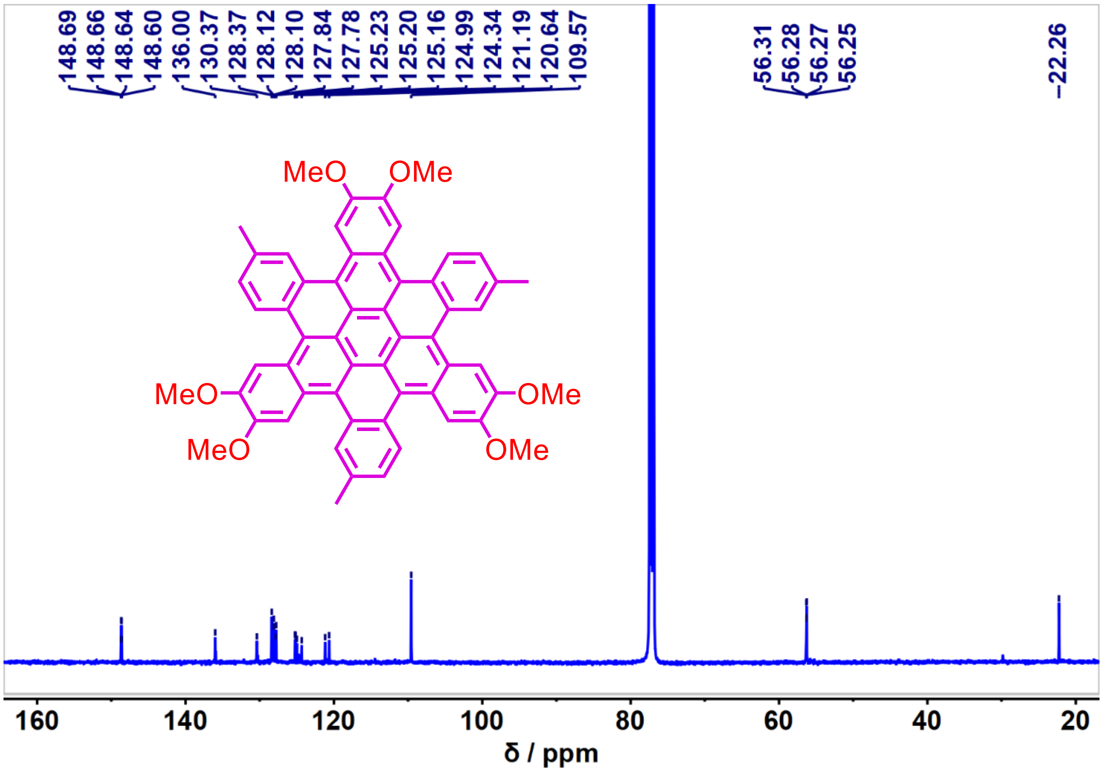

Supplementary Figure 19. ^{13}C NMR spectrum (125 MHz, $CDCl_3$, 298 K) of **3Me-HBC**

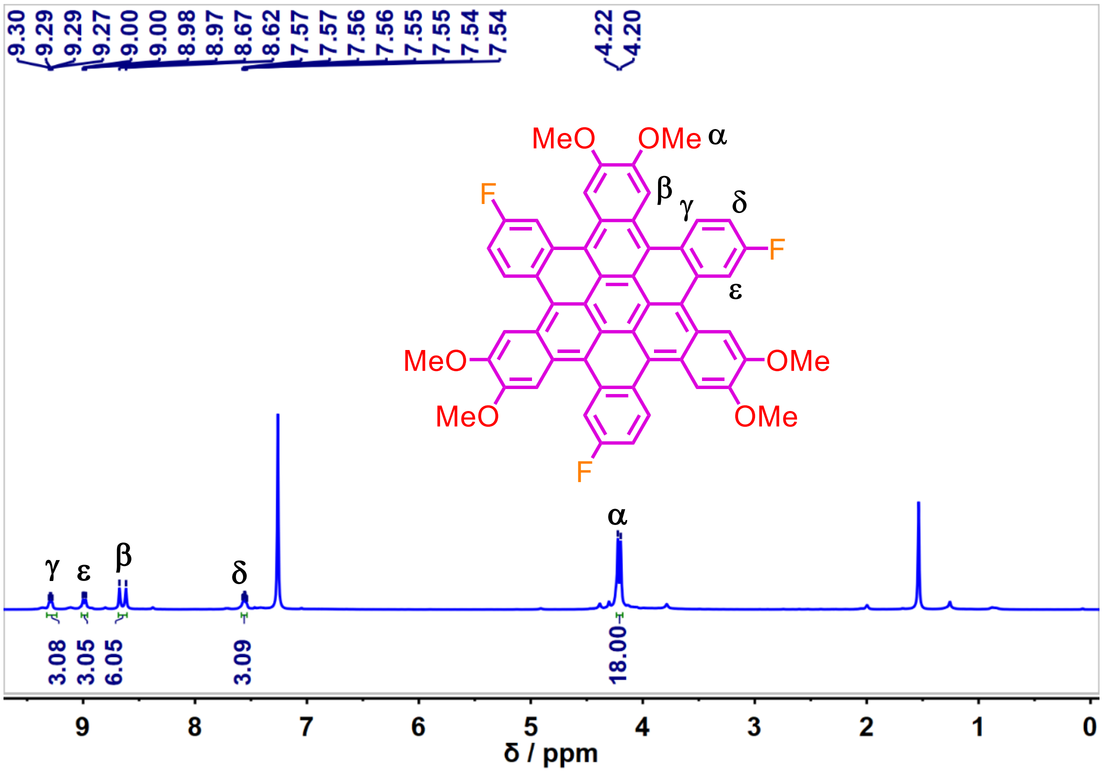

Supplementary Figure 20. ^{1}H NMR spectrum (500 MHz, $CDCl_3$, 298 K) of **3F-HBC**

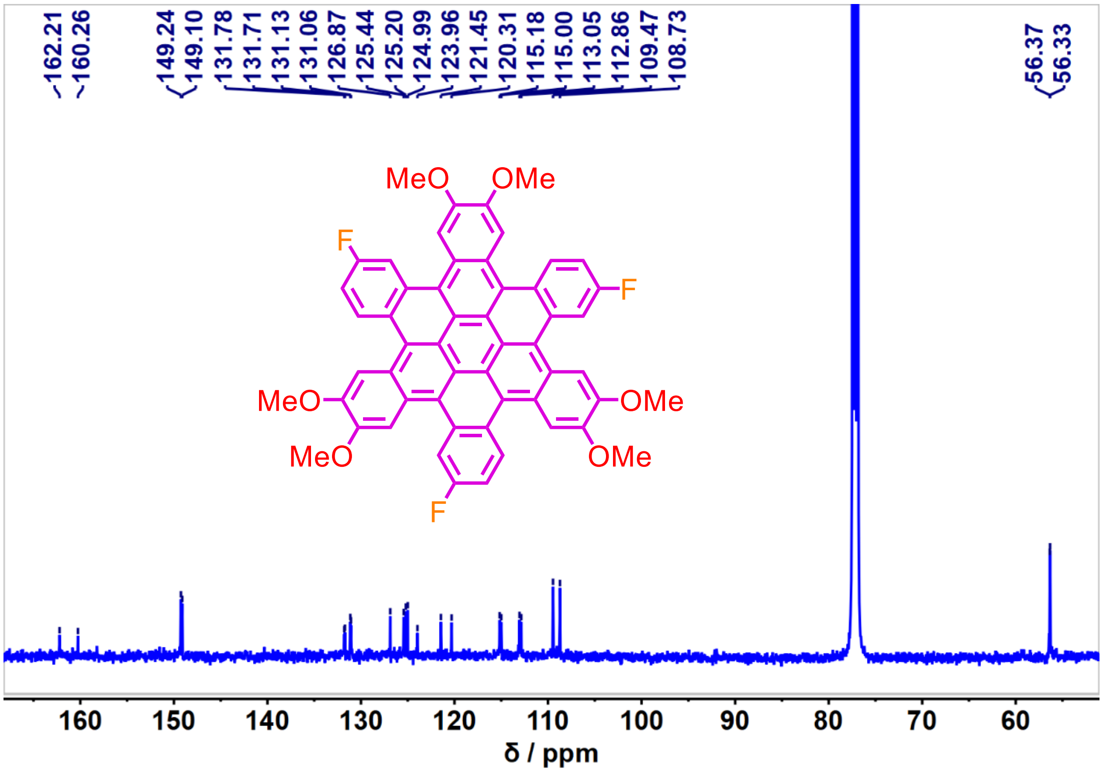

Supplementary Figure 21. ^{13}C NMR spectrum (125 MHz, $CDCl_3$, 298 K) of **3F-HBC**

(2) Two-dimensional ^{1}H–^{1}H COSY, ROESY, and ^{1}H–^{13}C HSQC, HMBC, as well as DOSY NMR spectra for TPACage•6PF$_6$

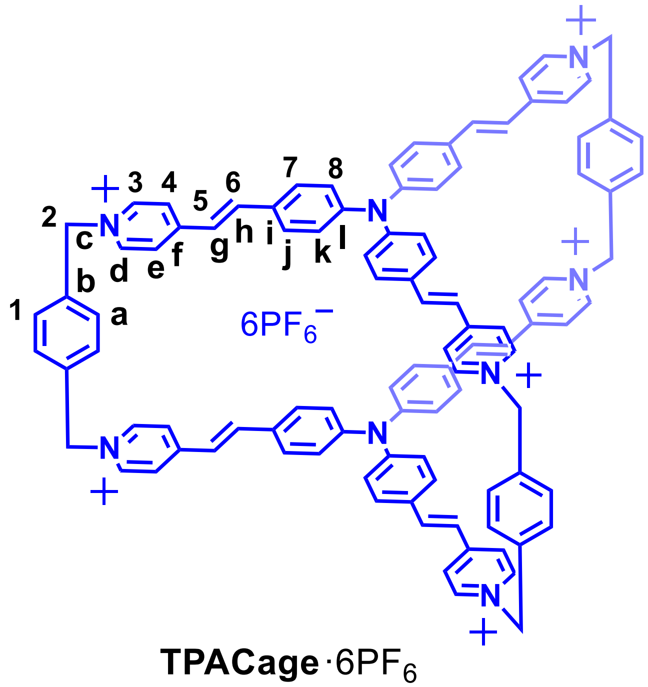

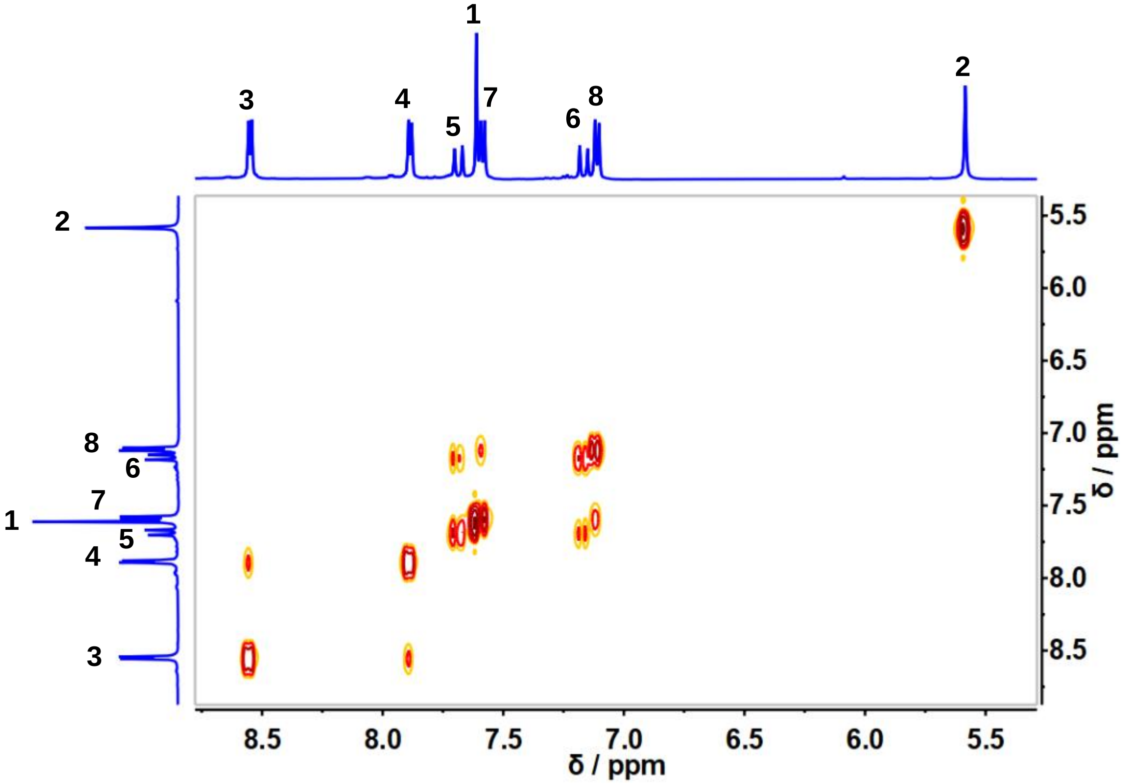

Supplementary Figure 22. 2D ^{1}H–^{1}H COSY NMR Spectrum (500 MHz, CD_3CN, 298 K) of **TPACage•**6PF$_6$

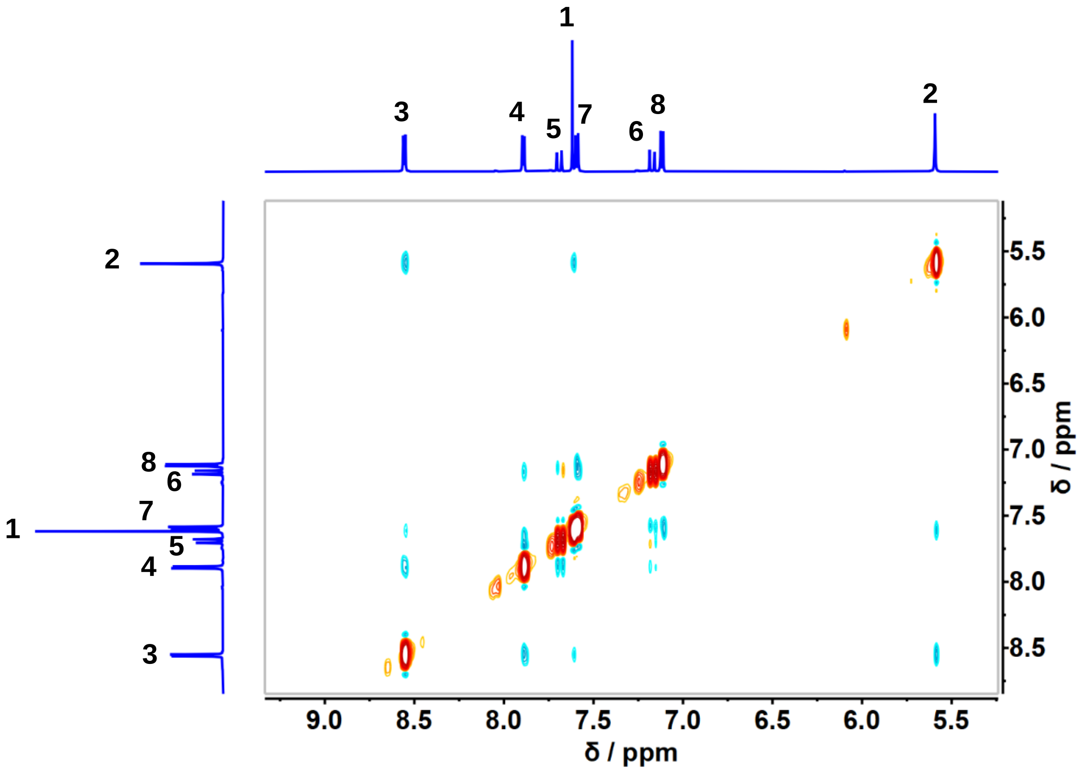

Supplementary Figure 23. 2D ^{1}H–^{1}H ROESY NMR Spectrum (500 MHz, CD_3CN, 298 K) of **TPACage•**$6PF_6$

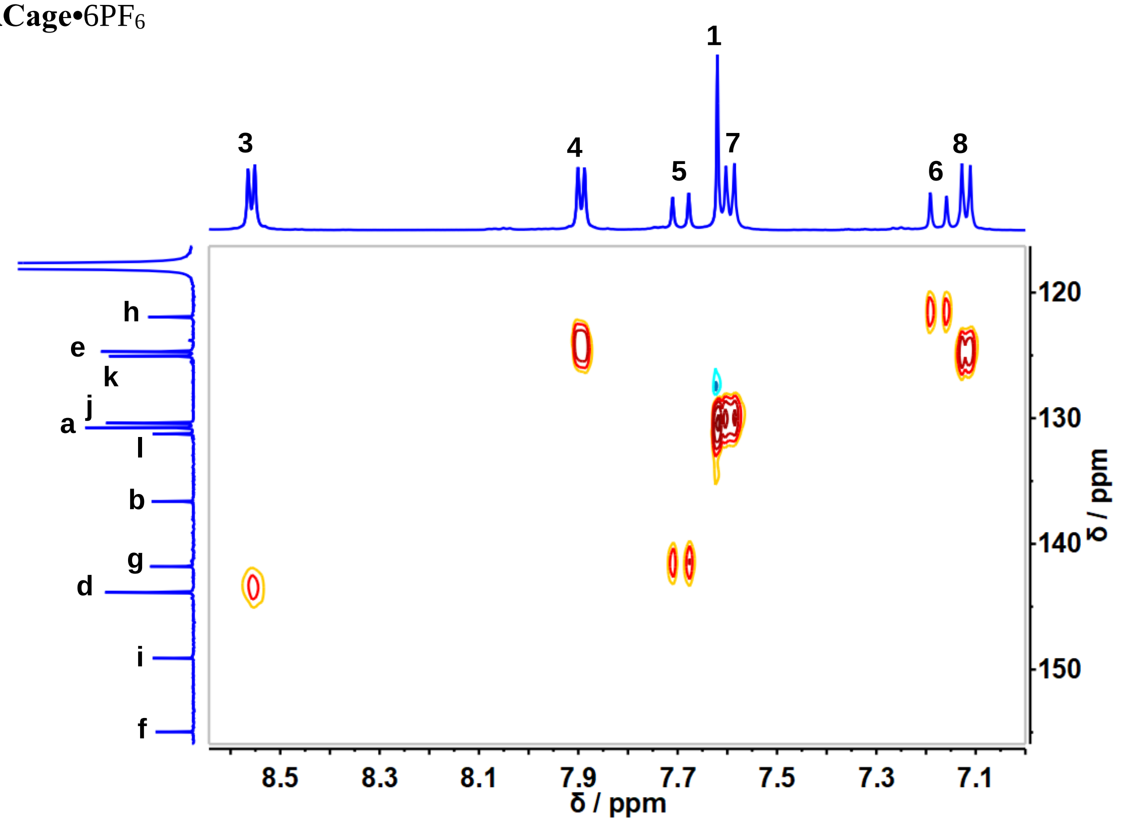

Supplementary Figure 24. Part of the ^{1}H–^{13}C Heteronuclear Single Quantum Coherence (HSQC) spectrum (125 MHz, CD_3CN, 298 K) of **TPACage•**$6PF_6$

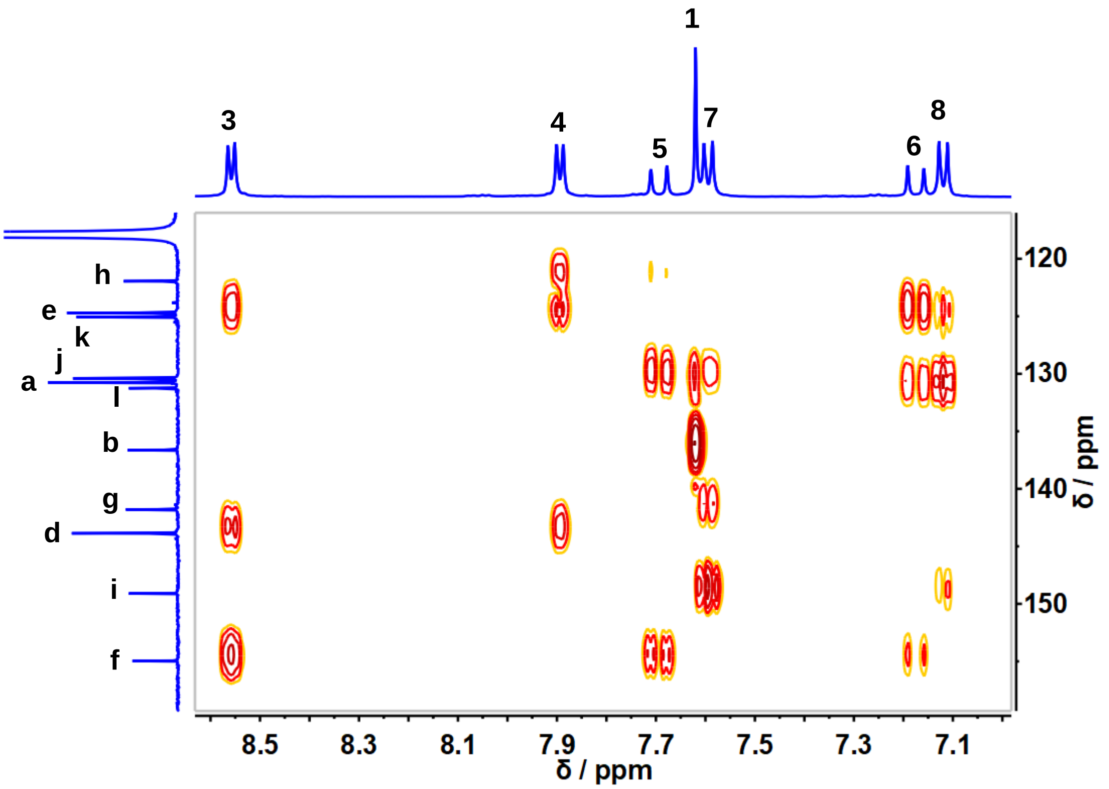

Supplementary Figure 25. Part of the ^{1}H–^{13}C Heteronuclear Multiple Bond Coherence (HMBC) spectrum (125 MHz, CD_3CN, 298 K) of **TPACage**•$6PF_6$

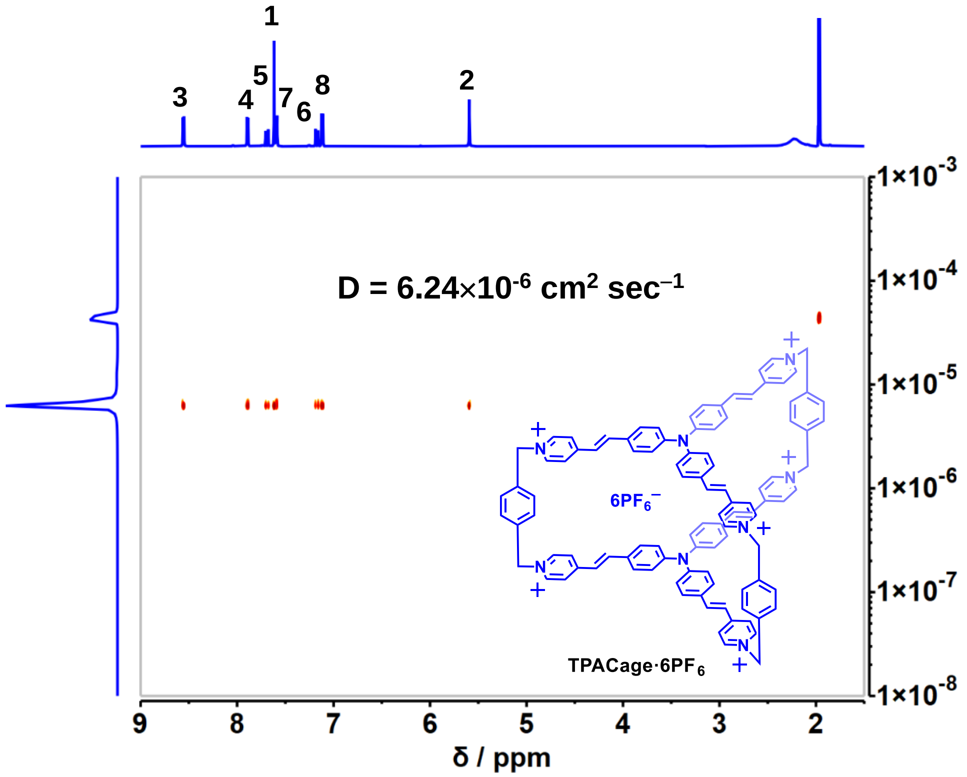

Supplementary Figure 26. 2D DOSY spectrum (600 MHz, CD_3CN, 298 K) of **TPACage**•$6PF_6$

(3) One-dimensional ^{1}H NMR spectra and two-dimensional ^{1}H–^{1}H COSY, ROESY NMR spectra of the host-guest complexes: COR⊂TPACage•6PF$_6$, 3H-HBC⊂TPACage•6PF$_6$, 3Me-HBC⊂TPACage•6PF$_6$, 3F-HBC⊂TPACage•6PF$_6$

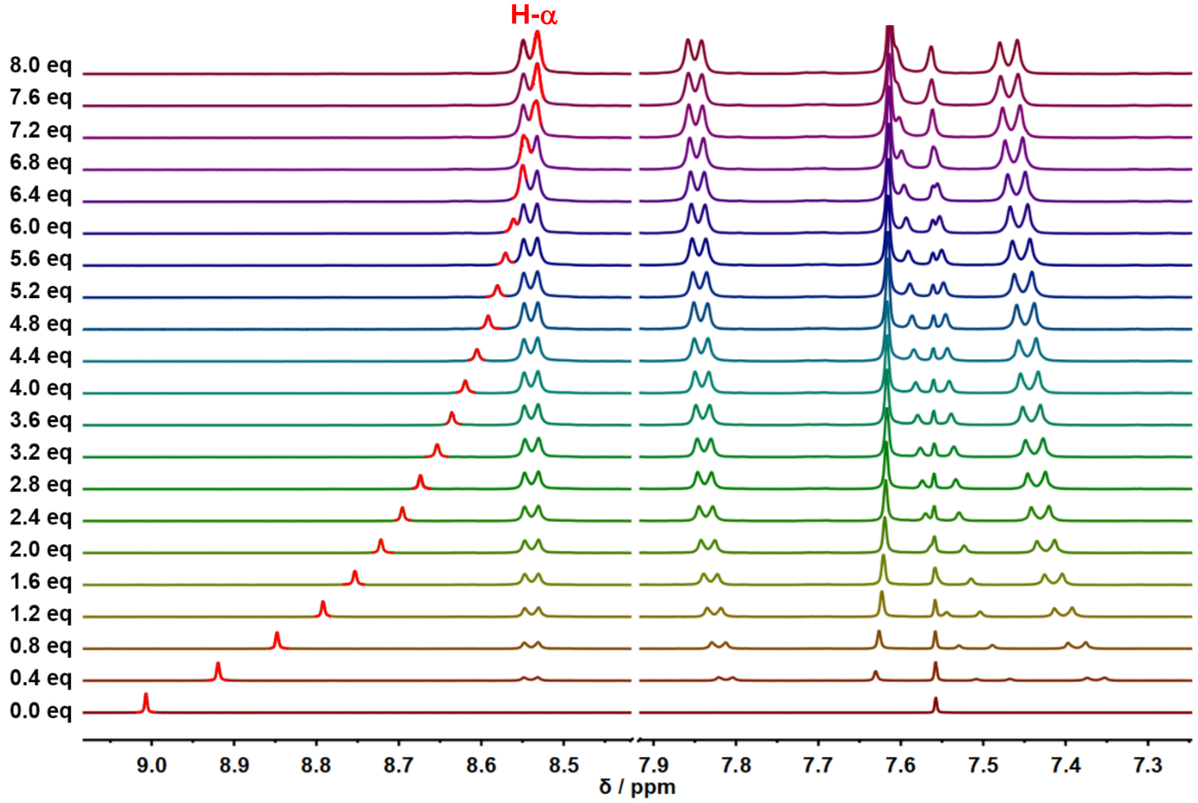

Supplementary Figure 27. ^{1}H NMR Titration (400 MHz, 298 K) of **COR** upon addition of different equivalents of **TPACage^{6+}** in the solution of CD_3CN / $CDCl_3$ (4:1), ([**COR**] = 5 × 10^{-4} M, [**TPACage^{6+}**] / [**COR**] = 0 – 8 equiv).

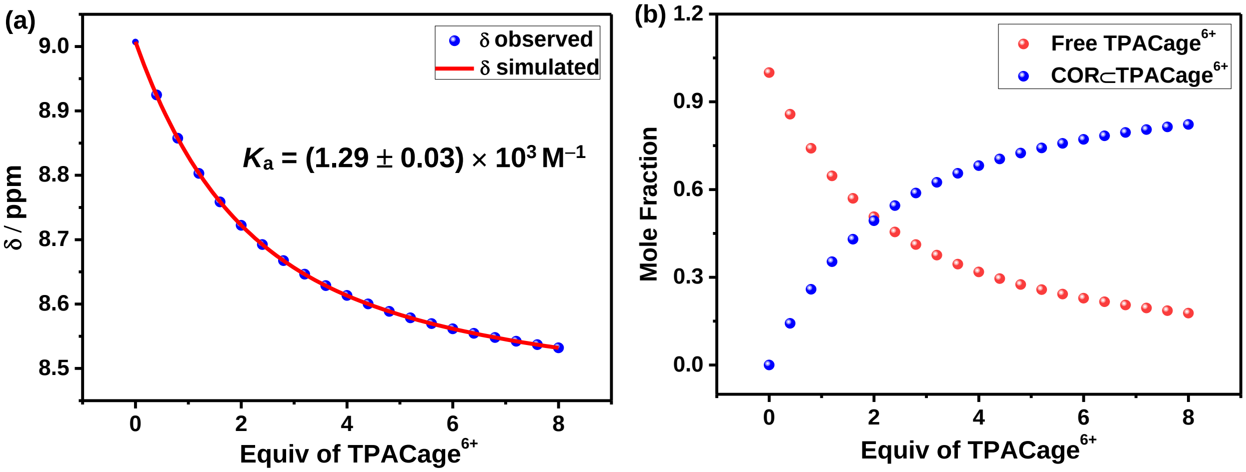

Supplementary Figure 28. (a) Observed and calculated binding curves for ^{1}H NMR spectra signal of H−α in **COR**; (b) Mole fractions are based on the fitting results, indicating that the concentration of the free **COR** undergoes a continuous decrease (red trace), while the concentration of **COR⊂TPACage**•6PF$_6$ complex undergoes a continuous increase (blue trace).

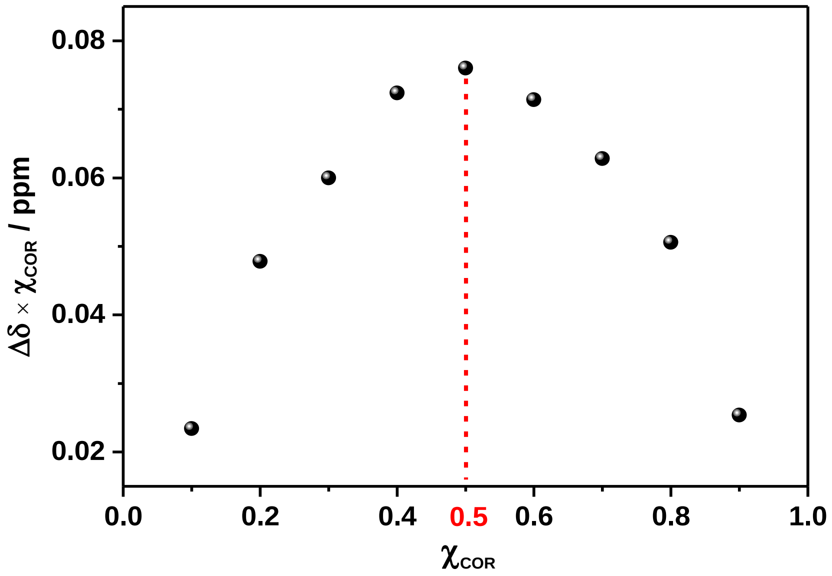

Supplementary Figure 29. Job-plot analysis of the binding ratio between **TPACage**•6PF_6 and **COR** according to the chemical shifts of H-α in **COR**. The total concentration of host and guest is constant ([**TPACage**•6PF_6] + [**COR**] = 1.0 × 10^{-3} M).

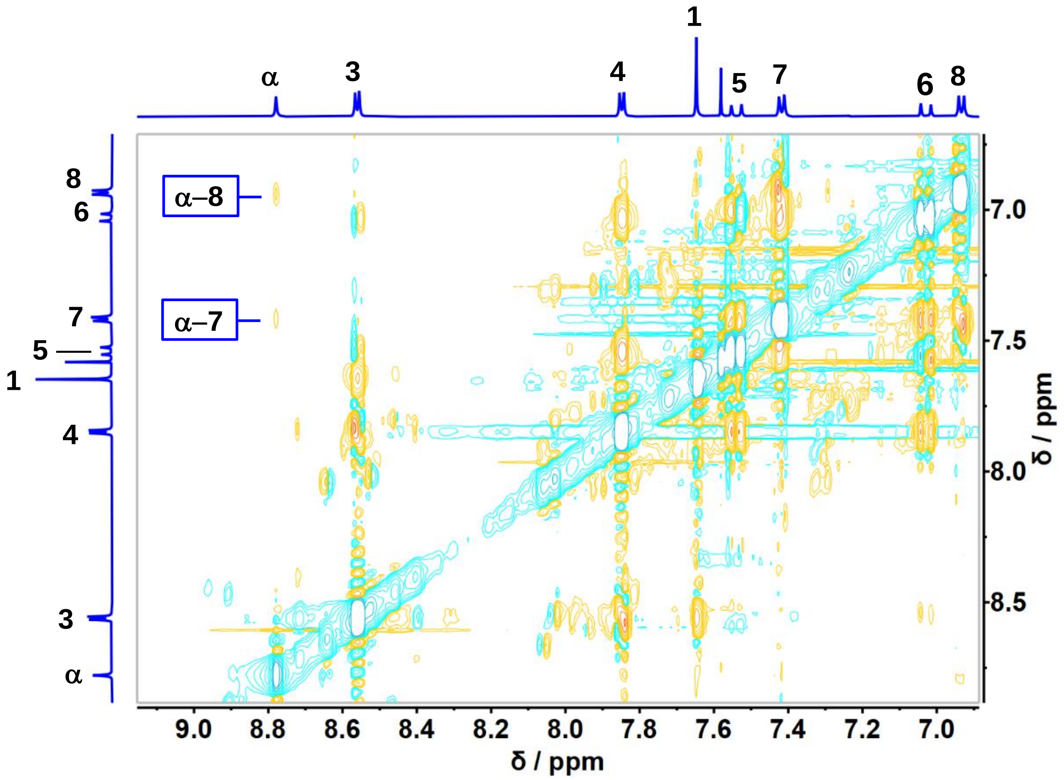

Supplementary Figure 30. Annotated 2D ^1H–^1H ROESY NMR spectrum (500 MHz, CD_3CN:$CDCl_3$ = 4:1, 298 K) of **COR⊂TPACage**•6PF_6

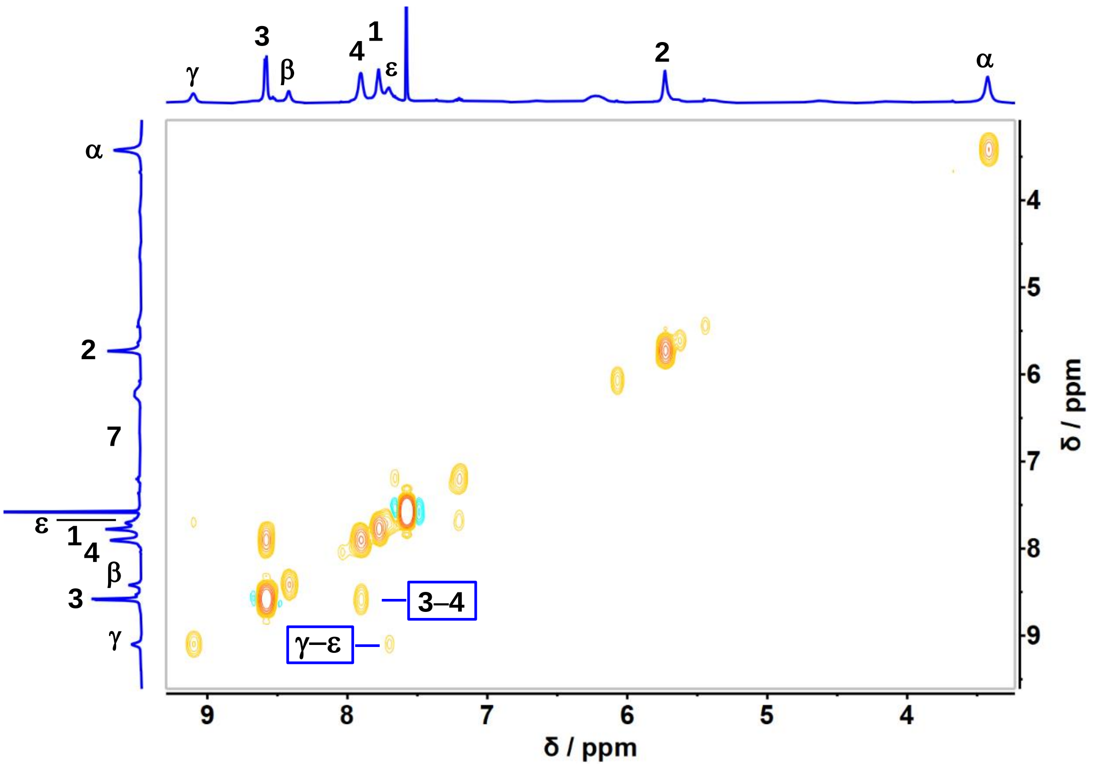

Supplementary Figure 31. Annotated 2D ^{1}H–^{1}H COSY NMR spectrum (500 MHz, CD_3CN:$CDCl_3$ = 4:1, 298 K) of **3H-HBC⊂TPACage**•$6PF_6$

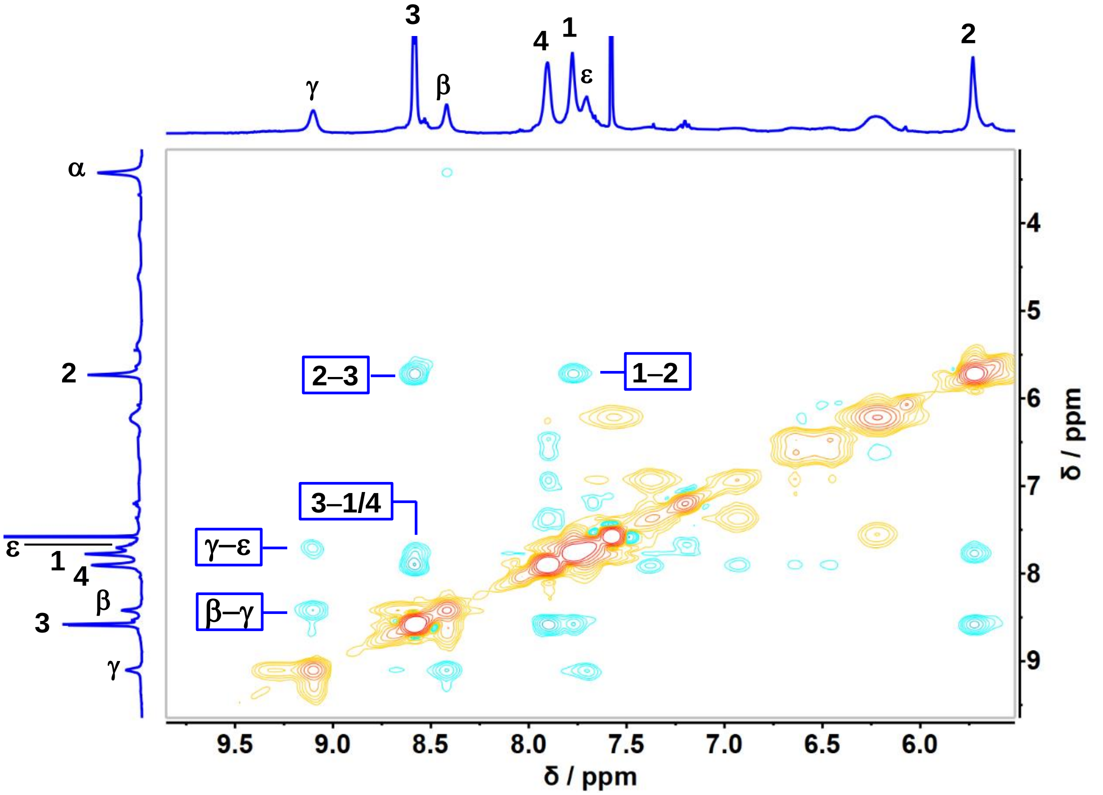

Supplementary Figure 32. Annotated 2D ^{1}H–^{1}H ROESY NMR spectrum (500 MHz, CD_3CN:$CDCl_3$ = 4:1, 298 K) of **3H-HBC⊂TPACage**•$6PF_6$

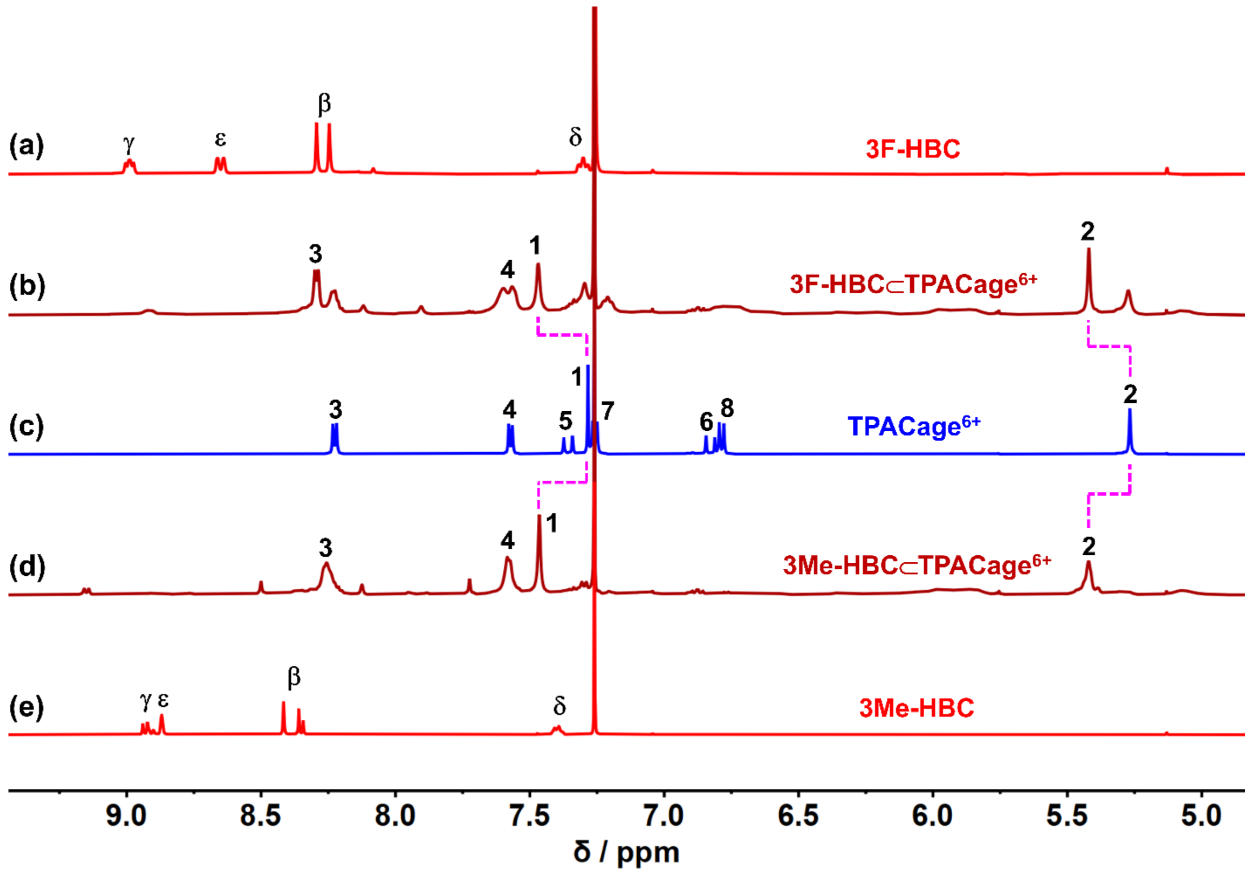

Supplementary Figure 33. ^{1}H NMR Spectra (500 MHz, $CD_3CN : CDCl_3$ = 4:1, [**TPACage**•$6PF_6$] = [**3F-HBC**] = [**3Me-HBC**] = 5.0×10^{-4} M, 298 K) of (a) **3F-HBC**, (b) **3F-HBC⊂TPACage**•$6PF_6$, (c) **TPACage**•$6PF_6$, (d) **3Me-HBC⊂TPACage**•$6PF_6$, (e) **3Me-HBC**, showing how the signal changes for protons upon forming the host-guest complexes. The chemical shifts for protons in **TPACage**$^{6+}$ host, as well as those for the **3Me-HBC** and **3F-HBC** guests all show marked changes and the resonances undergo severe broadening. The protons H-1 and H-2 attached to the *p*-xylylene linkers in **TPACage**•$6PF_6$ show characteristic downfield shifts, conforming that the **3Me-HBC** and **3F-HBC** are encapsulated by **TPACage**$^{6+}$.

Supplementary Note 3. Mass Spectrometry

(1) High-resolution mass spectrometry (HRMS) of TPAP, TPAB•$3PF_6$, TPACage•$6PF_6$, TPACage•6Cl, TPACage•$6AsF_6$ and the guest molecules 3H-HBC, 3Me-HBC, 3F-HBC

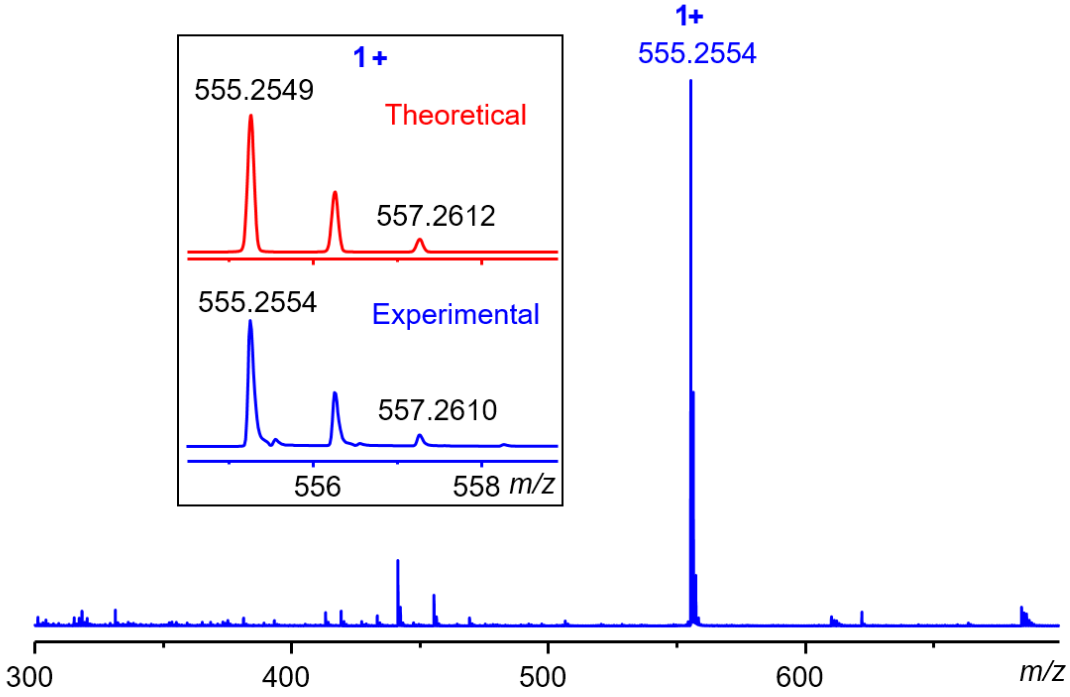

Supplementary Figure 34. HRMS of **TPAP**. Inset shows the theoretical (red) and experimental (blue) isotope patterns for the one positive charge state of **TPAP**.

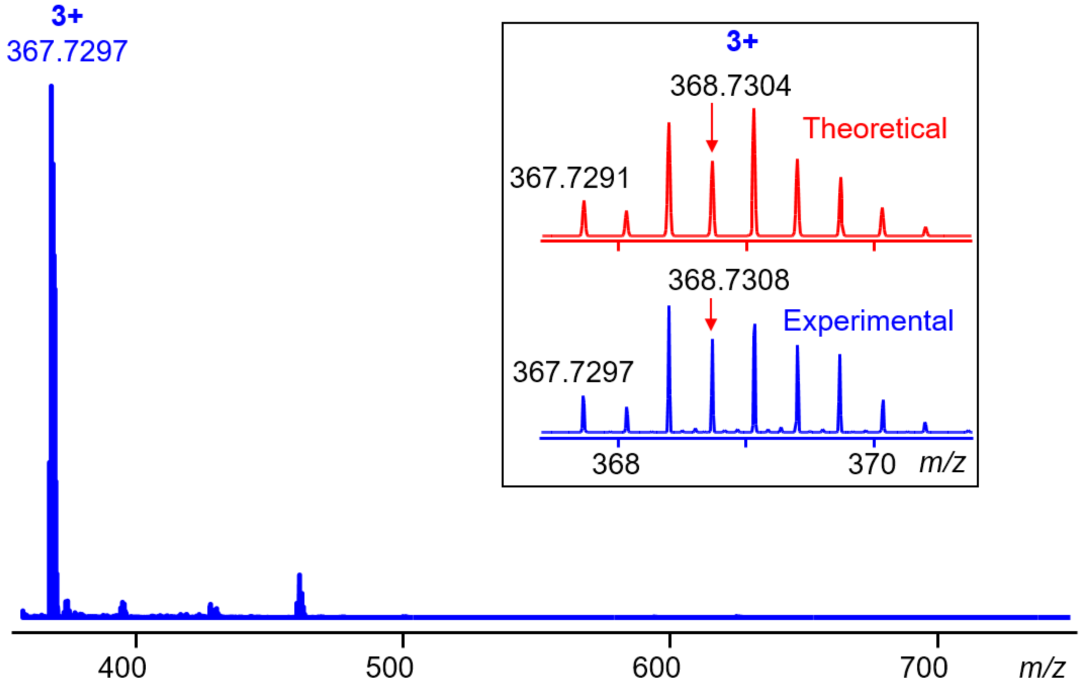

Supplementary Figure 35. HRMS of **TPAB**•$3PF_6$. Inset shows the theoretical (red) and experimental (blue) isotope patterns for the three positive charge state of **TPAB**•$3PF_6$.

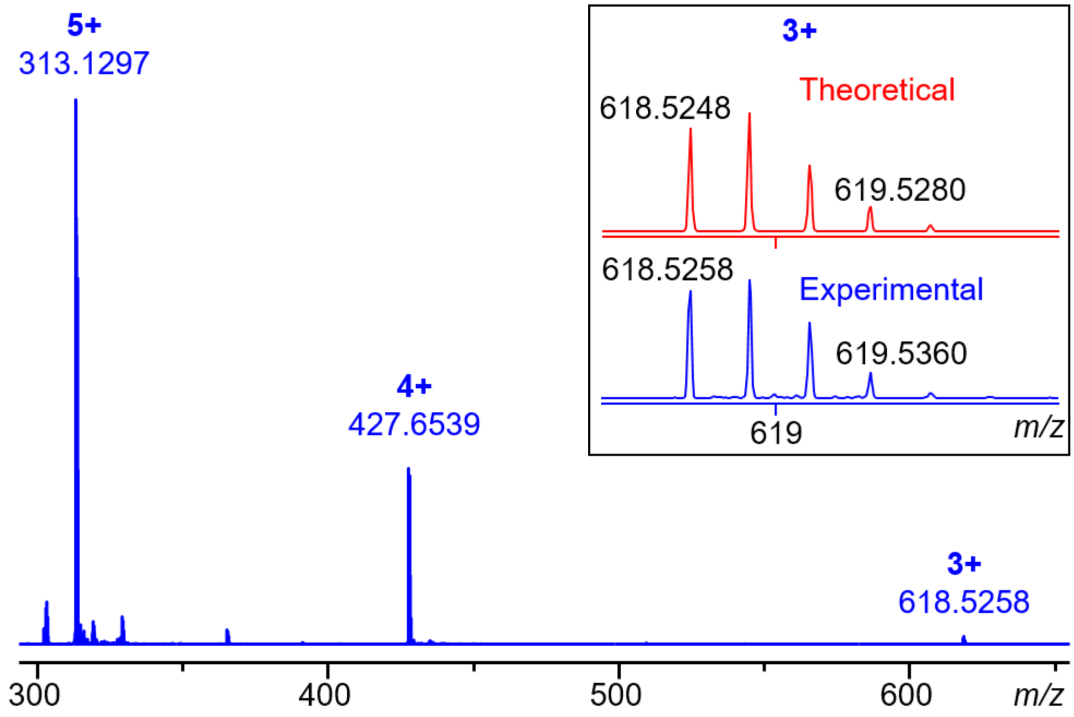

Supplementary Figure 36. HRMS of **TPACage**•6PF_6. Inset shows the theoretical (red) and experimental (blue) isotope patterns for the three positive charge state of **TPACage**•6PF_6.

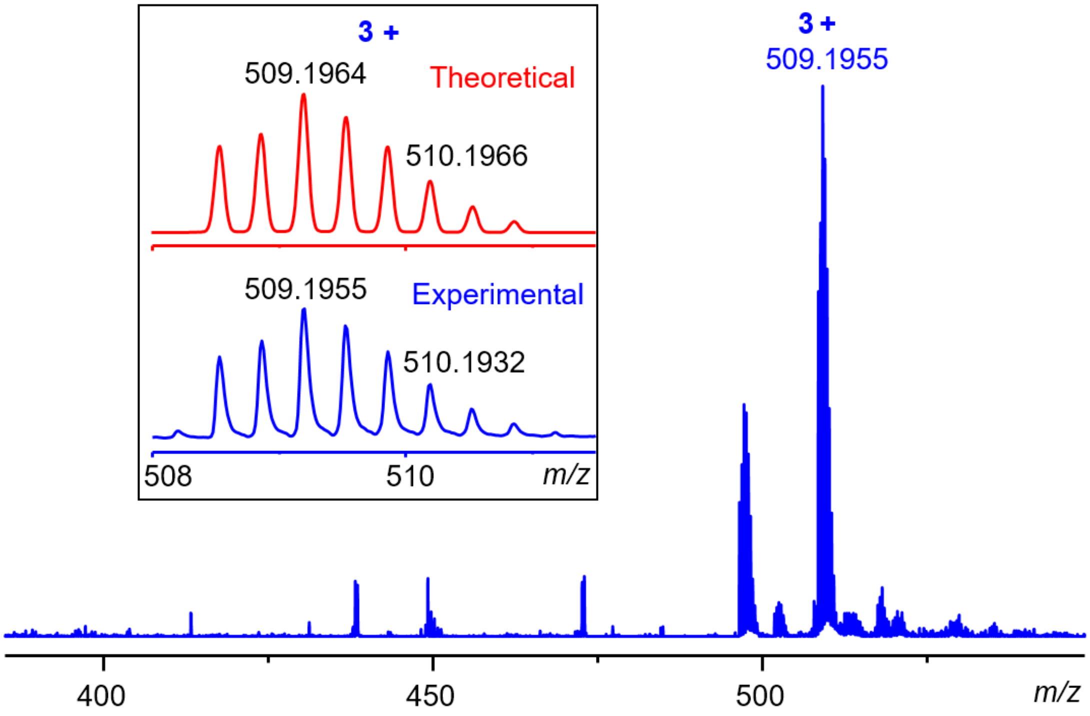

Supplementary Figure 37. HRMS of **TPACage**•6Cl. Inset shows the theoretical (red) and experimental (blue) isotope patterns for the three positive charge state of **TPACage**•6Cl.

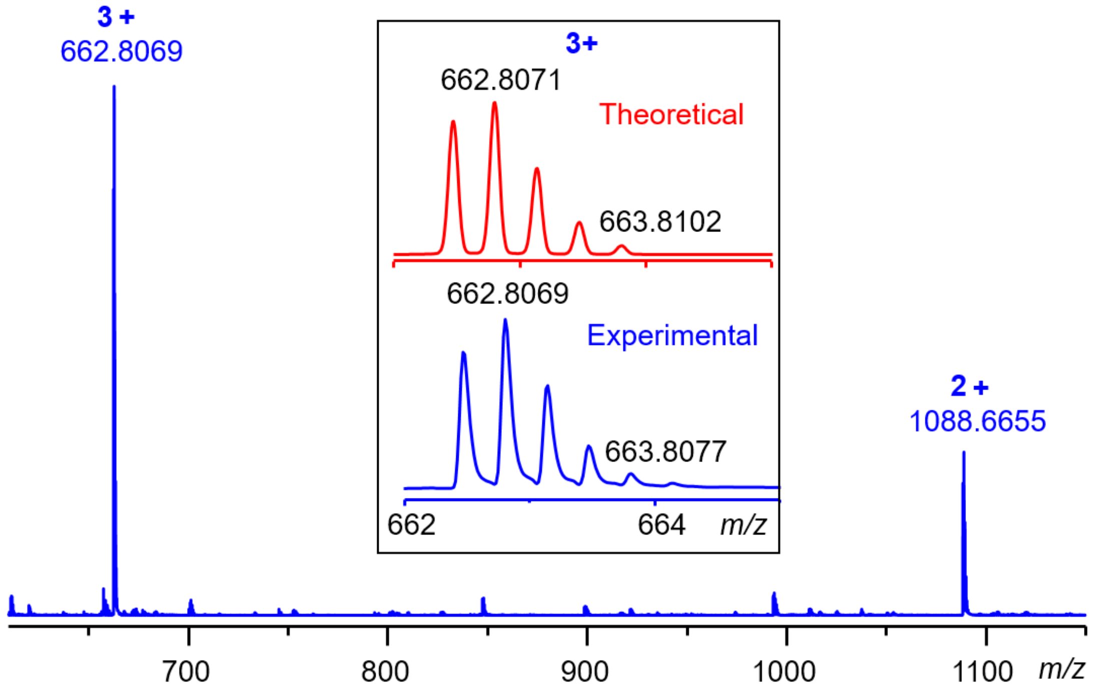

Supplementary Figure 38. HRMS of **TPACage•**6AsF$_6$. Inset shows the theoretical (red) and experimental (blue) isotope patterns for the three positive charge state of **TPACage•**6AsF$_6$.

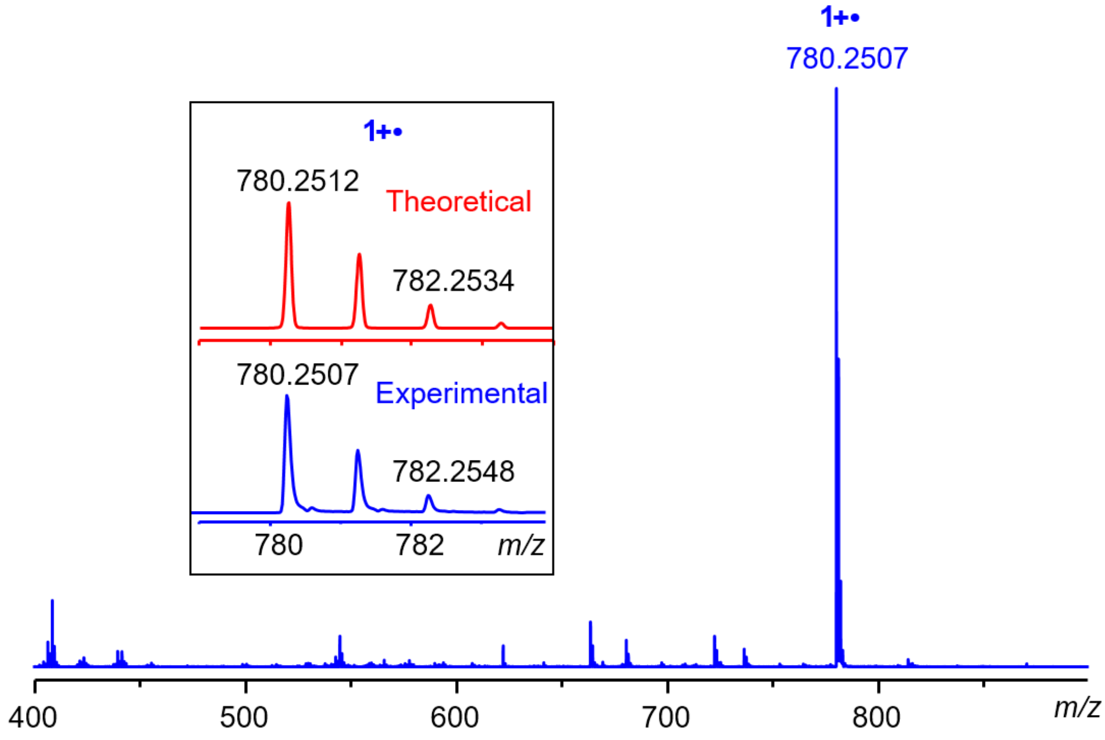

Supplementary Figure 39. HRMS of **3H-HBC**. Inset shows the theoretical (red) and experimental (blue) isotope patterns for the cationic radical charge state of **3H-HBC**.

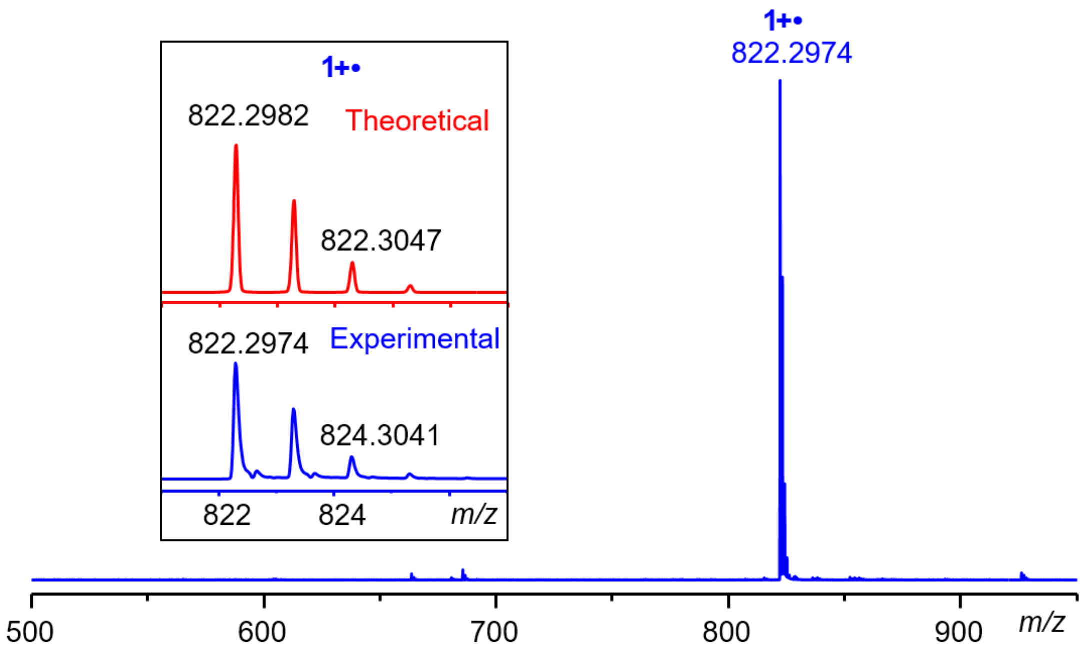

Supplementary Figure 40. HRMS of **3Me-HBC**. Inset shows the theoretical (red) and experimental (blue) isotope patterns for the cationic radical charge state of **3Me-HBC**.

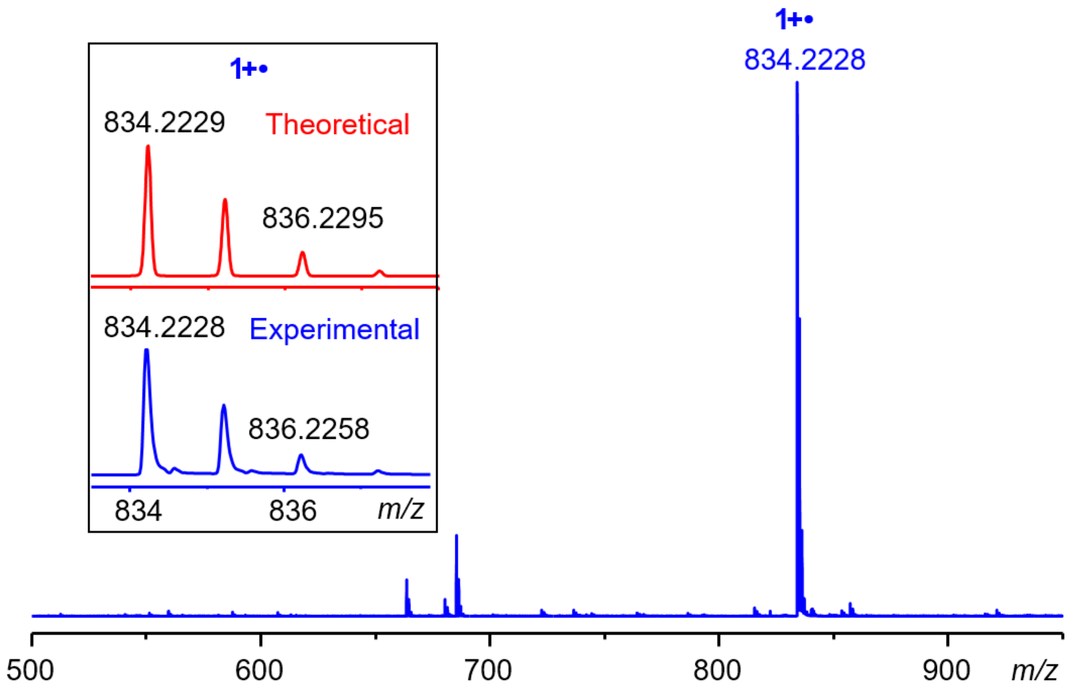

Supplementary Figure 41. HRMS of **3F-HBC**. Inset shows the theoretical (red) and experimental (blue) isotope patterns for the cationic radical charge state of **3F-HBC**.

(2) High-resolution mass spectrometry (HRMS) of the four host-guest complexes

In the mass spectra of four host-guest complexes (Supplementary Figs. 42–45), signals for free host cage, i.e., *m/z* = 427.65, *m/z* = 618.53 were also observed as a result of the dissociation of the complexes during mass spectrometric measurements. The blue "**4+**" and "**3+**" represent the four and three positively charged states of the free **TPACage**•6PF_6, while the brown "**5+**" "**4+**"and "**3+**" represent the five, four and three positively charged states of the host-guest complexes, respectively.

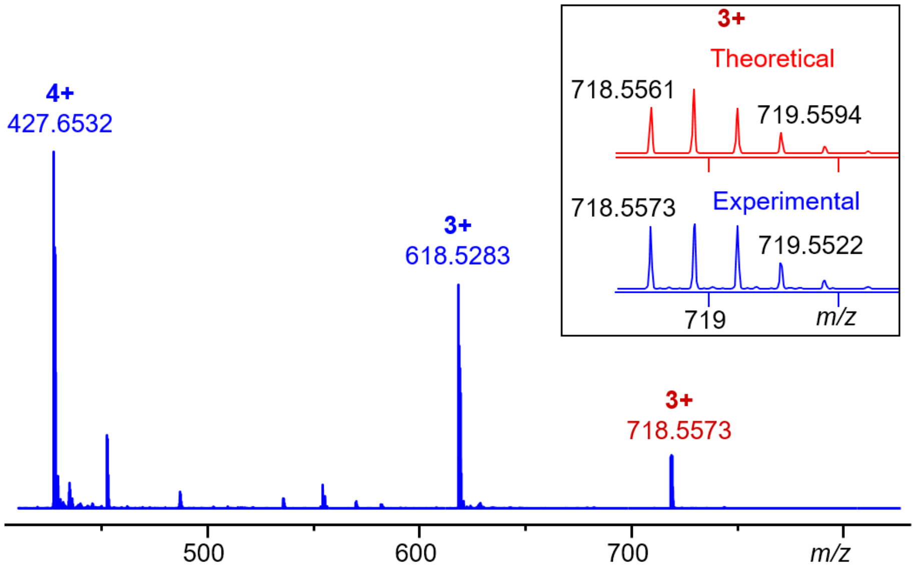

Supplementary Figure 42. HRMS of **COR⊂TPACage**•6PF_6. Inset shows the theoretical (red) and experimental (blue) isotope patterns for the three positive charge state of **COR⊂TPACage**•6PF_6.

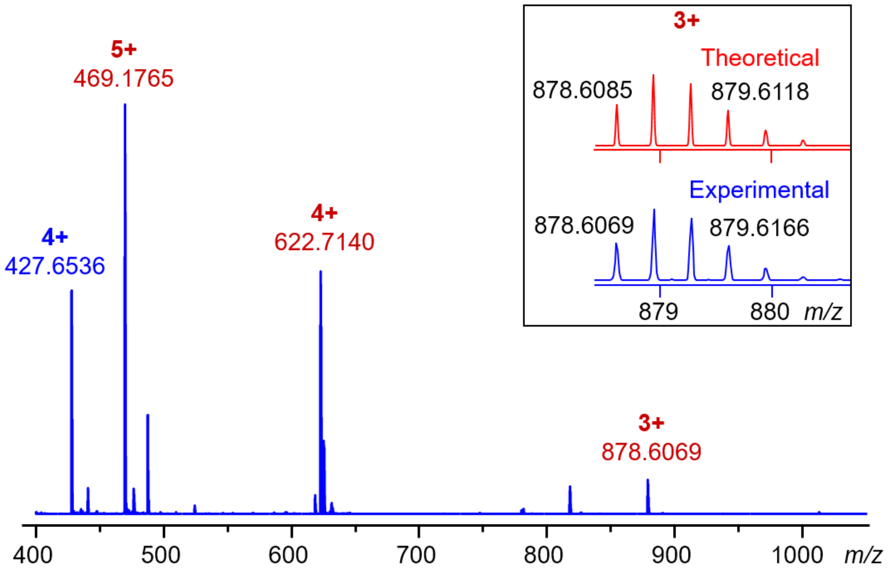

Supplementary Figure 43. HRMS of **3H-HBC⊂TPACage**•6PF_6. Inset shows the theoretical (red) and experimental (blue) isotope patterns for the three positive charge state of **3H-HBC⊂TPACage**•6PF_6.

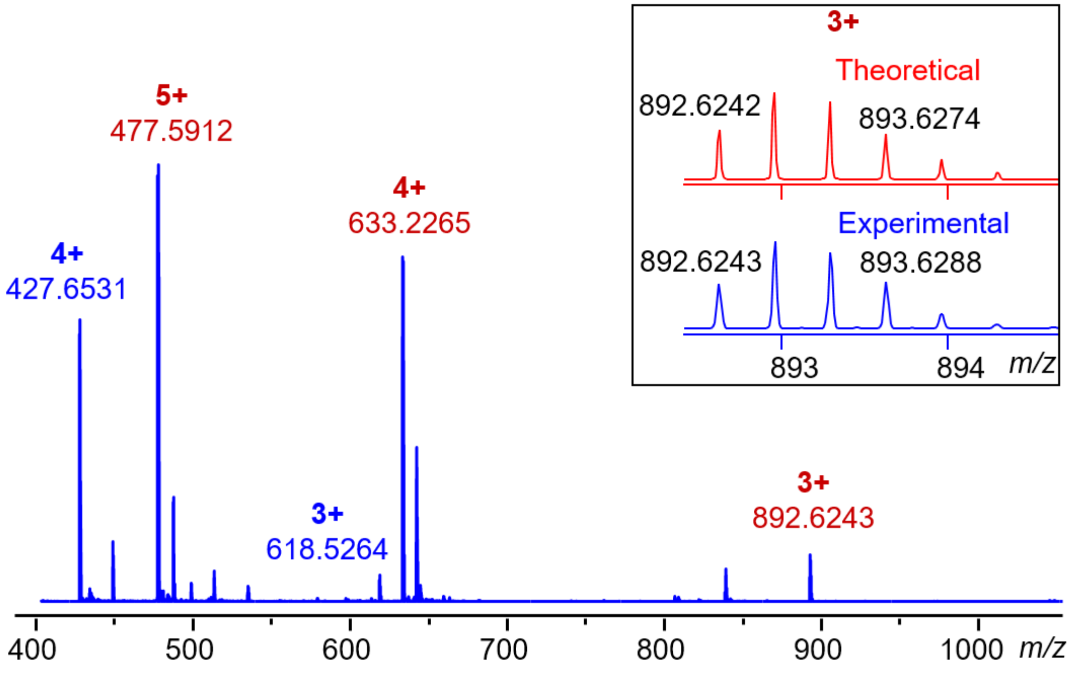

Supplementary Figure 44. HRMS of **3Me-HBC⊂TPACage**•6PF_6. Inset shows the theoretical (red) and experimental (blue) isotope patterns for the three positive charge state of **3Me-HBC⊂TPACage**•6PF_6.

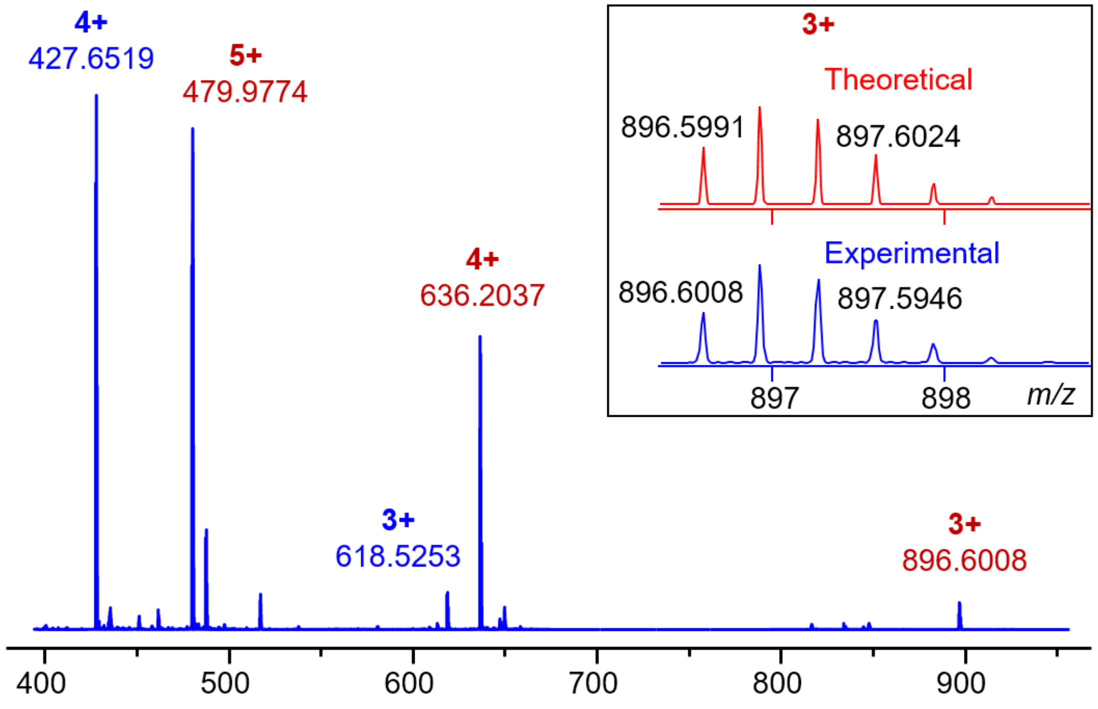

Supplementary Figure 45. HRMS of **3F-HBC⊂TPACage**•6PF_6. Inset shows the theoretical (red) and experimental (blue) isotope patterns for the three positive charge state of **3F-HBC⊂TPACage**•6PF_6.

Supplementary Note 4. Photophysical Characterization

(1) UV−Vis Titration and Job plot between TPACage^{6+} and *c*-HBC guests

In the case of UV-Vis titration experiments, a 1mM $CHCl_3$ solution of ***c*-HBC** guests as the titrating solution was added dropwise to a micromolar solution of **TPACage•**$6PF_6$ in MeCN / $CHCl_3$ (4:1).

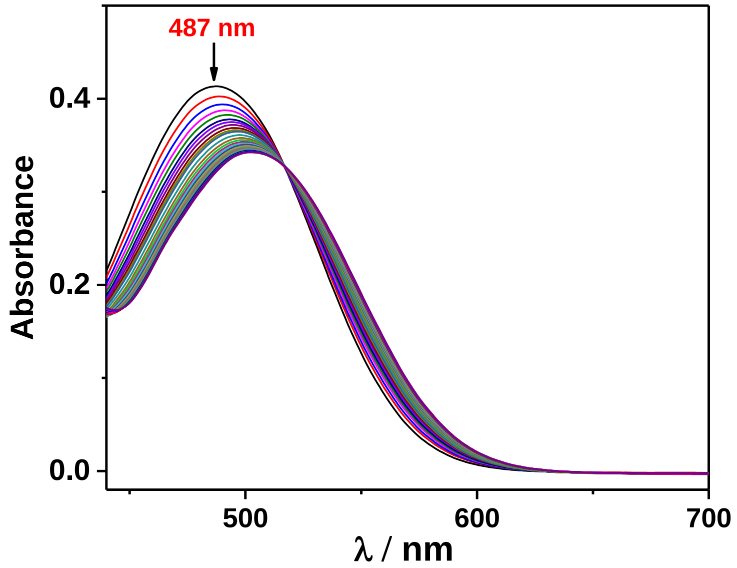

Supplementary Figure 46. UV-Vis Spectroscopic changes of **TPACage^{6+}** ([**TPACage^{6+}**] = 2.5 × 10^{-6} M, [**3H-HBC**] / [**TPACage^{6+}**] = 0 – 9.2 equiv, MeCN:$CHCl_3$ = 4:1, optical path: 10 mm, 298 K) upon dropwise addition of **3H-HBC**. Note that a series of **3H-HBC** solutions of the same concentration were used as references.

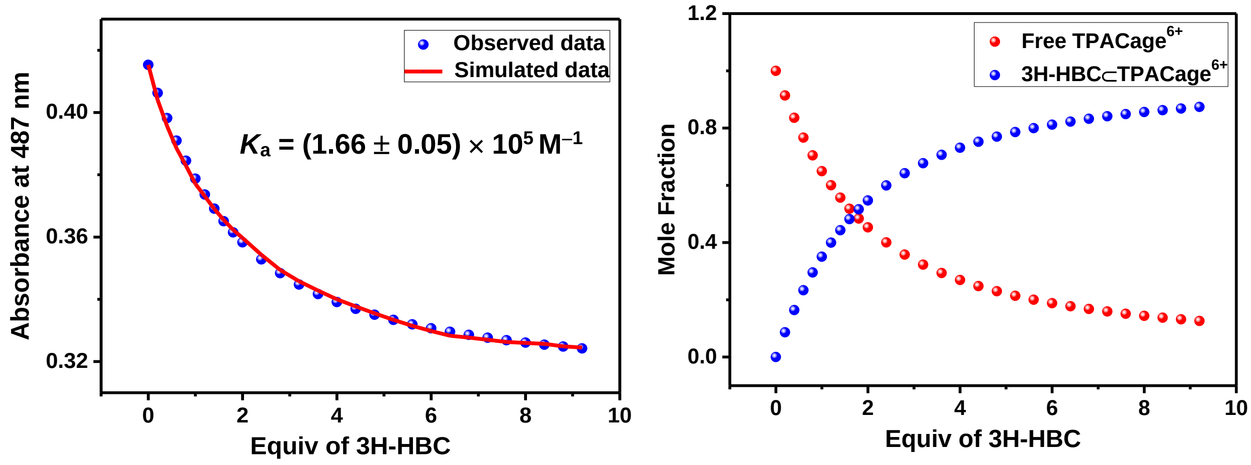

Supplementary Figure 47. (a) Nonlinear least-squares analysis of the absorbance intensity changes at 487 nm used to calculate the K_a value between **TPACage^{6+}** and **3H-HBC** in a UV-Vis titration experiment as shown in Supplementary Fig. 46. (b) Mole fractions are based on fitting results, indicating that the concentration of the free **TPACage^{6+}** undergoes a continuous decrease (red trace), while the concentration of **3H-HBC⊂TPACage^{6+}** complex undergoes a continuous increase (blue trace).

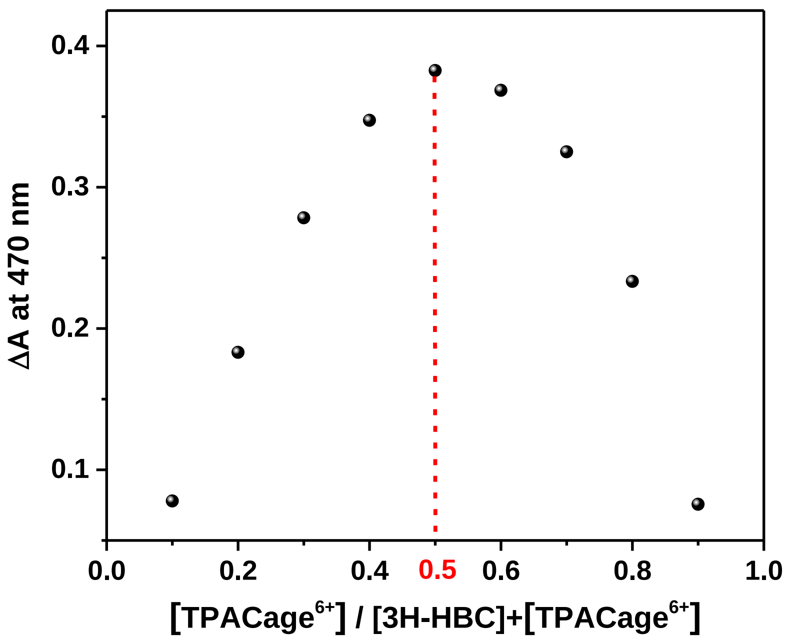

Supplementary Figure 48. Job-plot analysis of the stoichiometric ratio between **TPACage^{6+}** and **3H-HBC** in a solution of MeCN / $CHCl_3$ (4:1) at 298 K. Absorbance intensity changes of **TPACage^{6+}** recorded at 470 nm was used to analyze the binding ratio. The total concentration of host and guest is held constant ([**TPACage^{6+}**] + [**3H-HBC**] = 2.0×10^{-4} M).

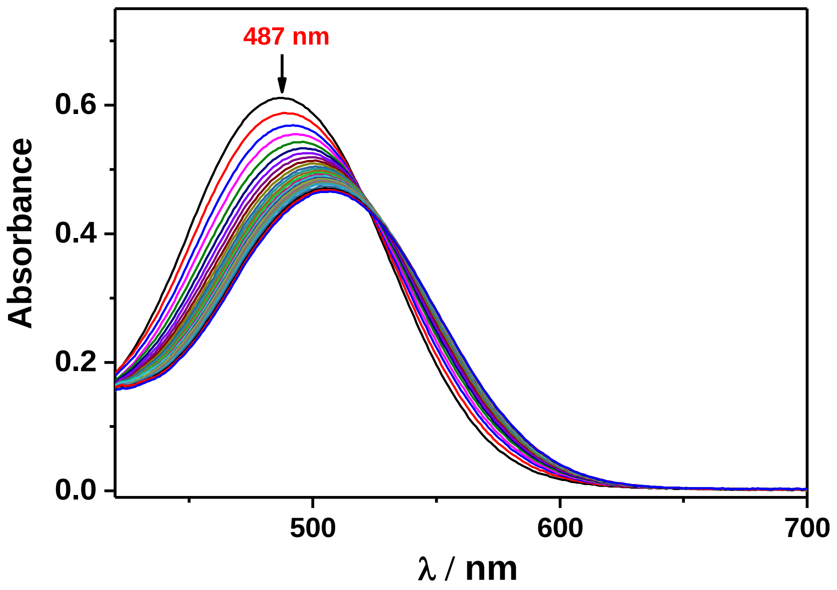

Supplementary Figure 49. UV-Vis Spectroscopic changes of **TPACage^{6+}** ([**TPACage^{6+}**] = 4.0×10^{-6} M, [**3Me-HBC**] / [**TPACage^{6+}**] = 0 – 6 equiv, MeCN:$CHCl_3$ = 4:1, optical path: 10 mm, 298 K) upon dropwise addition of **3Me-HBC**. Note that a series of **3Me-HBC** solutions of the same concentration were used as references.

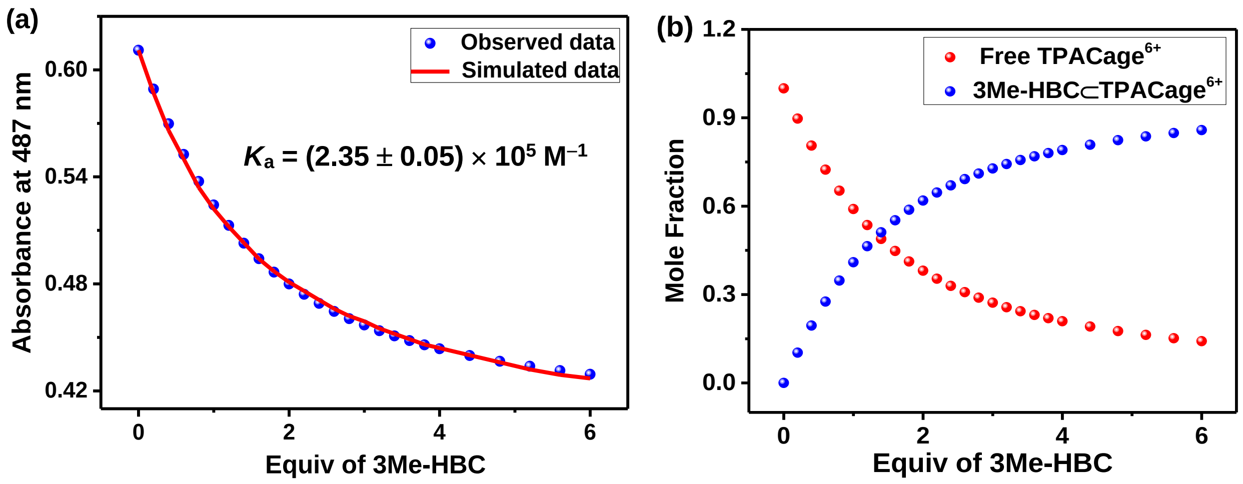

Supplementary Figure 50. (a) Nonlinear least-squares analysis of the absorbance intensity changes at 487 nm used to calculate the K_a value between **TPACage^{6+}** and **3Me-HBC** in a UV-Vis titration experiment as shown in Supplementary Fig. 49. (b) Mole fractions are based on fitting results, indicating that the concentration of the free **TPACage^{6+}** undergoes a continuous decrease (red trace), while the concentration of **3Me-HBC⊂TPACage^{6+}** complex undergoes a continuous increase (blue trace).

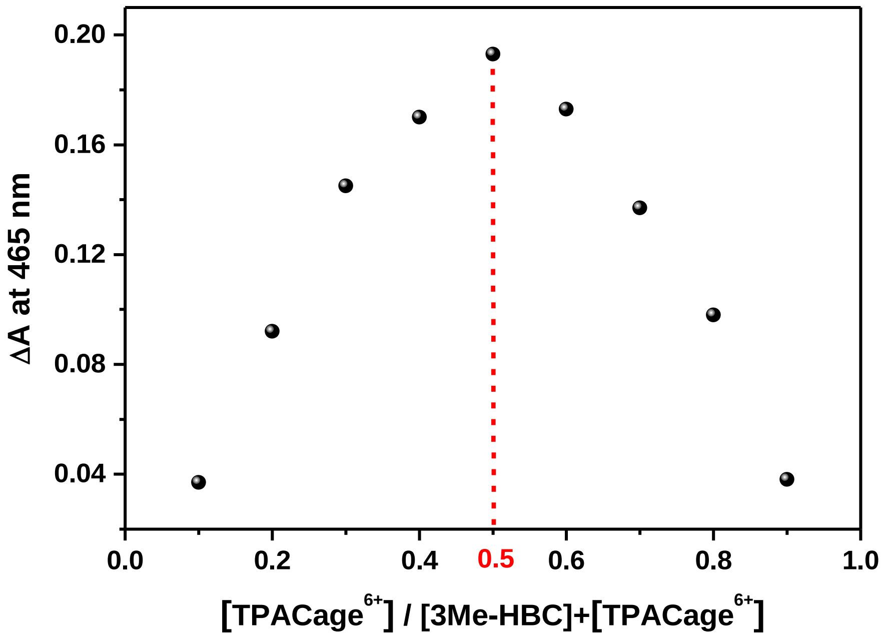

Supplementary Figure 51. Job-plot analysis of the stoichiometric ratio between **TPACage^{6+}** and **3Me-HBC** in a solution of MeCN / $CHCl_3$ (4:1) at 298 K. Absorbance intensity changes of **TPACage^{6+}** recorded at 465 nm was used to analyze the binding ratio. The total concentration of host and guest is held constant ([**TPACage^{6+}**] + [**3Me-HBC**] = 1.0 × 10^{-4} M).

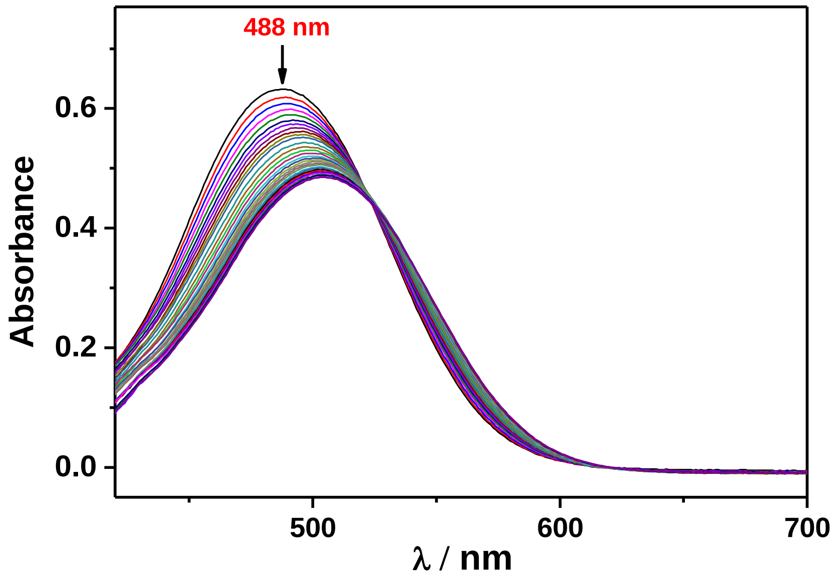

Supplementary Figure 52. UV-Vis Spectroscopic changes of **TPACage^{6+}** ([**TPACage^{6+}**] = 4.0 × 10^{-6} M, [**3F-HBC**] / [**TPACage^{6+}**] = 0 – 10 equiv, MeCN:$CHCl_3$ = 4:1, optical path: 10 mm, 298 K) upon dropwise addition of **3F-HBC**. Note that a series of **3F-HBC** solutions of the same concentration were used as references.

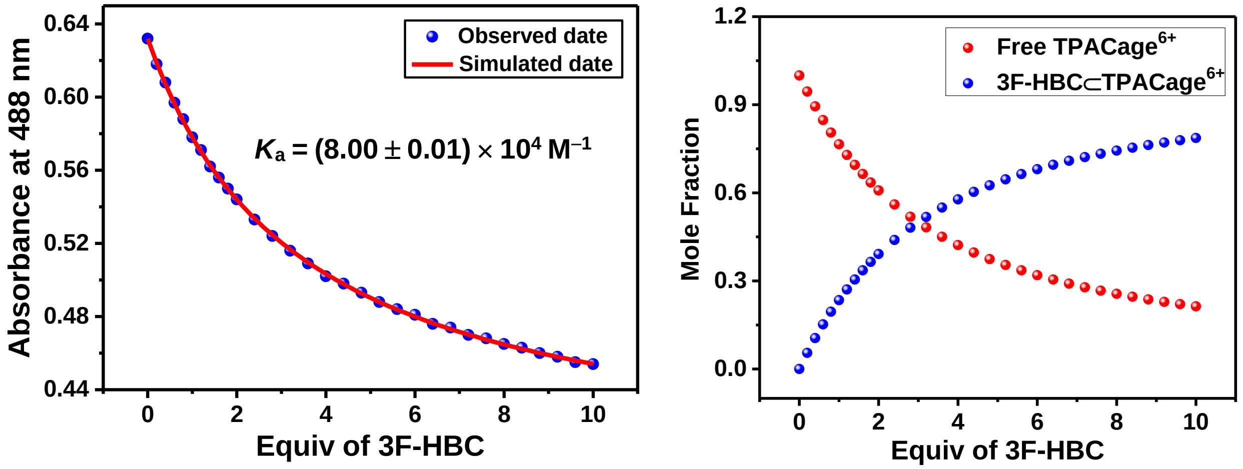

Supplementary Figure 53. (a) Nonlinear least-squares analysis of the absorbance intensity changes at 488 nm used to calculate the K_a value between **TPACage^{6+}** and **3F-HBC** in a UV-Vis titration experiment as shown in Supplementary Fig. 52. (b) Mole fractions are based on fitting results, indicating that the concentration of the free **TPACage^{6+}** undergoes a continuous decrease (red trace), while the concentration of **3F-HBC⊂TPACage•**$6PF_6$ complex undergoes a continuous increase (blue trace).

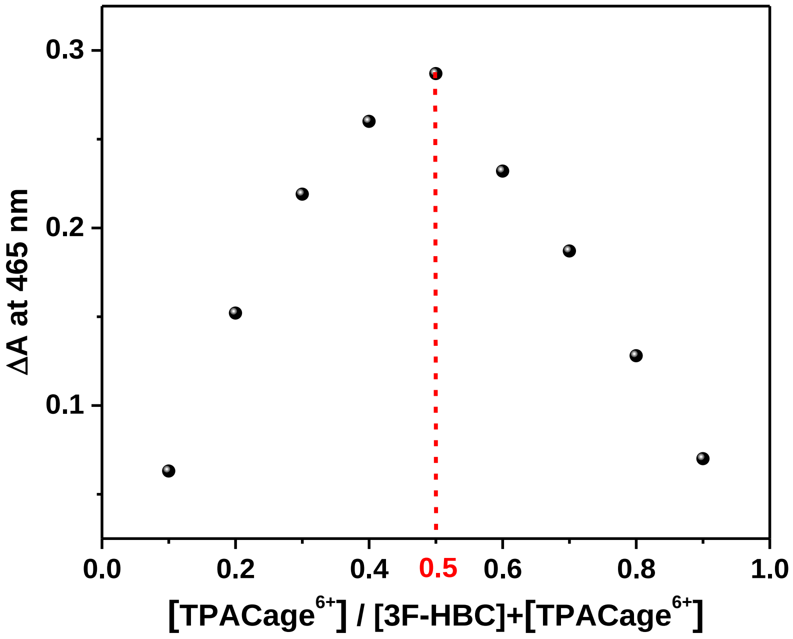

Supplementary Figure 54. Job-plot analysis of the stoichiometric ratio between **TPACage^{6+}** and **3F-HBC** in a solution of MeCN / $CHCl_3$ (4:1) at 298 K. Absorbance intensity changes of **TPACage^{6+}** recorded at 465 nm was used to analyze the binding ratio. The total concentration of host and guest is held constant ([**TPACage^{6+}**] + [**3F-HBC**] = 2.0×10^{-4} M).

(2) Fluorescence spectroscopic analysis

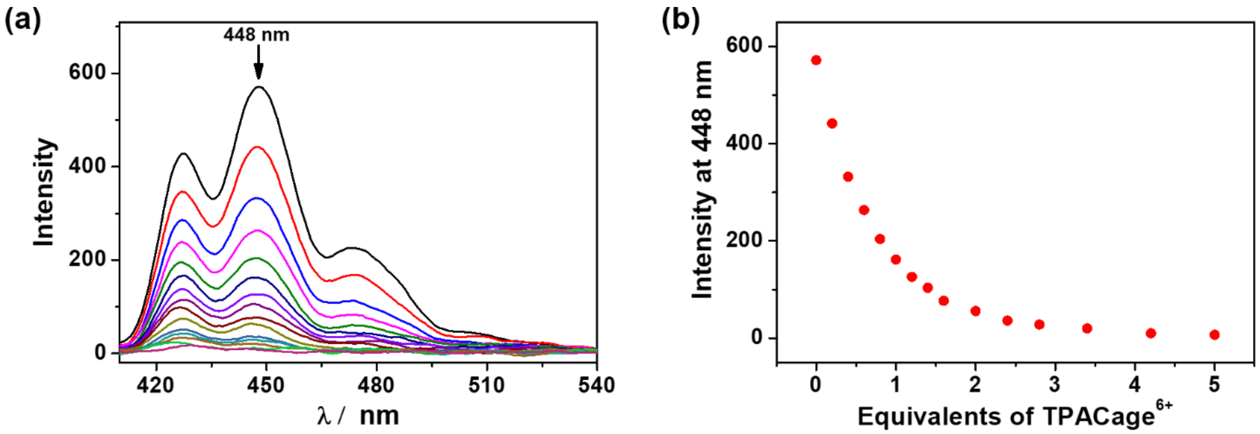

Supplementary Figure 55. (a) Emission spectroscopic changes of **COR** ([**COR**] = 5×10^{-6} M, [**TPACage^{6+}**] = 0−2.5×10^{-5} M, λ_{ex} = 302 nm, MeCN:$CHCl_3$ = 4:1, 298 K), and (b) fluorescence intensity changes at 448 nm upon dropwise addition of **TPACage^{6+}**.

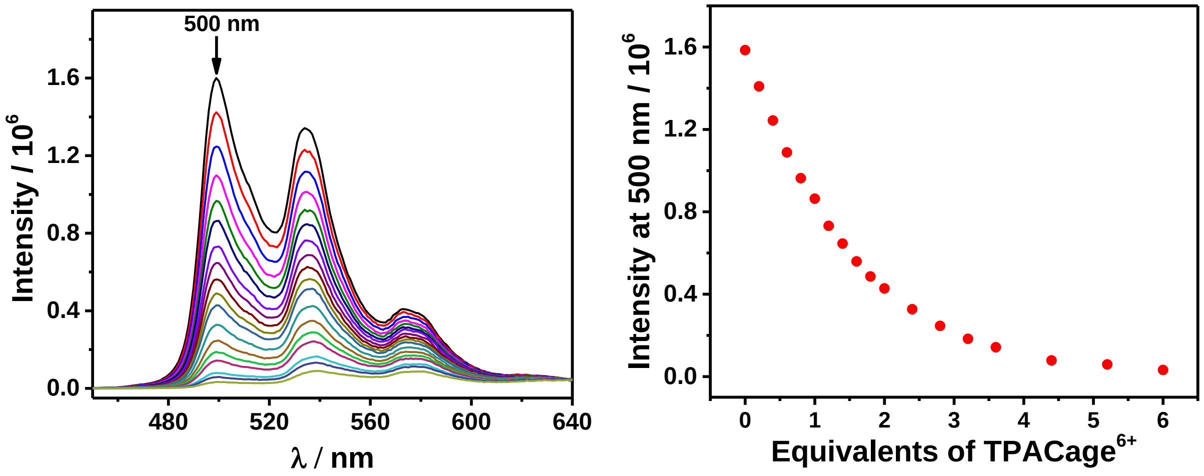

Supplementary Figure 56. (a) Emission spectroscopic changes of **3H-HBC** ([**3H-HBC**] = 2.5 × 10^{-6} M, [**TPACage^{6+}**] = 0−1.5 × 10^{-5} M, λ_{ex} = 384 nm, MeCN:$CHCl_3$ = 4:1, 298 K), and (b) fluorescence intensity changes at 500 nm upon dropwise addition of **TPACage^{6+}**.

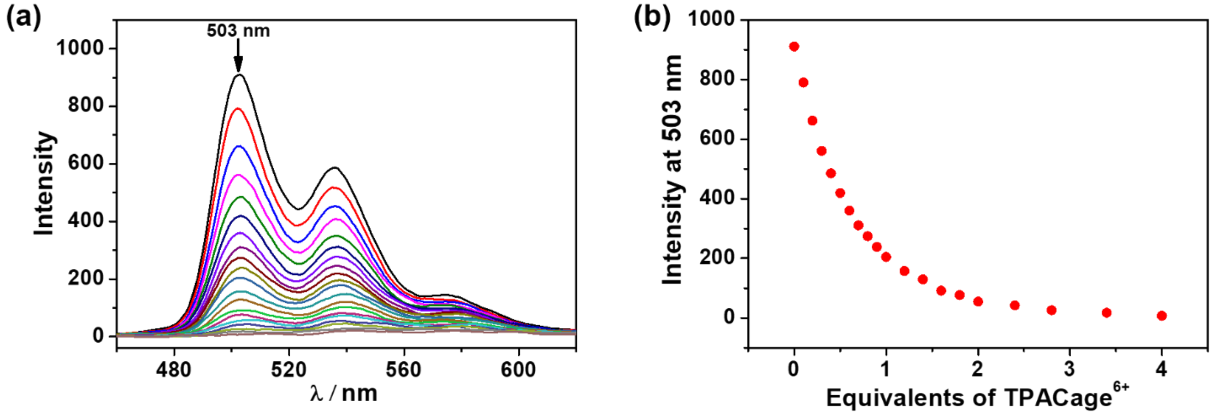

Supplementary Figure 57. (a) Emission spectroscopic changes of **3Me-HBC** ([**3Me-HBC**] = 5 × 10^{-6} M, [**TPACage^{6+}**] = 0−2.0 × 10^{-5} M, λ_{ex} = 384 nm, MeCN:$CHCl_3$ = 4:1, 298 K), and (b) fluorescence intensity changes at 503 nm upon dropwise addition of **TPACage^{6+}**.

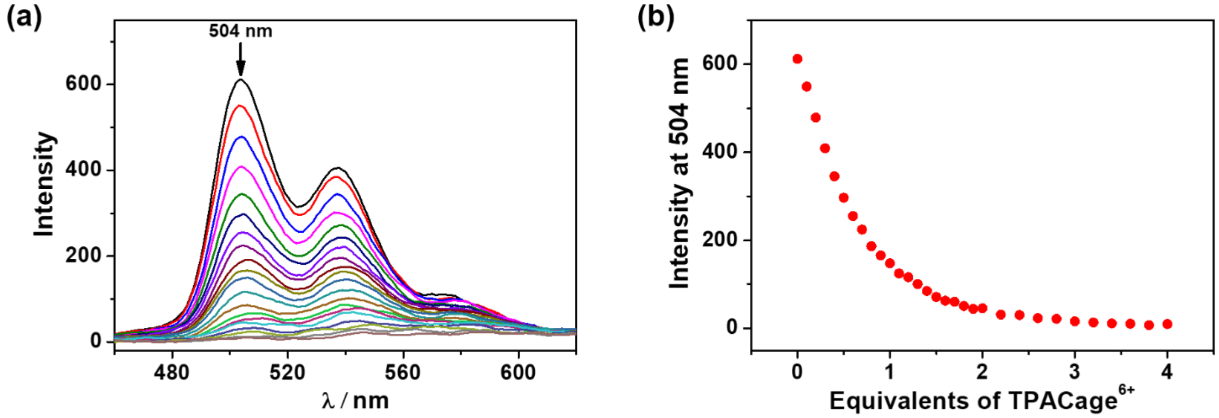

Supplementary Figure 58. (a) Emission spectroscopic changes of **3F-HBC** ([**3F-HBC**] = 5 × 10^{-6} M, [**TPACage^{6+}**] = 0−2.0 × 10^{-5} M, λ_{ex} = 384 nm, MeCN:$CHCl_3$ = 4:1, 298 K), and (b) fluorescence intensity changes at 504 nm upon dropwise addition of **TPACage^{6+}**.

(3) Binding kinetics study

The binding kinetics were obtained by following the change of absorbance at 487 nm with time after mixing equal molar amounts of ***c*-HBC** and **TPACage^{6+}**. The resulting kinetic was fitted[2] according to the second order kinetics model. The binding kinetics between **3H-HBC** and **TPACage^{6+}** are very fast, we can only capture part of signals in absorbance decrease.

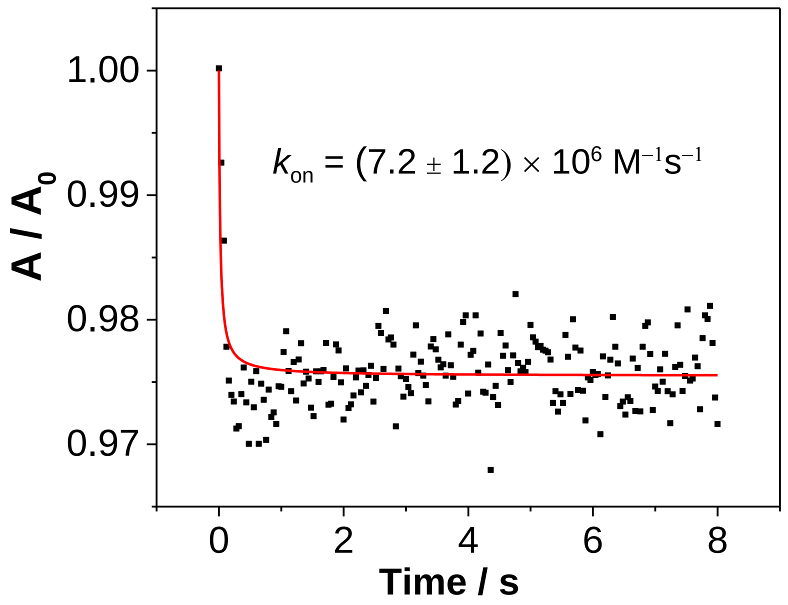

Supplementary Figure 59. Change of absorbance at 487 nm over time upon the mixing equimolar amounts of **3H-HBC** (6 μ M) and **TPACage^{6+}** (6 μ M) in a solution of MeCN / $CHCl_3$ (4:1), and the corresponding non-linear fitting curve for the determination of the association rate constant k_{on}.

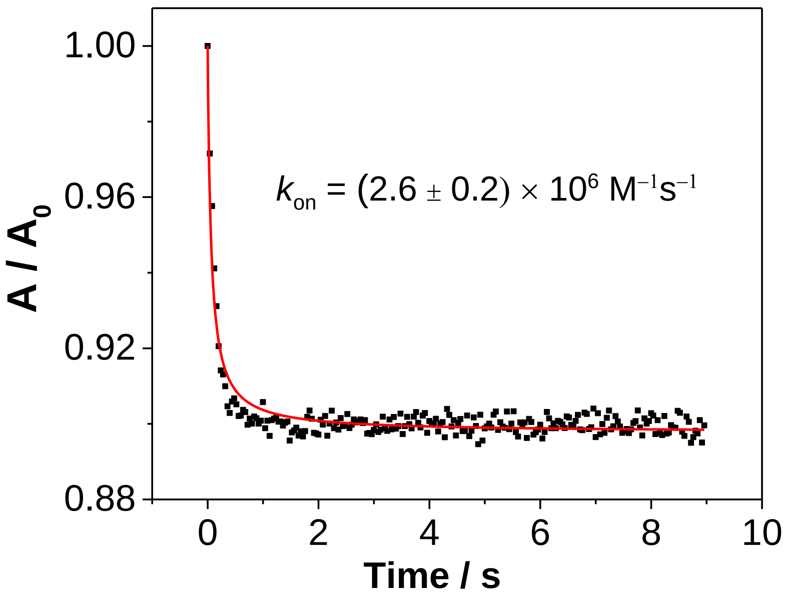

Supplementary Figure 60. Change of absorbance at 487 nm over time upon the mixing equimolar amounts of **3Me-HBC** (6 μ M) and **TPACage^{6+}** (6 μ M) in a solution of MeCN / $CHCl_3$ (4:1), and the corresponding non-linear fitting curve for the determination of the association rate constant k_{on}.

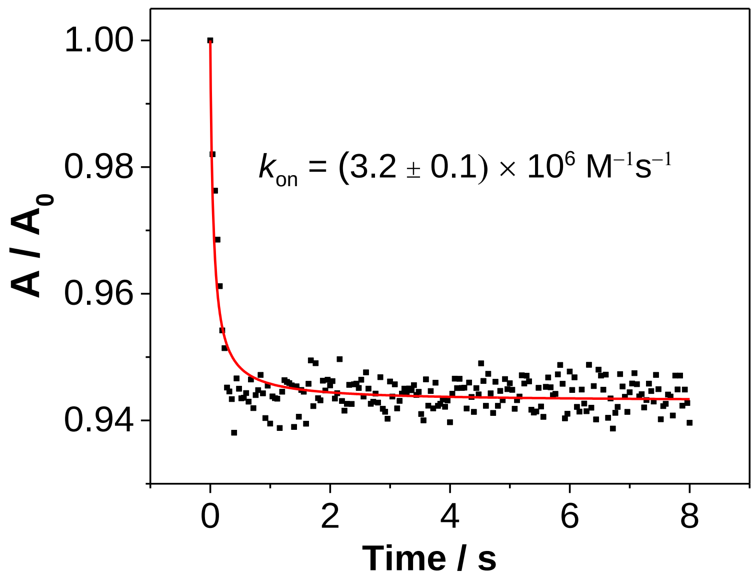

Supplementary Figure 61. Change of absorbance at 487 nm over time upon the mixing equimolar amounts of **3F-HBC** (6 μ M) and **TPACage^{6+}** (6 μ M) in a solution of MeCN / $CHCl_3$ (4:1), and the corresponding non-linear fitting curve for the determination of the association rate constant k_{on}.

Supplementary Table 1 The changes of fluorescence quantum yields for guests upon adding 5 equiv of TPACage^{6+} in MeCN / $CHCl_3$ (4:1)[a]

Entry	λ_{ex} / nm	λ_{em} / nm	Quantum Yield / %
**COR**	302	448	1.76
**COR⊂TPACage^{6+}**	302	448	0.17
**3H-HBC**	384	500	3.77
**3H-HBC⊂TPACage^{6+}**	384	500	0.63
**3Me-HBC**	384	503	3.66
**3Me-HBC⊂TPACage^{6+}**	384	503	0.85
**3F-HBC**	384	504	3.65
**3F-HBC⊂TPACage^{6+}**	384	504	0.29

[a]The quantum yield was recorded using a 10 × 10 × 45 mm quartz cell and the concentrations of guests are 5 × 10^{-7} M.

Supplementary Note 5. Isothermal Titration Calorimetry

All microcalorimetric titrations were performed using a thermostated TA Nano Isothermal Titration Calorimeter at atmospheric pressure and 298 K. The samples were dissolved in MeCN / $CHCl_3$ (4:1) and allowed to equilibrate overnight before use. A solution of **TPACage**•6PF_6 in a syringe was injected with stirring at 75 rpm into a solution of ***c*-HBC** guest molecules in the sample cell with an active volume of 185 µL. Hindered by (i) the relatively low solubility of ***c*-HBC** guests in MeCN / $CHCl_3$ (4:1) and (ii) the small enthalpy changes upon forming the complexes, we found it is difficult to obtain accurate binding constants using a continuous calorimetric titration protocol after multiple attempts. We explored the independent single injection experiments[2] to estimate the binding enthalpies for the formation of the host-guest complexes. The net reaction heat was obtained by subtracting the dilution heat from the apparent reaction heat.

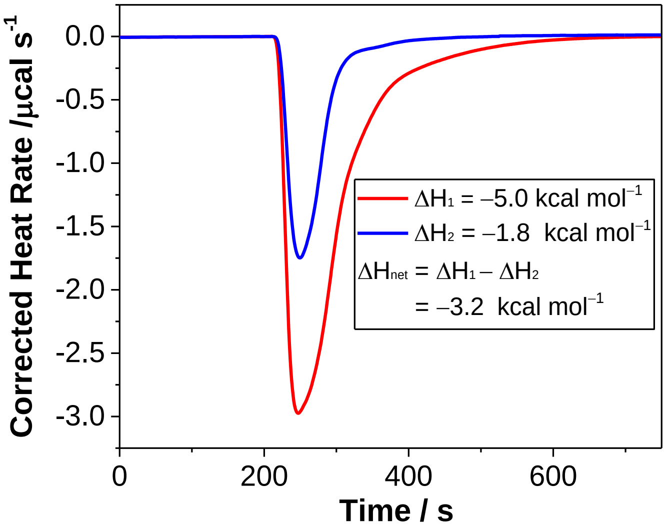

Supplementary Figure 62. ITC Single injection of **TPACage**•6PF_6 (3×10^{-3} M) into MeCN / $CHCl_3$ (4:1) solution (blue curve) or a solution of **3H-HBC** (3×10^{-4} M, red curve). The ΔH_1, ΔH_2 and ΔH_{Net} are represented as apparent reaction heat, dilution heat and net reaction heat, respectively. The binding enthalpy (ΔH_{Net}) for the formation of **3H-HBC⊂TPACage**•6PF_6 complex was estimated to be -3.2 kcal mol^{-1}.

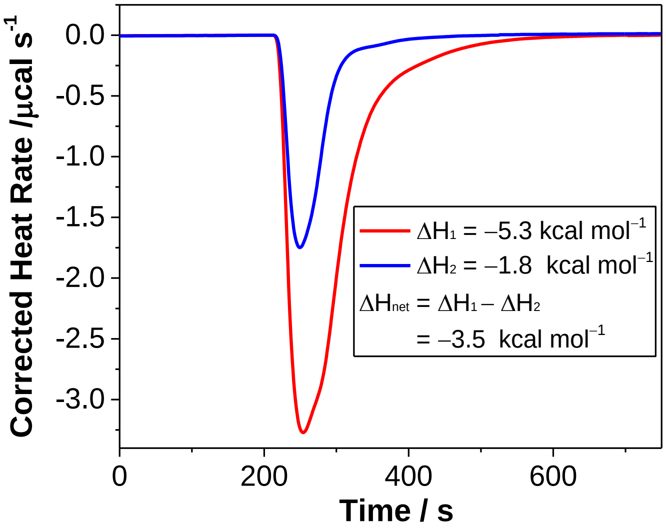

Supplementary Figure 63. ITC Single injection of **TPACage**•6PF$_6$ (3 × 10^{-3} M) into MeCN / CHCl$_3$ (4:1) solution (blue curve) or a solution of **3Me-HBC** (3 × 10^{-4} M, red curve). The ΔH_1, ΔH_2 and ΔH_{Net} are represented as apparent reaction heat, dilution heat and net reaction heat, respectively. The binding enthalpy (ΔH_{Net}) for the formation of **3Me-HBC⊂TPACage**•6PF$_6$ complex was estimated to be –3.5 kcal mol^{-1}.

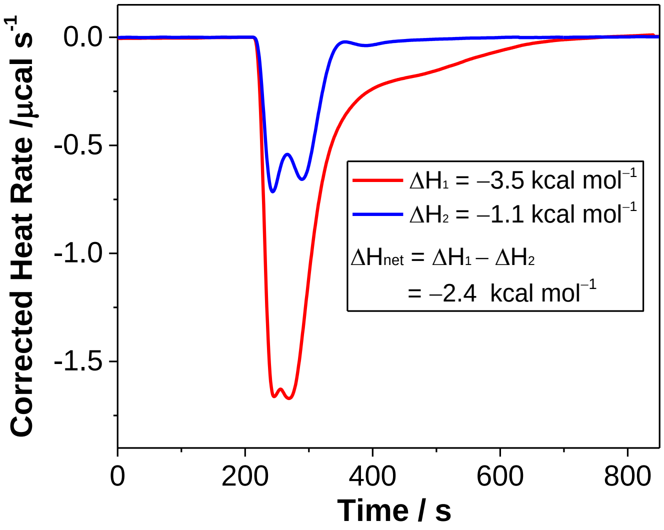

Supplementary Figure 64. ITC Single injection of **TPACage**•6PF$_6$ (3 × 10^{-3} M) into MeCN / CHCl$_3$ (4:1) solution (blue curve) or a solution of **3F-HBC** (3 × 10^{-4} M, red curve). The ΔH_1, ΔH_2 and ΔH_{Net} are represented as apparent reaction heat, dilution heat and net reaction heat, respectively. The binding enthalpy (ΔH_{Net}) for the formation of **3F-HBC⊂TPACage**•6PF$_6$ complex was estimated to be –2.4 kcal mol^{-1}.

Supplementary Note 6. Crystallographic Characterization

All crystallographic data are available free of charge from the Cambridge Crystallographic Data Centre (CCDC) via www.ccdc.cam.ac.uk/data_request/cif.

(1) TPACage•6Cl

(a) Method. **TPACage**•6Cl (1.6 mg, 1.0 μmol) was dissolved in MeOH (1.0 mL). The solution was passed through a 0.45-μm filter and added to three 1-mL tubes with the volumes of 0.20, 0.35, and 0.45 mL, respectively. The tubes were placed together in one 20-mL vial containing $i\mathrm{Pr_2O}$ (~3 mL) and the vial was capped. Slow vapor diffusion of $i\mathrm{Pr_2O}$ into the MeOH solution of **TPACage**•6Cl (1.0 mM) over the course of four days yielded dark red single crystals of **TPACage**•6Cl. A single crystal was mounted on a MITIGEN holder in Paratone oil on a Bruker APEX-II CCD diffractometer. The crystal was kept at 100.02 K during data collection. Using Olex2[3], the structure was solved with the ShelXT[4] structure solution program using Intrinsic Phasing and refined with XL[5] refinement package using Least Squares minimization. The solid-state (super)structure of **TPACage**•6Cl is shown in Fig. 2.

(b) Crystal Parameters. [$\mathrm{C_{102}H_{84}N_8 \cdot 6Cl}$]. Mr = 1634.47. Dark red block (0.137 × 0.032 × 0.025 $\mathrm{mm^3}$). Hexagonal, space group $P6_3/m$ (no. 176), a = 25.105(3), b = 25.105(3), c = 21.871(3) Å, α = 90.000, β = 90.000, γ = 120.000°, V = 11937(3) $\mathrm{Å^3}$, Z = 2, T = 100.02 K, μ(CuKα) = 0.805 $\mathrm{mm^{-1}}$, $Dcalc$ = 0.455 g $\mathrm{mm^{-3}}$, 18074 reflections measured ($5.732 \leq 2\Theta \leq 89.028$), 3248 unique ($R_{\mathrm{int}}$ = 0.0843, R_{sigma} = 0.0623) which were used in all calculations. The final R_1 was 0.0816 ($I > 2\sigma(I)$) and wR_2 was 0.2567 (all data). CCDC Number: 2045289.

(c) Solvent Treatment Details. The solvent masking procedure as implemented in Olex2 was used to remove the electronic contribution of solvent molecules from the refinement. As the exact solvent content is not known, only the atoms used in the refinement model are reported in the formula here. Total solvent accessible volume / cell = 8634.5 $\mathrm{Å^3}$ [71.7%], Total electron count / cell = 1443.4.

(2) 3H-HBC

(a) Method. **3H-HBC** (1.6 mg, 2.0 μmol) was dissolved in a 4:1 PhMe / $\mathrm{CHCl_3}$ solution (1 mL). The solution was passed through a 0.45-μm filter and added to two 1-mL tubes with the volume of 0.30 and 0.70 mL, respectively. The tubes were placed together in one 20-mL vial without capping.

The yellow single crystals of **3H-HBC** were obtained by slow evaporation over the course of five days. A single crystal was mounted on a MITIGEN holder in Paratone oil on a XtaLAB Synergy R, DW system, HyPix diffractometer. The crystal was kept at 100.01 K during data collection. Using Olex2[3], the structure was solved with the ShelXT[4] structure solution program using Intrinsic Phasing and refined with XL[5] refinement package using Least Squares minimization. The solid-state structure of **3H-HBC** is shown in Fig. 3e.

(b) Crystal Parameters. [$C_{54}H_{36}O_6\cdot 2(C_7H_8)$].*M*r = 965.09. Yellow block (0.182 × 0.054 × 0.019 mm^3). Triclinic, space group $P\bar{1}$ (no. 2), a = 10.5810(2), b = 14.1629(2), c = 17.1788(3) Å, α = 72.891(2), β = 79.986(2), γ = 78.4470(10)°, V = 2392.36(8) Å^3, Z = 2, T = 100.01(10) K, μ(CuKα) = 0.666 mm^{-1}, *Dcalc* = 1.340 g/mm^3, 45679 reflections measured (5.424 ≤ 2Θ ≤ 157.226), 9830 unique (R_{int} = 0.0357, R_{sigma} = 0.0293) which were used in all calculations. The final R_1 was 0.0509 ($I > 2\sigma(I)$) and wR_2 was 0.1509 (all data). CCDC Number: 2045282.

(c) Refinement Details. Distance restraints were imposed on the disordered toluene solvent molecule. The enhanced rigid-bond restraint was also applied to the disordered solvent.

(d) Solvent Treatment Details: None.

(3) COR⊂TPACage•6Cl

(a) Method. The $CHCl_3$ (0.25 mL) solution of **COR** (0.9 mg, 3.0 µmol) was added to a MeOH (1 mL) solution of **TPACage**•6Cl (1.6 mg, 1.0 µmol). This solution was passed through a 0.45-µm filter and added to two 1-mL tubes with the volumes of 0.20 and 0.45 mL. The tubes were placed together in one 20-mL vial containing iPr$_2$O (~3 mL) and the vial was capped. Slow vapor diffusion of iPr$_2$O into the solution of **COR** and **TPACage**•6Cl in MeOH:$CHCl_3$ (4:1) over the period of three days yielded dark red single crystals of **COR⊂TPACage**•6Cl. A suitable crystal was selected and the crystal was mounted on a MITIGEN holder with Paratone oil on a XtaLAB Synergy R, DW system, HyPix diffractometer. The crystal was kept at 175.01(11) K during data collection. Using Olex2[3], the structure was solved with the XT[4] structure solution program using Intrinsic Phasing and refined with the XL[5] refinement package using Least Squares minimization. The solid-state superstructure of **COR⊂TPACage**•6Cl is shown in Fig.6 and Supplementary Fig. 65.

(b) Crystal Parameters. [$C_{24}H_{12}\subset C_{102}H_{84}N_8\cdot Cl_6$]. *M*r =1934.76. Dark red block (0.18 × 0.137 × 0.042 mm^3). Hexagonal, space group P6$_3$/m (no. 176), a = 25.4158(16), b = 25.4158(16), c = 22.180(3), α = 90.000, β = 90.000, γ = 120.000°, V = 12408(2) Å^3, Z = 2.00004, T = 175.01(11) K,

μ(CuKα) = 0.809 mm^{-1}, *Dcalc* = 0.518 g mm^{-3}, 15391 reflections measured (5.656 ≤ 2Θ ≤ 100.842), 4291 unique (R_{int} = 0.0876, R_{sigma} = 0.0527) which were used in all calculations. The final R_1 was 0.2540 ($I > 2\sigma(I)$) and wR_2 was 0.5786 (all data). CCDC Number: 2045290.

(c) Refinement Details. Distance restraints were imposed on the disordered **COR**. The enhanced rigid-bond restraint was applied to the **COR** as well as restraints on similar amplitudes (esd 0.05) separated by less than 1.7 Å and restraints that its Uij components approximate to isotropic.

(d) Solvent Treatment Details: None.

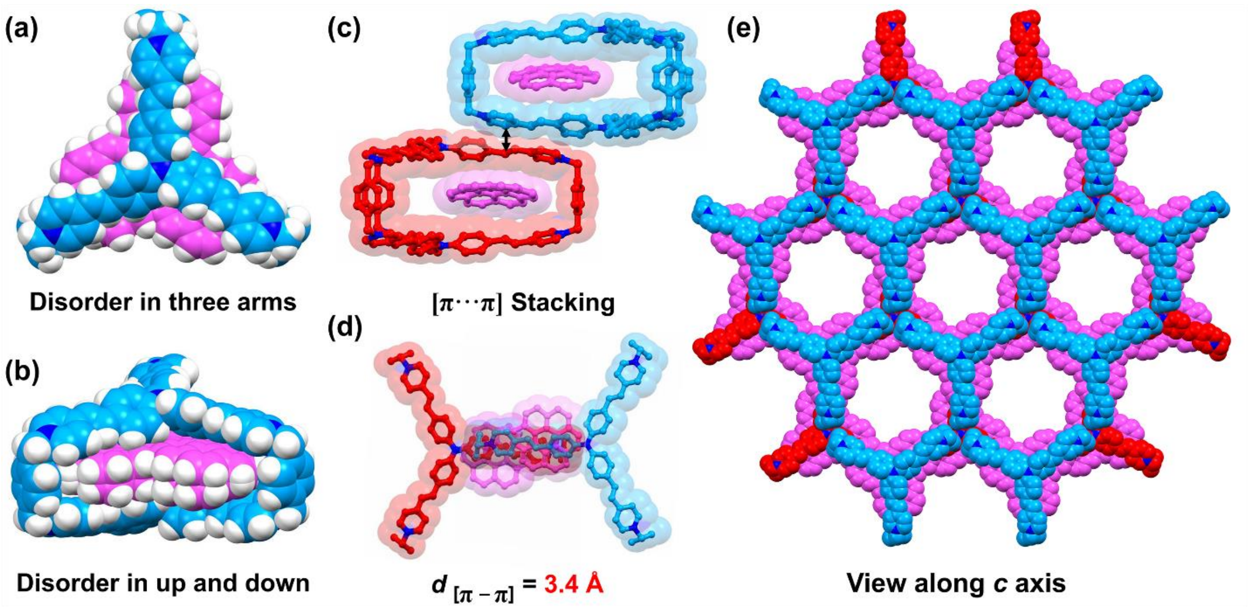

Supplementary Figure 65. Space-filling and ball-and-stick representations of the solid-state superstructure of **COR⊂TPACage^{6+}**. (a, b) Different crystallographic views of disordered **COR** inside the cavity of **TPACage^{6+}** showing that **COR** resides in any of the three pairs of styrene-pyridinium arms formed slots. The **COR** also can move up-and-down between two positions in each slot. (c, d) Different crystallographic views of [π···π] stacking interaction between two adjacent host-guest complexes. (e) Solid-state superstructure of **COR⊂TPACage^{6+}**, revealing how the complexes assemble into a 3D framework with relatively large 1D channels. The counterions and solvent molecules are omitted for the sake of clarity. **TPACage^{6+}**: C skyblue and red, N blue, H white. **COR**: C pink, H white.

(4) 3H-HBC⊂TPACage•6AsF$_6$

(a) Method. **TPACage•**6AsF$_6$ (2.6 mg, 1.0 μmol) and **3H-HBC** (0.8 mg, 1.0 μmol) were dissolved in a solution of $MeNO_2$:$CHCl_3$ (4:1, 1mL). The resulting solution was passed through a

0.45-μm filter before being added to two 1-mL tubes with the volumes of 0.20 and 0.45 mL. The tubes were placed together in one 20-mL vial containing *i*Pr_2O (~3 mL). The vial was sealed with a cap. Slow vapor diffusion of *i*Pr_2O into the 1:1 solution of **3H-HBC** and **TPACage**•6AsF_6 over the period of three days yielded dark red single crystals of **3H-HBC⊂TPACage**•6AsF_6. A suitable orange crystal was mounted on a MITIGEN holder in Paratone oil on a Rigaku XtaLAB Synergy diffractometer, with a Cu Kα microsource and a shutter-less electronic-noise free Hybrid Photon Counting (HPC) detector. The crystal was kept at 200(2) K during data collection. Using Olex2[3], the structure was solved with the ShelXT[4] structure solution program using intrinsic phasing and refined with the ShelXL[5] refinement package using least squares minimization. The solid-state superstructure of **3H-HBC⊂TPACage**•6AsF_6 is shown in Fig.6 and Supplementary Fig. 66.

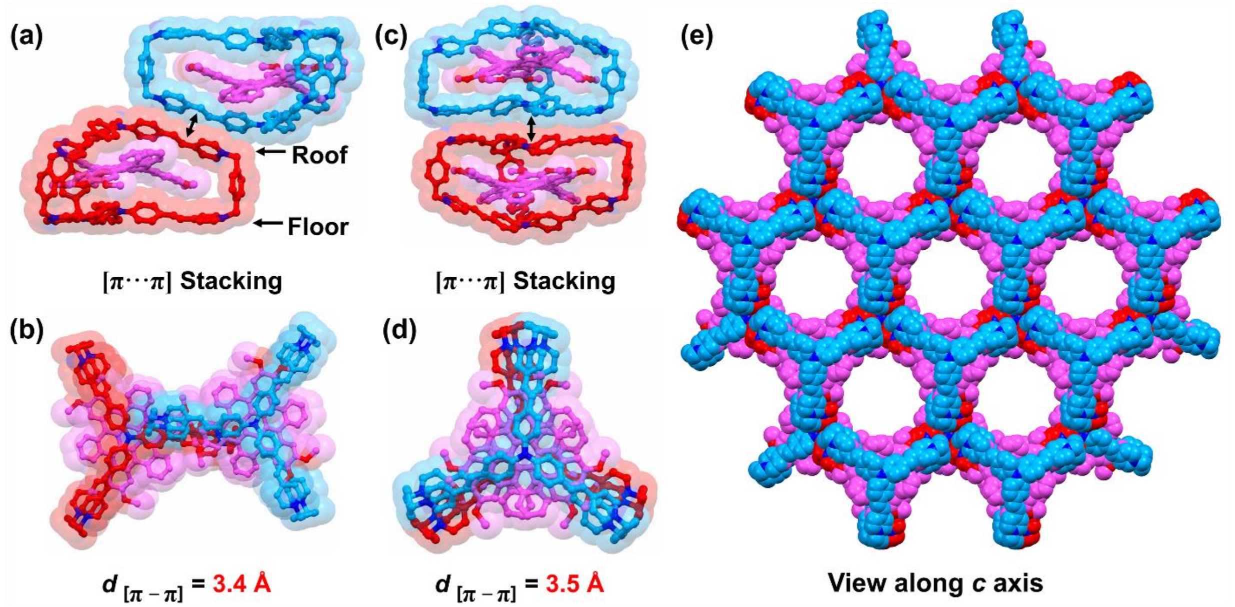

Supplementary Figure 66. Space-filling and ball-and-stick representations of the solid-state superstructure of **3H-HBC⊂TPACage^{6+}**. (a, b) Different crystallographic views of "roof" [π···π] stacking interaction between two adjacent host-guest complexes. (c, d) Different crystallographic views of "floor" [π···π] stacking interaction between two adjacent host-guest complexes. (e) Solid-state superstructure of **3H-HBC⊂TPACage^{6+}**, revealing how the complexes assemble into a 3D framework with relatively large 1D channels. The hydrogen atoms, counterions and solvent molecules are omitted for the sake of clarity. **TPACage^{6+}**: C skyblue and red, N blue. **3H-HBC** C pink, O red.

(b) Crystal Parameters. [$C_{54}H_{36}O_6$⊂$C_{102}H_{84}N_8$·$(AsF_6)_6$]. *M*r =3334.40. Dark red block (0.15 × 0.12

× 0.11 mm^3). Trigonal, space group $P\bar{3}1c$ (no. 163), a = 25.2556(5) Å, b = 25.556(5) Å, c = 43.6213(10) Å, α = 90.000, β = 90.000, γ = 120.000°, V = 24096(5) Å^3, Z = 3.99996, T = 200(2) K, μ(CuKα) = 1.103 mm^{-1}, $Dcalc$ = 0.815 g cm^{-3}, 46411 reflections measured (8.084° ≤ 2Θ ≤ 98.17°), 7951 unique (R_{int} = 0.0316, R_{sigma} = 0.0207) which were used in all calculations. The final R_1 was 0.1812 ($I > 2\sigma(I)$) and wR_2 was 0.5225 (all data). CCDC Number: 2044797.

(c) Refinement Details. Distance restraints were imposed on the atoms. The enhanced rigid-bond restraint was applied globally. Two of the $[AsF_6]^-$ anions (per formula) were not located, most likely a result of disorder and weak diffraction of the crystal.

(d) Solvent Treatment Details. The solvent masking procedure as implemented in Olex2 was used to remove the electronic contribution of solvent molecules from the refinement. As the exact solvent content is not known, only the atoms used in the refinement model are reported in the formula here. Total solvent accessible volume / cell = 12825.1 Å^3 [53.2%], Total electron count / cell = 5201.3.

(5) 3Me-HBC⊂TPACage•6AsF$_6$

(a) Method. **TPACage•**6AsF$_6$ (2.6 mg, 1.0 µmol) and **3Me-HBC** (0.8 mg, 1.0 µmol) were dissolved in a solution of $MeNO_2$:$CHCl_3$ (4:1, 1 mL). The resulting solution was passed through a 0.45-µm filter before being added to two 1-mL tubes with the volumes of 0.20 and 0.45 mL. The tubes were placed together in one 20-mL vial containing *i*Pr_2O (~3 mL). The vial was sealed with a cap. Slow vapor diffusion of *i*Pr_2O into the 1:1 solution of **3Me-HBC** and **TPACage•**6AsF$_6$ during a period of three days yielded dark red single crystals of **3Me-HBC⊂TPACage•**6AsF$_6$. A suitable crystal was mounted on a MITIGEN holder in Paratone oil on a XtaLAB Synergy R, DW system, HyPix diffractometer. The crystal was kept at 200.01 K during data collection. Using Olex2[3], the structure was solved with the ShelXT[4] structure solution program using intrinsic phasing and refined with the ShelXL[5] refinement package using least squares minimization. The solid-state superstructure of **3Me-HBC⊂TPACage•**6AsF$_6$ is shown in Fig.6 and Supplementary Fig.67.

(b) Crystal Parameters. $[C_{57}H_{42}O_6{\subset}C_{102}H_{84}N_8{\cdot}(AsF_6)_6]$. Mr =3378.19. Dark red block (0.201 × 0.165 × 0.081 mm^3). Trigonal, space group group $P\bar{3}1c$ (no. 163), a = 25.6837(7), b = 25.6837(7), c = 44.7785(8) Å, α = 90.000, β = 90.000, γ = 120.000°, V = 25580.9(15) Å^3, Z = 4, T = 200.01(10) K, μ(CuKα) = 1.427 mm^{-1}, $Dcalc$ = 0.877 g/mm^3, 81404 reflections measured (3.946

≤ 2Θ ≤ 100.868), 8951 unique (R_{int} = 0.0837, R_{sigma} = 0.0284) which were used in all calculations. The final R_1 was 0.1851 ($I > 2\sigma(I)$) and wR_2 was 0.4696 (all data) CCDC Number: 2045291.

(c) Refinement Details. Distance restraints were imposed on the disordered $[AsF_6]^-$ anions. The enhanced rigid-bond restraint was applied globally. Restraints on similar amplitudes separated by less than 1.7 Å.

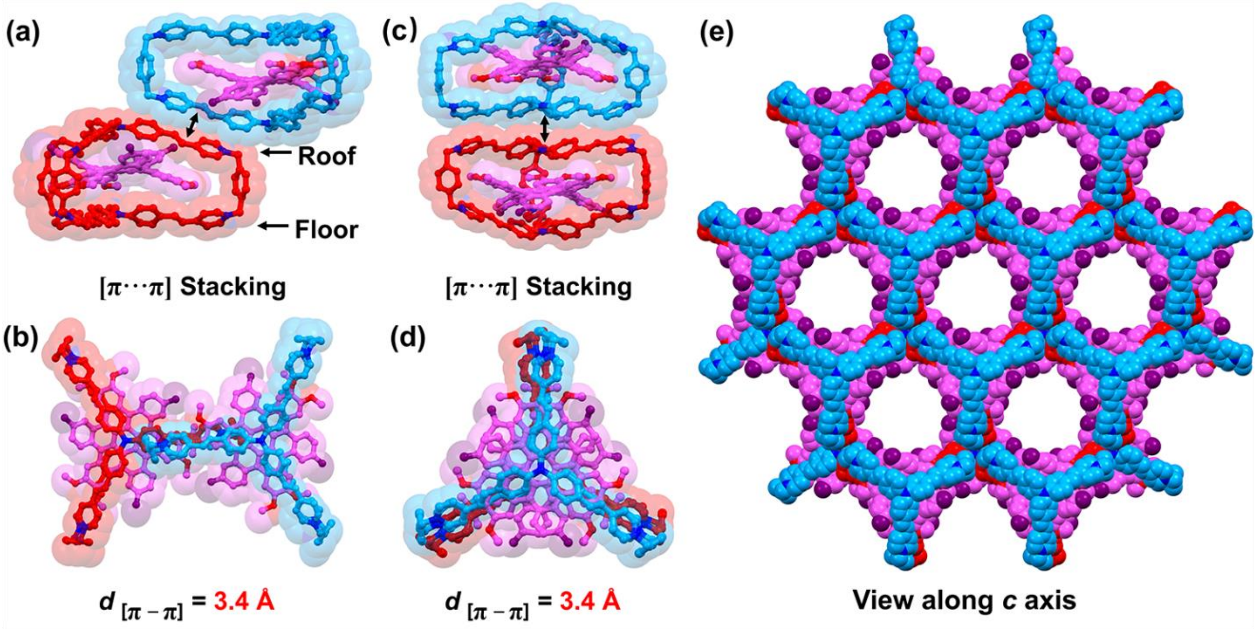

Supplementary Figure 67. Space-filling and ball-and-stick representations of the solid-state superstructure of **3Me-HBC⊂TPACage^{6+}**. (a, b) Different crystallographic views of "roof" [π···π] stacking interaction between two adjacent host-guest complexes. (c, d) Different crystallographic views of "floor" [π···π] stacking interaction between two adjacent host-guest complexes. (e) Solid-state superstructure of **3Me-HBC⊂TPACage^{6+}**, revealing how the complexes assemble into a 3D framework with relatively large 1D channels. The hydrogen atoms, counterions and solvent molecules are omitted for the sake of clarity. **TPACage^{6+}**: C skyblue and red, N blue. **3Me-HBC**: C pink and purple, O red.

(d) Solvent Treatment Details. The solvent masking procedure as implemented in Olex2 was used to remove the electronic contribution of solvent molecules from the refinement. As the exact solvent content is not known, only the atoms used in the refinement model are reported in the formula here. Total solvent accessible volume / cell = 10644.0 Å^3 [41.6%] Total electron count / cell = 3133.5.

Supplementary Note 7. Theoretical Calculation

(1) Volume calculation

The cavity volume of **TPACage^{6+}** was calculated using a 3V channel program[6]. The volume was probed by taking the difference between two rolling-probe solvent-excluded surfaces, one with as large as possible a probe radius and the other with a small solvent radius. The small probe size was set at 1.4 Å while the big probe size was set as 10.0 Å.

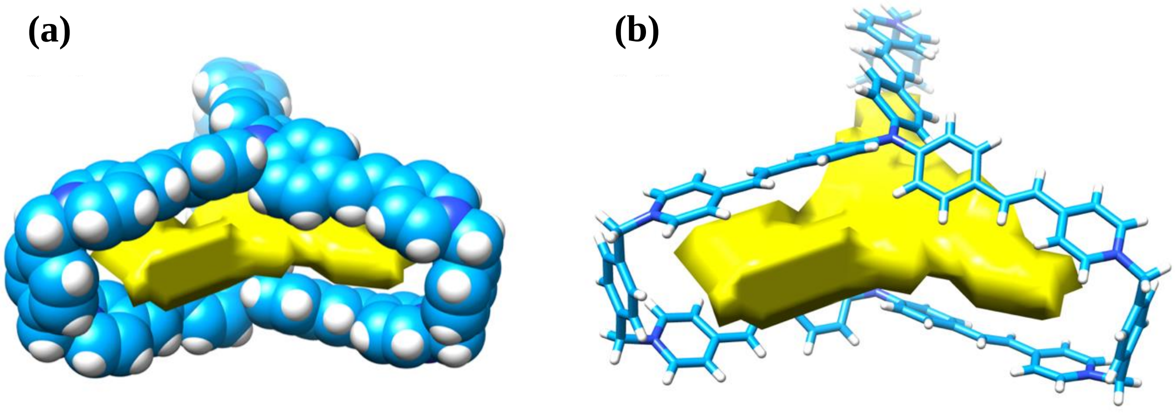

Supplementary Figure 68. Space-filling (a) and capped-stick (b) representation of **TPACage^{6+}** showing the inner volume (yellow filling, 368 Å^3). The volume was calculated by using a virtual rolling probe with a 1.4 Å radius.

(2) Visualization of noncovalent interactions

Independent gradient model (IGM) analysis is an approach[7] based on promolecular density (an electron density model prior to molecule formation) to identify and isolate intermolecular interactions. Strong polar attractions and van der Waals contacts are visualized as an iso-surface with blue and green color, respectively. Single crystal superstructures were used as input files. The binding surface was calculated by Multiwfn 3.6 program[8] through function 20 (visual study of weak interaction) and visualized using Chimera software[9].

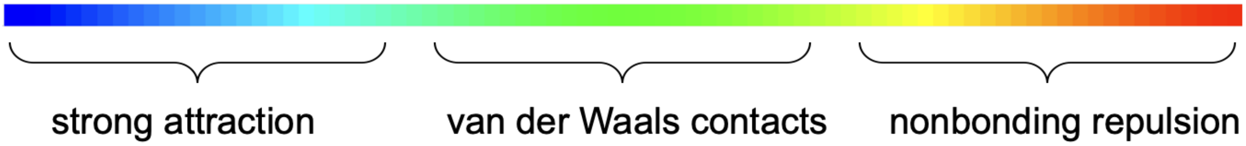

Supplementary Figure 69. Color-coded sign $(\lambda_2)_\rho$ scale bar

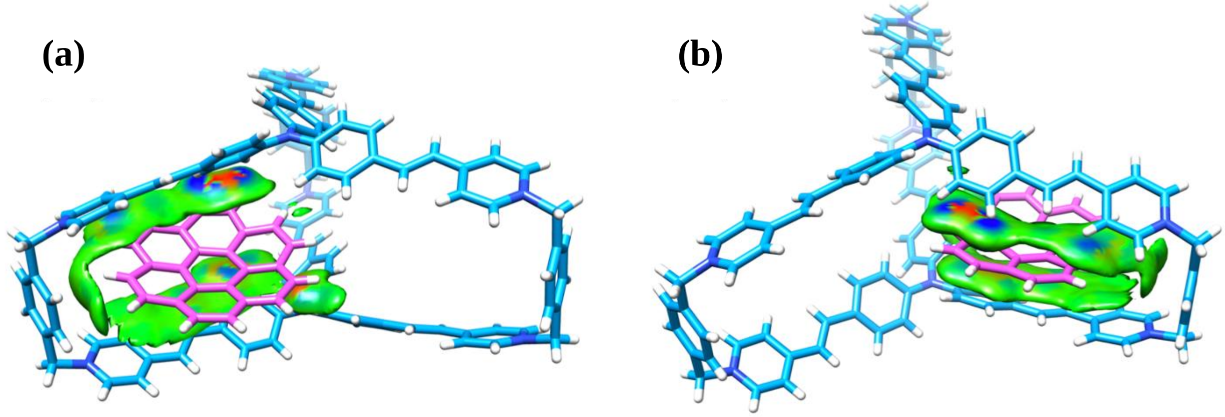

Supplementary Figure 70. Side-on views (a, b) for the capped-stick representations of **COR⊂TPACage^{6+}** complex showing the intermolecular binding iso-surfaces. $\Delta\kappa$ inter (ρ) = 0.003 a.u. Iso-surfaces are colored according to a BGR scheme over the range –0.05 < sign(λ_2)ρ < +0.05 a.u.

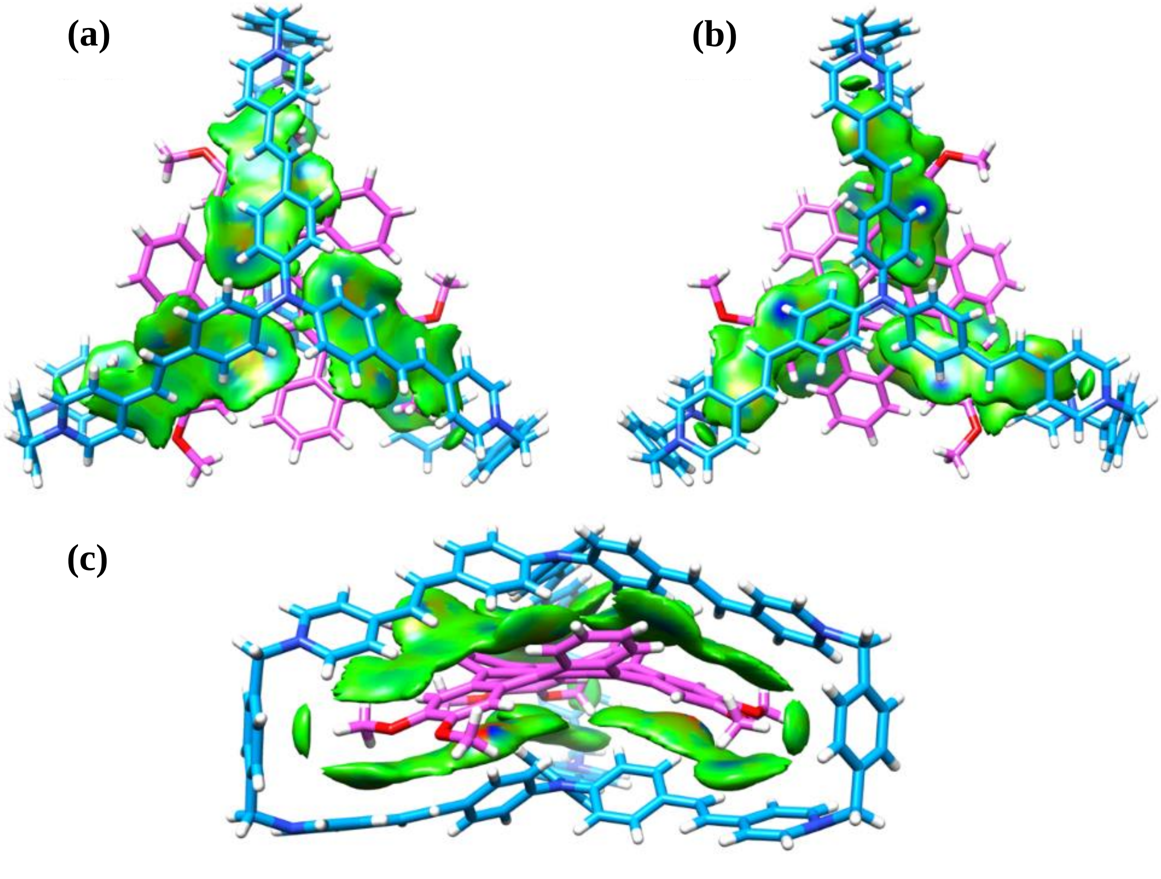

Supplementary Figure 71. Top down (a, b) and side-on views (c) for the capped-stick representations of **3H-HBC⊂TPACage^{6+}** complex showing the intermolecular binding iso-surfaces. $\Delta\kappa$ inter (ρ) = 0.003 a.u. Iso-surfaces are colored according to a BGR scheme over the range –0.05 < sign(λ_2)ρ < +0.05 a.u.

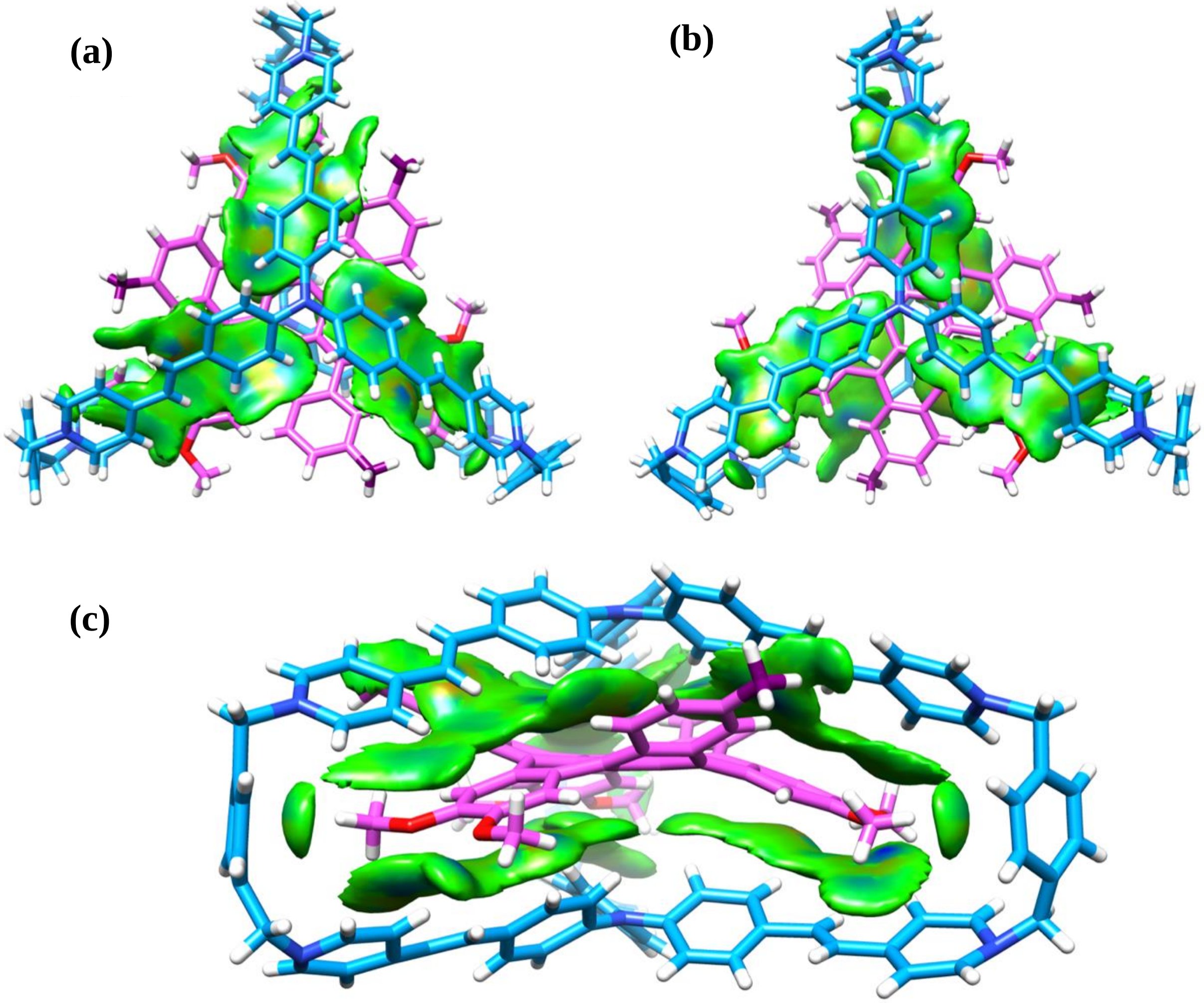

Supplementary Figure 72. Top down (a, b) and side-on views (c) for the capped-stick representations of **3Me-HBC⊂TPACage^{6+}** complex showing the intermolecular binding iso-surfaces. $\Delta\kappa$ inter (ρ) = 0.003 a.u. Iso-surfaces are colored according to a BGR scheme over the range $-0.05 < \mathrm{sign}(\lambda_2)\rho < +0.05$ a.u.

(3) Surface-Area Overlap (SOA) analysis

Surface-area overlap analysis[10] was performed by Chimera[9] and ImageJ software[11]. Single crystal structures of the host-guest complexes were truncated by removing the top half of **TPACage^{6+}** and visualized by Chimera. The guests and **TPACage^{6+}** were colored in order to show the bridging units (yellow), the binding cavity of **TPACage^{6+}** (light blue), the area of guests (pink), and the overlapping portion between the **TPACage^{6+}** and guests (purple). ImageJ 1.53a software was used to measure the percent of SAO in each host-guest complex. Values were calculated for the SAO-

TPACage^{6+} (the overlapping portion between the host and guest divided by the total area of the host) and SAO-guest (the overlapping portion between the host and guest divided by the total area of the guest).

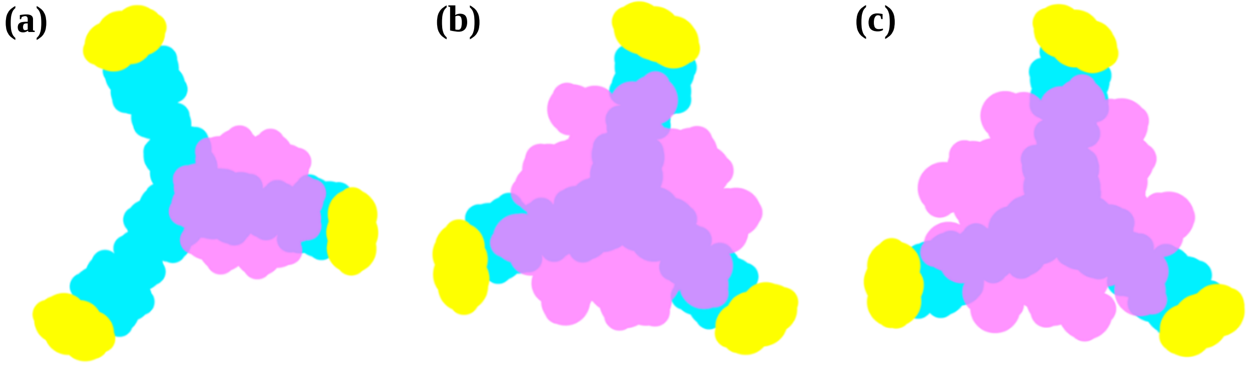

SOA(TPACage^{6+}) = 38% SOA(COR) = 60%

SOA(TPACage^{6+}) = 81% SOA(3H-HBC) = 52%

SOA(TPACage^{6+}) = 82% SOA(3Me-HBC) = 50%

Supplementary Figure 73. Surface-area overlap (SAO) images for (a) **COR⊂TPACage^{6+}**, (b) **3H-HBC⊂TPACage^{6+}**, and (c) **3Me-HBC⊂TPACage^{6+}**.

(4) Frontier molecular orbital calculations

The xyz coordinates from the X-ray single crystals structures were used as the starting geometries for quantum mechanical calculations (QM), specifically density functional theory (DFT). The **TPACage^{6+}** and the guests, e.g. **COR**, **3H-HBC**, **3Me-HBC**, **3F-HBC**, as well as their host-guest complexes were optimized in the Orca program[12] (version 4.1.2) using the hybrid generalized gradient approximation (GGA) Becke three-parameter Lee-Yang-Parr[13] (B3LYP) functional, the Ahlrich's double zeta basis set with a polarization function[14] Def2-SVP, and Grimme's third generation atom-pairwise dispersion correction with Becke Johnson damping[15] (D3BJ). The default integration grid was used throughout. The more advanced algorithm in Orca for direct inversion in iterative subspace (DIIS), KDIIS[16], was applied. KDIIS is a combination of DIIS extrapolation and first order perturbation theory and thus diagonalization free. In order to further speed up the DFT optimizations, the Coulomb integral and numerical chain-of-sphere integration for the HF exchange[17,18] (RIJCOSX) method was applied with the Def2/J auxiliary basis[19,20] (AuxJ). All structures were optimized in vacuum and MeCN continuum, using Truhlar's SMD[21] variant of the Conductor-like polarizable continuum model (CPCM)[22] in Orca. A minimum could not be

found for the **3F-HBC⊂TPACage^{6+}** complex in the MeCN SMD continuum, which instead is described as semi-optimized, but should be close to the minimum. The frontier molecular orbitals (FMOs) were computed using the SMD, these surfaces were visualized in ChemCraft[23] (version b574b) at the isolevel ±0.02. It should be noted that in Orca, the effective-core potentials (ECPs) are applied if the input elements that either includes, or are heavier than Kr, so the C / O / N / H atoms involved in the calculations here are *all-electron*.

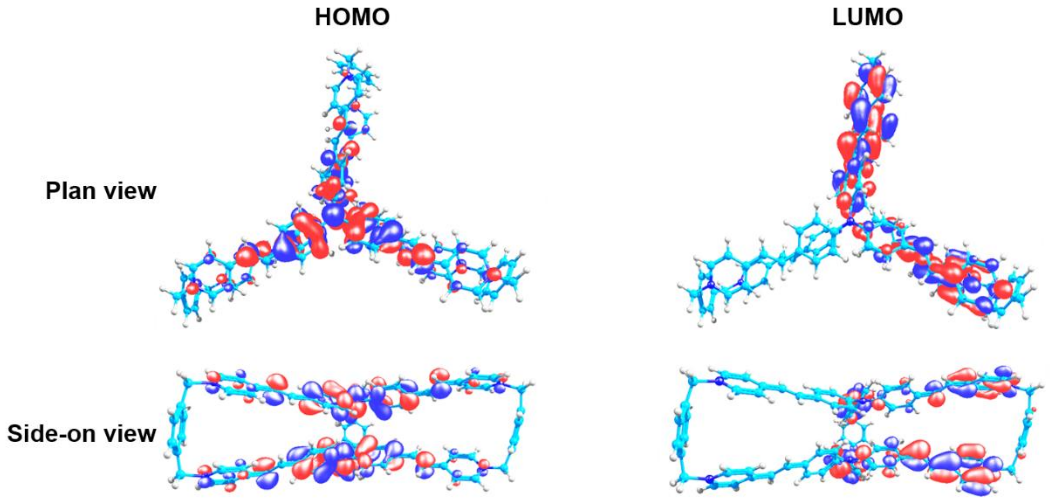

Supplementary Figure 74. Plan and side-on views of frontier molecular orbitals for **TPACage^{6+}** obtained from DFT calculations.

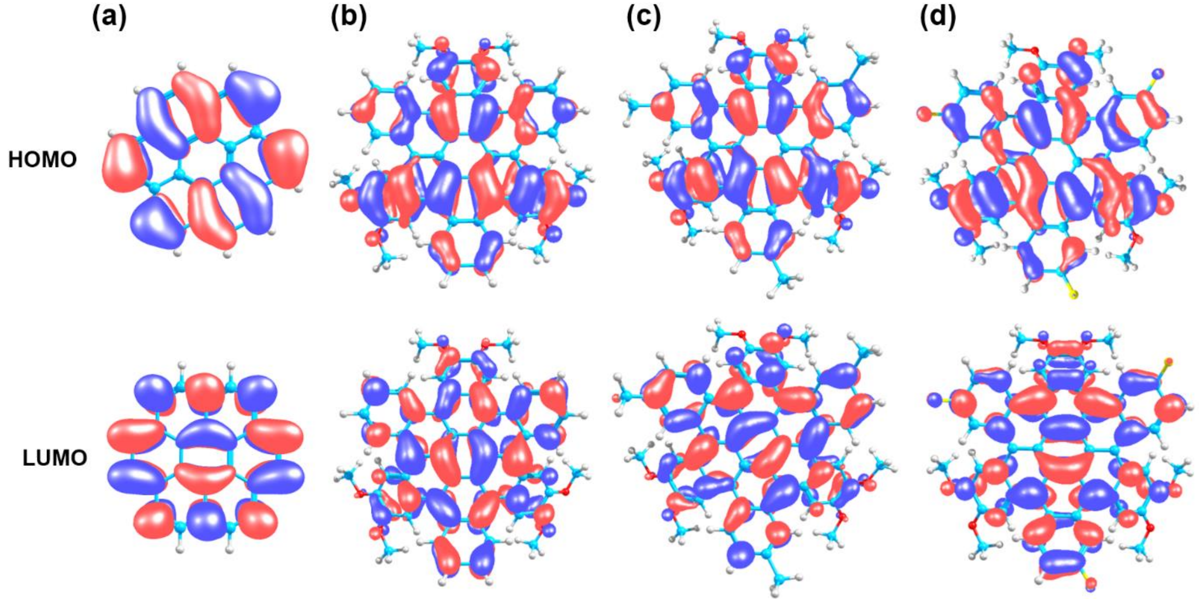

Supplementary Figure 75. Plan views of frontier molecular orbitals for (a) **COR**, (b) **3H-HBC**, (c) **3Me-HBC**, and (d) **3F-HBC** obtained from DFT calculations.

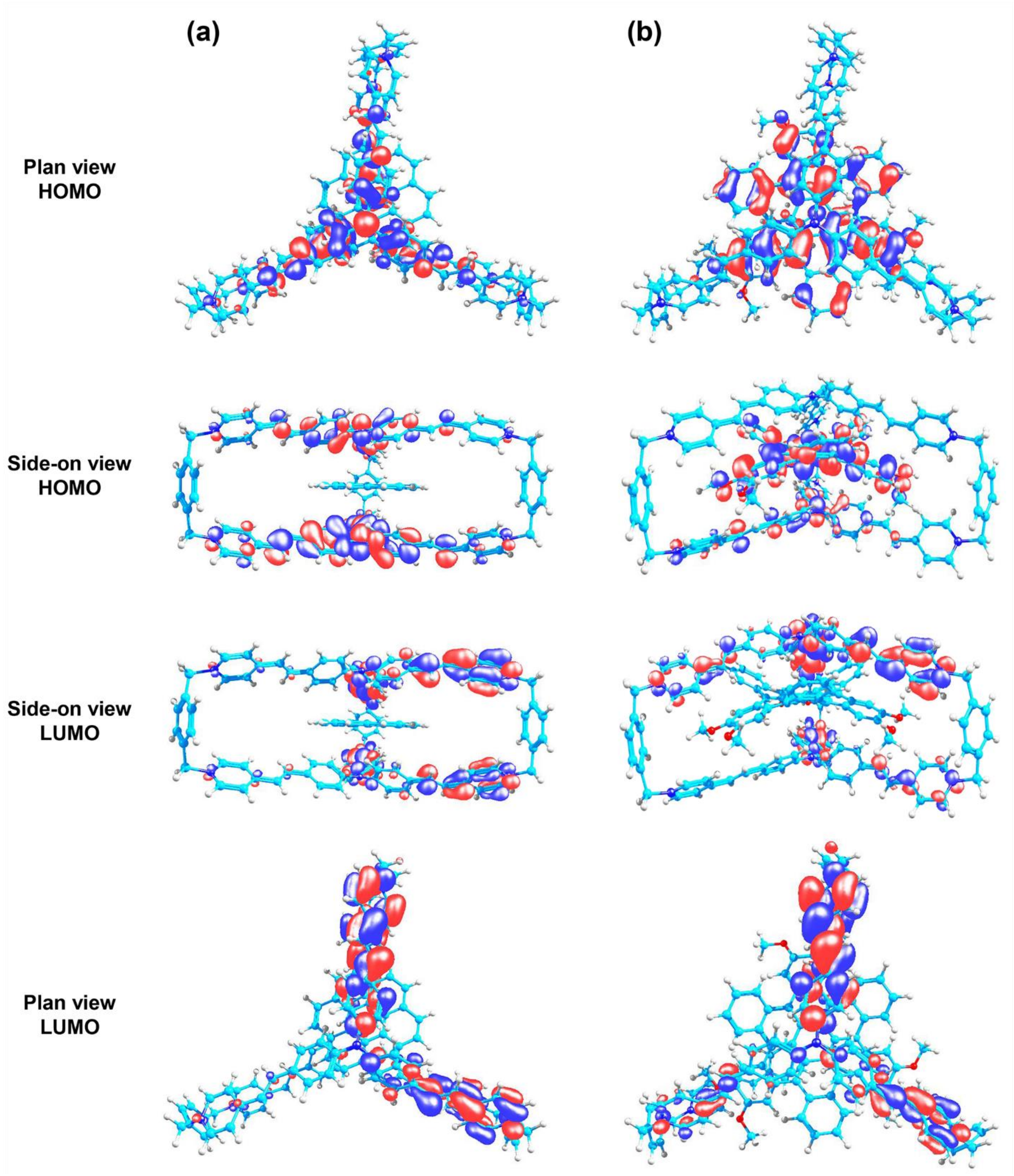

Supplementary Figure 76. Plan and side-on views of frontier molecular orbitals for (a) **COR⊂TPACage^{6+}**, showing that both the HOMO and LUMO of the complex are localized predominantly on **TPACage^{6+}**; (b) **3H-HBC⊂TPACage^{6+}**, showing that the HOMO of the complex is localized predominantly on **3H-HBC**, while the LUMO is localized mainly on **TPACage^{6+}**.

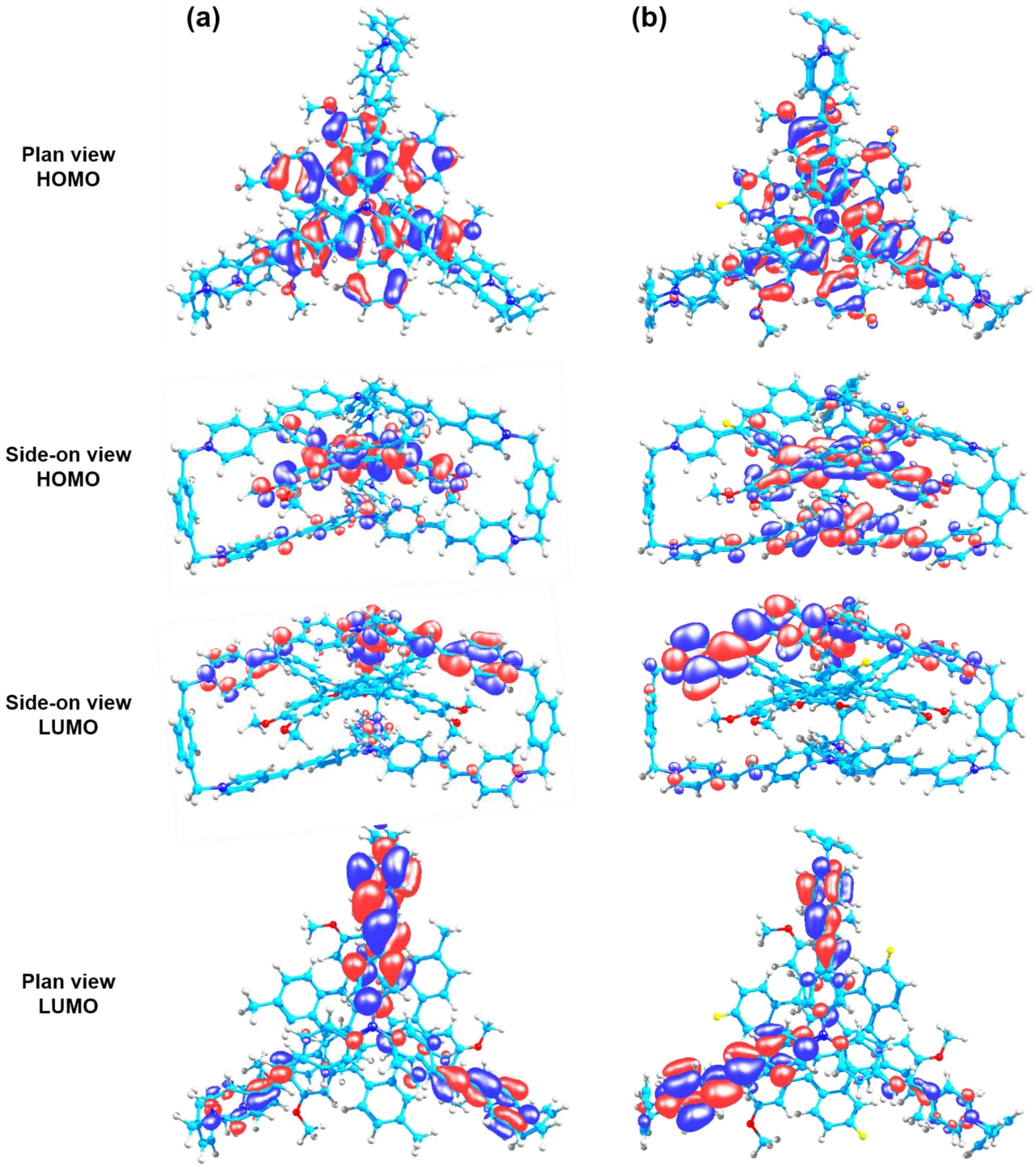

Supplementary Figure 77. Plan and side-on views of frontier molecular orbitals for (a) **3Me-HBC⊂TPACage^{6+}** and (b) **3F-HBC⊂TPACage^{6+}** complexes obtained from DFT calculations. These results show that the HOMOs are localized predominantly on guests, while the LUMOs are localized mainly on **TPACage^{6+}**.

**Supplementary Table 2 The calculated energy levels of the frontier molecular orbitals in MeCN**			
**Entry**	**Ψ_H / eV**	**Ψ_L / eV**	**$\Delta E_{H\text{-}L}$ / eV**
**$TPACage^{6+}$**	–5.56	–3.08	2.48
**COR**	–5.66	–1.59	4.07
**3H-HBC**	–5.06	–1.84	3.22
**3Me-HBC**	–5.01	–1.81	3.20
**3F-HBC**	–5.09	–1.90	3.19
**$COR \subset TPACage^{6+}$**	–5.47	–3.03	2.44
**$3H\text{-}HBC \subset TPACage^{6+}$**	–5.35	–3.05	2.30
**$3Me\text{-}HBC \subset TPACage^{6+}$**	–5.31	–3.07	2.24
**$3F\text{-}HBC \subset TPACage^{6+}$**	–5.41	–3.09	2.32

**Supplementary Table 3 The calculated energy levels of the frontier molecular orbitals in vacuum**			
**Entry**	**Ψ_H / eV**	**Ψ_L / eV**	**$\Delta E_{H\text{-}L}$ / eV**
**$TPACage^{6+}$**	–14.07	–11.77	2.30
**COR**	–5.66	–1.59	4.07
**3H-HBC**	–4.89	–1.65	3.24
**3Me-HBC**	–4.80	–1.58	3.22
**3F-HBC**	–5.04	–1.82	3.22
**$COR \subset TPACage^{6+}$**	–14.13	–11.91	2.22
**$3H\text{-}HBC \subset TPACage^{6+}$**	–13.93	–11.76	2.17
**$3Me\text{-}HBC \subset TPACage^{6+}$**	–13.84	–11.74	2.10
**$3F\text{-}HBC \subset TPACage^{6+}$**	–14.00	–11.79	2.21

(5) Electrostatic potential map calculations

The electrostatic potential maps were computed with B3LYP, the Slater-type basis set[24] DZP and D3BJ in the Amsterdam Density Functional program[25] (ADF, version 2018.104), using Truhlar's solvent model[26] SM12 as implemented in ADF[27]. The Becke grid quality was set to v*ery good*[28] and large frozen cores were used (which for C, O and N relates to the 1s core being frozen) to reduce the computational cost in ADF.

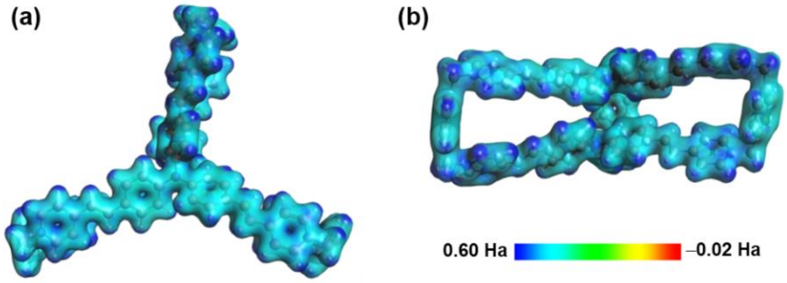

Supplementary Figure 78. Plan and side-on views of electrostatic potential maps for **TPACage^{6+}** obtained from DFT calculations. These maps demonstrate that the **TPACage^{6+}** is relatively electron-deficient.

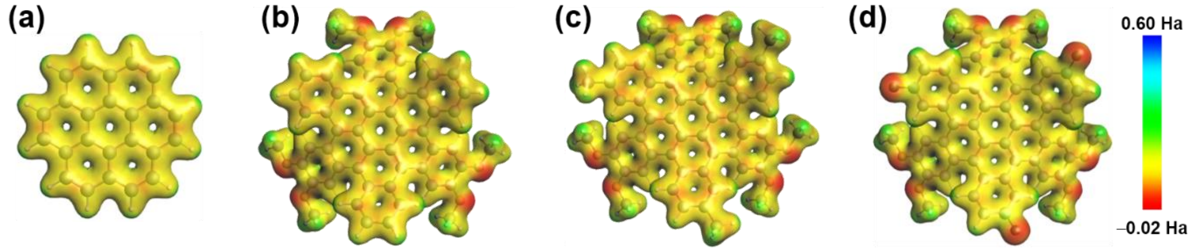

Supplementary Figure 79. Plan views of electrostatic potential maps for (a) **COR**, (b) **3H-HBC**, (c) **3Me-HBC**, and (d) **3F-HBC** obtained from DFT calculations. These maps demonstrate that the ***c*-HBC** guests are relatively electron-rich, and the 1,2-dimethoxybenzene groups are more electron-rich than outer benzene rings in ***c*-HBC** guests.

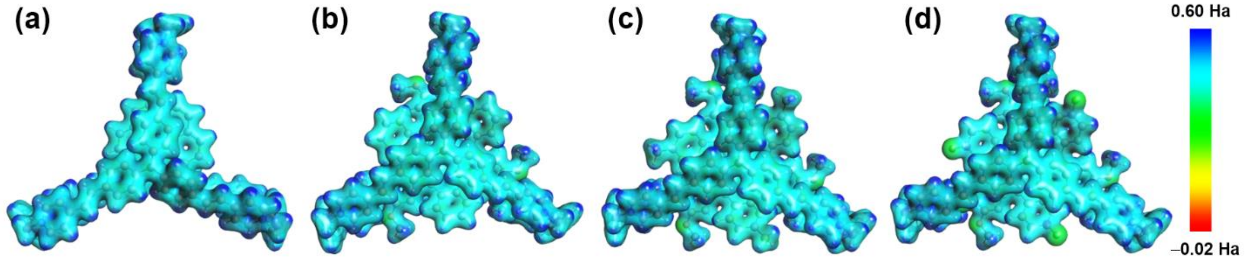

Supplementary Figure 80. Plan views of electrostatic potential maps for (a) **COR⊂TPACage^{6+}**, (b) **3H-HBC⊂TPACage^{6+}**, (c) **3Me-HBC⊂TPACage^{6+}** and (d) **3F-HBC⊂TPACage^{6+}** obtained from DFT calculations. These maps reveals that electron in the original electron-rich guests delocalized in the whole host-guest complexes as a result of intermolecular electron delocalization.

(6) Strain energy calculations

The strain energies were computed as the difference between the single point energies of the guest molecules taken from the complexes and their optimized energies. All of which were only performed in vacuum because the surface area (top and bottom) of each guest taken from the complexes would now be accessible to the solvent continuum if this was added, where it was shielded before inside the complex, and thus add artificial stabilization.

**Supplementary Table 4 The calculated strain energy for *c*-HBC guests after forming the host-guest complexes in vacuum**			
**Entry**	**E_{DFT} / Hartree Free guests**	**E_{DFT} / Hartree Guests in complexes**	**ΔE_{DFT} / kcal mol^{-1}**
**COR⊂TPACage^{6+}**	–920.77047	–920.77078	–0.21
**3H-HBC⊂TPACage^{6+}**	–2527.76499	–2527.76171	2.05
**3Me-HBC⊂TPACage^{6+}**	–2645.56056	–2645.55697	2.25
**3F-HBC⊂TPACage^{6+}**	–2825.15201	–2825.14808	2.47

(7) Binding energy calculations

The binding energies for the formation of host-guest complexes were computed as $\Delta E = E_{HG} - E_{H} - E_{G}$, where E_{HG}, E_{H}, and E_{G} are the electronic energies of optimized structures for the host-guest complexes, host and guest molecules, respectively.

**Supplementary Table 5 The binding energy for the formation of the four host-guest complexes**		
**Entry**	**ΔE_{DFT} / kcal mol^{-1} in MeCN**	**ΔE_{DFT} / kcal mol^{-1} in Vaccum**
**COR⊂TPACage^{6+}**	–30.08	–41.20
**3H-HBC⊂TPACage^{6+}**	–77.57	–114.46
**3Me-HBC⊂TPACage^{6+}**	–79.82	–120.25
**3F-HBC⊂TPACage^{6+}**	–78.51	–112.92

Supplementary Note 8. Photostability Study

(1) The difference in photostability between *c*-HBC and their host-guest complexes

Experiment methods: Two rectangular quartz cells, containing ***c*-HBC** ([***c*-HBC**] = 0.5 mM) and ***c*-HBC** with 2 equiv of **TPACage^{6+}** ([***c*-HBC**] = 0.5 mM, [**TPACage^{6+}**] = 1 mM), respectively, were exposed simultaneously to UV light (370 nm) in MeCN / $CHCl_3$ (4:1) solution. The UV-Vis absorption spectra were recorded by diluting the irradiated solution to 10 μM in a 10 × 2 × 45 mm quartz cell every 12 min. The emission spectra were recorded by diluting the irradiated solution to 0.4 μM in a 10 × 10 × 45 mm quartz cell every 12 min.

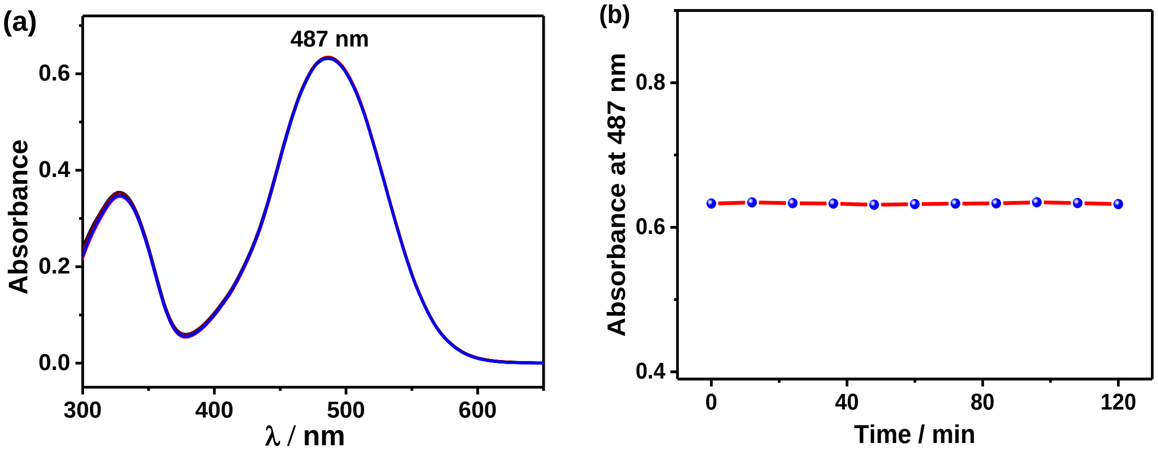

Supplementary Figure 81. (a) UV-Vis Spectroscopic changes of **TPACage•**6PF_6 upon irradiation with UV light (370 nm, 120 min). (b) The changes of absorbance at 487 nm of **TPACage•**6PF_6 upon irradiation with UV light (370 nm, 120 min).

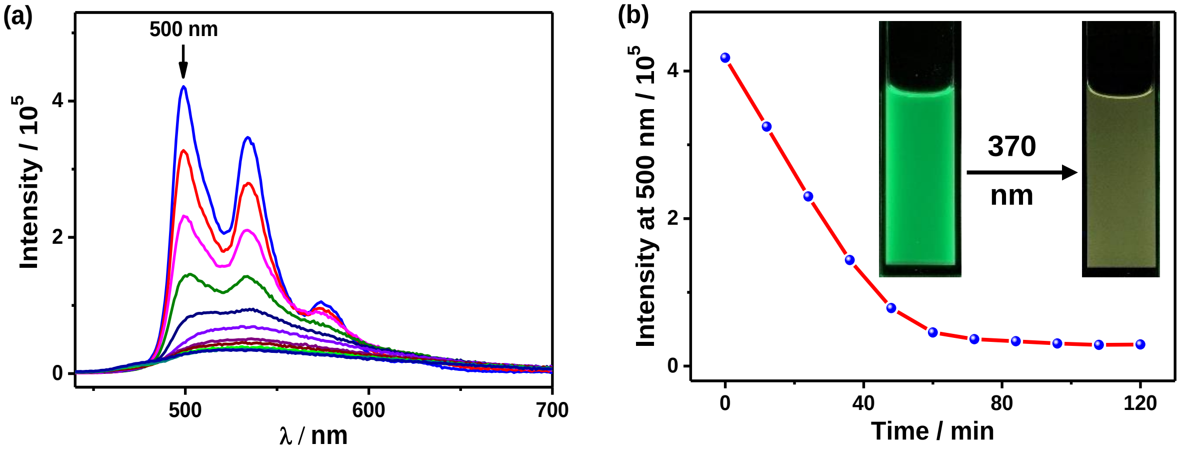

Supplementary Figure 82. (a) Emission spectroscopic (λ_{ex} = 384 nm) changes of **3H-HBC** upon irradiation with UV light (370 nm, 120 min). (b) Fluorescent intensity and fluorescent photographs (*inset*) changes of **3H-HBC** upon irradiation with UV light (370 nm, 120 min).

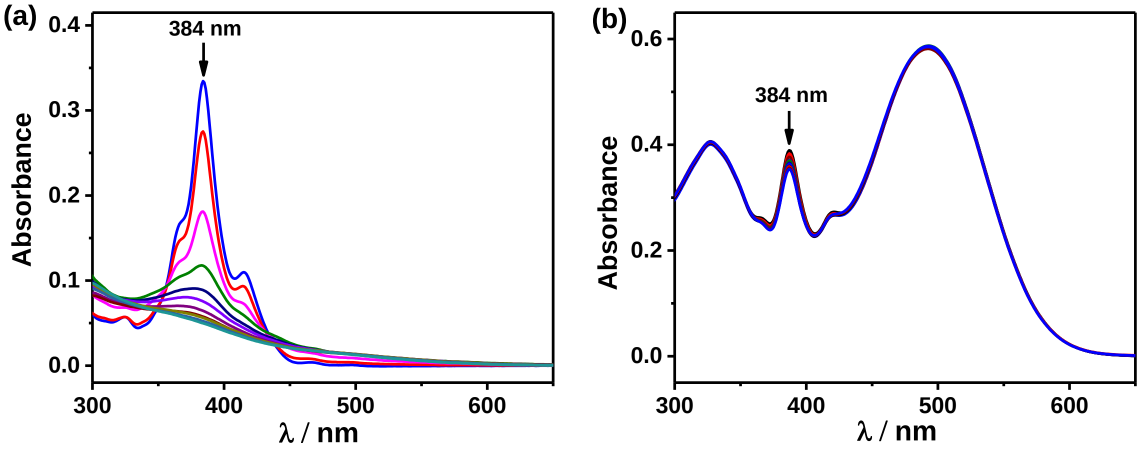

Supplementary Figure 83. UV−Vis Absorption spectroscopic changes of (a) **3Me-HBC**, and (b) **3Me-HBC⊂TPACage•**6PF_6 upon irradiation with UV light (370 nm, 120 min).

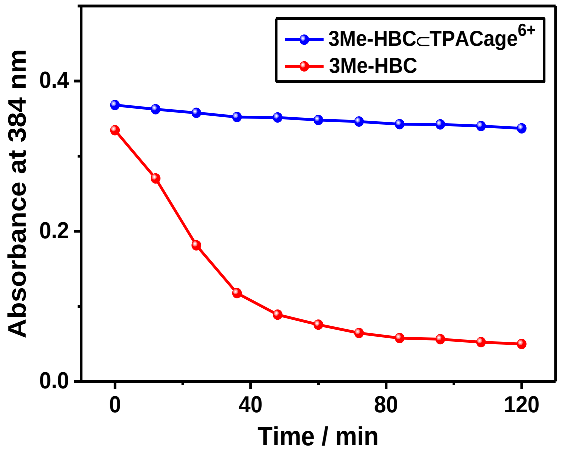

Supplementary Figure 84. Absorbance changes at 384 nm of (a) **3Me-HBC** (red), and (b) **3Me-HBC⊂TPACage•**6PF$_6$ (blue) upon irradiation with UV light (370 nm, 120 min).

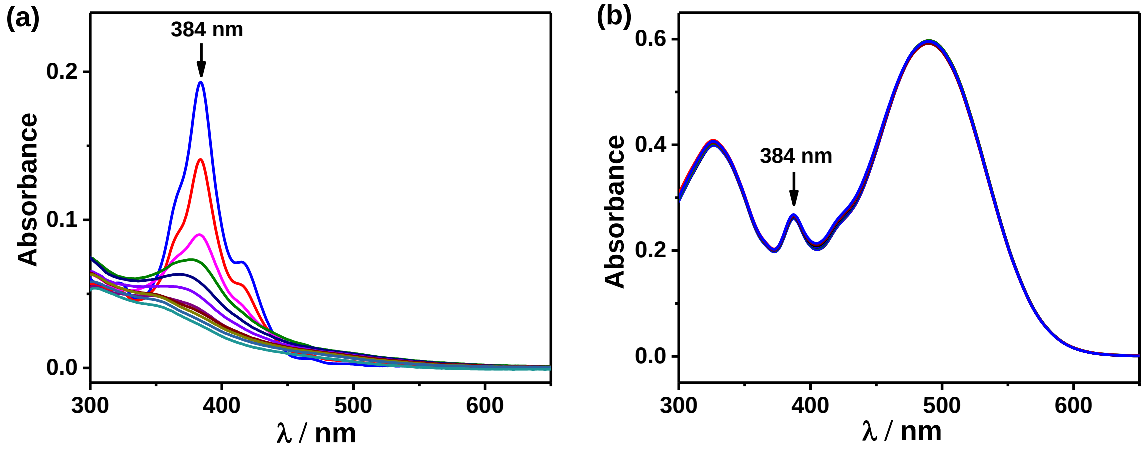

Supplementary Figure 85. UV−Vis Absorption spectroscopic changes of (a) **3F-HBC**, and (b) **3F-HBC⊂TPACage•**6PF$_6$ (b) upon irradiation with UV light (370 nm, 120 min).

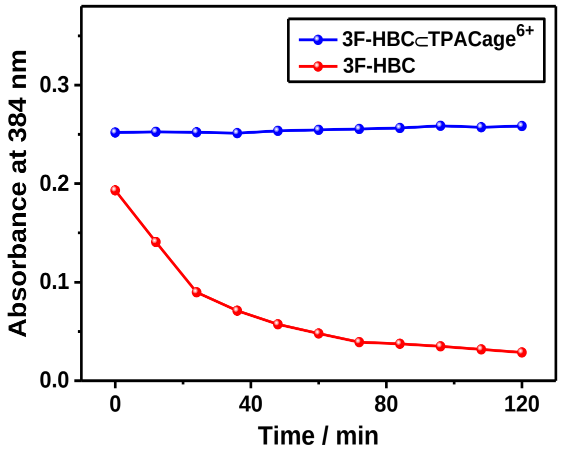

Supplementary Figure 86. Absorbance changes at 384 nm of (a) **3F-HBC** (red) and (b) **3F-HBC⊂TPACage•**6PF$_6$ (blue) upon irradiation with UV light (370 nm, 120 min).

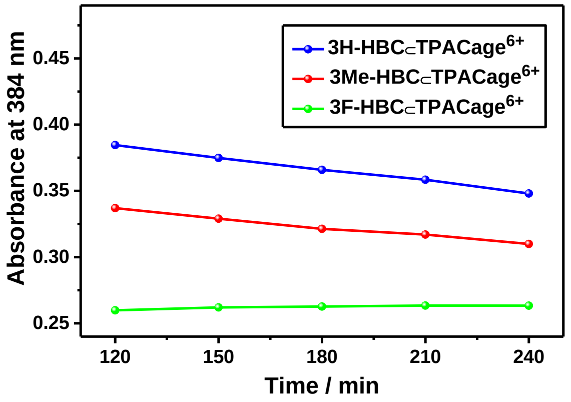

Supplementary Figure 87. Absorbance changes at 384 nm of **3H-HBC⊂TPACage**•6PF_6 (blue), **3Me-HBC⊂TPACage**•6PF_6 (red) and **3F-HBC⊂TPACage**•6PF_6 (green) upon irradiation with UV light（370 nm）up to 240 min.

(2) The difference in photostability between three individual *c*-HBC guests

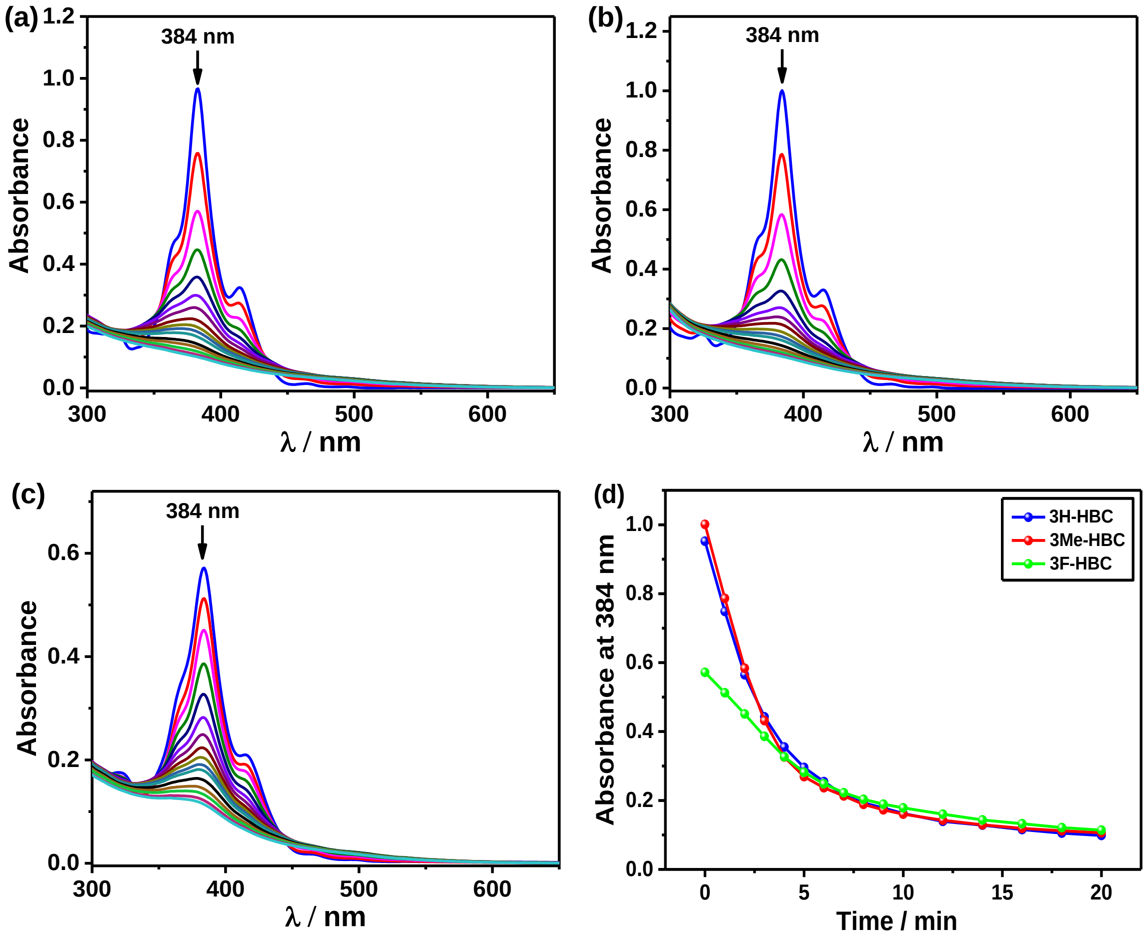

Supplementary Figure 88. UV−Vis Absorption spectroscopic (1.5 × 10^{-5} M, MeCN:$CHCl_3$ = 4:1, 298 K, optical path: 4 mm) changes of (a) **3H-HBC**, (b) **3Me-HBC**, and (c) **3F-HBC** when exposed simultaneously to UV light (370 nm, 20 min). (d) Absorbance changes at 384 nm of **3H-HBC** (blue), **3Me-HBC** (red) and **3F-HBC** (green) upon irradiation with UV light.

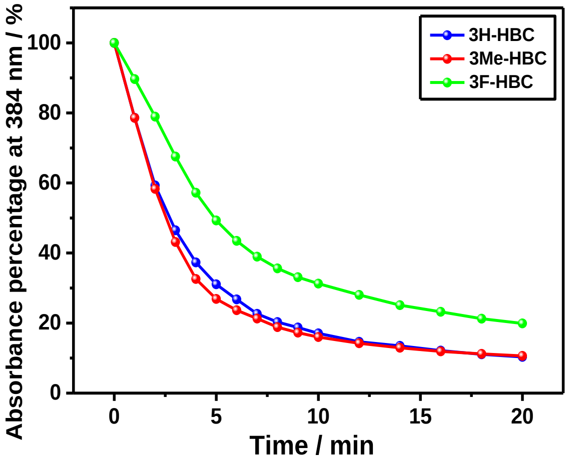

Supplementary Figure 89. Absorbance percentage at 384 nm of **3H-HBC** (blue), **3Me-HBC** (red) and **3F-HBC** (green) upon irradiation with UV light (370 nm, 20 min).

(3) Femtosecond transient absorption (FsTA) measurements

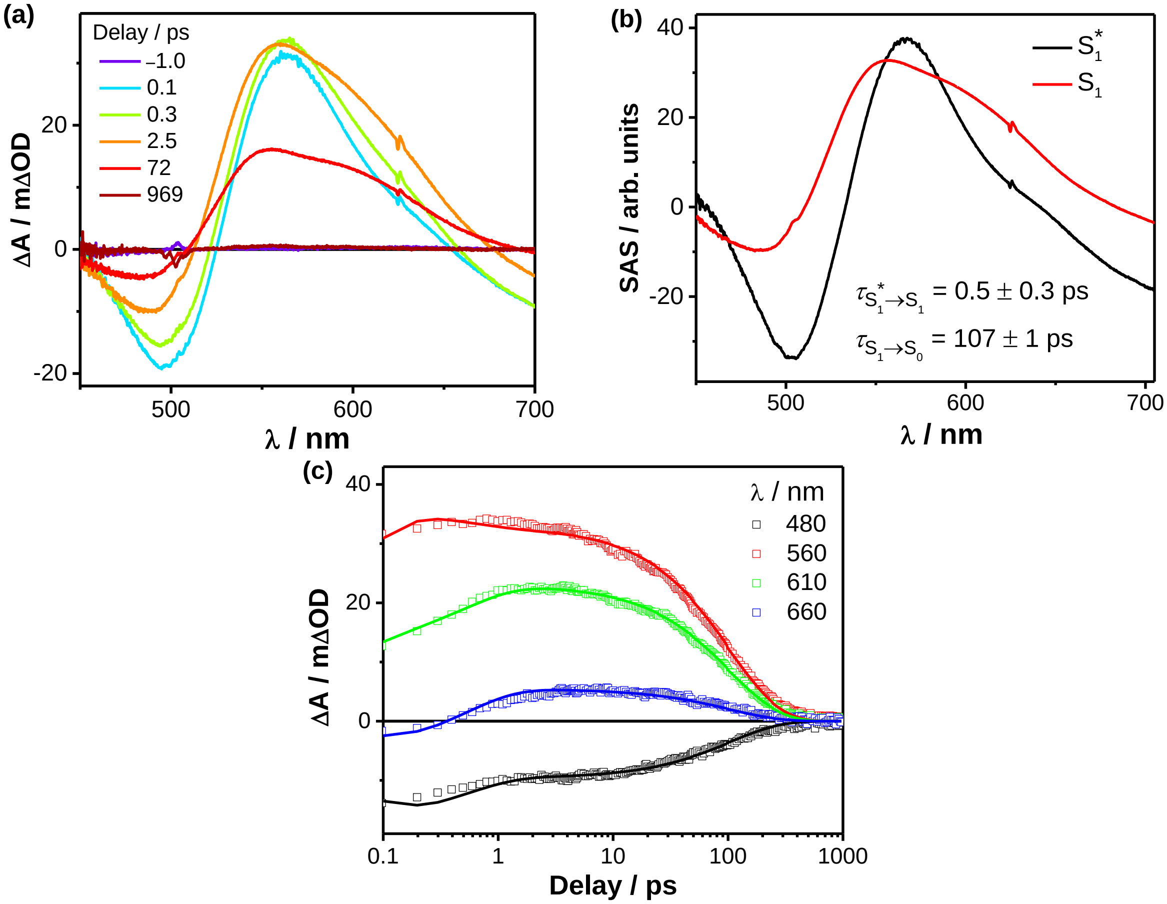

Supplementary Figure 90. FsTA Spectra of **TPACage**$^{6+}$ excited at 500 nm in a solution of MeCN / $CHCl_3$ (4:1). (a) FsTA Spectra at selected delay times, (b) species associated spectra, and (c) fits to kinetic model $S^*_1 \rightarrow S_1 \rightarrow S_0$ at selected wavelengths obtained using global analysis. S^*_1, S_1, and S_0 represent the vibrationally-hot first singlet excited state, first singlet excited state, and ground state of **TPACage**$^{6+}$, respectively.

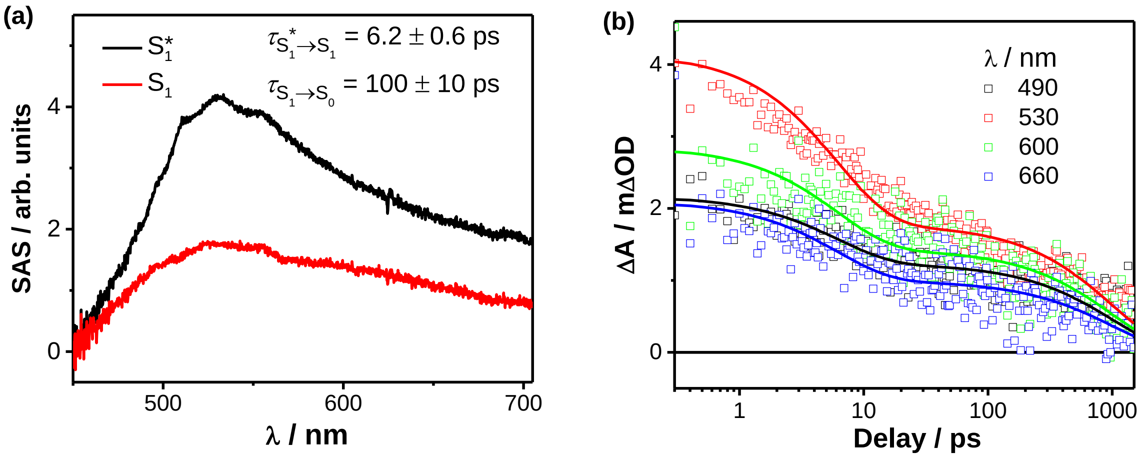

Supplementary Figure 91. FsTA Spectra of **3H-HBC** excited at 350 nm in a solution of MeCN / $CHCl_3$ (4:1). (a) Species associated spectra and (b) fits to kinetic model $S^*_1 \rightarrow S_1 \rightarrow S_0$ at selected wavelengths obtained using global analysis. S^*_1, S_1, and S_0 represent the vibrationally-hot first singlet excited state, first singlet excited state, and ground state of **3H-HBC**, respectively.

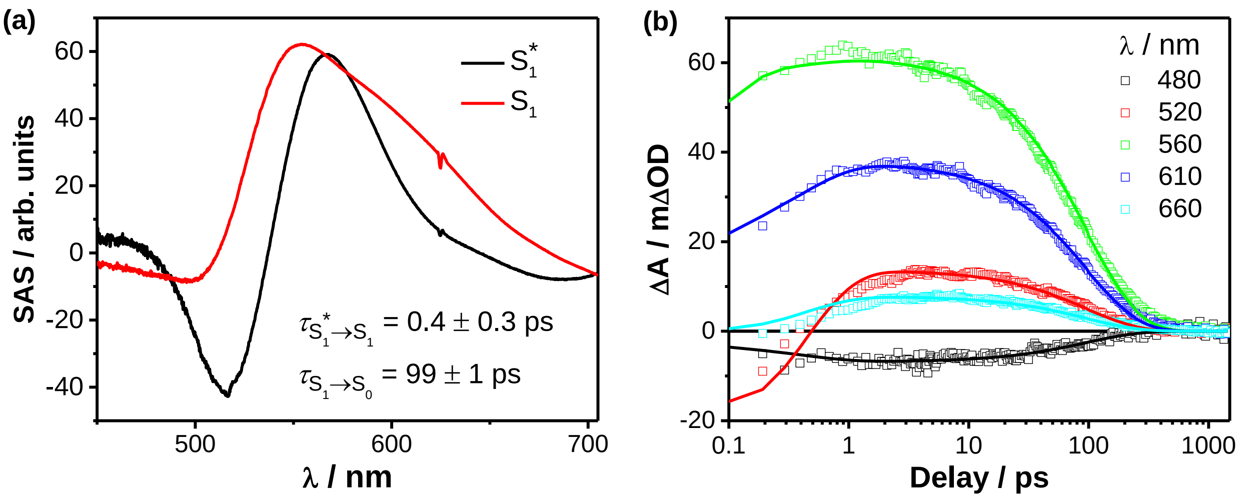

Supplementary Figure 92. FsTA Spectra of **3H-HBC⊂TPACage**$^{6+}$ complex excited at 350 nm in a solution of MeCN / $CHCl_3$ (4:1). (a) Species associated spectra and (b) fits to kinetic model $S^*_1 \rightarrow S_1 \rightarrow S_0$ at selected wavelengths obtained using global analysis. S^*_1, S_1, and S_0 represent the vibrationally-hot first singlet excited state, first singlet excited state, and ground state of **3H-HBC⊂TPACage**$^{6+}$ complex, respectively.

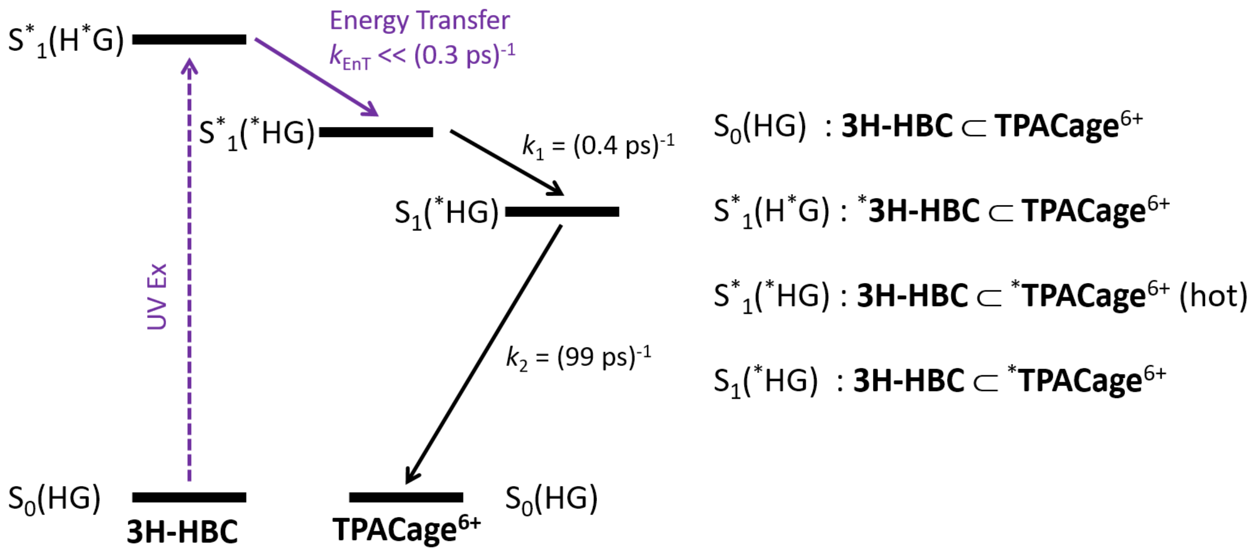

Supplementary Figure 93. A possible Jablonski diagram for the **3H-HBC⊂TPACage**$^{6+}$ complex assuming excitation of the **3H-HBC** guest with the 350 nm pump pulse. H and G represent host and guest, respectively.

Supplementary References

1. Zhang, Q., *et al.* Facile bottom-up synthesis of coronene-based 3-fold symmetrical and highly substituted nanographenes from simple aromatics. *J. Am. Chem. Soc.* **136**, 5057−5064 (2014).

2. Peck, E. M., *et al.* Rapid macrocycle threading by a fluorescent dye-polymer conjugate in water with nanomolar affinity. *J. Am. Chem. Soc.* **137**, 8668–8671 (2015).

3. Dolomanov, O. V., *et al.* OLEX2: A complete structure solution, refinement and analysis program. *J. Appl. Cryst.* **42**, 339−341 (2009).

4. Sheldrick, G. M. SHELXT—Integrated space-group and crystal-structure determination. *Acta. Cryst*. **A71**, 3−8 (2015).

5. Sheldrick, G. M. A short history of SHELX. *Acta. Cryst*. **A64**, 112−122 (2008).

6. Voss, N. R., Gerstein, M. 3V: Cavity, channel and cleft volume calculator and extractor. *Nucleic Acids Res.* **38**, 555–562 (2010).

7. Lefebvre, C., *et al.* Accurately extracting the signature of intermolecular interactions present in the NCl plot of the reduced density gradient versus electron density. *Phys. Chem. Chem. Phys.* **19**, 17928–17936 (2017).

8. Lu, T., Chen, F. Multiwfn: A multifunctional wavefunction analyzer. *J. Comput. Chem.* **33**, 580–592 (2012).

9. Pettersen, E. F., *et al.* UCSF Chimera−A visualization system for exploratory research and

analysis. *J. Comput. Chem.* **25**, 1605–1612 (2004).
10. Dale, E. J., *et al.* ExCage. *J. Am. Chem. Soc.* **136**, 10669–10682 (2014).
11. Schneider, C. A., Rasband, W. S., Eliceiri, K. W. NIH Image to ImageJ: 25 years of image analysis. *Nat. Methods* **9**, 671–675 (2012).
12. Neese, F. The ORCA program system. *Wiley Interdiscip. Rev.*: *Comput. Mol. Sci*. **2**, 73−78 (2012).
13. Becke, A. D. Density functional thermochemistry. III. The role of exact exchange. *J. Chem. Phys*. **98**, 5648−5652 (1993).
14. Weigend, F., Ahlrichs, R. Balanced basis sets of split valence, triple zeta valence and quadruple zeta valence quality for H to Rn: Design and assessment of accuracy. *Phys. Chem. Chem. Phys*. **7**, 3297−3305 (2005).
15. Grimme, S., Ehrlich, S., Goerigk, L. Effect of the damping function in dispersion corrected density functional theory. *J. Comput. Chem*. **32**, 1456–1465 (2011).
16. Kollmar, C. The role of energy denominators in self-consistent field (SCF) calculations for open shell systems. *J. Chem. Phys.* **105**, 8204–8212 (1996).
17. Neese, F. An improvement of the resolution of the identity approximation for the calculation of the Coulomb matrix. *J. Comp. Chem*., **24**, 1740−1747 (2003).
18. Izsák, R., Neese, F. An overlap fitted chain of spheres exchange method, *J. Chem. Phys*. **135**, 144105 (2011).
19. Weigend, F. Accurate Coulomb-fitting basis sets for H to Rn. *Phys. Chem. Chem. Phys.* **8**, 1057−1065 (2006).
20. Stoychev, G. L., Auer, A. A., Neese, F. Automatic generation of auxiliary basis sets. *J. Theo. Comp. Chem*. **13**, 554−562 (2017).
21. Marenich, A. V., Cramer, C. J., Truhlar, D. G. Universal solvation model based on solute electron density and on a continuum model of the solvent defined by the bulk dielectric constant and atomic surface tensions. *J. Phys. Chem. B,* **113**, 6378−6396 (2009).
22. Pascual-Ahuir, J. L., Silla, E. GEPOL: An improved description of molecular surfaces. I. Building the spherical surface set. *J. Comput. Chem*. **11**, 1047–1060 (1990).
23. https://www.chemcraftprog.com.
24. Van Lenthe, E., Baerends, E. J. Optimized slater-type basis sets for the elements 1−118. *J. Comput. Chem*. **24**, 1142−1156 (2003).
25. te Velde, G., *et al.* Chemistry with ADF. *J. Comput. Chem*. **22**, 931−967 (2001).

26. Marenich, A.V., Cramer, C. J., Truhlar, D. G. Generalized born solvation model SM12. *J. Chem. Theory Comput.* **9**, 609–620 (2013).
27. Peeples, C. A., Schreckenbach, G. Implementation of the SM12 solvation model into ADF and comparison with COSMO, *J. Chem. Theory Comput.* **12**, 4033–4041 (2016).
28. Franchini, M., Philipsen, P. H. T., Visscher, L. The Becke fuzzy cells integration scheme in the Amsterdam density functional program suite, *J. Comput. Chem*. **34**, 1818–1827 (2013).
