# Supplementary material for: A contorted nanographene shelter
Source: Nat Commun. 2021 Aug 31;12:5191. doi: 10.1038/s41467-021-25255-6 (PMC8408160; doi:10.1038/s41467-021-25255-6)
Supplement: Supplementary file 2 — Description of Additional Supplementary Files [file 41467_2021_25255_MOESM2_ESM.pdf]

Description of Additional Supplementary Files

File Name: Supplementary Data 1

Description: the folder includes all the cif. files of single-crystal superstructures for **TPACage•6Cl, 3H-HBC, COR⊂TPACage•6Cl, 3HHBC⊂TPACage•6AsF$_6$ and 3Me-HBC⊂TPACage•6AsF$_6$**, and the Cartesian coordinates for optimized structures.
