# Supplementary material for: A contorted nanographene shelter
Source: Nat Commun. 2021 Aug 31;12:5191. doi: 10.1038/s41467-021-25255-6 (PMC8408160; doi:10.1038/s41467-021-25255-6)
Supplement: Supplementary file 3 — Supplementary Data 1 [file 41467_2021_25255_MOESM3_ESM.zip › Supplementary Data Set/Cartesian Coordinates.pdf]

Cartesian Coordinates for Optimized Structures

(1) Optimized In Vacuum

(a) TPACage^{6+}

H	13.38108688873088	9.59659269105763	9.69895045643073
H	14.20999774153892	5.77768865094881	9.98391044464356
H	10.16686442660228	6.91306668475011	9.73716829944372
H	15.51747268213260	10.75422343241634	10.09144252652358
H	14.25535586491639	3.33611748173767	10.24336476770060
H	8.01705680347692	8.06721961361238	9.98720240399777
C	14.05025485487233	9.24552734679562	10.48700132385516
C	13.42621674461924	5.28182836530254	10.55897666521215
C	10.09731143699072	7.78644281972111	10.38689633776664
C	15.25766097618432	9.89708327055579	10.71622369512959
C	13.44893278458241	3.90272115221031	10.71256211451896
C	8.88446239939119	8.44391221474005	10.53259067110411
H	17.51415951780026	11.73246302819936	10.70619044587793
H	14.37586077752658	1.23910523767237	11.05018507309292
H	6.09513799504306	9.05765195650778	10.62022298794846
H	19.16248032120292	13.54603091095653	10.77996340605861
H	15.51683449688848	-0.84257841496275	11.49452861315193
H	3.68229756629467	9.22734139315860	10.70270079077634
N	12.43890509350426	7.46886724404224	11.05606791254259
C	13.68393095767848	8.12445293397249	11.25979612166411
C	12.39795483685648	6.05589560715845	11.14246183512696
C	11.24253259260136	8.22186228053706	11.09102637608432
C	18.01075979602975	11.17454456350827	11.50435596467758
C	13.44069970807645	0.90790046884429	11.50935976458003
C	6.25207391389071	9.90908626656124	11.28803787711567
C	19.73696666908165	12.97502152103636	11.51198337169752
C	14.59299134183052	-1.25663250766705	11.90195886806516
C	3.77374611708082	10.05782535454897	11.40508990906289
C	16.14519488607576	9.45377043227133	11.72596122735048
C	12.43797048420029	3.23062350387295	11.44192372681947
C	8.76485063033247	9.58051774017140	11.36925024967255
C	19.27765771231651	11.71359093458553	11.96484739584821
C	13.40909191835755	-0.47622476902548	11.93436262933305
C	5.04967350311772	10.55648158248279	11.77097168968698
H	21.26595360704273	14.50381049385693	11.68037906860754
H	15.51310922020125	-3.15769434816118	12.38817818467932
H	1.63047423576410	10.21280119013137	11.68607027016505
C	17.41932301641730	10.07501058884838	12.04674467391745
C	12.43832667568965	1.80579769442465	11.72179135790436
C	7.51404179660986	10.25076780023104	11.67145795974309
C	20.90520182665075	13.52659950350227	12.00492295610758
C	14.60961350342937	-2.54574515255366	12.39400105396187
C	2.62047472305153	10.59265047167362	11.94498251603299
C	20.12646471727366	11.04680421124562	12.88965542945847
C	12.25127896889898	-1.13384146171316	12.42974526974852
C	5.04483932189267	11.66914447566052	12.65687284252173
H	19.88824795370424	10.04951957137980	13.25670500309104
H	11.27751858270923	-0.64453001214033	12.42942106773127
H	5.97004717101261	12.15641128263914	12.96192715726863
C	14.55799550402684	7.67167050483458	12.26378618735436
C	11.36809389494597	5.39526199672535	11.84040373585090
C	11.15295131925500	9.39140284835239	11.86993594415554
H	17.94609499446069	9.57097501496548	12.86293161247338
H	11.53035608712257	1.45353224828807	12.22249498315493
H	7.63160486709289	11.11136696249794	12.33794141097298
C	15.76184229921615	8.32499525065550	12.48549251678096
C	11.38654294043882	4.01287541493117	11.97040717254222
C	9.93946359993010	10.05422771513461	11.99535872807316
N	21.66270363773520	12.87167612149554	12.92053464165319
N	13.49209002914074	-3.11627954275867	12.91117278232901
N	2.66691308121140	11.61684080535444	12.83373781238170
C	21.28236535974411	11.63649295070287	13.34249217094319
C	12.32320925356079	-2.42264791888672	12.90595235023146
C	3.86783946353160	12.16162824343704	13.16659536733889
H	22.97908920286904	14.49573576013411	13.07603410340205
H	14.43355155163998	-4.97304138604660	13.12023694348024
H	0.59187606289833	11.55066710876228	13.08437902434190
H	23.76491192791880	12.90757555748668	13.08397110650639
H	12.66335857611020	-5.02906827175335	13.13320724474981
H	1.27866056206014	13.18427515968918	13.07472132313398
H	14.28171891970608	6.81013924646634	12.87296153918051
H	10.56189862911595	5.96968745536188	12.29590731662358
H	12.02941629852740	9.76198827952172	12.40136215792472
H	21.94608393190742	11.15119862192184	14.05907181875115
H	11.44952873660858	-2.94553992911305	13.29606998871206
H	3.83621285499241	12.99789883949003	13.86636590921107
H	16.42607005892501	7.96429083572733	13.27546754718511
H	10.58308376103121	3.52081186777461	12.52513843085476
H	9.88493236653425	10.94805095829300	12.62187614353235
C	22.91058262334284	13.47602573863183	13.47904212795449
C	13.53567343921100	-4.48010504330415	13.51337646138531
C	1.42462500431259	12.16288592022031	13.45497272629963
C	22.91174034429489	13.47817972535779	14.99318376314578
C	13.54512051076311	-4.45121031976695	15.02794591573573
C	1.47872548807855	12.15318339454939	14.96746565286556
H	24.73132882507519	12.31443583011169	15.16648738533836
H	11.40167121911989	-4.72122511809150	15.22748314538452
H	1.36686819086738	14.30838593523157	15.15700801510706

H	21.11699571498031	14.68891747974071	15.17038999001157
H	15.70167221480848	-4.27339999953961	15.19066956669814
H	1.55043629591662	9.98873286549551	15.13292876774856
C	23.92468557024475	12.82214138514872	15.70265824556777
C	12.35043878378943	-4.57635383705347	15.75108888420655
C	1.43305722230156	13.35352633262928	15.68573402683879
C	21.90275886870746	14.15024720294180	15.70687194916593
C	14.75350166723664	-4.33365170419855	15.73198804750505
C	1.53634506549783	10.93897605275003	15.67398084590381
C	21.90209995167269	14.14948930126782	17.09942219158853
C	14.76572295861223	-4.34038682478481	17.12754340588886
C	1.53701085453243	10.92993370143331	17.06658227034050
C	23.92403533683248	12.82139361168760	17.10410009797988
C	12.36269946016796	-4.58291423983607	17.14821592590158
C	1.43361891430449	13.34444021120559	17.08616686286816
H	21.11577348222727	14.68747754679699	17.63577049307141
H	15.72324673728574	-4.28538958200671	17.65274708197161
H	1.55170305447295	9.97283634664175	17.59541212260628
H	24.73012201084183	12.31303208859178	17.64050434767384
H	11.42307760175234	-4.73232607119510	17.68680395853950
H	1.36783538025640	14.29243189675202	17.62720267571797
C	22.91045232127351	13.47671799987555	17.81331826724304
C	13.56985505199840	-4.46498207552157	17.85143984056241
C	1.47979809634841	12.13489910057662	17.78880862223491
C	22.90752703887295	13.47239965731057	19.32742576600459
C	13.58602533909890	-4.50584335634442	19.36586654034316
C	1.42521359649918	12.12506236304897	19.30112946306914
H	16.43381895156196	7.94398925521161	19.52179942023000
H	10.60466244712726	3.47129453288824	20.31427926898693
H	9.88704386216125	10.90457790830928	20.13581197389115
H	21.94668337776644	11.14785170061427	18.74087448149921
H	11.48221473314231	-3.00893755588431	19.66753637854204
H	3.83855316908710	12.95334915180255	18.88248549640685
H	14.29117133759368	6.78538414314225	19.92416392038956
H	10.57354529933831	5.92286649526780	20.51376716898569
H	12.03345462112314	9.72501355873061	20.37193809480061
H	23.76221982771322	12.90466522426642	19.72275510143486
H	12.72499940471333	-5.06501234289442	19.75641983626739
H	1.27565941329175	13.14115630763912	19.69403644578460
H	22.97378988911298	14.49160119705938	19.73208832544141
H	14.49487799327336	-4.99303909899477	19.74071884719070
H	0.59387060011989	11.50573882449510	19.66294185012332
C	21.28165163817901	11.63047444106996	19.45803099329212
C	12.36164934401402	-2.47053056654804	20.02180324609270
C	3.86963414427800	12.11805130927204	19.58342039047164
N	21.65980124319738	12.86520721509816	19.88324855508838
N	13.54003574747207	-3.14601054299877	19.97620641127808
N	2.66830957446260	11.57500430231346	19.91763007381141
C	15.76719752942341	8.30515860456363	20.30955365040262
C	11.40690587572668	3.97354162734572	20.86158613180755
C	9.94158205774288	10.01555929495086	20.76917285237732
H	17.94883059701465	9.55669999590515	19.93240181527418
H	11.55830586878392	1.40870007534222	20.65022933105368
H	7.63303469778946	11.06789657845921	20.41543996321322
C	14.56466604939000	7.64933068044052	20.53123772984607
C	11.38286702469909	5.35735041021846	20.97488913979607
C	11.15620621516668	9.35658841100927	20.90357631348535
H	19.88928213706318	10.04165979526523	19.53827016354746
H	11.30760711121827	-0.70557421962202	20.53031907277101
H	5.97189652309515	12.11157877585950	19.78777176593807
C	20.12585967299122	11.03835953182688	19.90794010332851
C	12.28802383376102	-1.18005292485711	20.49329367908256
C	5.04639367772742	11.62539748980508	20.09365183543513
C	20.90066278059497	13.51690033153289	20.79972971044838
C	14.66626485815757	-2.55820831141063	20.45350320850296
C	2.62141779574754	10.55173099595361	20.80749306280260
C	17.42001920164306	10.05991861090552	20.74776698755138
C	12.46913651408278	1.77409556505018	21.13634451246188
C	7.51509533352257	10.21073215834900	21.08630209186876
H	21.25984782499036	14.49377882375367	21.12697227565869
H	15.57771462025217	-3.15775251598559	20.43031590146712
H	1.63123089194666	10.17285142665377	21.06713792153078
C	19.27548487708922	11.70173534770377	20.83370274013853
C	13.45386846872136	-0.50236047255949	20.93969115972866
C	5.05062835965837	10.51488286126771	20.98222148675707
C	16.14651147955890	9.43661065626461	21.06713515998621
C	12.46276183903040	3.20243508785803	21.39749387340721
C	8.76619133300048	9.54444584200597	21.39583709497377
C	19.73255044856184	12.96270374611930	21.29001722386614
C	14.64845225373038	-1.26682633620193	20.93915501952517
C	3.77438439774631	10.01687099865768	21.34796432824021
C	18.00885258029963	11.16001493983905	21.29172104405132
C	13.48116229717384	0.88691087608637	21.34761865688412
C	6.25268079260341	9.86955912106908	21.46882739802387
C	13.68788633547399	8.10300550067229	21.53234844185231
C	12.41032456213745	6.03041683130036	21.66450225338639
C	11.24600095668308	8.19294615956404	21.69138748754098
N	12.44449517146646	7.44423514315125	21.73658624155978
H	19.15659509504004	13.53113164351887	22.02289202059158
H	15.57950810128509	-0.83757826838702	21.31310511939407
H	3.68240661614089	9.18730009296746	22.05136772746541
H	17.51028119071578	11.71624934727113	22.08981885783966
H	14.42107927965416	1.23206029820343	21.78633744956478
H	6.09529473887375	9.02083152124677	22.13998968797720
C	15.25597607497371	9.88093734325441	22.07372269562883
C	13.47208263844549	3.88723316299133	22.11694803306262
C	8.88577318789454	8.41427287224893	22.24122352299358
C	14.04988086589735	9.22691316108663	22.30278516843392

C	13.44295874681140	5.26772554641348	22.25534333243285	H	8.01776168188968	8.04020980589217	22.78747076982242
C	10.09967504633909	7.76076173818419	22.39574975557597	H	13.37813191862273	9.57849293614203	23.08842143113066
H	15.51254935394694	10.74055234921907	22.69639528598264	H	14.22498654097416	5.77348363370573	22.82400419093887
H	14.28162906197825	3.32926364015306	22.59111283913942	H	10.16916162829249	6.89271666480912	23.05262278465083

(b) COR

H	0.45937685616487	0.46680868124577	-4.74948801436135	C	-1.04135169324429	-0.96362187502755	0.17872394429100
H	-1.34661996738153	-1.21252635357778	-4.43961778988811	C	-2.07902454963307	-1.92230854213337	0.35673870071595
C	0.14686944467375	0.16610127258298	-3.74571978896643	H	3.49749284887734	3.22028518186352	0.65178239482709
H	2.15417893013001	2.01431166854864	-3.78467551246975	C	2.69920022839434	2.48715397894249	0.79601884086869
C	-0.85310848303191	-0.76225842345855	-3.57379022819920	C	0.63870533411230	0.58205669255847	1.13805874026869
H	-2.79878389389683	-2.56540899725402	-2.93438035302794	C	-0.40210648320859	-0.38149904296815	1.31680018990488
C	1.84541402913961	1.72400915239642	-2.77660071079751	C	-2.46126868093457	-2.28300841632657	1.68183872017154
C	0.80266522978200	0.76237801636517	-2.62853373179212	H	-3.26121933370937	-3.01616501863334	1.81573287489642
C	-1.27488074696857	-1.16278622564526	-2.27171660487549	C	2.31060237542693	2.12154707580873	2.06296015850301
C	-2.31061403138522	-2.12153750313128	-2.06295992872260	C	1.27486938890659	1.16279702660712	2.27171594264911
H	3.26124177752204	3.01614344995147	-1.81573148453970	C	-0.80266118758349	-0.76238097691227	2.62853287251235
C	2.46128341999715	2.28299454747218	-1.68183920165969	C	-1.84539734199980	-1.72402383543613	2.77659971698609
C	0.40210395197573	0.38150179371539	-1.31680104831377	H	2.79876680424428	2.56542042409088	2.93438206301218
C	-0.63871105153939	-0.58205071285452	-1.13805991648650	C	0.85309698211906	0.76226992973780	3.57379087858799
C	-2.69920340459201	-2.48715109542271	-0.79601875348161	H	-2.15415001891236	-2.01433775281894	3.78467506514495
H	-3.49749479992969	-3.22028420155908	-0.65178224821179	C	-0.14687273848907	-0.16609821137509	3.74572009615746
C	2.07902671111865	1.92230790121523	-0.35673914180168	H	1.34660250058271	1.21254115491676	4.43962017065210
C	1.04134950709960	0.96362580168079	-0.17872559808295	H	-0.45937791382718	-0.46680656516515	4.74948868552866

(c) 3H-HBC

C	5.58079672603202	9.60260238699434	16.21394553674737	C	-4.96858331851094	15.11418264277954	19.14145623451964
H	5.37928615120557	9.23859495906347	17.23810458900683	C	-5.73434152511479	16.13714742400684	19.67416577668065
H	6.38823793031680	9.00377789127197	15.77200869259895	H	-6.71411853438126	15.91460432298268	20.10377779525033
O	6.02067015045366	10.94027819311799	16.21074011004996	C	-5.22163793777130	17.44591310519882	19.71678963728445
C	5.21085694668506	11.88900025220436	16.73353293746982	H	-5.80062501917531	18.24846362763560	20.17945962721523
C	3.96267449668690	11.65288131017149	17.28218630104604	C	-3.95499400843805	17.70696033720498	19.22158951843311
C	3.14482584977160	12.70103580417183	17.79309289390467	H	-3.53614606568660	18.70651998104902	19.33261437532506
C	1.78390224434983	12.47713294256224	18.22992546181569	H	-5.34079143100391	14.09098800866920	19.19109866661761
C	1.25306823614975	11.18856686906076	18.64406210601044	C	-3.15448317028800	16.68485010724224	18.65171009093650
C	2.07602218181006	10.18114539841911	19.20979975006637	C	-1.78392382508114	16.92218691492539	18.23500039290099
C	1.54797013513978	9.00026461693205	19.70609330838112	C	-1.27862391517763	18.19814559466053	17.78371676596644
H	2.20770158506186	8.25710316625014	20.16071440974358	C	0.13002165871218	18.40936035833258	17.72424939472556
C	0.15847411867450	8.78529041194063	19.67324218880109	C	1.02603860283939	17.35731342323392	18.14693803318597
H	-0.27109878122795	7.87637867019764	20.10267751058446	C	2.42018073878429	17.56911289614409	18.49134230824816
C	-0.67397902499864	9.75943916410644	19.14870915043527	C	2.90272699967925	18.82229131947777	18.95216532674696
H	-1.75346762706941	9.62161387044629	19.20824806475659	C	4.21231708342432	18.99118807375412	19.36861057608711
H	3.14295020937969	10.37252999805055	19.31994107361879	H	4.54803254933929	19.96172325327625	19.74303539363779
C	-0.16199357738052	10.97160798552096	18.61787728329317	C	5.08835634988685	17.89147053595674	19.37923938037693
C	-1.03824932470750	12.04920594378685	18.19943709006079	H	6.10543578237801	18.00423348412125	19.76279137586592
C	-0.52256498566302	13.35889266338719	18.23811275124253	C	4.63753889238075	16.64869081688000	18.96964741116100
C	-1.41974735020617	14.48152147699242	18.24011940539217	H	5.29491624301999	15.78644366209734	19.07332421052098
C	-2.80900495942866	14.26566587837850	18.19550820677614	H	2.20867214124165	19.65664014935394	19.04277707241307
C	-3.67308124649960	15.35191608021290	18.61630644087325	C	3.31355534291494	16.45136369427459	18.49903263864846

C	2.80885500429463	15.13355302916023	18.15706836135536	H	-2.14185312106520	9.81333912925015	17.09530158447294
C	1.42007302730111	14.91914440107221	18.22005448515711	O	-4.54451768495311	9.41598597076124	15.93855211221773
C	0.51944962460660	16.04508553433443	18.21794411704113	C	-3.69501770459538	8.30080919537279	15.81250809208768
C	0.61365234255980	19.60429801500529	17.12187675979782	H	-3.37999697069186	7.90475272748738	16.79514946565232
H	1.68493615880104	19.72099955548045	16.98925377329615	H	-2.78872470342367	8.54334644818729	15.22817984531050
C	-0.23486395669609	20.56999442435953	16.61615867576527	H	-4.26997110058381	7.52920039921367	15.28269343714496
C	-1.64865158642639	20.36633002556052	16.69136043075449	O	-6.12339319353116	11.46603531825395	15.86894678931050
O	-2.40329560236178	21.34247647851571	16.14083553929737	C	-6.94486440417108	12.58538540633640	15.63111286114490
C	-3.80457061189430	21.23691224207615	16.21238749620612	H	-7.31613001022472	13.03540209633639	16.56941026916213
H	-4.15525112139636	21.17879208192852	17.25891785120243	H	-7.80039961406722	12.22657569609055	15.04351906629587
H	-4.18181797489752	20.35383154908828	15.66656967735151	H	-6.40943479144067	13.36511246410196	15.05807725177423
H	-4.21283859316511	22.14338620315538	15.74624133872273	C	-0.90072542921097	15.82542913864110	18.24679653811310
O	0.16076687750782	21.70522453688774	15.99712807296154	C	-2.39697768697092	11.86563598950360	17.74050112336930
C	1.54103243971212	21.96953749049147	15.90014358884879	C	0.90233636294683	13.57682983282319	18.24303439612128
H	2.01396695779144	22.04018025609113	16.89650653414128	C	3.64623880216879	14.03474055871808	17.73622850172745
H	1.64192159847783	22.93357667085924	15.38379707169793	C	4.91390492045699	14.26133823146288	17.13487365164833
H	2.06934884939715	21.19334322341382	15.31785786089450	H	5.24405037483365	15.28655684576565	17.00002083227352
C	-2.13796908019666	19.20313461612134	17.25527642676386	H	3.56010687939023	10.64452646251911	17.25946602731881
H	-3.20707400515720	19.01020922631688	17.22778573253114	C	5.69094443047700	13.23376280047104	16.63720872143811
C	-3.28177973251519	12.98316087394226	17.72908610583255	O	6.87732475648591	13.40039474848954	16.00764109506632
C	-4.55735059620195	12.83942579810560	17.11564053780217	C	7.32581060580523	14.71592041528983	15.77025395659467
H	-5.19100728446638	13.71824159503701	17.03062435611163	H	7.49674428101014	15.26797912170696	16.71168563940110
C	-4.96137770630705	11.65157254158850	16.53560626312570	H	6.60546758255223	15.28719041976318	15.15631894753295
C	-4.08306283698947	10.52209921874532	16.56477264877030	H	8.27616412798853	14.63277675801429	15.22654406028970
C	-2.83827731078026	10.64504952990316	17.15457722010792	H	4.66429469130876	9.47186504170180	15.61051754357169

(d) 3Me-HBC

C	-5.27958351315868	17.43616243309778	19.62447617360652	C	5.14825594581165	17.88432178775889	19.24008116706219
H	-5.87734137534443	18.23929430669042	20.06457649671600	H	6.18465309993247	17.99736497438091	19.57105546252678
C	-5.82915131889227	16.13933038641159	19.51407105483196	C	4.67006460539097	16.63212247095442	18.90720186439538
C	-7.24790211729489	15.87169614323362	19.94660056439641	H	5.32587905644665	15.76995641774366	19.02044252012054
H	-7.41436401507029	16.16138992931467	20.99833224687492	H	2.27941412698792	19.65253401258644	18.98862284853021
H	-7.50996844308641	14.80876879129650	19.83603396117156	C	3.32967600443845	16.42707448896508	18.48780626875919
H	-7.95968552525059	16.45796945628033	19.34190220797730	C	2.81244604655600	15.10982099220078	18.17462087808060
C	-5.01471164543495	15.12531061270294	19.02993050028197	C	3.63940517592853	14.00463309802200	17.74812771741061
H	-5.39744431537457	14.10537119935974	19.04011693335564	C	3.13528558473121	12.67402237044829	17.82713986407349
C	-3.68363238553300	15.35162092842725	18.59489061787004	C	1.77681149932154	12.46158946326240	18.27394031892463
C	-3.15542138028257	16.67651454779174	18.67541527824360	C	1.24191914995746	11.17562224192397	18.68779729955582
C	-1.77562150082800	16.91690652077493	18.29618929713552	C	2.05948006024847	10.17807360559938	19.27183905887864
C	-0.89458173717569	15.81787555049074	18.29684977860814	C	1.54964732266635	8.98375695981551	19.76844264389311
C	0.52758182906874	16.03430159133286	18.26343264588204	C	2.45727424598995	7.96414656211758	20.40946595779191
C	1.04071756207945	17.34536854646985	18.18754754488488	H	1.89050724652147	7.26209991051894	21.04057436954712
C	2.44610594637240	17.54982815152402	18.49112216291134	H	3.22201944019399	8.44878102104319	21.03759203624318
C	2.96269225463734	18.81069548938290	18.89056082279578	H	2.99230466960781	7.36562979339543	19.65039712014615
C	4.29459397210748	19.00984023673746	19.22382732633497	C	0.15670997239841	8.77355329466286	19.69046186711563
C	4.81839082609955	20.37453537087123	19.59141157252149	H	-0.27568705460262	7.86248955378320	20.11401349672077
H	5.24317767876168	20.38976001498497	20.61005044794980	C	-0.67682426273739	9.73903873209151	19.15230560870854
H	4.02689957972547	21.13732911877519	19.53875664212859	H	-1.75519232077450	9.58589811489042	19.19519455045182
H	5.62816481389669	20.68132628311700	18.90826418610448	H	3.12170977582258	10.38631163523723	19.40577347429612

C	-0.17115120969673	10.95857359663523	18.63731532834493	O	5.97699654830432	10.88391922779916	16.21638157046134
C	-1.04213275913917	12.04074390035896	18.21915638605041	C	5.53220435760978	9.54857035193064	16.24393425839503
C	-0.52605883382579	13.35017128391448	18.27271919498707	H	5.35058317528255	9.19673018683389	17.27609806518729
C	-2.39333856781758	11.86314140214429	17.73799944450536	H	6.32779631198379	8.94120907981699	15.79223723786817
C	-3.27351488825005	12.98415677720996	17.71125079179850	H	4.60276046325047	9.41469520372600	15.66122781756322
C	-4.53382115494246	12.84382303503780	17.06680214466764	C	4.89533036108661	14.21995260683760	17.11879565480012
H	-5.16139432229085	13.72420375421131	16.96406226250448	H	5.22538813454628	15.24212226010185	16.96415337543213
C	-4.93184411984420	11.65730473974712	16.48079774157868	C	0.14096928694066	18.40497103514958	17.78793723800283
C	-4.05887603184474	10.52508498254887	16.52634530289533	C	0.61621045045700	19.60948553541495	17.19759345648872
O	-4.51070850328583	9.42048828611940	15.89018887778832	H	1.68309943012609	19.72261497350805	17.03625340989112
C	-3.65859596194766	8.30671663538664	15.77341885310356	C	-0.23497767837564	20.59678371017869	16.74170364143897
H	-3.35502420510044	7.90924993521709	16.75908883297957	C	-1.64683411829530	20.39809707742356	16.83538881446821
H	-2.74518107444192	8.55188039155089	15.20126252953885	C	-2.12944944684421	19.21887573794479	17.36923549465629
H	-4.22568514462853	7.53520418308602	15.23502933221963	H	-3.19863752531864	19.02963063255358	17.34876972338891
O	-6.08423610044568	11.47825078234408	15.79480899746996	O	-2.40894560292072	21.39547569208968	16.33423941265905
C	-6.90575101989795	12.59927686791049	15.56666411998318	C	-3.80762322315048	21.30189412176780	16.45606614956269
H	-7.27757418854503	13.04081957007274	16.50921478875993	H	-4.11849896131222	21.21066137455385	17.51300059950707
H	-7.76075308214013	12.24598232421651	14.97504452282068	H	-4.21660017052349	20.44268605792885	15.89577182427939
H	-6.37072690988626	13.38525502854184	15.00181361444477	H	-4.22273282682794	22.22826923042268	16.03731747800567
C	-2.82606623089426	10.64459353892504	17.14116957222421	O	0.15914193484840	21.75225670828046	16.15891275693242
H	-2.12995599639161	9.81195425120604	17.09432993563794	C	1.53693591827678	22.04192111394451	16.11728410278633
C	0.89953542486742	13.56403753533446	18.28454219118255	H	1.97716072156419	22.07039445154946	17.13119154962947
C	3.94214324600479	11.61619386960121	17.31834839158247	H	1.63758257428747	23.03153707983286	15.65174535952290
H	3.53660925853329	10.60870001914857	17.31872913855486	H	2.09792797129418	21.30495863184205	15.51538074871843
C	5.17965549954136	11.84200228235035	16.74262078457920	C	1.42285873965996	14.90378836593179	18.25600626319163
C	5.65937437690677	13.18438550205796	16.61789549172980	C	-1.41988145728932	14.47567339835193	18.27149069592212
O	6.83222277676469	13.34152020683904	15.96017949501160	C	-1.26762917306186	18.19893353506397	17.86434918791971
C	7.27502922153416	14.65362151169474	15.69611253866447	C	-2.80995393656409	14.26429345019125	18.19677505482054
H	7.46289105295009	15.21901012130173	16.62634457666150	C	-3.97978876926881	17.68948475349488	19.22890643200251
H	6.54280721435306	15.21591368851410	15.08773280911964	H	-3.55935485174439	18.67976694484310	19.39888450194994
H	8.21539901492007	14.56349035699439	15.13635527734339				

(e) 3F-HBC

F	-7.02710053759113	16.02024440436295	19.91273322257377	F	4.55639068744704	20.03960721541040	20.20567485294784
C	-5.77578302700550	16.23765597207913	19.47459113428064	C	5.05191492237549	17.80309556021835	19.63609511856748
C	-5.27108069456027	17.54324146639890	19.51244024380550	H	6.04518776098960	17.91913223423688	20.07304237060269
H	-5.88736139368405	18.34772561132856	19.91638820323400	C	4.61466196240634	16.59387976642210	19.12472513238740
C	-3.97370265904001	17.75925373243378	19.08443187541426	H	5.27341480144657	15.72825338599023	19.18777987326303
H	-3.54738947948147	18.75640066065960	19.18394735613510	H	2.20123672579101	19.62184401401110	19.28815010745807
C	-3.16041808748697	16.70834309878759	18.58933883667030	C	3.30510556939787	16.42767231183962	18.60670141781112
C	-3.68754891387712	15.37727404173952	18.58155511180484	C	2.80223707516164	15.12849933026133	18.20467261387450
C	-2.81897083586287	14.27173892314815	18.21704563848548	C	3.63750865205208	14.04924969097243	17.73928733303150
C	-1.42765270768819	14.48377111709981	18.26919241746262	C	3.13749552005661	12.71498964749988	17.75450040613267
C	-0.90180732486643	15.82580636019626	18.26787325678767	C	1.78168846528658	12.47913793157034	18.20367188681581
C	0.51818114340031	16.04177632497874	18.26976209616070	C	1.26380357510119	11.18443963520970	18.60516224424330
C	1.03046150040956	17.35293747382468	18.23526390940811	C	2.11482387619270	10.16612480966841	19.10101582016704
C	2.41292372910572	17.54563251981904	18.62964449288498	C	1.58983989772292	8.98457991126450	19.57977411106510
C	2.87230092010991	18.77285843413854	19.16915594672861	F	2.42310208085898	8.04522425319245	20.05802341776892
C	4.15930666357713	18.88273504623660	19.65217626239005	C	0.20875387781090	8.75480246813137	19.62704909202725

H	-0.17330412288843	7.83099463261813	20.06522853168441	H	8.14133725143780	14.75416910952697	15.03620961841434
C	-0.63887393575908	9.74849416220432	19.16670051708854	O	6.00253651898325	11.02427415267038	16.08182453367312
H	-1.71374597644855	9.60895521385230	19.27824344141438	C	5.58122189078668	9.67965822087103	16.06259668025643
H	3.18861414534326	10.32363213487506	19.18079171620519	H	5.40234655784993	9.29114578798025	17.08165434258732
C	-0.15196586617370	10.96874153848614	18.63097217217141	H	6.38980994949776	9.10267461884161	15.59486592887251
C	-1.04378187797995	12.04797015141820	18.24679457312103	H	4.65750132736338	9.54946355318550	15.47024615489344
C	-2.40742345550161	11.85950638875667	17.80559557485329	C	4.89418451342676	14.30568466224576	17.12763528508270
C	-2.85133349394393	10.62869302345682	17.24228828529521	H	5.21752145732260	15.33760342154121	17.02790410899085
H	-2.15450835644130	9.79726944878545	17.18991776613847	C	0.15816643499092	18.41457303593680	17.79187948590799
C	-4.09827254126524	10.49293329053002	16.66123257491040	C	-1.25009449605086	18.19735077446170	17.77316917142569
C	-4.97727098464578	11.62100372067657	16.61718992592776	C	-2.08153273607381	19.18596318645517	17.17494952114444
C	-4.56917055249864	12.81937559466314	17.17144174942915	H	-3.14438294955296	18.98278316737242	17.07651888035322
H	-5.20507750202423	13.69325585480351	17.06672219823271	C	-1.56534970757683	20.34234338060330	16.62227669332465
O	-6.14413556476994	11.42282871919704	15.96615032706224	C	-0.15232273304275	20.56617558114632	16.65218124075038
C	-6.99716917799387	12.52671242166136	15.76722686542684	O	0.26858454666509	21.70123932921812	16.05363123745975
H	-7.34346321894692	12.95959984271492	16.72284381279322	C	1.64876953790953	21.98597646278066	16.04377059734405
H	-7.86475989182414	12.15427161877786	15.20684393776034	H	2.05450093256689	22.07437371504158	17.06786407220337
H	-6.49930538059953	13.32255280227330	15.18305136699679	H	1.76835004562057	22.94584095734831	15.52416368030724
O	-4.56105821725834	9.37665907906799	16.05752109609938	H	2.22408397421679	21.21209819468356	15.50481274936827
C	-3.73407386360929	8.23828313715040	16.00395780438484	O	-2.28847027772858	21.29268845356524	15.99187387293586
H	-3.44548641610845	7.88934314023757	17.01227985566904	C	-3.68947882406952	21.16495838183219	15.95289471397123
H	-2.81242727555547	8.42929428261200	15.42505927845624	H	-4.12362076716590	21.12401674370304	16.96820701676423
H	-4.31695545718695	7.45266532775967	15.50480767870329	H	-4.00669834271277	20.26363202522995	15.39813098909079
C	0.89581631577944	13.57466762952185	18.25582146830600	H	-4.07192151953399	22.05407799500438	15.43468378388168
C	3.95397431542879	11.68785618695422	17.20074381039904	C	0.67060528423741	19.61562929027129	17.22597885066299
H	3.55849552746512	10.67801162806821	17.14711377018658	H	1.74741439027870	19.74644456201560	17.17011236094348
C	5.19305874309423	11.94946646828629	16.64168000629158	C	1.41422772176553	14.91471814354076	18.25869196585226
C	5.66189145544242	13.30049563160865	16.57368636742391	C	-0.53000014160899	13.35882424513652	18.27271379910833
O	6.82406804012492	13.49388071283590	15.90998430252338	C	-3.29198961990210	12.97808802965725	17.77782443974294
C	7.21590955522051	14.81826444151196	15.62378704144053	C	-1.77623301408430	16.92750648540456	18.21914186817135
H	7.41479993258217	15.39814298015901	16.54228174846359	C	-5.01153184762449	15.17192752500702	19.04627902776240
H	6.44573088861550	15.34863893265605	15.03370401833962	H	-5.42787489342090	14.17039931640976	19.13692394316394

(f) COR⊂TPACage^{6+}

H	12.70993789665936	9.62761296631867	11.50084412791269	H	18.28607620618509	13.59553944255033	11.78829403693075
H	13.69105834684388	5.45672101607482	10.91729375110694	H	14.41831017612470	-1.55491205305442	11.37382139670959
H	10.02896730878130	6.50533965634852	10.90208134611352	H	3.46969692592370	8.44263174975418	11.14729430314415
H	14.81985822988402	10.82697474944694	11.76615338600284	N	12.16021078020652	7.13598728403825	12.37459459772927
H	13.61430129845328	2.99808486888285	10.96865775387263	C	13.38834709702889	7.82088213167924	12.49900507538879
H	7.81445544152162	7.55308633021948	10.99010566481884	C	12.12764493001502	5.71714101169851	12.39340968214362
C	13.53992590896458	9.13833170309456	12.01074381609907	C	10.92001907511705	7.82058849856042	12.37688580032889
C	12.99067252017580	4.95084952469113	11.58374017179931	C	17.50139792482531	11.09774167931652	12.52946890918126
C	9.85965594302588	7.35011360081783	11.57095907385872	C	12.70916952543233	0.47266131861555	12.02743522278611
C	14.74171129978439	9.81299841857562	12.16149715418136	C	5.87796728590510	9.27700387755917	12.08178828596539
C	12.94478586674593	3.56228081145693	11.62063187508864	C	19.06144795518804	13.01606030523246	12.29247130414781
C	8.60910149169796	7.94596313340296	11.62686735474539	C	13.52705243575617	-1.87857851551265	11.91460424834151
H	16.77015324595244	11.72735191496106	12.01656629393556	C	3.40807524816527	9.28526609627886	11.83859570110786
H	13.57218843721849	0.74396016378324	11.41361382789902	C	15.85887615035916	9.20054754660767	12.77688297687238
H	5.85868300723186	8.42426772650499	11.39812875032896	C	12.02652923581331	2.88413580563182	12.45692290808197

C	8.36096801144819	9.04681624591799	12.48162654545519	C	22.55894123586596	13.62035078966846	13.52366884404226
C	18.80646332494285	11.68725053883350	12.71574247766818	C	12.25690764491890	-5.08676562795308	13.41738961800973
C	12.52806304561821	-0.94409110313030	12.28281610350243	C	0.67155753561892	11.23701012057819	13.53316464587627
C	4.58614614485244	9.86294143161092	12.37486383881526	C	22.65200887680421	13.70634641335754	15.03080425951631
H	20.50438982787606	14.63358252235088	12.20588972912339	C	12.25193041162989	-5.14295192407112	14.93020605314720
H	14.17918197540861	-3.94533531821130	12.00131512459449	C	0.59829897546335	11.26996313680722	15.04317433043543
H	1.24010446628408	9.30309316751280	11.82026927245138	H	24.35270347655866	12.37652147858877	15.19777353139816
C	17.14419611205671	9.84972404695109	12.94418655214305	H	10.13738585385084	-5.59394911591049	15.08727196412869
C	11.91706554326265	1.43989925553304	12.56519025350845	H	0.40350920828730	13.42416836016269	15.17317674132031
C	7.05729273405893	9.66682564873975	12.64070283121904	H	20.99893867483883	15.10399426963883	15.20410499479767
C	20.28755863231378	13.61025209854731	12.51589544873059	H	14.38498151078172	-4.78898185168589	15.12748444826584
C	13.41106469595985	-3.21327954404836	12.25596503819272	H	0.71331360456688	9.11270090668539	15.26060624921428
C	2.15913373484800	9.75028105493171	12.20253477577951	C	23.60218399687343	12.96101589344731	15.73710222673420
C	19.89846548563795	11.00937217101357	13.32025886378894	C	11.06151419436693	-5.37831889182774	15.63014600827850
C	11.38430720925123	-1.48567857165943	12.92868105962464	C	0.49268193989428	12.48603246046768	15.72787536677745
C	4.39290531703840	10.98328235053202	13.22731371444623	C	21.72500932349044	14.48817675656283	15.74189653355199
H	19.81113209645300	9.97537347029660	13.64819450522212	C	13.43772130955165	-4.93457681750566	15.65434780731083
H	10.53210687772451	-0.85984017996612	13.19048604919332	C	0.66574520740128	10.07443137209024	15.77918017206638
H	5.23607921312665	11.53025821405379	13.64777155946665	H	19.33557401112591	12.49192128949312	16.43070075511767
C	14.49319499154918	7.20102512930725	13.11335145376050	H	17.22149735183679	13.78477693022952	16.43782570744824
C	11.21262186292728	5.05508451255401	13.23140055338044	C	18.37287738042089	11.97380230645523	16.42461971864514
C	10.70025298756412	8.93918708102601	13.20123798488676	C	17.19966561413169	12.69174461223862	16.42830604313184
H	17.88950805974640	9.23801590772329	13.46124483310016	H	20.51912015889739	10.29339517073644	16.41956233956456
H	11.09093852595145	1.11981932057126	13.20815740505544	C	18.36101643656608	10.54912910989656	16.41349522683302
H	7.05089974363344	10.52148005959817	13.32589994109431	C	19.55809316644797	9.77280514368797	16.41192160418633
C	15.70057130776655	7.87417223753091	13.23188816516864	H	20.44819496706385	7.81948491899612	16.40055241687290
C	11.15998921170537	3.66888051374672	13.25082639386257	C	19.52051220949055	8.39767779081077	16.40132898063756
C	9.44707363436390	9.53772936869298	13.24183522385195	C	15.93188640802433	12.03939223133072	16.42052186364769
N	21.28691352928165	12.94063467455928	13.14145167749374	H	14.73915010386778	13.85665903789780	16.43548368365488
N	12.33018374890819	-3.67435627627593	12.93270535618107	C	17.10825660315157	9.87142726450979	16.40449651302744
N	2.02000981135595	10.78348587571266	13.06982634603311	H	19.13990796711665	5.70042140868235	16.37927991542578
C	21.09587218550796	11.64918266105191	13.52280254473724	C	18.27729540628515	7.69574573197851	16.39172095526428
C	11.31695800120288	-2.82289190031522	13.23930505104952	C	14.70437502481450	12.76405551797512	16.42558511895206
C	3.12698934687096	11.40694927006064	13.55446234101444	C	15.89121759909417	10.61814818403467	16.40797748374807
H	22.55299811179043	14.61038208024478	13.05016037242692	C	17.06587316170038	8.44373505227068	16.39372449857277
H	13.11920731018004	-5.61649136084046	12.99285235642052	C	18.20857744120800	6.27169788258950	16.38125219696276
H	-0.06194543477085	10.54110595610374	13.10555436574323	C	13.49457131678038	12.11192313587131	16.41904753979623
H	23.39506533160857	13.04941797099115	13.09674385904623	C	14.63457271356695	9.93932024420739	16.40044651283880
H	11.34674045194206	-5.53617230765774	12.99600471180861	C	15.80802506296742	7.76590652566133	16.38541972558758
H	0.48139654478137	12.23101802467075	13.10340344832163	C	16.99705375860240	5.62090940541236	16.37391044976609
H	14.39924393950106	6.19502888792578	13.51655630242969	C	13.42397991950035	10.68814863507259	16.40638692587534
H	10.54258906202616	5.63900318549632	13.86328664977296	H	12.56473078339653	12.68582275894729	16.42353182739763
H	11.50731044736613	9.32028915477292	13.82460427227292	C	14.59248349885042	8.51295402428687	16.38824254165552
H	21.94048654915014	11.16524865456550	14.01412684616368	H	16.96342751032994	4.52833926060441	16.36602994136894
H	10.46072568027476	-3.26001427447624	13.75436515554437	C	15.76892687147100	6.34411332391926	16.37651446508867
H	2.94582097998878	12.24569117435870	14.22697697333658	C	12.18522187398205	9.98302856300393	16.40006082359795
H	16.54493005727591	7.36725264527002	13.70198256895775	H	11.25593420110837	10.55919249772093	16.40403736793596
H	10.44195116028688	3.16905165965010	13.90635017207394	C	12.14545043123652	8.60801488709044	16.38873080686777
H	9.29058673214792	10.39727547305889	13.89887231343906	C	13.34145397700998	7.83573314398261	16.38241598943407

H	11.18437648911695	8.08742750723779	16.38449500718902	H	7.02217713245755	10.60229887131585	19.53840193163718
C	14.50149372813318	5.69143360084224	16.36987645892064	C	14.43988689795918	7.22293244586811	19.65186867061144
C	13.32988596121578	6.41086339945961	16.37232287472189	C	11.10915247211340	5.09252386667341	19.56773011347564
H	14.47799430828940	4.59845712981451	16.36206046421102	C	10.64857121062144	8.96864055931044	19.58670038100925
H	12.36735316018584	5.89341504764006	16.36758018571524	H	19.78212857051709	9.96906103204075	19.18194957271994
C	21.72142435933888	14.48382331459419	17.13310221901586	H	10.47765263499933	-0.83566853509878	19.46652236965501
C	13.42382845355313	-4.92851799261125	17.04822351499353	H	5.21949234944211	11.65242341020795	19.27203832095809
C	0.66083495254552	10.10127336810461	17.17071026681293	C	19.87167448661715	11.00023024509263	19.51822647833781
C	23.59859350314190	12.95663895896110	17.13819579137865	C	11.32676000660847	-1.46148480658412	19.73816450847427
C	11.04756813758217	-5.37199595974309	17.02921243068754	C	4.37611625315782	11.10343570796885	19.68950354842950
C	0.48796826251463	12.51298718891275	17.12807352701209	C	20.26714356330222	13.59257679720153	20.34677588458542
H	20.99251621265508	15.09621180757711	17.67099745042048	C	13.34888261780932	-3.18920925166431	20.42546229671563
H	14.36057811862805	-4.77858793900826	17.59228408363354	C	2.14183726117730	9.86937884228192	20.71240178093194
H	0.70434961328677	9.15992697625037	17.72578130069945	C	17.10655673831428	9.85368016068371	19.86035666220464
H	24.34633000538459	12.36878792326548	17.67774945747476	C	11.83518094651775	1.46734847388446	20.15553106307707
H	10.11282570487539	-5.58298793264462	17.55554011555988	C	7.03196152407853	9.75269352381602	20.22984420015131
H	0.39545510951908	13.47149887523470	17.64607728787983	H	20.48640091304791	14.61243918792682	20.66633470139628
C	22.64481334557978	13.69766898047106	17.84412825399173	H	14.11510029097215	-3.92123965658008	20.68572727486911
C	12.22378568052659	-5.13021279295155	17.75054501470878	H	1.22262302917325	9.42184340738198	21.09379755338862
C	0.58862612688286	11.32421186132130	17.85965235518185	C	18.77922633374008	11.67759749084863	20.12247570457507
C	22.54347598962170	13.60213634566355	19.35014364177798	C	12.46507984096110	-0.91976425918683	20.39409788177218
C	12.20014850484589	-5.06471797384300	19.26290864797692	C	4.56816697563396	9.97607139706659	20.53282978979014
C	0.65740790718804	11.34929789036558	19.37041717851601	C	15.81664831901522	9.21049176832913	20.01542812092399
H	16.49281356871804	7.38539070898448	19.06743889690853	C	11.94722524998237	2.90957241669516	20.28141661877394
H	10.30374020684094	3.21703082202721	18.90391157109608	C	8.33124812456330	9.11717079807684	20.36596657851006
H	9.24009042662569	10.43816911203609	18.90834596140375	C	19.03755536857124	13.00142577514441	20.55895568564580
H	21.91821219557373	11.15327380945712	18.83729965256229	C	13.46303640761202	-1.85441488156339	20.76597892585762
H	10.40926559304394	-3.23605975118082	18.90599264082481	C	3.39076591796074	9.40074740592633	21.07241314253271
H	2.92956102638639	12.37384984618089	18.69935505996774	C	17.47025149308179	11.09273407934979	20.29561409317940
H	14.34115142491315	6.22335778965827	19.23404182127672	C	12.64429728313952	0.49495383889943	20.65859990538553
H	10.41433950792911	5.68514712161679	18.97189262288659	C	5.85869379848979	9.37937186676471	20.81157782119846
H	11.44668057239840	9.33129958213557	18.94074599411738	C	13.33877191535232	7.83988197690818	20.27599033876144
H	23.37532633396233	13.02551209833960	19.77782198942673	C	12.06240426846944	5.74272678186000	20.37283401952568
H	11.28007776911845	-5.50760444555879	19.66939151448243	C	10.87364087059940	7.85773952436432	20.41936634658643
H	0.47306618996044	12.35978979805622	19.76231242129349	N	12.10771941496042	7.15979728154354	20.39978027781441
H	22.53824219762091	14.58902888175557	19.83014409534883	H	18.26187290764231	13.57978984905345	21.06389615069438
H	13.05151835506443	-5.59693571654647	19.70588937966892	H	14.35077764223974	-1.53062878796822	21.31244264623304
H	-0.08003092180786	10.67423457260130	19.82378486869518	H	3.45233064272463	8.55531576193177	21.76027895177436
C	21.07278667754912	11.63674194077285	19.32761124274413	H	16.74108225308742	11.71867606985275	20.81593963586499
C	11.26151564181266	-2.79891905284615	19.42774844787958	H	13.51925616797516	0.76255437566290	21.25688496749484
C	3.11034419746246	11.53090486650457	19.36684970580894	H	5.84086988124821	8.52901262063775	21.49826082922209
N	21.26692714864948	12.92428271366290	19.72055652509509	C	14.70145632821053	9.82228686909522	20.63491869366761
N	12.27170756119044	-3.65100467774100	19.74309221192283	C	12.89856816504999	3.57565291364946	21.09059506508442
N	2.00340139140256	10.90533985164365	19.84869115878106	C	8.58557206225369	8.02308323802598	21.22731772522416
C	15.65092459085528	7.89127307303616	19.54278316302481	C	13.49625536938201	9.15198188091670	20.77679693810757
C	11.05095128152462	3.70670642631009	19.53412931940854	C	12.95127603997675	4.96304726643103	21.14189594935191
C	9.40292060483580	9.58422477882866	19.57104642811747	C	9.82931627314845	7.41064315689304	21.25813047728030
H	17.84879978264124	9.24565921228864	19.33456564516723	H	14.78367222625015	10.83199022719335	21.04021014574376
H	10.99054648984688	1.15474249083875	19.53325668780097	H	13.59115437591054	3.00195543462702	21.70926487729559

H	7.80207424570589	7.64921754847004	21.88901393156733	H	13.67926407498820	5.45786777301413	21.78664336282209
H	12.66815748221343	9.64032932468353	21.29057960202911	H	10.00513689470023	6.57128231368073	21.93233026783914

(g) 3H-HBC⊂TPACage^{6+}

H	-4.28309746818863	14.13498285740313	11.71110890468213	C	-1.32324800174761	14.06193446604053	13.40383666417815
H	1.53922253610335	18.81718245622131	12.03678376865522	C	-0.12182180126362	16.18129354022423	13.42723165410566
H	2.55836220395475	11.18285540431458	12.43856376442473	C	1.12282272537304	14.06547278844899	13.55981794191712
H	-8.53949307649116	12.92033709784191	11.44474717000179	N	-0.09712749443416	14.76829912775997	13.48239377684892
H	1.80815961882035	23.31529270172082	11.32414712230283	H	-5.04339598291185	11.06038220760885	13.91110128002404
H	6.06188033594939	8.55574963393507	11.87197496431550	H	-1.13940716012573	20.83379304802119	13.94329061241625
H	-2.14441796222316	15.32877519523011	11.84700338990975	H	5.67087319747561	12.59534347455236	14.19895153131518
H	1.62116245789173	16.36664990166916	12.14864941231802	C	-7.57746162405767	10.32550336732153	13.44222883479861
H	0.43185507989592	12.38401647921389	12.37473907704307	C	-0.57589207631883	23.39923083133706	13.76741874300591
C	-8.58858033035059	12.03033365593141	12.07434709264174	C	7.67503083454829	10.85325167625438	13.81224250273252
C	1.11925121165824	23.69296663402217	12.08241040681525	C	-8.82646698314532	9.78512729689281	13.62659418085956
C	6.83844636526626	9.04448805110239	12.46294231865631	C	-0.58883411799017	24.76092305679558	13.94202212185633
H	-10.74198626376363	11.85460256467125	11.87896546810779	C	8.87284501953487	10.18976996500038	13.89628236820726
H	1.69684262090870	25.75984472949832	11.75738753023996	C	-11.27907724600324	9.83079344564588	13.50364109394837
H	8.27176951921911	7.44985279998988	12.12751922286071	C	0.27513018474783	27.03792368389026	13.58699181233083
C	-3.53208774752108	13.79358383225983	12.42632483810747	C	10.31678456293232	8.22500067782986	13.61872932640620
C	0.78395943648004	18.28907651162392	12.62182015021916	C	-2.75501726336394	12.25119260606815	14.12156304068196
C	2.48098790463337	12.13981548702107	12.95706616757235	C	-1.20060471752603	18.26772580985227	14.00179106270212
C	-2.32572732686117	14.47334856643471	12.49862167294405	C	3.43820300639149	13.93272766930503	14.26119467163925
C	0.83833703738829	16.90364723559162	12.68540750740685	H	-11.40468331373452	8.82340902527172	13.08228748316835
C	1.27133909941420	12.81389223761255	12.92084056247821	H	-0.65591212836509	27.50273714094540	13.23139337539958
C	-9.82114103365718	11.45036039198584	12.30248947405542	H	11.16985229777706	8.81468233669171	13.25420996606672
C	1.06962653678473	25.05614574313067	12.30719830258961	H	-8.99000322077308	8.90056933687594	14.24145497682313
C	8.06209559469521	8.41575138036476	12.58945444416904	H	-1.23144096700340	25.24699545127655	14.67583996748674
H	-6.19083015536376	13.10912696786799	11.90734726844617	H	9.71490645730604	10.58389076316577	14.46481151439569
H	1.22137382324234	21.04257125851097	11.96866978906346	C	-1.55438225809195	12.94105014552432	14.21816355355327
H	4.53258978488210	10.32672978728766	12.44669781314903	C	-1.14092422223428	16.88578851455936	14.09280381348322
C	-7.41722148592044	11.51663592389753	12.68442343866842	C	2.22337168782881	14.60465079581594	14.25310877747970
C	0.31043131861554	22.80174286890863	12.83049008305373	H	-6.73710958307749	9.84101692860358	13.93686395903253
C	6.58415899214796	10.28378506872086	13.10242706243108	H	-1.21585615828150	22.79736444866363	14.40987359170102
C	-6.16751402667694	12.23364718370578	12.56127505681370	H	7.57729052439460	11.79216415679111	14.35162801325555
C	0.43755633587000	21.37075114429645	12.65612727834039	H	-2.92239524935502	11.38704872917701	14.76324856639373
C	5.26992452252947	10.88231381207051	13.03205667388763	H	-1.99509018329808	18.79995196291817	14.52291471328219
N	-9.94072415847726	10.35583889302537	13.09243795868104	H	4.28308629502706	14.37245043933410	14.79170800500090
N	0.23836026674277	25.58213918210490	13.23913311078272	H	-0.79579889118827	12.62216874612185	14.93312064997079
N	9.06242000400816	8.97431583055507	13.31291530952756	H	-1.87684582222422	16.34306360700445	14.68656160190603
H	-12.03589292089701	10.48446711977936	13.05105601853744	H	2.12076155973772	15.55082345713954	14.78355138241650
H	1.11003928544211	27.48083581635109	13.02810467841280	C	-11.35536135692734	9.81768337494210	15.01348388481765
H	10.28608080718305	7.28762341495601	13.04872201522334	C	0.43921758634870	27.18846409962513	15.08206719748281
C	-5.03013650742419	11.92859675155897	13.24546260444471	C	10.39548227486203	7.99252886769998	15.11258994555449
C	-0.33878411614709	20.45665015125772	13.30143228550323	H	-11.29896817665913	11.98100196507864	15.18463602282939
C	4.91005912294088	12.05126627117577	13.63389764097995	H	2.45187134344263	26.37778390782501	15.09143319411110
C	-3.77968241897916	12.66450252108182	13.24264855519967	H	8.63375757899871	6.72411270037877	15.16383270065664
C	-0.23865822950192	19.00997062470314	13.28230982925704	H	-7.05908709287222	12.95119050071800	15.57305054152264
C	3.60782018877399	12.68897705878236	13.61286236737962	H	2.31285593888951	21.16471782567924	15.17854272356280

H	4.88896120074182	9.10731029535440	15.63505291490746	H	-2.22272200010626	9.55412789201372	17.09189955315622
H	-11.48603817816230	7.66326432283121	15.18682155432358	H	-2.94206252687082	19.03682009320913	17.09845895487927
H	-1.50537836992965	28.08576000387093	15.40892063746726	H	5.57176658379345	14.86541068428405	17.11204522012356
H	12.20131839263601	9.15419936450501	15.39508422190865	C	-2.89381588414706	10.40676200211375	17.13953033391212
H	-4.63025730776506	7.11658776304228	15.88966823425280	C	-1.86405193253126	19.16566902898784	17.11542083307766
H	-3.84038979455468	22.37515118987989	16.07258904197710	C	5.16558464714895	13.86421943675218	17.21306025959197
H	8.89565185494359	14.00408196728640	16.06699559036340	H	-3.56725459731043	7.62452630761174	17.24351113138604
C	-11.27923458351313	11.02899700658229	15.72270687414330	H	-3.80275974047759	21.21085689719350	17.43898122425577
C	1.60912616547895	26.71526444339830	15.70074229568815	H	7.90653895935463	14.61902946662013	17.43293772430713
C	9.39278347606409	7.24438259898609	15.75467727568945	C	-11.28700187833338	8.61020151323197	17.11775259836108
C	-11.38935463429856	8.61232145397883	15.72112371021199	C	-0.52180607750411	27.60458424279921	17.27216462968309
C	-0.60581622640296	27.67488773604159	15.87570476588714	C	11.34809810131966	8.52957505335683	17.27975147801835
C	11.39230997602494	8.60215133196188	15.88159957715151	H	-11.10602913275594	11.97618108738837	17.64817518495354
H	-3.14008129770812	8.05750703415016	15.55289189884455	H	2.59813313018130	26.25049660875931	17.55711200978810
H	-3.87470825795905	20.61669857458549	15.74469653337935	H	8.54699118117800	6.61088648886120	17.63176435820790
H	7.34611461194554	14.84206701110810	15.74059975283609	C	-3.22982026355066	12.81273918111462	17.46069148746631
O	-4.77813924325313	9.08343798579135	16.35267934321769	C	0.36686372107155	18.21429662382047	17.39864996055599
O	-2.04884160426570	21.45892503889103	16.32846055355545	C	3.25002560156460	12.40016555285139	17.61618830052416
O	7.25461020538503	12.88039565569485	16.46673429636023	C	-2.36934630139687	11.67682095333262	17.51535435221621
C	-3.97392754893341	7.91379090162067	16.25981801609123	C	-1.04654328005411	18.05473821028639	17.46580264604630
C	-3.46704546029951	21.39898176978224	16.40551293440704	C	3.80407132849001	13.71261596390802	17.59573599367293
C	7.87225254330770	14.16146594841465	16.42971109286970	C	-11.14438267869661	9.81301247166857	17.81775439992309
O	-6.34219945070776	11.12686047991407	16.29650748901442	C	0.60710075429775	27.04731727416685	17.88435727260229
O	0.53095970724103	21.70101750840977	16.12170542851887	C	10.30133587017503	7.85469248491524	17.91943268134217
O	6.22594330691130	10.48285630062248	16.46025598977923	H	-11.30873014700980	7.65907515108296	17.65673866903945
C	-7.29661500631112	12.16972380511552	16.31422442331669	H	-1.35786592163824	27.96083389430287	17.87999207533518
C	1.91958528154678	21.88264361634322	15.91697410214832	H	12.12339375431182	9.02683959812871	17.86912699991355
C	5.72134493759795	9.16453030513615	16.35652134019828	C	-0.43085698802543	13.15598663063952	17.78571064163004
H	-8.25975055866343	11.70815278038668	16.05959652343993	C	-1.30258345572853	14.30177282883303	17.73953810911435
H	2.04283022622315	22.90105109094154	15.52713473422951	C	-0.74569952393341	15.63222151089949	17.73273509004870
H	6.55285621343757	8.54362177123368	16.00022502751628	C	0.68375113091112	15.81291316904221	17.74262650371150
C	-4.21442500509328	10.24272450872990	16.75560472546566	C	1.55876200861138	14.66846796614454	17.79061911606474
C	-1.32488342083130	20.37252133923393	16.70911946841866	C	0.99997710696014	13.34211460658886	17.82313070055492
C	5.95884995827052	12.78686967118804	16.86922461356597	C	-0.97868004763363	11.85931439966100	17.87407595800425
C	-5.07617823348455	11.38229448194834	16.70976162637678	C	-1.59492643396131	16.75714075842865	17.79847065127141
C	0.09104076148378	20.51014892776832	16.59093177051425	C	2.95482788622567	14.84959797828913	17.86859322190145
C	5.39684716679858	11.47338911687578	16.86520764309165	C	1.85142109213787	12.22644473783502	17.94243998193090
C	-11.17167880446122	11.02657368528879	17.10952616270259	C	-2.69953248936967	14.12027622076072	17.78028300404872
C	1.69257947683610	26.64505015754849	17.08771555612546	C	1.22451999940430	17.11185688349525	17.76454767510110
C	9.34425957738321	7.17828198121892	17.14359785239319	C	1.28231084868724	10.98966066515123	18.44595012188382
H	-5.19930777741062	13.50522279477229	16.88617270838178	C	-3.51185366236929	15.23168318343052	18.24145223150073
H	1.97033876371023	19.51287762425688	16.75370554446492	C	2.58715234714038	17.28485387747830	18.23097644433048
H	3.62456047250139	10.32061507360408	17.15669278826546	C	-0.13755709131771	10.80450856961381	18.41321412764354
H	-7.36862762157682	12.63072840476447	17.31408393040050	C	-2.95161458347379	16.54892098661426	18.27218006103813
H	2.48212079178418	21.78037961072595	16.85995465416964	C	3.45536240488194	16.14830946863206	18.28671174701369
H	5.37613759694105	8.78387823466116	17.33316471439863	H	-8.70134668833090	8.95035905049615	18.23348498914712
C	-4.57322589535042	12.62860690228652	17.02701361410278	H	-0.71151054977364	24.99664488967654	17.98476392085286
C	0.89894387054138	19.43942295849719	16.91623365479381	H	9.52416522372885	10.20816314392849	18.25572068156932
C	4.07227897587212	11.30785966899007	17.22474123243015	C	2.07389308027567	10.03098049018463	19.13065245657488

C	-4.78048252952712	15.02673200178345	18.84267605484975
C	3.03394605443300	18.50900662694040	18.79738768131873
H	-6.36311645820142	9.64220325780275	18.65564272371848
H	-0.92057767938826	22.55714598660419	18.33161146464606
H	7.65033151655635	11.74945573208899	18.69203579273029
C	-0.68150011604857	9.67029993981125	19.07408944068634
C	-3.68116670151248	17.57332581592020	18.93166951679518
C	4.72013993616808	16.30675138131631	18.91431261879602
H	3.14152067585391	10.21031301184333	19.24198321152164
H	-5.18078807093397	14.01863661346642	18.90363442333584
H	2.34715280835374	19.35080979177778	18.85327154499897
H	-1.76178877434588	9.54812955676186	19.13535798288583
H	-3.23070223274568	18.55612598282676	19.05576658209924
H	5.36019936720292	15.43902997604782	19.05584876165703
C	-8.45914347008895	9.71383715121434	18.97269101252821
C	-0.21818139776786	24.49856184156071	18.81707203829456
C	8.80113897229864	10.00775471647884	19.04519232478151
C	-10.90338903164422	9.81858456672573	19.30765190412827
C	0.62557360315115	26.76539786764802	19.36518701526651
C	10.11918689256604	7.94677172762464	19.41373138187890
C	-7.16630015364744	10.11345386055619	19.22146515508575
C	-0.32690642195593	23.14457577583320	19.03161198106051
C	7.74876055761616	10.85346980873567	19.30301862756041
C	1.51258606410062	8.92644752090093	19.74723876389462
C	-5.47904629780902	16.05414619418221	19.44911232450729
C	4.28662365215974	18.63536119064086	19.36990267656152
C	0.11856453855048	8.74525492295264	19.72207641847744
C	-4.92019107409896	17.34184151051850	19.50216640749432
C	5.13879066995797	17.51951232258609	19.43191274295607
H	-11.04076770071856	8.81648603877600	19.73975835033608
H	-0.21469021554954	27.25560211306701	19.87906991478340
H	11.01932576120323	8.33990045327022	19.90946891806002
H	-11.58623252718724	10.50355062502857	19.82691533179436
H	1.55695515900959	27.10842709822278	19.83524781426307
H	9.88100487991517	6.97141095045719	19.85862057385208
N	-9.51274705300875	10.27367762021560	19.62413152671275
N	0.53041791400432	25.28901156483544	19.62929988521420
N	8.97427524508548	8.85815533637073	19.74686134762665
H	-0.33822016600167	7.90160896451221	20.24463064739729
H	-5.43844699677713	18.14684196828637	20.02700293326555
H	6.10767868994939	17.59702264316032	19.92951805352637
H	2.14458831017478	8.22022978949625	20.29044350528591
H	-6.43820504312624	15.85293484361084	19.93089738377532
H	4.59493585829177	19.58177402104041	19.82033387811041
H	-4.77712610252742	11.03787078665186	19.77895632898187
H	-0.48305251492996	20.61633393554326	19.59031266340807
H	5.68922406271443	12.32895205525089	19.86422134790880
C	-6.88583995895759	11.13341896100430	20.17093405286206
C	0.33908371441590	22.52078918348656	20.11910222056196
C	6.79461851031426	10.54869745186563	20.30783678297363
C	-9.28745316287116	11.23782821835519	20.55551747047209
C	1.17596422780498	24.73316312091538	20.68935570220215
C	8.10059356287881	8.54238182005145	20.74003708942741
C	-5.52275274159156	11.55939174484555	20.38342141757855
C	0.18836451787513	21.09698991985094	20.30527156444767
C	5.68159687705250	11.44572439620291	20.50685882963016
H	-2.75863064106019	11.53159915040584	20.21704166609719
H	-1.17160358765414	18.74857799700989	20.15967388372404
H	4.34220224219632	13.81907894304456	20.34767252496869
H	-10.16567861593217	11.64289021134467	21.06019560370385
H	1.75015499684456	25.41423869197342	21.32000790863572
H	8.29605990347542	7.61679056285236	21.28412939597599
C	-8.01307394292328	11.67584572769432	20.84447014357424
C	1.09959828101162	23.38135647133503	20.95450507074566
C	7.02612539423751	9.35368117747495	21.03982196606723
H	-0.52831774067088	12.47079714748134	20.50589821487853
H	-1.45511660783378	16.34335948599508	20.44186624698676
H	2.40024695001169	15.26537904686551	20.56561200458933
C	-2.64254557006711	12.42026098462502	20.83511093006650
C	-0.47309041966200	18.21009108030407	20.79725278075847
C	3.49421014824958	13.46401609886897	20.93041687507321
H	-7.90253856110965	12.44794721927752	21.60638275535872
H	1.63107207444443	23.00268153963243	21.82808250813952
H	6.36712357478422	9.04459372256994	21.85179993193867
C	-1.37376367822243	12.94734835669955	20.99454573004128
C	-0.64065902303167	16.84694453732130	20.95557487642338
C	2.39379046898962	14.29241839839808	21.05133716033014
C	-5.12200114667798	12.54982841633242	21.23282095317381
C	0.79262199264874	20.32549978158836	21.25382014059410
C	4.64269040663901	11.27531591081631	21.37198522108540
C	-3.76966737615891	13.02574093351550	21.43598633127187
C	0.57814453219671	18.90270339530727	21.43916558028794
C	3.52669662622270	12.18686355961768	21.53449660340593
H	-5.88077521001145	13.08328268818389	21.81483035137779
H	1.51480135759805	20.78300890039601	21.93856347640780
H	4.60104494476865	10.37110781563768	21.98861866352745
C	-1.17117076076213	14.11198116022847	21.76298310679429
C	0.24548097276790	16.10514974825536	21.76235871204555
C	1.26501091225523	13.87879646963454	21.78817564679382
N	0.11321615374050	14.69775017897027	21.84964972504762
C	-3.54710673425245	14.16616509107539	22.24209212761582
C	1.43249957589427	18.15654121140190	22.28281087055222
C	2.40579895818779	11.80011515444128	22.30278412358766
C	-2.27606303331295	14.70072313216339	22.40755074613786
C	1.27403080108965	16.78457082425653	22.44356406887454
C	1.29638533815922	12.62635310040722	22.43130497108374
H	-4.39151879335651	14.64150439233375	22.74636911183762
H	2.23498587745649	18.66636603436072	22.82169142484408

H	2.40723775790607	10.83158399122279	22.80762939289938	H	1.94957751020050	16.23185124115466	23.09777021578996
H	-2.13189467442593	15.58213846747716	23.03293102676570	H	0.44582115299005	12.30114342905395	23.03111930718762

(h) 3Me-HBC⊂TPACage^{6+}

H	-4.32954388861127	14.11339966904441	11.72116543149616	C	-1.37422677957205	14.06170468506186	13.42235211315163
H	1.47858836183039	18.83975153362542	12.10670389111342	C	-0.19053725996796	16.19423876478786	13.46728190927170
H	2.52817137051220	11.23267584435898	12.43069718377257	C	1.06779821835849	14.08653508894986	13.59207749796290
H	-8.56095096566003	12.82622319840810	11.42667950813165	N	-0.15784613830264	14.78171378140829	13.51769930835665
H	1.76090905274577	23.29489947878258	11.36761277687692	H	-5.02699440142967	10.96373258323114	13.83542023769341
H	6.02633524748176	8.64962441467754	11.81480810958306	H	-1.22206133237778	20.84998326119005	13.98336194322810
H	-2.21953159917403	15.35009289460915	11.89561445321096	H	5.60972879754041	12.61219474882964	14.26990020113845
H	1.56881033314532	16.39040678106143	12.21275489327894	C	-7.55832849365907	10.20860957594778	13.37404090186964
H	0.39947515356089	12.42933329232272	12.36118260471838	C	-0.62736176303262	23.42516173796281	13.80542522709914
C	-8.59699965639688	11.92728688094494	12.04426574377909	C	7.62799775463295	10.87971666539832	13.84271543758928
C	1.07664620994079	23.68683809040957	12.12274776173649	C	-8.80178111671998	9.65826106165634	13.56652923051088
C	6.80077296946032	9.11875919075654	12.42410422566987	C	-0.61839476822218	24.78784456868210	13.97524943019488
H	-10.75140852798579	11.74097502492322	11.87306402429379	C	8.82872593590447	10.21840736089026	13.90045308447196
H	1.68533607257533	25.74320400267050	11.78930993812894	C	-11.25460490081012	9.70811446676280	13.49707418564026
H	8.23966646897025	7.54257829593691	12.03110191652631	C	0.29036442710348	27.04639874649175	13.61900949985375
C	-3.57362275556657	13.76990886061427	12.43022119611200	C	10.27845994394500	8.26948446749418	13.55543630539092
C	0.71713919373598	18.30751298701130	12.67968208663108	C	-2.76724202210881	12.20034391294283	14.08568106928628
C	2.44049962141511	12.17853860091414	12.96748794656898	C	-1.28481038686867	18.27535401547677	14.03446366093592
C	-2.38321368841548	14.47492790698080	12.52513187430536	C	3.37310056965899	13.94700703634520	14.32280697879090
C	0.77642476319825	16.92257107548097	12.74021426189662	H	-11.37124165998875	8.68329165897325	13.11773026985692
C	1.22985533085257	12.84993392468947	12.92786906556290	H	-0.64871274920405	27.52339051066566	13.30324279751693
C	-9.82288153306410	11.33659276052234	12.27939017562947	H	11.13182846520242	8.86884737131233	13.20747325357285
C	1.04718615445653	25.05109445849306	12.34119190692533	H	-8.95259713507144	8.76402539819401	14.17095929383827
C	8.02632679913606	8.49036840482217	12.52772834938700	H	-1.25422309080800	25.28733268327353	14.70629100618731
H	-6.21703172863392	13.04139768056823	11.88773518444702	H	9.66971187646858	10.59450154817216	14.48256068443592
H	1.15205919246030	21.04281637552871	12.02327648258069	C	-1.58456722770495	12.91584661354887	14.20711291220765
H	4.49453293370365	10.38961848608995	12.44397282730469	C	-1.22102371615422	16.89308420953754	14.12159285873416
C	-7.41558850914025	11.41451634501122	12.63570663450029	C	2.15664905142425	14.61629516501643	14.31003371798404
C	0.25346686565916	22.81090751309772	12.87352477285267	H	-6.70896836285212	9.72261774952592	13.85124058201109
C	6.54084906016799	10.33235633653071	13.10949760383374	H	-1.27744877928367	22.83373418879300	14.44794494036746
C	-6.17664494151149	12.15082070336050	12.52007188087287	H	7.52606770333217	11.79769640980257	14.41719098541702
C	0.36690556930835	21.37877895800748	12.70536704622081	H	-2.91437402444233	11.31628797119511	14.70470993056236
C	5.22592156999503	10.92889001775591	13.05149518700956	H	-2.08682286929480	18.80257512251899	14.54946745327202
N	-9.92616781160312	10.23127669482817	13.05650174078846	H	4.20860579737793	14.37644533444485	14.87572872295445
N	0.22275800130145	25.59325745070669	13.26991845659203	H	-0.82319278476716	12.59641602530604	14.91864012588010
N	9.02400302399140	9.02642570152530	13.27163932098887	H	-1.96204432387957	16.34625136740592	14.70504417384240
H	-12.02266709224175	10.33376305422897	13.02433662514401	H	2.04386034061478	15.55138597366362	14.85781720890012
H	1.10619568168771	27.48226923945458	13.02724967568310	C	-11.31856068967817	9.75518936709453	15.00760950160295
H	10.24790424026833	7.34792742688706	12.95981854297462	C	0.51625179106528	27.19105858481333	15.10712594651431
C	-5.03185364697245	11.85012750880002	13.19430742376616	C	10.35746594402366	7.99585476560130	15.04256304558521
C	-0.41801507528961	20.46945413419833	13.34768675881070	H	-11.27778287205009	11.92430732869594	15.09427463919782
C	4.85820263038979	12.08124561926395	13.68007128282066	H	2.50828422207813	26.33243179833027	15.04174912205112
C	-3.79792981901912	12.61269240809103	13.21348996513173	H	8.56433595503922	6.77096091166788	15.06968471510856
C	-0.31688600059878	19.02295231124481	13.32848044581866	H	-7.07137554538062	12.85965305260719	15.45260595024980
C	3.55508082839268	12.71666964654188	13.65293234338441	H	2.20503669381096	21.20767580776445	15.22171418222131

H	4.86497143073931	9.13577850641841	15.72371269679893	H	-2.21147617162963	9.52781851804125	17.06506350573470
H	-11.43098061420194	7.60876000165814	15.26604546025863	H	-3.04319675903227	19.02922826389863	17.14324902405628
H	-1.39151521659921	28.13628816861178	15.50646622527726	H	5.52459794834319	14.90419979455636	17.16500177680436
H	12.19065320749279	9.10797686472692	15.34742080269392	C	-2.89410429851170	10.37214346186756	17.09565932708649
H	-4.57649469829080	7.05158198080932	15.85112520990805	C	-1.96553750963899	19.16066917026604	17.15015017461651
H	-3.95928518897618	22.38450164649422	16.17684974236478	C	5.12186521536903	13.90206870822504	17.27143722065661
H	8.85891902109861	14.04507912114321	16.14151495003635	H	-3.54285107460081	7.58860951920636	17.21659997450261
C	-11.24751113901540	10.99391409363973	15.66821707653271	H	-3.90342790437243	21.19802379079918	17.52324277612973
C	1.69797616175909	26.69174468783406	15.68157250129511	H	7.86337206420419	14.65782229533240	17.50406113903676
C	9.33932029123083	7.25584386397201	15.66983111668289	C	-11.22925236285238	8.63200737815406	17.15737057488524
C	-11.33871659310783	8.57849568088145	15.76246521677641	C	-0.34636772623158	27.63969545799561	17.33153713668091
C	-0.48452883532156	27.70517974861940	15.93930465333342	C	11.33089759504899	8.45345193005557	17.21855747703959
C	11.37149078814404	8.56106000526713	15.82251742927510	H	-11.07037957301910	12.01711621481990	17.55431874807774
H	-3.08807466601568	8.00021693104284	15.52816271042772	H	2.74927330638629	26.21300664725737	17.50002540541534
H	-3.99877233443937	20.63147160368200	15.82069139925788	H	8.48535435781352	6.59550185702095	17.53400559330768
H	7.31031697243324	14.88257426629710	15.80942208912483	C	-3.26232593794099	12.77706145680497	17.39535867976219
O	-4.74938711278861	9.02314833154428	16.28253169467299	C	0.27102963461398	18.21603952206696	17.39605178105823
O	-2.16518023561417	21.46416628845974	16.39269125260980	C	3.21379216668200	12.43086215160348	17.67900637777527
O	7.21686104191490	12.92034807910165	16.53333084491890	C	-2.39112368836006	11.65042122721545	17.47328577273037
C	-3.93394416345936	7.85985864867191	16.22146424963682	C	-1.14023902105143	18.04812287991546	17.47894283753808
C	-3.58173004528949	21.40297645951234	16.48852232729347	C	3.75835407683775	13.74682769665029	17.64716576744601
C	7.83386877505231	14.20147847332401	16.50006837173221	C	-11.09246054643172	9.86252191486350	17.80879392863951
O	-6.33685112286758	11.04959283144869	16.19352064151420	C	0.79278612706883	27.05936119936630	17.90159186551387
O	0.41315302514164	21.71752155387244	16.16153741447112	C	10.27140493451996	7.78688860620313	17.84555214225422
O	6.19992925787592	10.52066996069505	16.53628290668808	H	-11.24112936504750	7.70252940920356	17.73324114378133
C	-7.30211369876360	12.08191409284020	16.19963596948130	H	-1.14949163538027	28.01747129514443	17.96985982710541
C	1.80267784381798	21.90714571809227	15.97281553539930	H	12.11940543626017	8.91855051578662	17.81647953055750
C	5.70171430102421	9.19984031883838	16.43961687139144	C	-0.47537960462553	13.15196575001559	17.77241358690636
H	-8.26017348158518	11.60855054297350	15.94766016382128	C	-1.35878171874969	14.28844672967170	17.71136562786464
H	1.92717343716262	22.93457861669618	15.60788131297834	C	-0.81446672450651	15.62375940866654	17.72027596904179
H	6.53429682245826	8.58174124367057	16.08054561464962	C	0.61394230009453	15.81900438043590	17.73828060638060
C	-4.20592349288551	10.19022811580383	16.69017732380226	C	1.50086561718933	14.68494998357103	17.80357786935300
C	-1.43573401434392	20.37582796153872	16.75273886416810	C	0.95273451792604	13.35456488356352	17.83796308706977
C	5.92084681709394	12.82540608175618	16.93641389437235	C	-1.00881802568790	11.84859864822201	17.85534994217111
C	-5.07834256635921	11.31974502119915	16.62166608840980	C	-1.67585204103665	16.74000016429692	17.79833198948514
C	-0.02231175079502	20.51827996159923	16.61742141342513	C	2.89544348020873	14.88007633140045	17.89740328500334
C	5.36612524478286	11.50884149297742	16.94077314053715	C	1.81258983909076	12.25011539422253	17.98603315211292
C	-11.13221762097118	11.04686961644781	17.05343703596177	C	-2.75547014303771	14.09245525142672	17.72554301485699
C	1.83548322288195	26.62681940669505	17.06443687482448	C	1.14182657016000	17.12249033088862	17.75565926935122
C	9.29469017114171	7.15505246624742	17.05675560297501	C	1.24699443208380	11.01477287305448	18.49569973813102
H	-5.22412217995992	13.44091002596645	16.76467467589560	C	-3.59138320129008	15.19427766289852	18.16741057014952
H	1.85975896219739	19.52228142099561	16.73668473263163	C	2.50146760872470	17.31463910977108	18.22190316197088
H	3.60082214237977	10.34749594345856	17.25227317515949	C	-0.16705745883554	10.80739255404766	18.41933285177028
H	-7.38106500727116	12.55101693119857	17.19497781187857	C	-3.03747271817458	16.51069926203770	18.24215437671655
H	2.35688255969554	21.78422645452884	16.91853499117274	C	3.37990599777942	16.18995816931035	18.29520364362301
H	5.36419895940017	8.82069239579757	17.41964309048950	H	-8.65456437624696	9.02002129972666	18.22843196449644
C	-4.59300971915620	12.57373637593825	16.93293963463635	H	-0.55145845126460	25.04118544247355	18.03755417186791
C	0.79167817909298	19.44564785244358	16.91547156747981	H	9.52604230259788	10.13564421219198	18.23162597758548
C	4.04384943993092	11.33804486994793	17.30536458915177	C	2.03316835208719	10.09436263759660	19.23080318254749

C	-4.89382973276699	14.98363227256245	18.68848201621470	C	4.59807543675931	20.06435067748634	19.90735630565011
C	2.93421616892921	18.55203804299970	18.76697848129594	H	3.07285857361680	8.64064606927248	21.31191275814071
H	-6.30821614926039	9.68563865177913	18.64283541568352	H	-7.33568022189786	14.67719659064539	19.63135518310122
H	-0.80549631779161	22.60832052942654	18.39124266116686	H	3.75984098652080	20.77773716114453	19.90018429274946
H	7.68022728496230	11.69677296977687	18.71484991546184	C	-6.81231282642690	11.21722996819376	20.12369022050527
C	-0.69951673655598	9.66993983292117	19.08051417248378	C	0.49414470976027	22.54534839523880	20.14921384741801
C	-3.80228952176170	17.51402315143442	18.89504075156583	C	6.82711213130645	10.48233339484244	20.32250180226058
C	4.63949529141403	16.38300645042799	18.92544185093603	C	-9.21270284564181	11.36213596596741	20.50298607058216
H	3.09080414312346	10.30973076426907	19.37326045571291	C	1.38875347270659	24.73966170758061	20.70101952145211
H	-5.29238113402215	13.97404670865648	18.71109138484205	C	8.10572902203229	8.44865742092824	20.70360022294904
H	2.23577416092620	19.38465111013291	18.80659913747939	C	-5.44565343797250	11.62843167286138	20.33263824369759
H	-1.77703659650606	9.51477321438523	19.10727197487706	C	0.32448244934517	21.12444161594456	20.33271481187534
H	-3.35775834303685	18.49013226690169	19.07866792525103	C	5.73008581662913	11.39238842251483	20.54561486927169
H	5.29313843961726	15.52913253779195	19.08952082369926	H	-2.67851378401670	11.58079373318093	20.17968474063481
C	-8.40334385983032	9.79495567609639	18.95245697902989	H	-1.00746931901298	18.77601412820130	20.12009105943888
C	-0.04992680948436	24.53389682709195	18.85917822171967	H	4.44774101834504	13.78240600963221	20.40445495167161
C	8.80900304399583	9.93244607849674	19.02582027029177	H	-10.08590036689151	11.78712854627447	20.99990469797884
C	-10.84583779550025	9.92809593575635	19.29606459995956	H	1.99096359779960	25.40887005085428	21.31818651363248
C	0.86296557499851	26.78134646192967	19.38121323180576	H	8.29341443821843	7.51030163680498	21.22821936941806
C	10.09691822289393	7.84344985299489	19.34234852501763	C	-7.93326430032034	11.78960139295830	20.78384762033861
C	-7.10570645831566	10.18171214016688	19.19519744352612	C	1.28963031219986	23.39010679374513	20.96875769471793
C	-0.18356876342141	23.18289615322103	19.07740371048406	C	7.04833501837320	9.27150586217327	21.03122265014902
C	7.77334290959844	10.78986475625617	19.31088915414083	H	-0.43709753482658	12.48424600932567	20.50231267529902
C	1.49421613157964	8.98742815505561	19.87587944950783	H	-1.30088968088380	16.36884340108066	20.38165934804206
C	-5.65455892133923	15.99059801048987	19.26253006848788	H	2.52569688619475	15.25403689190689	20.59797926500285
C	4.18236913332656	18.73155354030081	19.34321188093694	H	1.76764507949437	7.43063189205286	21.36367425987216
C	0.10087471106207	8.78555498910888	19.78230998939947	H	-7.12356281003093	15.95850445054260	20.86148246758994
C	-5.07279027107076	17.27223570548312	19.38029271081873	H	4.96985906254553	19.97320211640735	20.94122054724934
C	5.03818283592046	17.60985885220894	19.41991453033719	C	-2.55887082666669	12.47422464233382	20.78979457257594
H	-10.99116058241165	8.94592673243199	19.76938292750031	C	-0.32843566279492	18.23304258483065	20.77481536754128
H	0.06021253466580	27.29858745423402	19.92733965784070	C	3.58894521879489	13.43926729484261	20.97894728557997
H	11.00687765245927	8.20523055601048	19.84409967526631	H	-7.81199400864967	12.57228811029406	21.53276355044683
H	2.95660560587422	7.39701752330268	20.04755682709503	H	1.83348774894458	22.99996972150513	21.82970239439504
H	-7.78201884356297	16.36030388185808	19.27320892144190	H	6.39258358859662	8.95793434012890	21.84402949302357
H	5.42124134642429	20.50227739019092	19.31835087500240	C	-1.28407721426394	12.98050191919628	20.96944787427872
H	-11.52082409658542	10.64032813567756	19.78823276396412	C	-0.50192197640723	16.86989563344962	20.92194878436101
H	1.82321919582665	27.09604049975395	19.81138945675854	C	2.49895528657866	14.28234228934240	21.08447539513123
H	9.84070217080484	6.86151899905424	19.76226899048472	C	-5.04202829362224	12.64654246803145	21.14694623326718
N	-9.45005052558674	10.38167907930845	19.59159430167523	C	0.92861292424107	20.34226671774325	21.27267875418686
N	0.73322287912255	25.30864208606999	19.65376281548041	C	4.69940842908769	11.23555194657190	21.42285800955560
N	8.97179897711712	8.76799549618960	19.70511016390991	C	-3.68729750570929	13.10729234513013	21.35871357385679
H	-0.35931387009080	7.94857912491925	20.31346956956704	C	0.70834524231574	18.91996394685980	21.44561199446460
H	-5.61731201956339	18.06952421101473	19.89259310484374	C	3.59641890130248	12.16270319912476	21.58523031436545
H	6.00557476729717	17.70200536001549	19.91996284536672	H	-5.80153445498452	13.21504606267997	21.69312263163855
H	-4.70185400534822	11.07429047718241	19.75599855708039	H	1.65702305186449	20.78980463395185	21.95680058823035
H	-0.36075830106107	20.65539980725206	19.62316690321660	H	4.65292886827890	10.33281005436178	22.04126640714133
H	5.73859079344751	12.27595970962908	19.90402744389581	C	-1.07850774380519	14.14988703227399	21.72940407811209
C	2.36385377497282	8.06689629972474	20.69337872837549	C	0.36149027347169	16.12282440498326	21.74869153821192
C	-7.04146594897373	15.72612794507510	19.78620867465608	C	1.35384263083021	13.88379735766215	21.80312276300541

N	0.21203950282139	14.71734285680076	21.83869804949592	H	-4.30953717734489	14.75081605009455	22.63347885167543
C	-3.46280172119759	14.25504609854900	22.15347817130154	H	2.33800363795920	18.67271192182761	22.85797928168434
C	1.54560034290414	18.16731105118679	22.30023363644992	H	2.44222607281061	10.82539834624932	22.84785449869331
C	2.46103399079285	11.79284281253248	22.34091332457837	H	-2.03907899994798	15.65143751480718	22.96172656675943
C	-2.18631446762634	14.76640282633596	22.34236411860982	H	2.03921856752084	16.23730745366770	23.11787433048746
C	1.37968240894601	16.79522266532765	22.45186932974076	H	0.49607378492894	12.31920570243278	23.03755042139414
C	1.35976237378436	12.63314189170486	22.45058859522346				

(i) 3F-HBC⊂TPACage^{6+}

H	2.67141827717903	11.17337586220809	12.39740906450009	C	5.01468660212173	12.09906009884350	13.58006240675104
H	1.55919740048124	18.75958225093133	12.00624395441535	C	-0.33501105665387	20.39269209513050	13.25692534021676
H	-4.25702252346178	14.04532907116019	11.74715418747879	C	-4.99699700389887	11.86991482085059	13.32605779748003
H	6.21541810906527	8.60421384298980	11.86543048079074	C	3.69593619561712	12.70368574787493	13.56316819834359
H	1.90487759117815	23.24289225555668	11.37795949820111	C	-0.22736112041772	18.94617951359238	13.24082977417046
H	-8.47174675093723	12.86677942412982	11.46913802716566	C	-3.73779849320566	12.59201637606353	13.29040242924687
H	0.51486290504080	12.32335765742972	12.35694305734850	C	1.17859830103614	14.02074772994587	13.53431088926677
H	1.65364335724966	16.31186704409771	12.12252645323346	C	-0.09295927141254	16.11699232088120	13.39521385651118
H	-2.11527308569114	15.23292451118551	11.83568162587209	C	-1.27276353066961	13.98457646795640	13.39609890503389
C	6.98557402776899	9.10582028853094	12.45402795627520	N	-0.05324633321295	14.70439879271931	13.45982838512965
C	1.18071363794834	23.62534235913918	12.10012084268129	H	5.76465398714828	12.66661067813323	14.13697424005243
C	-8.55019249350300	12.00378715868193	12.13264610054234	H	-1.15255640637165	20.76725072052075	13.87858647400969
H	8.42687567376680	7.51047031533205	12.15945305591097	H	-5.01540642002017	11.01898278517407	14.01446227930003
H	1.80234176166250	25.68633528926274	11.82252428081787	C	7.80504694149500	10.94372350365808	13.77547134846470
H	-10.70305597986640	11.86597657476741	11.90756881885827	C	-0.60819041867513	23.34399593776969	13.68793397138890
C	2.57630668010225	12.12765642060088	12.91806108668403	C	-7.59740867235509	10.33282286866448	13.58211592005532
C	0.80369188586676	18.22925626947579	12.58894982226169	C	9.00289522331623	10.28232631124572	13.88692153922569
C	-3.49650378665083	13.70822716307966	12.45412965494670	C	-0.61152852100924	24.70443075427264	13.87335130969692
C	1.34939620553749	12.77232545692353	12.89558734620744	C	-8.86192461674814	9.82842818675198	13.76930094611680
C	0.86497324733411	16.84443925358488	12.65487709355751	C	10.44963938108615	8.31113973966700	13.65961981998427
C	-2.28754206545309	14.38492462382561	12.49931160827565	C	0.31833880515294	26.96391205377610	13.59951753520248
C	8.20938807457492	8.48314626772110	12.60304462152275	C	-11.31214350301705	9.91177744474328	13.58579700227550
C	1.13727761368919	24.98730655538452	12.33227954575135	C	3.50180440500448	13.94021145875555	14.21732210739846
C	-9.79842987093218	11.45987284312430	12.36285587080594	C	-1.19025383405463	18.19903412922243	13.95362839159330
H	4.67139337450485	10.35779409723972	12.40942611671006	C	-2.69829508184284	12.18320759153167	14.15394529300459
H	1.26428353608791	20.98053900208345	11.97449483583642	H	11.30807593112606	8.90941419783175	13.32319101637457
H	-6.14058794597974	13.02869210372215	11.95827980235122	H	-0.61532598604979	27.44249817295981	13.27061754894653
C	6.72133012605982	10.35457522052796	13.07007437377659	H	-11.44143499669634	8.89152504980528	13.19749198060404
C	0.32447852587649	22.74036569059649	12.80092450568555	H	9.83811990097021	10.68765183337797	14.45821635818703
C	-7.39898746995585	11.48808976122344	12.77874606539971	H	-1.28605418509589	25.19448640130764	14.57585932562097
C	5.39869463012881	10.93381963998864	12.98765629562881	H	-9.05552251462536	8.97301934821755	14.41640518911040
C	0.45643060164322	21.30887063997245	12.63346750555300	C	2.27143051688585	14.58246694796098	14.22201612025881
C	-6.13319957041834	12.17338841290543	12.63879247346356	C	-1.12139652841332	16.81765009693821	14.05072381720257
N	9.19960414223313	9.05622992939492	13.32869474799564	C	-1.49216806183515	12.86889572136699	14.22118103986969
N	0.26528997062613	25.51770123739314	13.22351612359019	H	7.70311760593178	11.89588182664886	14.29148657196894
N	-9.95466871582940	10.40048537321333	13.19347064185162	H	-1.29312542327176	22.74653304078970	14.28605828595455
H	10.43923518968118	7.37983256976003	13.07900265866993	H	-6.77475637195530	9.85051384057058	14.10836412289865
H	1.14569012072008	27.41395706421383	13.03498586149117	H	4.34120557912047	14.39686067427035	14.74239556537194
H	-12.04433975488460	10.55842533095361	13.08496438584009	H	-1.99625270278916	18.72682900816313	14.46139653852397

H	-2.85742392409272	11.32684510188596	14.80856775683345	C	9.40765914336164	7.20279237731238	17.14695360664684
H	2.15136566703284	15.52525045044500	14.75458490770568	C	1.79097884253950	26.49618672277213	17.07164733763170
H	-1.85859426763531	16.27287898047116	14.64087053526470	C	-11.32171794138749	11.22800544525541	17.15401602626057
H	-0.72217289525536	12.55344115167726	14.92535018325346	H	3.79397777520514	10.49643365356977	16.90948768120041
C	10.49242950153868	8.06176071694596	15.15206168126024	H	1.90291865330214	19.60121421534924	16.77547985252193
C	0.51008359251316	27.08198479052654	15.09500825394566	H	-5.16097283119367	13.42128072892061	16.96667243599860
C	-11.44321898733969	9.95452310311844	15.09143227855823	H	5.62011290125764	9.06130817822182	17.33832015409844
H	8.74248157761611	6.77511728281849	15.14642331199340	H	2.34034151080958	21.88299096095303	16.91411451833652
H	2.50399471465794	26.22540022043422	15.05940297856260	H	-7.28796535519537	12.54484201669685	17.45836116772067
H	-11.31175889833633	12.11948958997652	15.19541367216427	C	4.20880140259586	11.48839910972154	17.05654982529537
H	5.19588766179836	9.23809576567475	15.60015813417815	C	0.82912715261410	19.50038207632342	16.90613158944218
H	2.26063450683578	21.26777937881638	15.22540393728578	C	-4.51521617021430	12.55910824834126	17.10353340104566
H	-7.02967381150785	12.82412250192357	15.70167783250141	H	5.61573683991035	15.08472401539055	17.15309231692101
H	12.27929377653494	9.23683385890231	15.49226914221954	H	-3.00652752872092	19.00136343800370	16.99144695793818
H	-1.40633298180788	28.02072413790719	15.46686606875475	H	-2.10749418796389	9.53146104686869	17.13002053361990
H	-11.66155685368508	7.81329727163780	15.32820791338102	C	5.23508550326433	14.06875963172135	17.19763531148589
H	8.99294396000032	14.32889675149281	16.13895216119054	C	-1.93225671024206	19.15676580209430	17.03363779689439
H	-3.95325354333589	22.30396850471172	15.88743516690647	C	-2.79186721390729	10.37224752128107	17.19296064400608
H	-4.51092643711705	7.03521982298673	16.04039701504080	H	7.96419467445981	14.87147114375154	17.50650465649339
C	9.48353904711322	7.29392541708637	15.76064089612142	H	-3.92987668679644	21.15758440673504	17.26855936069255
C	1.68008897445165	26.57543532914487	15.68705248918470	H	-3.42868904122573	7.57975293547301	17.36459305236907
C	-11.35691673994538	11.18609394806241	15.76381442434901	C	11.39240749827555	8.57326707495997	17.34756691369332
C	11.46543820113237	8.66931209399257	15.95207527295299	C	-0.39611800797386	27.50616326730383	17.30675934177577
C	-0.50810340520331	27.58451274647506	15.91279939727948	C	-11.52707579525777	8.81855046882449	17.23497305423231
C	-11.55684875576764	8.77601233950087	15.83557033023670	H	8.60689691280421	6.61818452598423	17.60878131224786
H	7.43351978424920	15.14736662960189	15.81148589785190	H	2.69741291928559	26.08039874409555	17.52049990872704
H	-3.93309881636205	20.54080555781355	15.58051300789520	H	-11.24774794516867	12.19279043336267	17.66403898198314
H	-3.03850233951491	7.98579325191988	15.65720532676525	C	3.34400505476739	12.53917268223432	17.46874885024504
O	7.36716249167140	13.15936270848397	16.46231917367933	C	0.31352776657603	18.26121339233092	17.36806608410911
O	-2.14879861140397	21.43495662187171	16.21050206001718	C	-3.16399568883878	12.77446785331258	17.49206254888366
O	-4.67240791466683	9.00763225130139	16.47161295791692	C	3.86631952052190	13.86341847383022	17.53187661393446
C	7.95962631754802	14.45229313715128	16.48597224154755	C	-1.09577078858908	18.06827064852642	17.40922268819214
C	-3.56677294568270	21.34138227938625	16.24371483272067	C	-2.27922554185315	11.65706853797532	17.53602386552883
C	-3.85663666521774	7.84602737518899	16.38334615430853	C	10.34097537558389	7.87575473095664	17.95366360053022
O	6.41795434700348	10.73998750748163	16.38769514493873	C	0.73313224051648	26.92217952987672	17.89285811791494
O	0.42517584007634	21.74750138561600	16.09336524611397	C	-11.37720936671878	10.03923656972843	17.90188470987785
O	-6.27852555929351	11.02275219043848	16.44826672889118	H	12.15025998550486	9.06870841185262	17.96057331980723
C	5.98805113578293	9.39175493391947	16.35174010964348	H	-1.21075261874948	27.87902617678335	17.93338307773683
C	1.81542675864461	21.97115167109036	15.94825419658195	H	-11.61165913130826	7.88788616285233	17.80266247613754
C	-7.24077345488590	12.06014710346785	16.46869071847411	C	1.06297230919903	13.40470669855708	17.71931900021725
H	6.86669810454980	8.79488495885083	16.07655613052507	C	1.58648627555453	14.74441480304948	17.72456560836405
H	1.92448624637308	22.99457294103460	15.56776370085768	C	0.68158947816369	15.86491511047533	17.69243984542434
H	-8.20748229240259	11.58690819360229	16.25258035322292	C	-0.74233688972635	15.65304482026908	17.68373243038817
C	6.06928950221748	13.02632046333020	16.83521492979480	C	-1.26575304525861	14.30971665564014	17.70744898545853
C	-1.41232340335898	20.37333078794057	16.62841087729288	C	-0.36478234713120	13.18425421446487	17.72840648281575
C	-4.12045703832894	10.17711387682647	16.85120065404706	C	2.97650404126378	14.96111629020465	17.83343388437471
C	5.54328646869220	11.69901709483991	16.77138234342999	C	-1.61542833600892	16.76002421287713	17.74718522326507
C	0.00316547882993	20.54776904606307	16.55335522473710	C	-0.87974306513611	11.87427081232984	17.83873495574125
C	-5.00478617435341	11.30053431371347	16.81953407369899	C	1.18786069208328	17.17503715154302	17.73495019923558

C	-2.65658226882024	14.09637981891852	17.77854635780243	N	-9.79441815708328	10.47572127291574	19.74902915351731
C	1.94879160087815	12.31209686246467	17.77688106058508	H	5.90744166293673	17.73198153286148	20.21012821158673
C	2.52802872119516	17.37056583820572	18.24765189094797	H	-5.47467246929376	18.08290929639813	20.02218606893246
C	-3.48819916832957	15.19578968523422	18.23632371713865	H	0.01775574628081	7.86821919915143	20.09127603567994
C	1.42884144163459	11.04495388898084	18.25166338759793	H	5.65621087982039	12.27123837178629	19.88321470667000
C	3.42363833348243	16.25726898591682	18.31537408112110	H	-0.55239615394716	20.53489290467123	19.63581854705222
C	-2.96281797954612	16.52800678230806	18.23260460282782	H	-5.04855620696044	11.06013617429982	19.91212048249462
C	0.01421454424780	10.83156341080346	18.31251777070954	F	4.37841562522579	19.84594493415714	20.16677094287060
H	9.63240735252148	10.28858979335103	18.45739663515888	F	-6.58330163053756	15.72781837657642	20.08807331280283
H	-0.66037323478700	24.92251734342669	18.03022799749727	F	2.63001944741389	8.09370886759906	20.00267982291666
H	-9.02029148331511	9.07113533476449	18.41826424013765	C	6.69788462263116	10.43514305065745	20.23198616855245
C	2.89995089497514	18.59005349914012	18.86373656677132	C	0.36346636859218	22.40675602218628	20.12933867937315
C	-4.74033964845641	14.96719855492553	18.85871524258645	C	-7.14673585482410	11.24773754155710	20.30404097242385
C	2.28340905442077	10.07724291109691	18.82789101056082	C	7.97053317956675	8.39716571272806	20.60916962791784
H	7.69813431913652	11.77901758324152	18.82567087085938	C	1.29807756798979	24.58492140601317	20.67659658255995
H	-0.95506022510327	22.49635198371877	18.38598459944614	C	-9.54398550527650	11.45904082438211	20.65318223042607
H	-6.65936939221645	9.68930771835240	18.84810622464410	C	5.57773363715322	11.32404748328632	20.41995080556160
C	4.64238933930258	16.42662623161338	19.02706558284282	C	0.16665839734235	20.98949220437062	20.32084343413591
C	-3.70858348925975	17.54644428864504	18.88479407161251	C	-5.76910355035183	11.62844761973231	20.50545693186238
C	-0.45910523441877	9.67453564782185	18.99062112216438	H	4.22546245613426	13.68972849944793	20.27293508623364
H	2.21259805994060	19.43042950270003	18.91061577408087	H	-1.23237428828768	18.66861137680528	20.16724263694296
H	-5.14512052282632	13.96553811491703	18.96061185413339	H	-3.03857051334256	11.55666777249393	20.18375749410077
H	3.35475268825610	10.23806240847025	18.88609875778218	H	8.11587138064951	7.42424602682631	21.08127762293335
H	5.30299159058815	15.57347872735708	19.17070133138508	H	1.91555171832939	25.24316183089116	21.29039424002102
H	-3.28369062196298	18.54371539332422	18.97785036886941	H	-10.41268419694145	11.91780051850631	21.12752186488057
H	-1.52894430955598	9.53958327276096	19.14226648420040	C	6.85891593318682	9.17364239006420	20.86379526547990
C	8.83040250044633	10.01596609246044	19.14207954742031	C	1.18232726273835	23.23581910020244	20.94075495328696
C	-0.16088454411962	24.40622853070768	18.84785835076582	C	-8.25661622427359	11.85604868194988	20.94696656149786
C	-8.75674339024283	9.84911067528495	19.13454111739055	H	2.31132977469407	15.15486313079094	20.54049724122212
C	10.12517620355974	7.93957470020122	19.44583739631878	H	-1.55538048052813	16.26553488200410	20.43184883196139
C	0.77931296414318	26.63929188213855	19.37268768698178	H	-0.76998985561796	12.42449118268904	20.39480810984035
C	-11.19727095872103	10.08514162988256	19.40007162907899	C	3.36533009284003	13.31979408406834	20.82903789055760
C	7.74527573900329	10.83009517755829	19.35901071853733	C	-0.53494841624599	18.11179830870154	20.79056887293599
C	-0.31352421338142	23.05693162878297	19.06524809187291	C	-2.86594109083322	12.41660150407586	20.83015782361552
C	-7.45305419247724	10.20618098703920	19.38784046709847	H	6.11630849011328	8.78742354291451	21.56302269450029
C	4.09677998794625	18.69986248639421	19.53585146523156	H	1.72851726296822	22.83449094053594	21.79493217069456
C	-5.43038055694195	15.99723123431524	19.45912731746695	H	-8.12319362870356	12.65470908392668	21.67702674883068
C	1.77312357623950	8.95549822441901	19.43995674509917	C	2.27802797871766	14.16026563880643	20.97765815151837
C	4.98901399953381	17.62378496660562	19.63062004127375	C	-0.72409570511190	16.75012504827517	20.93716699232771
C	-4.92783739445770	17.30292543710588	19.49032935491789	C	-1.57567486055564	12.90200884836221	20.94542088049630
C	0.39404814947828	8.73595147980393	19.54635794179557	C	4.47156757671553	11.09156469300818	21.18119908960032
H	10.99920114996130	8.35770726688547	19.96649419217639	C	0.78724027263581	20.19556534371014	21.23844198614493
H	-0.03007163017449	27.15762285477584	19.90786531539748	C	-5.32193481282375	12.61716238849111	21.33138176200606
H	-11.40421307021401	9.10750891643725	19.85929971183408	C	3.36574549561061	12.01051729481652	21.36140548822792
H	9.91227911013117	6.95198305855891	19.87557824526429	C	0.54032554726362	18.77764306021147	21.42084152360350
H	1.73374449890935	26.94909637356227	19.81884602628669	C	-3.94561003633979	13.03818968976478	21.49877967402196
H	-11.85955210910980	10.82439586933370	19.86907784854165	H	4.36378692891255	10.12650387873608	21.68652541643428
N	8.94390933727914	8.80891305976597	19.75413733451977	H	1.55940938835075	20.62430119741455	21.88541614160346
N	0.63918760126104	25.16674417818001	19.63865293715163	H	-6.04942605284829	13.19493245373992	21.91063534100160

C	1.13179163567246	13.73484096173698	21.68260131876069	C	-2.35672315429533	14.63054807878417	22.45762577280171
C	0.16132338725811	15.98443216795179	21.72157020826638	H	2.19520924788571	10.60613417894513	22.52951571614263
C	-1.30164482082397	14.03023718383696	21.74490238205166	H	2.21535140279251	18.49872018234798	22.77153881892756
N	0.00319430817527	14.57745516159314	21.79047345364331	H	-4.45964951720300	14.63078348548754	22.88026462695238
C	2.22120836294793	11.60248091243937	22.08244111877054	H	0.27016272812320	12.10472644818431	22.82786423337042
C	1.39390335711419	18.00821904050496	22.24330337408934	H	1.88510917874359	16.06699167881211	23.03463610959540
C	-3.65203670439383	14.14437209112104	22.32843733713169	H	-2.15622167089255	15.48484215213077	23.10516342943538
C	1.12999428764617	12.44496130328192	22.25044547989966				
C	1.21084569775474	16.63802070584937	22.39520603811835				

(2) Optimized In MeCN

(a) TPACage^{6+}

H	13.11790515432126	10.43996968596557	12.76486385872359	H	1.90850656121366	10.58516133450869	12.35531473761269
H	13.82730351658118	6.55179735938223	12.22862321739116	C	17.74276232236155	10.42147043613188	13.44749720341258
H	10.31327437240971	7.13471146175129	13.02083710013335	C	12.91836311278744	2.38540897456628	14.12149291075919
H	15.32850200744290	11.45865740158228	12.50486250439026	C	7.56018114072633	10.76482726220440	14.00390498909948
H	14.00008640591651	4.09605551228494	12.24278302984461	C	21.21139252915700	13.74595160772044	12.72972197131891
H	8.14459418798229	8.24499295908473	13.00359503293963	C	15.08803220123863	-1.86491103534543	12.96647297943299
C	13.98162645229693	9.95877187648930	13.22328859415243	C	2.79023436680057	11.04012440228814	12.80556666371947
C	13.33698146861297	5.97778166370650	13.01607625677451	C	20.60581533739061	11.07720820298936	12.99323966924066
C	10.21827822138143	8.12060091765240	13.47512659421342	C	12.98724930101655	-0.63108771915829	14.24932939836320
C	15.23925435699066	10.53088049813169	13.07389084129985	C	4.92595905846745	12.31498543787176	13.98591337994634
C	13.43615674755574	4.59477844300379	13.03254437903688	H	20.42951475846144	10.00496086583952	13.07211925631264
C	8.98928131049150	8.75819405301469	13.46619863773551	H	12.12952339678154	-0.20269503054084	14.76718084097648
H	17.41188875080143	12.33203517116689	12.54991916752192	H	5.73188016578130	12.87255717244014	14.46262245582018
H	14.47773450293844	2.08760855958634	12.71146482601185	C	14.96911516502511	8.14018141225986	14.48702862478080
H	6.36322764611393	9.31493236572168	13.02117965210392	C	11.95886546452321	5.91259916970724	15.01382557098061
H	19.12899175602116	14.10525272132962	12.45535295989962	C	11.20106142847961	10.02140206902577	14.61544211575171
H	15.67987444628564	0.06073342055204	12.27072272926366	H	18.51255383949424	9.75568938668809	13.84906901854872
H	4.10164318774399	9.41734318863446	12.38857553932421	H	12.26093625426618	1.90852969629993	14.85472487780318
N	12.58962945471376	8.07648435273362	14.02977995086516	H	7.58692231481885	11.75958951556631	14.45920355537294
C	13.82822626991340	8.74312773575203	13.92489862117342	C	16.22047503944880	8.71043456462989	14.32506765832286
C	12.61627621913881	6.65163050482910	14.01963642250147	C	12.04253327079859	4.52240919829859	15.01301515242252
C	11.35234857297488	8.74314138401193	14.04227461463333	C	9.96426688638169	10.65711519433498	14.59343856139930
C	18.14326411665970	11.59883820452854	12.90032621157636	N	22.19372639944872	12.84878956227373	12.97863223054699
C	13.77677340248057	1.61676625884804	13.40512713289280	N	14.21082368610304	-2.59686636312248	13.68995127052337
C	6.39321893050307	10.30640681286208	13.47831305280857	N	2.62738246709535	12.26709242080591	13.35153390734273
C	19.89279685276350	13.34799184784300	12.63609894732550	C	21.90192935638638	11.52603753586883	13.06816219111393
C	14.94813561495480	-0.49547407919080	12.85824910134666	C	13.16956681045254	-1.99216735119206	14.31762602143746
C	4.01409536035267	10.40687317822080	12.83770669019408	C	3.68143375533966	12.89942623141561	13.93036575591096
C	16.39343760890937	9.91504361801504	13.61025182980941	H	23.66184909116101	14.34328046325924	13.06527304542412
C	12.80230594095046	3.83548639806733	14.04182077101178	H	15.29248504472162	-4.33383672505727	13.27647632839883
C	8.82636588215381	10.04998702062004	14.01744389972677	H	0.61303460910718	12.30577974620345	12.79323993573697
C	19.53641510512997	11.99455264244611	12.83423618417330	H	24.29160447908235	12.68096156045782	12.90531832217781
C	13.88931139760104	0.17422896904739	13.51207876045529	H	13.52930722054921	-4.56027477704199	13.37012548844694
C	5.13459062905996	11.02110958248895	13.44464995018105	H	1.31572905492390	13.90314215638274	13.15166844207370
H	21.51509336687069	14.78876938234382	12.63724425417471	H	14.86805732302801	7.20789356350389	15.03650257066647
H	15.89989010450734	-2.41176492667409	12.48722665486019	H	11.39989878946663	6.43112732839150	15.79427654037160

H	12.05277792567423	10.51864746973919	15.08012034198837	H	13.88456109233337	6.10218406753017	17.35140115690096
H	22.74236235830784	10.85198223456678	13.23230776584370	H	10.14474363063237	5.09309349988291	17.77889200913951
H	12.49622505442931	-2.64014968560045	14.87768920093250	H	11.07219409701281	9.10460620124336	17.16552550697858
H	3.48020275940669	13.88022210106509	14.36123690629666	H	23.52195239037656	11.41221356904136	19.41692061503365
H	17.09203411860928	8.21757432247479	14.76196962454165	H	13.70911823903282	-5.43154164190584	19.97668177518060
H	11.54369705022065	3.95467339681646	15.80118667586833	H	0.66452255058787	13.16530295536305	19.72597140221401
H	9.86930889927904	11.64778710384527	15.04437968061035	H	22.95360760036598	13.06702920940310	19.75612249584328
C	23.54353570177892	13.30779487440168	13.40471629153498	H	15.43400190537419	-4.98380213241747	20.07394494164207
C	14.39043149867294	-4.06676763833539	13.83926251638063	H	-0.08641316500470	11.55157113539601	19.59508991103212
C	1.26743390693844	12.85699621117244	13.47805194497675	C	21.12708358090924	10.67191879748970	18.33551765626959
C	23.60128235825416	13.17233463266742	14.90959696004269	C	12.85661305029263	-2.91637733719395	19.72538421061541
C	14.50190626124542	-4.42634166749299	15.29950084836211	C	3.11738017149577	12.15921182667810	18.92161608699226
C	0.82912550685384	12.73669096362516	14.91809926665484	N	21.47335706814909	11.75495811689855	19.07807358574301
H	24.85859717619405	11.41164882108164	14.89089489347539	N	14.10934156440627	-3.36468496370590	19.99564175107893
H	12.62121970806571	-5.49223334815483	15.36598116501019	N	1.99682126846982	11.55906783966580	19.40140796093770
H	0.94935407465962	14.85567604158480	15.32810815677372	C	15.30339338407193	7.67393339844898	17.73177715324216
H	22.33407643326579	14.89745437484850	15.26532819408066	C	11.06215679156620	3.36794168872701	18.69000784957316
H	16.41423974791807	-3.43567446044653	15.55244569286507	C	9.09643304516182	9.46348509266974	17.92965331085295
H	0.59894503778329	10.57966205125557	14.82608253437823	H	17.61477312684522	8.83374594830911	17.70801497393024
C	24.24969097884561	12.08300436586442	15.50167507295917	H	11.46718051289138	0.82426886947882	19.18223514302166
C	13.47087891253850	-5.11941345792165	15.94347075371669	H	6.81590115641450	10.72164213196350	18.23254224433006
C	0.79830332237090	13.85943121510407	15.75098556430813	C	14.05358788265795	7.07644777143216	17.81069172989134
C	22.83352577581921	14.03330844484463	15.71127387575781	C	10.93828842451027	4.73071054905670	18.43541910972827
C	15.59702433342729	-3.96647307018224	16.04883662039905	C	10.30802628930138	8.78598426397786	17.87383675297392
C	0.60492839165960	11.46370948978167	15.46894471656652	H	19.61143590800921	9.36505263852552	17.65108220975759
C	22.66060269019137	13.76440981794857	17.06572364418489	H	11.52114079399239	-1.27668548483687	19.58873082977438
C	15.62441611960054	-4.14142194524919	17.42998451615819	H	5.19838468612355	12.06398491757306	18.55442573451112
C	0.44979955768820	11.31242566205471	16.84246607560687	C	19.82357531860048	10.24138218074584	18.26265496354289
C	24.07863233738253	11.81445179306042	16.86330206596760	C	12.53864898534297	-1.58277496306067	19.82998960921363
C	13.49940396238465	-5.29704966182560	17.33041575125676	C	4.33802228666590	11.52974199415271	18.95630691762306
C	0.64329195707333	13.70679414687569	17.13277587261050	C	20.53042129492511	12.43107322228630	19.77253292843874
H	22.03105700353331	14.42229128277638	17.67087149726815	C	15.05802073804014	-2.50955486962366	20.44209259168538
H	16.46277062527860	-3.74403395260064	18.00814119018256	C	2.07511251798422	10.33331082531175	19.96601045841492
H	0.32629008134996	10.31281331380355	17.26761743523417	C	16.92050049154119	9.43970202788590	18.29812881585791
H	24.56204820550759	10.94199565167277	17.30961970782408	C	12.26290457146659	1.42279119015274	19.63665049252207
H	12.67013796038813	-5.80548774074113	17.82865707711592	C	6.81998573026697	9.80945805466482	18.83699760397458
H	0.67709921158385	14.58511897206172	17.78248946731265	H	20.87193485909981	13.29332160204192	20.34531923929985
C	23.25385633592824	12.63099093132620	17.64528047705513	H	16.03469899201243	-2.93718487188075	20.66786676317789
C	14.56009069668644	-4.78393258168822	18.08509441041398	H	1.14392279498863	9.90753223323578	20.33944420945223
C	0.51823452282560	12.43027262995001	17.68994851843229	C	18.80179882103193	10.92140420868197	18.97247396885901
C	22.88561039904546	12.22929693420455	19.05226209602717	C	13.52646418234261	-0.64198138109445	20.21209459033388
C	14.47725676133409	-4.74933175532111	19.59392334425743	C	4.45575634420312	10.22477961595234	19.49589221825432
C	0.67649149064260	12.21652116647794	19.17492796124976	C	15.55979895163150	8.92894071471019	18.32440655555711
H	16.11072437214409	7.15575712945528	17.21008024239687	C	12.10418511018790	2.86163009779764	19.49724635743882
H	10.36107051870995	2.67302710087449	18.22159855803334	C	8.07061917142087	9.06835527758506	18.81690977550746
H	8.93016109401789	10.30924418663195	17.25924387501701	C	19.20700309793443	12.03801708227574	19.73943517929122
H	21.93219840166540	10.17346714142636	17.79676517860965	C	14.79083650371505	-1.16261072994158	20.57096286195837
H	12.13345248558020	-3.66198864166806	19.39573803041316	C	3.27849682497044	9.66022154414610	20.03665696867696
H	2.99121116156423	13.14983727633176	18.48711812074933	C	17.40780092734851	10.52028395761209	18.96216043438544

C	13.32524785357559	0.79224490363893	20.20176393628201	C	12.98302119424351	3.79011256751907	20.10021183781608
C	5.68821642376163	9.46278474796711	19.50450525430411	C	8.32416728807303	7.96370090681374	19.66307789681584
C	13.00760796905451	7.70927019240677	18.50591893330030	C	13.22936192834887	8.98980109213882	19.05315693071758
C	11.85122139930990	5.63533709181077	19.00131298725827	C	12.85544359765909	5.14898957176614	19.86302477143007
C	10.55260097141880	7.69733094151806	18.73141212384230	C	9.53942080021641	7.29796377635959	19.63062399469022
N	11.78761892083325	7.01944904349028	18.69987386674768	H	14.62744946074531	10.56500418029783	19.41857785652907
H	18.47846014142003	12.61069562886630	20.31442173404949	H	13.77530702397969	3.44678669979332	20.76804850462918
H	15.58754525546185	-0.50091135622773	20.91241498687086	H	7.56782867655498	7.63496321922325	20.37847764019262
H	3.29799879156506	8.66898346321240	20.49019477037668	H	12.42612789113316	9.50203438750864	19.58389072924441
H	16.74093988320057	11.13440750427036	19.57334782864741	H	13.54359116384745	5.84765655484359	20.33916549175497
H	14.15054642093519	1.37721162646977	20.61476432757302	H	9.71661744801845	6.46724569674285	20.31387431119417
H	5.65157279988411	8.52609856543981	20.06577413139238				
C	14.48075156692231	9.58538979104481	18.96003155394547				

(b) COR

H	0.45879092836614	0.46650745010604	-4.75183428551218	C	-1.04163052805222	-0.96425408627665	0.17879821067357
H	-1.34691793436205	-1.21253646958131	-4.44180573590996	C	-2.07970708127284	-1.92337586790417	0.35692931912243
C	0.14707974648399	0.16615442154111	-3.74786153074282	H	3.49956575525384	3.22153989871348	0.65286262260830
H	2.15533365808426	2.01540343344451	-3.78619961516040	C	2.70085014369722	2.48882687396060	0.79644532181635
C	-0.85347542005790	-0.76295553436697	-3.57574631595554	C	0.63874384030374	0.58245742118834	1.13839247894711
H	-2.80023656749304	-2.56628007386158	-2.93553453639708	C	-0.40230231973871	-0.38165073393486	1.31726089560122
C	1.84641077671411	1.72513863180002	-2.77832073871768	C	-2.46270412861901	-2.28459272409157	1.68287327086285
C	0.80301002474814	0.76272557398938	-2.62955473178868	H	-3.26280431455631	-3.01743464439738	1.81695455309872
C	-1.27509444163968	-1.16352868370074	-2.27248969148621	C	2.31180516684058	2.12288223320105	2.06421235737829
C	-2.31176896038292	-2.12293782063869	-2.06416021523992	C	1.27510017248913	1.16348745230100	2.27243578172805
H	3.26287740122952	3.01728544899301	-1.81711180478483	C	-0.80304075971438	-0.76267202264655	2.62949555055907
C	2.46266606868858	2.28460858132443	-1.68283710596072	C	-1.84643853827337	-1.72508212959185	2.77834824035094
C	0.40228158303759	0.38168502782280	-1.31731620486480	H	2.79986690722113	2.56596829987201	2.93589145293315
C	-0.63875057965257	-0.58246086667803	-1.13844651115456	C	0.85348491260395	0.76295577288525	3.57571818269296
C	-2.70080446112190	-2.48888120379536	-0.79640205251749	H	-2.15485744634455	-2.01509727059044	3.78641888414718
H	-3.49955206277955	-3.22152937164776	-0.65273791444696	C	-0.14711640219081	-0.16613154029831	3.74784335485349
C	2.07971659825850	1.92338881432141	-0.35690662407344	H	1.34711587406881	1.21271808201951	4.44155813382162
C	1.04161917768144	0.96427390490934	-0.17884004897266	H	-0.45911678951887	-0.46660627839112	4.75166705249063

(c) 3H-HBC

C	5.57061229222369	9.56696959887937	16.21640905661554	C	-0.67389382388935	9.75932595933217	19.08106116311131
H	5.32804043806563	9.18164567954041	17.22214896368277	H	-1.75228674122433	9.61173469376017	19.13233482508376
H	6.37801384458573	8.95981289899804	15.78561305720571	H	3.14390879345594	10.37066209994992	19.27044789076666
O	6.05015393437867	10.89983486739757	16.26693972685397	C	-0.16079112293022	10.97458173842614	18.55635229415611
C	5.24365051354874	11.85846397013891	16.76730635253689	C	-1.03674122219766	12.05365656806009	18.13863565785966
C	3.96837622606983	11.64345858658196	17.25826283524334	C	-0.51949923285526	13.36394225353213	18.17582796015084
C	3.15270210633690	12.70195060824034	17.75015198452642	C	-1.41763457066154	14.48561286863451	18.18953475527867
C	1.78647353579499	12.48217274683499	18.17134249280813	C	-2.80853573914448	14.26889383500218	18.16444393840112
C	1.25431392294868	11.19316593837225	18.58785056158685	C	-3.66531841606510	15.35120730211821	18.61283524285050
C	2.07642460264913	10.18609381455456	19.15736854778049	C	-4.95138722544694	15.10778683923857	19.15977450764800
C	1.54738872689669	9.00346393897791	19.64939779574834	C	-5.70321592345941	16.12449025607727	19.72478358001324
H	2.20742703317704	8.25930279345839	20.10171059859354	H	-6.67624379874544	15.89788610927103	20.16717071154890
C	0.15803946630931	8.78547105674117	19.60791001698907	C	-5.18705385815139	17.43220923239581	19.77523833235236
H	-0.27230638531693	7.87258920546099	20.02772449294113	H	-5.75565251571752	18.23030248271418	20.25791719287622

C	-3.93162779368851	17.69963366821449	19.25393426009732	C	-2.16662419830105	19.21553828603956	17.26156296140082
H	-3.51719494534217	18.70077388496722	19.36581007219063	H	-3.23681225190208	19.02782527053821	17.26521726284919
H	-5.33216225039974	14.08747250102002	19.19918973512474	C	-3.28819092339111	12.98827521147537	17.69798654535765
C	-3.14473906172716	16.68378106919910	18.65344922000326	C	-4.57647774510147	12.84303561940424	17.11076577773789
C	-1.78317115906145	16.92555394563090	18.20619612229126	H	-5.21887186035414	13.71675513599054	17.03918568655038
C	-1.29054882716373	18.20510496772836	17.75069292168382	C	-4.98914088370998	11.65400559842305	16.53658342567378
C	0.11715085536309	18.41729667090913	17.65307219175716	C	-4.10573484329450	10.52571823402060	16.54556517309024
C	1.02464348254789	17.36587962336200	18.05876772026452	C	-2.84923043989188	10.65048514839201	17.11165178849845
C	2.42112046687742	17.58392089270809	18.39403104443481	H	-2.15734870086215	9.81634412137833	17.03707508084010
C	2.90131275693296	18.84790353064189	18.82988490667146	O	-4.57496194407668	9.42086338296986	15.93080771736788
C	4.20974929984325	19.02883675397042	19.24530836921410	C	-3.72895036417117	8.29095890430598	15.81315393291676
H	4.54345267687933	20.00954761783886	19.59379952421370	H	-3.41475928215888	7.90710180459103	16.79934752607708
C	5.08597310240763	17.92989741369549	19.28485157620305	H	-2.82895362491282	8.51993199381900	15.21668225054011
H	6.10259493544452	18.05077843694360	19.66683620015444	H	-4.31441556693782	7.51709106751555	15.29842005816225
C	4.63763658025438	16.67757296530585	18.90099228053971	O	-6.16389581570806	11.46491196904680	15.90090856900228
H	5.30076643403917	15.82427129349753	19.02975300066973	C	-7.02613668314849	12.57711388668305	15.73375991415416
H	2.21147316283988	19.68697689398617	18.90028147289930	H	-7.36451466973280	12.98390407038045	16.70214082795909
C	3.31713341168244	16.46669399089484	18.42401956481024	H	-7.89879073701892	12.21379621452886	15.17487471441707
C	2.81946787502373	15.14027917421205	18.09371147244617	H	-6.53667429978414	13.38332550868738	15.15935983336038
C	1.42734887917166	14.92605351392148	18.14688389945164	C	-0.89783583819465	15.82960407500435	18.19327942311546
C	0.52288159842592	16.05125159299412	18.14173593164858	C	-2.40058330540664	11.87119877249972	17.69181421943369
C	0.58218381147658	19.61854097078101	17.04771657457837	C	0.90648646274733	13.58337382915326	18.17700659974891
H	1.64760025047248	19.74295420985100	16.88010286388641	C	3.66666254697195	14.03360547265471	17.70441126357011
C	-0.28250657456603	20.59165316366046	16.58152391949264	C	4.96752879830483	14.23766522885712	17.16453933812615
C	-1.69512170372955	20.38781875926193	16.69979390233784	H	5.32774544668290	15.25304419548006	17.03446692794767
O	-2.46626382649453	21.37005306521508	16.19332174626593	H	3.55297683878119	10.64154807143168	17.21007758512661
C	-3.87317218860473	21.26540324047994	16.31795740151961	C	5.75899493565850	13.19451108857808	16.72089000315698
H	-4.18140428656369	21.20299304563450	17.37627813162475	O	6.99270049987954	13.33482390013178	16.19277542747712
H	-4.26997860087125	20.39097815988547	15.77520892236982	C	7.55061702542064	14.63526965650512	16.10258015052592
H	-4.29521521647948	22.17764355272708	15.87602455166062	H	7.66356423736857	15.09782981825283	17.09865533805267
O	0.09383589334646	21.73285314042542	15.96887768991329	H	6.94040025914212	15.29843319161181	15.46593132427178
C	1.47905105176957	22.01161634395514	15.85206217684115	H	8.54302055666193	14.51843319712940	15.64722322665826
H	1.96144421358465	22.08271334805349	16.84235516499838	H	4.67591175952758	9.48132922915485	15.57581662201935
H	1.56186903030572	22.98098497785468	15.34256679990505				
H	2.00171641628020	21.24666452324906	15.25292763744021				

(d) 3Me-HBC

C	-5.25596147481822	17.42173097268370	19.68349531681178	C	-0.89440960285737	15.82167499834538	18.25263523168843
H	-5.84481656915315	18.22003885879188	20.14327878633771	C	0.52845060443511	16.04014121212002	18.19925095314577
C	-5.81078247501850	16.12692687146563	19.56122586919174	C	1.03700964282168	17.35374986459944	18.11496657788923
C	-7.22243221309458	15.85729177077445	20.00972635379863	C	2.44442051479940	17.56347171494027	18.41148503314848
H	-7.37129386556723	16.13261911151377	21.06809433947441	C	2.95740873133167	18.83360024687998	18.78858371391918
H	-7.49195663437264	14.79780319207149	19.88365614502238	C	4.28859686303712	19.04356776407753	19.12109089171686
H	-7.93812801033821	16.45973682487371	19.42605852496088	C	4.81014349624910	20.41565948265083	19.45560816663970
C	-5.00667961916092	15.11892092982232	19.04513503264709	H	5.23212504536182	20.45419343704125	20.47482665341048
H	-5.39691095793015	14.10208697721448	19.04135246788645	H	4.02083435145756	21.17867466409520	19.37774847766989
C	-3.68166759190360	15.35096765036865	18.59274661850345	H	5.62487510273973	20.70040953970427	18.76895730664203
C	-3.15036112899178	16.67464283263477	18.68284238800695	C	5.14278519814801	17.91848372609382	19.16641301374328
C	-1.77752370402382	16.91965406961278	18.27744788175268	H	6.17823463649630	18.03835973462746	19.49704937777913

C	4.66767484177180	16.65875118835568	18.85693721932336
H	5.32982826167764	15.80603289656363	18.99451172505145
H	2.27622131241989	19.67827647595861	18.86736829525640
C	3.33082184270000	16.44084093309069	18.42931803481678
C	2.82085232890887	15.11589942877358	18.12421447741918
C	3.65846303872345	14.00338806461389	17.72803440276896
C	3.14004213007276	12.67505167887565	17.79034997592413
C	1.77565220003437	12.46608129433521	18.21974645577640
C	1.23908194994201	11.17889621177478	18.63326597820484
C	2.05643994852350	10.18065804883997	19.21786760974047
C	1.54691751757601	8.98174592674199	19.70586954736473
C	2.45535605524938	7.95912121582448	20.33659457440693
H	1.89006355123185	7.24782748268927	20.95857657290706
H	3.22187167805144	8.43879333767654	20.96666515534027
H	2.99046491115277	7.37274244385372	19.56813995525144
C	0.15375489965016	8.76890817108236	19.61941453305012
H	-0.27943713483006	7.85254485188291	20.02980225146579
C	-0.67950311726492	9.73580383018356	19.08251721721372
H	-1.75630540319545	9.57180097683846	19.11689884044022
H	3.11900447320616	10.38299494396608	19.35422659885985
C	-0.17390992606943	10.96026420925899	18.57651371619382
C	-1.04447543994923	12.04467712830561	18.16074864698921
C	-0.52656906336478	13.35476518996102	18.21505411839063
C	-2.40056564269987	11.86931912127300	17.68921880292964
C	-3.28284650832484	12.99074795801882	17.67817004086329
C	-4.55420286902915	12.85171471979717	17.05405670571782
H	-5.18933098922990	13.72809667629622	16.96344549492493
C	-4.96066493610676	11.66521716554052	16.47108866042575
C	-4.08439249131553	10.53243440653708	16.50033197031581
O	-4.54409602012612	9.42974401392980	15.87434918260260
C	-3.69897051290710	8.29790621654012	15.77261730568220
H	-3.40256261057373	7.91411485425000	16.76430614970124
H	-2.78815170205785	8.52462861974191	15.19167752209579
H	-4.27688206899258	7.52483383942052	15.24825560158133
O	-6.12305807447071	11.48555806962702	15.80955592672264
C	-6.98019906145489	12.60181892180147	15.64312602336490
H	-7.32471347109318	13.00383491773051	16.61165013292248
H	-7.84989787162013	12.24566413961373	15.07520021398425
H	-6.48380485089365	13.41056893851182	15.07823852639303
C	-2.84117437638283	10.65155553380659	17.09600307638818
H	-2.15078283712373	9.81529115748751	17.03553739478271
C	0.90025840185952	13.57018699258090	18.22498099074825
C	3.94402748713437	11.60759555093712	17.29805021307537
H	3.52331585042842	10.60713711642874	17.26847050925710
C	5.21163228547883	11.81254793751095	16.78430487203905
C	5.73041577067336	13.14628773044771	16.71633397726185
O	6.95508187270048	13.27787547963037	16.16468520818526
C	7.52275812495188	14.57332513148105	16.06880369088529
H	7.64908238627315	15.03492355088654	17.06378289352891
H	6.91230523853746	15.24186234440837	15.43791015663364
H	8.50986152593388	14.44824403654978	15.60419476555193
O	6.00587082100258	10.84535593534013	16.27970437769878
C	5.51863712859195	9.51495534504408	16.24845780655152
H	5.29186792469407	9.13880102978574	17.26137951177401
H	6.31509116746556	8.89963949779094	15.80896945456815
H	4.61237125145462	9.42870962626548	15.62442666404739
C	4.95123435838615	14.19660775261707	17.16488999164266
H	5.31447631573867	15.20861768684874	17.01951908204941
C	0.12657352549726	18.41312496666909	17.73145649239549
C	0.58422773815624	19.62294758016241	17.13693928507638
H	1.64510474116416	19.74530494005902	16.94619279659377
C	-0.28101459263812	20.61474393519631	16.71406342100250
C	-1.69144987328342	20.41487033786060	16.84758637772421
C	-2.15763941561523	19.22930786165813	17.38363429412023
H	-3.22788369009243	19.04620379669851	17.39407917460933
O	-2.46850229376606	21.41343728167683	16.38269473515079
C	-3.87040504621174	21.32634806088485	16.56709131861625
H	-4.13268695852368	21.24331685934082	17.63651519345507
H	-4.30540031643377	20.47184515044319	16.02246083442766
H	-4.29682142397081	22.25513894811990	16.16547733591359
O	0.09607863854676	21.77369604182994	16.13517950810250
C	1.48048854377984	22.07243970634076	16.06813770192266
H	1.93410755644908	22.10544549436633	17.07448317327125
H	1.56561499880141	23.06426774278408	15.60435998326202
H	2.03028374035498	21.34181527995729	15.45115116027854
C	1.42741161946894	14.91013154979966	18.19315479669535
C	-1.42107666076598	14.47958243153445	18.22622973811229
C	-1.28084563175701	18.20564617031673	17.84339089580697
C	-2.81327741933192	14.26816087823102	18.16739872551483
C	-3.96348109442137	17.68063936457099	19.26651769629078
H	-3.54567889817499	18.67103123057126	19.44143144081798

(e) 3F-HBC

F	-7.02886499751752	16.01891886538988	19.89918337417184
C	-5.77438359196295	16.23864414582213	19.45803066928867
C	-5.27532091114320	17.54539154427672	19.49410916550733
H	-5.89159039467338	18.35342844859823	19.89119177629483
C	-3.97817295486409	17.76171103831911	19.06543236242705
H	-3.56114620012119	18.76271351520752	19.16155104948000
C	-3.16317092135455	16.70964816879070	18.57382009795712
C	-3.68933232964039	15.37775610334444	18.56717955579094
C	-2.82029335706534	14.27160530901879	18.20229111032627
C	-1.42823562790887	14.48464780452848	18.25266935761487
C	-0.90254658075016	15.82757080738845	18.24920885545622
C	0.51846741031937	16.04404351889168	18.24824090765608

C	1.03011094053947	17.35624590868121	18.21323706790271	H	-4.32392553451136	7.42302588924042	15.55779656803966
C	2.41417395581355	17.54908459817113	18.60533823018191	C	0.89695085467462	13.57622111753712	18.23839232192928
C	2.87277300146366	18.77801396761604	19.14227977483124	C	3.95371651219327	11.68231190261147	17.18956119358820
C	4.16120814594760	18.88576936208819	19.61956896201300	H	3.55868149223942	10.67256889367549	17.13302384607956
F	4.55811797741084	20.04758423432108	20.17344509221227	C	5.19834132503072	11.93805442963608	16.63902780496377
C	5.05800455243422	17.81096165127733	19.60218655361266	C	5.67574327388794	13.28789932644903	16.57565764735107
H	6.05532298480388	17.92709881313846	20.03011245901229	O	6.84691045262201	13.47372136416302	15.93187494547437
C	4.62062827706536	16.60085206391923	19.09279358586545	C	7.27626250408032	14.80449016064661	15.69053749618176
H	5.28788671071772	15.74207773330429	19.15277904150160	H	7.46931144261979	15.34837892103043	16.63054081724519
H	2.20539988077089	19.63002971448473	19.26015331972429	H	6.53304791222706	15.36545750242175	15.09685769839066
C	3.30825526524798	16.43194431249155	18.58082135536047	H	8.21271446286305	14.73402823402540	15.12144741071208
C	2.80552660392265	15.13068365462338	18.18065989357256	O	6.00376422543433	11.00869094101898	16.08674238776854
C	3.64206786949063	14.04867542722801	17.71958069072841	C	5.56958283879408	9.65948552521453	16.06482428248513
C	3.13914385453126	12.71395788814162	17.73758690853236	H	5.39250335681007	9.27204510365319	17.08339961862480
C	1.78291386033847	12.47999945905357	18.18802715715551	H	6.37495850384788	9.07830002820961	15.59646526518171
C	1.26515471133776	11.18523801436442	18.59326343532514	H	4.64769942640148	9.53947017376559	15.47004631564195
C	2.11768828290499	10.16746224609453	19.08857565561751	C	4.90522943505309	14.29910639365910	17.11756992718905
C	1.58885682432985	8.98826016305027	19.56699125925639	H	5.23810045367396	15.32743294392912	17.01345674592380
F	2.42605401160235	8.04716277357974	20.04700583962053	C	0.15604757998364	18.41976708424903	17.77528285840355
C	0.20960210023730	8.75355395508978	19.61492246854003	C	-1.25343853585484	18.20136399426869	17.75761522180740
H	-0.17515718872387	7.82766427547920	20.04653942920906	C	-2.08756573088994	19.19337121735465	17.16879605467189
C	-0.63826775759286	9.74675528315274	19.15385406263497	H	-3.15095838509966	18.99514044754223	17.06861482127152
H	-1.71188460913597	9.59767724717261	19.26252203544858	C	-1.57348844088610	20.35698765719976	16.62779876371001
H	3.19264944434357	10.31896971067481	19.16436894480102	C	-0.15944984442582	20.58353207804904	16.65947369663542
C	-0.15081637670673	10.96857100182448	18.62059080459866	O	0.25890008477133	21.72517613682915	16.07805986974099
C	-1.04340279863396	12.04767406666954	18.23476020646058	C	1.64469276535128	22.02366681269498	16.09177408607147
C	-2.40834646969988	11.85743283477417	17.79555993803886	H	2.02962074197508	22.10855710309449	17.12307244853273
C	-2.85325357049985	10.62308212743118	17.24081732930675	H	1.76211750343076	22.99026610128623	15.58420118122806
H	-2.16105557930701	9.78789617664708	17.19007607209203	H	2.23273725353721	21.26255528624340	15.55125669529459
C	-4.10290970305614	10.48400495754284	16.66447090147378	O	-2.29889471058783	21.31049264088910	16.01167910168386
C	-4.98323071080041	11.61294399170752	16.61743107684059	C	-3.70842803429544	21.17811243420525	15.97499223857564
C	-4.57392946035699	12.81499185141149	17.16504702470830	H	-4.13757956993734	21.13725419192718	16.99106819591019
H	-5.21523873366272	13.68450051230447	17.05693244171118	H	-4.02124769658635	20.28004638642754	15.41526187968352
O	-6.15168660315566	11.41264209204862	15.97540226901045	H	-4.09284463519953	22.06871928651405	15.46072202694507
C	-7.01844384564409	12.51908508193251	15.79265059749211	C	0.66675051034444	19.62693657596216	17.22027054803298
H	-7.35766180770900	12.93815886570824	16.75555744740934	H	1.74250090097016	19.76459101098972	17.16054895316704
H	-7.88983680521732	12.14426443082507	15.23963875153022	C	1.41613224690670	14.91691717373119	18.23673977177675
H	-6.53217072821262	13.31883687101008	15.20674498116865	C	-0.52961552939050	13.35963599404468	18.25750760976086
O	-4.56501542549602	9.36478055444112	16.07280718822138	C	-3.29414229232642	12.97655171286876	17.76573326986585
C	-3.73941309645075	8.21347965739859	16.04728381091834	C	-1.77841170560118	16.92949908384490	18.20159761167710
H	-3.46677508492252	7.88223814451171	17.06459708053115	C	-5.01336571077200	15.17062267817844	19.03200476141978
H	-2.81492837043490	8.38944585322478	15.47091592757577	H	-5.43320577651185	14.17040726253056	19.12204068764341

(f) COR⊂TPACage^{6+}

H	13.40472627295455	10.14679306913780	11.21790057801880	H	8.46645385219500	8.22248627078342	10.85363835942763
H	14.54907831907436	6.01560304057633	11.16578907519722	C	14.21006080623479	9.70143038372684	11.80277936213448
H	10.68628112748811	7.17395460816237	10.73150519344233	C	13.75080939708572	5.54168389559979	11.73853917774908
H	15.51379819444818	11.36257336175416	11.47849640153890	C	10.52052454091465	8.01245767631262	11.40920806853735
H	14.51597396874578	3.57303831420345	11.40487742373272	C	15.40673568031305	10.38584121778124	11.95383779082714

C	13.72798580201552	4.16209707585311	11.87722290539282	N	13.22233850653024	-3.02177808077649	13.12012907826782
C	9.27057973342437	8.60580843658597	11.48439995656934	N	2.68743045008950	11.25021106151138	13.13755474757994
H	17.44941962316051	12.30911808483254	11.81803325677462	C	21.68710899440962	12.26090994569859	13.56471400878385
H	14.47831002160229	1.46423240540851	11.95656957138033	C	12.19187596262134	-2.20823173298809	13.46695152192944
H	6.52478695600732	8.96484306592396	11.39584464239800	C	3.78746793812125	11.87925394419153	13.62624470005058
H	18.97506521318406	14.17371531460044	11.64270837017014	H	23.18846392775185	15.17569226371411	13.00443103083499
H	15.36178566678663	-0.76907985300062	11.85934242115864	H	14.04935922750574	-4.93963388664465	13.06267159331381
H	4.13972620136730	8.92292016844648	11.20830471777489	H	0.61523457545408	11.00112562373213	13.14879101006969
N	12.80110061662250	7.74805439685886	12.25339471485719	H	24.00640751817220	13.59671814030296	13.06095218985138
C	14.02514051104324	8.43239504307048	12.39625375796723	H	12.27621809483061	-4.88691964700010	12.91254716917769
C	12.75303357893412	6.33684474339265	12.34146165034189	H	1.16106052929417	12.69547188027026	13.13051058226883
C	11.57538635253196	8.45979453273929	12.23308966854470	H	14.96157774356696	6.91299757343412	13.62926799981809
C	18.14450952864490	11.70110100721120	12.40328464913833	H	10.94107648963166	6.30328688202597	13.52881900906649
C	13.54995624763682	1.15706137673505	12.44467158893419	H	12.17385311015145	9.92282677695266	13.70948160851662
C	6.54114094557009	9.82714920345474	12.06738186633823	H	22.50322156482036	11.78116147522578	14.10419753461910
C	19.72517693491461	13.60312073734095	12.19226129821530	H	11.32678778674316	-2.68724397795378	13.92524592194261
C	14.44856289550568	-1.14802546629830	12.32028240663036	H	3.60838306997879	12.70804570785900	14.31044899628881
C	4.07202583774406	9.76911567519683	11.89367789295061	H	17.08664147469198	8.11352071501090	13.86136417264484
C	16.48027030473551	9.82906274670090	12.68815005319387	H	10.87146101857156	3.85054844446140	13.71625563607560
C	12.69345277331635	3.51907643654761	12.59595553772094	H	9.95584592093156	11.00600637631537	13.81048534428665
C	9.03126599673429	9.68488350838528	12.36782902978930	C	23.19211799433218	14.19389163048403	13.49098585695054
C	19.44455944966973	12.29251709324879	12.64397732222230	C	13.13764466133713	-4.46996381493495	13.44968031195813
C	13.40586622185241	-0.26334696397197	12.67993853959699	C	1.33899606867607	11.70641637992455	13.57348127349977
C	5.24239637603355	10.37218936474400	12.40861461457725	C	23.31948126349776	14.29957873699818	14.99159774205409
H	21.18573641240003	15.20626453459433	12.13175926138734	C	12.99388850633606	-4.64325294822034	14.94103532659035
H	15.12434548810947	-3.20821757937374	12.28947497367694	C	1.25028965881228	11.76278911157634	15.07818925409422
H	1.90359947296529	9.75656835161338	11.91430401899182	H	24.91605614843845	12.84926760988849	15.13677754845711
C	17.76088723410351	10.48426391007219	12.87394975176615	H	10.91142090483338	-5.21867247970425	14.85113278974016
C	12.62439978928335	2.08559055159186	12.80071151182352	H	1.11456696666482	13.91912867690967	15.16379643329246
C	7.71876997700302	10.27142010925330	12.57798590022275	H	21.74294346897169	15.77661508373960	15.17754452766921
C	20.94537925530474	14.19368991582211	12.45454109666000	H	15.04999983475062	-4.09725469007264	15.36085569628761
C	14.33473078229877	-2.50374375143933	12.55020354808505	H	1.36867210430896	9.61182301121715	15.32643650749451
C	2.82324290940405	10.21667055940795	12.27521769520175	C	24.22765889779419	13.49487398876872	15.68813827719762
C	20.48992253446049	11.62854038502606	13.33502892594220	C	11.75566686877057	-4.97584213781267	15.50091602082169
C	12.25387963634759	-0.85136900088833	13.26258822739039	C	1.17128259670019	12.99234519773197	15.74056669130551
C	5.05411226803472	11.47136122734679	13.28376760405509	C	22.44820699199296	15.13427577040609	15.71157727444372
H	20.37493630087059	10.61083216078667	13.70497676897161	C	14.07517743741724	-4.34901579847668	15.78761587861758
H	11.39215684016750	-0.25492471043056	13.55984434214015	C	1.31525967527677	10.57943750878597	15.83281788816959
H	5.89572429497725	12.01082036720726	13.71588328064933	H	15.66572626138220	9.36223511137264	16.42914441130727
C	15.08342772635937	7.87549005923556	13.13813831774573	H	13.52610597612339	10.60794253434516	16.44121894152497
C	11.71247362222952	5.70611717915073	13.04634279551794	C	14.71607527540714	8.82354053251568	16.41982142554890
C	11.36781918528766	9.56693779646470	13.06993051071611	C	13.52825459714217	9.51465176176258	16.42623859802656
H	18.47351029688372	9.90600723855396	13.47011160592736	H	16.90369605645359	7.19946976732885	16.40821750467867
H	11.71823213638218	1.75163839601629	13.31367744429130	C	14.73938687491916	7.39870080750850	16.40165541582903
H	7.71001990221138	11.12636220427871	13.26091822077519	C	15.95606534162945	6.65554020711517	16.39665067669108
C	16.28233316967328	8.55934377902907	13.27138732949812	H	16.89462578418567	4.72705575799846	16.37322091668013
C	11.67926234169446	4.32371247822151	13.15473931392378	C	15.95138055513214	5.27962186516180	16.37665946010154
C	10.11746308731277	10.17052241170057	13.12592037939509	C	12.27696735953739	8.83080846398438	16.41464279660267
N	21.90671866942341	13.53171393255354	13.13877806032869	H	11.04553521849761	10.61497419154171	16.44346505157475

C	13.50601931944193	6.69076535845503	16.38592170941187	H	14.96721145125681	6.90766228171711	19.18505316721292
H	15.64007514296798	2.57273958682974	16.33771569244981	H	10.95800110962934	6.41799553484039	19.17847494346482
C	14.72836067409796	4.54475752919679	16.36097623389969	H	12.21832265655904	9.99457925431216	19.14115098228821
C	11.03244911358242	9.52225814181400	16.42469805432822	H	24.05002567070753	13.56153216992187	19.71669359566213
C	12.27136787374861	7.40924960060182	16.39090051880888	H	11.41863662496113	-4.74181150743160	19.50953941940886
C	13.49941544157887	5.26225269916136	16.36506881885376	H	1.25630363360501	12.94893608825342	19.76179937443476
C	14.69357402781714	3.11933247453459	16.34172027821752	H	23.23040035517768	15.13994776578853	19.80075514906145
C	9.83817701219055	8.84351381774881	16.40767885556285	H	13.15650576033871	-4.99490140062064	19.80778163716791
C	11.03141757474764	6.70106988759629	16.37271300452479	H	0.66785874818177	11.27265890464406	19.88497965615753
C	12.26018417805887	4.55378362505567	16.34901431524089	C	21.72689676586313	12.22700654398245	19.23586356711667
C	13.49999351111690	2.43740829207258	16.32753188182573	C	11.81975925119219	-2.07459254628013	18.94705412683960
C	9.80209044415826	7.41959907585435	16.37977690219262	C	3.84454891521364	12.08641031512921	19.33276968579593
H	8.89330704900803	9.39178504963514	16.41454988462074	N	21.95000284862560	13.49521655556161	19.66795707991815
C	11.02605126400417	5.27266197972460	16.35059129528113	N	12.59392548687985	-3.00014006739811	19.56972478979590
H	13.49134573753250	1.34408603510625	16.31060947295664	N	2.74379779371386	11.46450749520109	19.82925121667145
C	12.25578624532997	3.13151247394836	16.33145138251507	C	16.30900691762089	8.53316541420368	19.55964429899453
C	8.57830349982229	6.68611056656234	16.36134568298703	C	11.61905959109773	4.40676244121422	19.52980311910083
H	7.63555725949417	7.23981465493889	16.36530748974640	C	10.16641901386821	10.26222739954051	19.73256990098662
C	8.57228021795599	5.31117621786615	16.34050509247859	H	18.51339835124416	9.86488417287430	19.35987830496572
C	9.79013704795848	4.56710487188207	16.33565398827633	H	11.61575134957849	1.87462712515588	19.21845764350205
H	7.62539220863588	4.76519521978944	16.32766552126315	H	7.77107379690110	11.26553058117423	19.63090547088299
C	11.00439424844208	2.44907869866686	16.31681480030808	C	15.10062695310204	7.86433638523937	19.68464927016309
C	9.81393656083040	3.14054071260904	16.31921549286822	C	11.69226676842025	5.78367659451119	19.67143292578993
H	11.00198886046835	1.35535538763090	16.29934765248296	C	11.40625899266795	9.63521700940110	19.77127152427701
H	8.86374667571003	2.60093071756641	16.30682314274656	H	20.41466155835323	10.57609892473253	19.09960720730085
C	22.45687832434002	15.12692891411751	17.10373288394885	H	11.33064823023530	-0.04054689571726	18.66063647565532
C	13.90341622174680	-4.34007130930221	17.16857381296468	H	5.95352482309302	12.19159070946203	19.21293718809912
C	1.32892055848856	10.63151020709966	17.22377207116558	C	20.53314082819435	11.59140473516851	19.47540444068514
C	24.23609388590056	13.48720004749807	17.08743646054795	C	11.97815562941082	-0.73332552914274	19.19531183490051
C	11.58164316551247	-4.96116938669970	16.88869419269396	C	5.11097366242601	11.65753158359792	19.65037787069575
C	1.18592138600461	13.04463746851697	17.13844043381651	C	20.99618633287409	14.15148654637575	20.36797503041907
H	21.75861955788050	15.76376879698393	17.65330904094504	C	13.53657065512978	-2.61101355274611	20.45883674682014
H	14.74511101453038	-4.08250330428625	17.81654398845250	C	2.87795997910126	10.41794088544849	20.67563792962890
H	1.39275628042748	9.70363481498704	17.79866813420400	C	17.80599916494470	10.44461501155435	19.96085470240148
H	24.93051184879596	12.83549139332031	17.62385427689284	C	12.44231279338839	2.12995970291480	19.88727614764338
H	10.60311628712681	-5.19152795688825	17.31697749428891	C	7.77041686646886	10.39656142180121	20.29596532283220
H	1.14129648843507	14.01118853750337	17.64655905662199	H	21.24018792901801	15.16187567874056	20.69471038005121
C	23.33668127048473	14.28425037048632	17.80380972368877	H	14.12227381880780	-3.40311687667830	20.92422029436069
C	12.64547926494229	-4.61758550815748	17.72913921573439	H	1.95718226297897	9.96615415731910	21.04393788104338
C	1.27904966719183	11.86794246916470	17.88895711490564	C	19.49532645718321	12.24992067424573	20.18298114882733
C	23.22936532370399	14.16283049912833	19.30474715741662	C	12.96286035319981	-0.27848629376691	20.10986843772857
C	12.42888496851727	-4.43294254431993	19.21066561003726	C	5.29667446142157	10.54137729578077	20.50475667027630
C	1.39840933358090	11.92471674707741	19.39246023784500	C	16.52142887780664	9.79783299833414	20.14891376235294
H	17.10891134467596	8.08001647043787	18.96913944927869	C	12.57723247800854	3.55482951081950	20.11706368168520
H	10.81617205355432	3.97145032443599	18.93265979529249	C	9.07222209673046	9.77721112236607	20.47968987090369
H	10.02034097394524	11.11980641043939	19.07207303282894	C	19.77992453731876	13.55762402997481	20.64063597968904
H	22.53766467885180	11.75227400680385	18.68379297713289	C	13.73795279489614	-1.27557389919023	20.74527184407288
H	11.08496608935271	-2.45222290373406	18.23593045807484	C	4.12637960419624	9.94856497790558	21.03140704020004
H	3.66489533072296	12.92629230750199	18.66206806286078	C	18.19717613702896	11.65849717880910	20.43298305231658

C	13.19207012021349	1.12117818980350	20.40361508248309	C	15.45453565067397	10.36196564444451	20.88726366105967
C	6.59077143270062	9.96725476194932	20.81469288367780	C	13.61005818023959	4.14693070056931	20.88019185854647
C	14.04637939261976	8.43242128450004	20.42412672880262	C	9.29235444769199	8.66840118603580	21.33060161564813
C	12.72807097980269	6.36320740065038	20.42445404770238	C	14.24864495166404	9.69277245383107	21.03075019613689
C	11.59543174539990	8.50375416171680	20.57990074866596	C	13.67959164566906	5.52388245199871	21.04095001684128
N	12.80890955865708	7.77115233264607	20.54638337858551	C	10.53118554528734	8.05076403486038	21.38825692285164
H	19.03507535000976	14.12306149786903	21.20252393377714	H	15.57344811257207	11.33357295026707	21.37012592145789
H	14.51140533232523	-1.00367261901301	21.46521421015459	H	14.36534332824493	3.52306600567852	21.36173287206844
H	4.19404461709783	9.09233119216038	21.70401926419679	H	8.48158445058749	8.27943267524377	21.94911200454409
H	17.50803793774635	12.26691042710831	21.02477054622895	H	13.44862663259715	10.14504738596540	21.61750981820299
H	14.01472112204415	1.33579397874083	21.09053995189675	H	14.47910234676281	5.96094541076460	21.64122006197174
H	6.56679019063379	9.09382704490971	21.47126429290632	H	10.68081513615497	7.18945764836518	22.04055049163359

(g) 3H-HBC⊂TPACage^{6+}

H	-4.28309746818863	14.13498285740313	11.71110890468213	N	9.06242000400816	8.97431583055507	13.31291530952756
H	1.53922253610335	18.81718245622131	12.03678376865522	H	-12.03589292089701	10.48446711977936	13.05105601853744
H	2.55836220395475	11.18285540431458	12.43856376442473	H	1.11003928544211	27.48083581635109	13.02810467841280
H	-8.53949307649116	12.92033709784191	11.44474717000179	H	10.28608080718305	7.28762341495601	13.04872201522334
H	1.80815961882035	23.31529270172082	11.32414712230283	C	-5.03013650742419	11.92859675155897	13.24546260444471
H	6.06188033594939	8.55574963393507	11.87197496431550	C	-0.33878411614709	20.45665015125772	13.30143228550323
H	-2.14441796222316	15.32877519523011	11.84700338990975	C	4.91005912294088	12.05126627117577	13.63389764097995
H	1.62116245789173	16.36664990166916	12.14864941231802	C	-3.77968241897916	12.66450252108182	13.24264855519967
H	0.43185507989592	12.38401647921389	12.37473907704307	C	-0.23865822950192	19.00997062470314	13.28230982925704
C	-8.58858033035059	12.03033365593141	12.07434709264174	C	3.60782018877399	12.68897705878236	13.61286236737962
C	1.11925121165824	23.69296663402217	12.08241040681525	C	-1.32324800174761	14.06193446604053	13.40383666417815
C	6.83844636526626	9.04448805110239	12.46294231865631	C	-0.12182180126362	16.18129354022423	13.42723165410566
H	-10.74198626376363	11.85460256467125	11.87896546810779	C	1.12282272537304	14.06547278844899	13.55981794191712
H	1.69684262090870	25.75984472949832	11.75738753023996	N	-0.09712749443416	14.76829912775997	13.48239377684892
H	8.27176951921911	7.44985279998988	12.12751922286071	H	-5.04339598291185	11.06038220760885	13.91110128002404
C	-3.53208774752108	13.79358383225983	12.42632483810747	H	-1.13940716012573	20.83379304802119	13.94329061241625
C	0.78395943648004	18.28907651162392	12.62182015021916	H	5.67087319747561	12.59534347455236	14.19895153131518
C	2.48098790463337	12.13981548702107	12.95706616757235	C	-7.57746162405767	10.32550336732153	13.44222883479861
C	-2.32572732686117	14.47334856643471	12.49862167294405	C	-0.57589207631883	23.39923083133706	13.76741874300591
C	0.83833703738829	16.90364723559162	12.68540750740685	C	7.67503083454829	10.85325167625438	13.81224250273252
C	1.27133909941420	12.81389223761255	12.92084056247821	C	-8.82646698314532	9.78512729689281	13.62659418085956
C	-9.82114103365718	11.45036039198584	12.30248947405542	C	-0.58883411799017	24.76092305679558	13.94202212185633
C	1.06962653678473	25.05614574313067	12.30719830258961	C	8.87284501953487	10.18976996500038	13.89628236820726
C	8.06209559469521	8.41575138036476	12.58945444416904	C	-11.27907724600324	9.83079344564588	13.50364109394837
H	-6.19083015536376	13.10912696786799	11.90734726844617	C	0.27513018474783	27.03792368389026	13.58699181233083
H	1.22137382324234	21.04257125851097	11.96866978906346	C	10.31678456293232	8.22500067782986	13.61872932640620
H	4.53258978488210	10.32672978728766	12.44669781314903	C	-2.75501726336394	12.25119260606815	14.12156304068196
C	-7.41722148592044	11.51663592389753	12.68442343866842	C	-1.20060471752603	18.26772580985227	14.00179106270212
C	0.31043131861554	22.80174286890863	12.83049008305373	C	3.43820300639149	13.93272766930503	14.26119467163925
C	6.58415899214796	10.28378506872086	13.10242706243108	H	-11.40468331373452	8.82340902527172	13.08228748316835
C	-6.16751402667694	12.23364718370578	12.56127505681370	H	-0.65591212836509	27.50273714094540	13.23139337539958
C	0.43755633587000	21.37075114429645	12.65612727834039	H	11.16985229777706	8.81468233669171	13.25420996606672
C	5.26992452252947	10.88231381207051	13.03205667388763	H	-8.99000322077308	8.90056933687594	14.24145497682313
N	-9.94072415847726	10.35583889302537	13.09243795868104	H	-1.23144096700340	25.24699545127655	14.67583996748674
N	0.23836026674277	25.58213918210490	13.23913311078272	H	9.71490645730604	10.58389076316577	14.46481151439569

C	-1.55438225809195	12.94105014552432	14.21816355355327	H	2.04283022622315	22.90105109094154	15.52713473422951
C	-1.14092422223428	16.88578851455936	14.09280381348322	H	6.55285621343757	8.54362177123368	16.00022502751628
C	2.22337168782881	14.60465079581594	14.25310877747970	C	-4.21442500509328	10.24272450872990	16.75560472546566
H	-6.73710958307749	9.84101692860358	13.93686395903253	C	-1.32488342083130	20.37252133923393	16.70911946841866
H	-1.21585615828150	22.79736444866363	14.40987359170102	C	5.95884995827052	12.78686967118804	16.86922461356597
H	7.57729052439460	11.79216415679111	14.35162801325555	C	-5.07617823348455	11.38229448194834	16.70976162637678
H	-2.92239524935502	11.38704872917701	14.76324856639373	C	0.09104076148378	20.51014892776832	16.59093177051425
H	-1.99509018329808	18.79995196291817	14.52291471328219	C	5.39684716679858	11.47338911687578	16.86520764309165
H	4.28308629502706	14.37245043933410	14.79170800500090	C	-11.17167880446122	11.02657368528879	17.10952616270259
H	-0.79579889118827	12.62216874612185	14.93312064997079	C	1.69257947683610	26.64505015754849	17.08771555612546
H	-1.87684582222422	16.34306360700445	14.68656160190603	C	9.34425957738321	7.17828198121892	17.14359785239319
H	2.12076155973772	15.55082345713954	14.78355138241650	H	-5.19930777741062	13.50522279477229	16.88617270838178
C	-11.35536135692734	9.81768337494210	15.01348388481765	H	1.97033876371023	19.51287762425688	16.75370554446492
C	0.43921758634870	27.18846409962513	15.08206719748281	H	3.62456047250139	10.32061507360408	17.15669278826546
C	10.39548227486203	7.99252886769998	15.11258994555449	H	-7.36862762157682	12.63072840476447	17.31408393040050
H	-11.29896817665913	11.98100196507864	15.18463602282939	H	2.48212079178418	21.78037961072595	16.85995465416964
H	2.45187134344263	26.37778390782501	15.09143319411110	H	5.37613759694105	8.78387823466116	17.33316471439863
H	8.63375757899871	6.72411270037877	15.16383270065664	C	-4.57322589535042	12.62860690228652	17.02701361410278
H	-7.05908709287222	12.95119050071800	15.57305054152264	C	0.89894387054138	19.43942295849719	16.91623365479381
H	2.31285593888951	21.16471782567924	15.17854272356280	C	4.07227897587212	11.30785966899007	17.22474123243015
H	4.88896120074182	9.10731029535440	15.63505291490746	H	-2.22272200010626	9.55412789201372	17.09189955315622
H	-11.48603817816230	7.66326432283121	15.18682155432358	H	-2.94206252687082	19.03682009320913	17.09845895487927
H	-1.50537836992965	28.08576000387093	15.40892063746726	H	5.57176658379345	14.86541068428405	17.11204522012356
H	12.20131839263601	9.15419936450501	15.39508422190865	C	-2.89381588414706	10.40676200211375	17.13953033391212
H	-4.63025730776506	7.11658776304228	15.88966823425280	C	-1.86405193253126	19.16566902898784	17.11542083307766
H	-3.84038979455468	22.37515118987989	16.07258904197710	C	5.16558464714895	13.86421943675218	17.21306025959197
H	8.89565185494359	14.00408196728640	16.06699559036340	H	-3.56725459731043	7.62452630761174	17.24351113138604
C	-11.27923458351313	11.02899700658229	15.72270687414330	H	-3.80275974047759	21.21085689719350	17.43898122425577
C	1.60912616547895	26.71526444339830	15.70074229568815	H	7.90653895935463	14.61902946662013	17.43293772430713
C	9.39278347606409	7.24438259898609	15.75467727568945	C	-11.28700187833338	8.61020151323197	17.11775259836108
C	-11.38935463429856	8.61232145397883	15.72112371021199	C	-0.52180607750411	27.60458424279921	17.27216462968309
C	-0.60581622640296	27.67488773604159	15.87570476588714	C	11.34809810131966	8.52957505335683	17.27975147801835
C	11.39230997602494	8.60215133196188	15.88159957715151	H	-11.10602913275594	11.97618108738837	17.64817518495354
H	-3.14008129770812	8.05750703415016	15.55289189884455	H	2.59813313018130	26.25049660875931	17.55711200978810
H	-3.87470825795905	20.61669857458549	15.74469653337935	H	8.54699118117800	6.61088648886120	17.63176435820790
H	7.34611461194554	14.84206701110810	15.74059975283609	C	-3.22982026355066	12.81273918111462	17.46069148746631
O	-4.77813924325313	9.08343798579135	16.35267934321769	C	0.36686372107155	18.21429662382047	17.39864996055599
O	-2.04884160426570	21.45892503889103	16.32846055355545	C	3.25002560156460	12.40016555285139	17.61618830052416
O	7.25461020538503	12.88039565569485	16.46673429636023	C	-2.36934630139687	11.67682095333262	17.51535435221621
C	-3.97392754893341	7.91379090162067	16.25981801609123	C	-1.04654328005411	18.05473821028639	17.46580264604630
C	-3.46704546029951	21.39898176978224	16.40551293440704	C	3.80407132849001	13.71261596390802	17.59573599367293
C	7.87225254330770	14.16146594841465	16.42971109286970	C	-11.14438267869661	9.81301247166857	17.81775439992309
O	-6.34219945070776	11.12686047991407	16.29650748901442	C	0.60710075429775	27.04731727416685	17.88435727260229
O	0.53095970724103	21.70101750840977	16.12170542851887	C	10.30133587017503	7.85469248491524	17.91943268134217
O	6.22594330691130	10.48285630062248	16.46025598977923	H	-11.30873014700980	7.65907515108296	17.65673866903945
C	-7.29661500631112	12.16972380511552	16.31422442331669	H	-1.35786592163824	27.96083389430287	17.87999207533518
C	1.91958528154678	21.88264361634322	15.91697410214832	H	12.12339375431182	9.02683959812871	17.86912699991355
C	5.72134493759795	9.16453030513615	16.35652134019828	C	-0.43085698802543	13.15598663063952	17.78571064163004
H	-8.25975055866343	11.70815278038668	16.05959652343993	C	-1.30258345572853	14.30177282883303	17.73953810911435

C	-0.74569952393341	15.63222151089949	17.73273509004870	H	-11.04076770071856	8.81648603877600	19.73975835033608
C	0.68375113091112	15.81291316904221	17.74262650371150	H	-0.21469021554954	27.25560211306701	19.87906991478340
C	1.55876200861138	14.66846796614454	17.79061911606474	H	11.01932576120323	8.33999045327022	19.90946891806002
C	0.99997710696014	13.34211460658886	17.82313070055492	H	-11.58623252718724	10.50355062502857	19.82691533179436
C	-0.97868004763363	11.85931439966100	17.87407595800425	H	1.55695515900959	27.10842709822278	19.83524781426307
C	-1.59492643396131	16.75714075842865	17.79847065127141	H	9.88100487991517	6.97141095045719	19.85862057385208
C	2.95482788622567	14.84959797828913	17.86859322190145	N	-9.51274705300875	10.27367762021560	19.62413152671275
C	1.85142109213787	12.22644473783502	17.94243998193090	N	0.53041791400432	25.28901156483544	19.62929988521420
C	-2.69953248936967	14.12027622076072	17.78028300404872	N	8.97427524508548	8.85815533637073	19.74686134762665
C	1.22451999940430	17.11185688349525	17.76454767510110	H	-0.33822016600167	7.90160896451221	20.24463064739729
C	1.28231084868724	10.98966066515123	18.44595012188382	H	-5.43844699677713	18.14684196828637	20.02700293326555
C	-3.51185366236929	15.23168318343052	18.24145223150073	H	6.10767868994939	17.59702264316032	19.92951805352637
C	2.58715234714038	17.28485387747830	18.23097644433048	H	2.14458831017478	8.22022978949625	20.29044350528591
C	-0.13755709131771	10.80450856961381	18.41321412764354	H	-6.43820504312624	15.85293484361084	19.93089738377532
C	-2.95161458347379	16.54892098661426	18.27218006103813	H	4.59493585829177	19.58177402104041	19.82033387811041
C	3.45536240488194	16.14830946863206	18.28671174701369	H	-4.77712610252742	11.03787078665186	19.77895632898187
H	-8.70134668833090	8.95035905049615	18.23348498914712	H	-0.48305251492996	20.61633393554326	19.59031266340807
H	-0.71151054977364	24.99664488967654	17.98476392085286	H	5.68922406271443	12.32895205525089	19.86422134790880
H	9.52416522372885	10.20816314392849	18.25572068156932	C	-6.88583995895759	11.13341896100430	20.17093405286206
C	2.07389308027567	10.03098049018463	19.13065245657488	C	0.33908371441590	22.52078918348656	20.11910222056196
C	-4.78048252952712	15.02673200178345	18.84267605484975	C	6.79461851031426	10.54869745186563	20.30783678297363
C	3.03394605443300	18.50900662694040	18.79738768131873	C	-9.28745316287116	11.23782821835519	20.55551747047209
H	-6.36311645820142	9.64220325780275	18.65564272371848	C	1.17596422780498	24.73316312091538	20.68935570220215
H	-0.92057767938826	22.55714598660419	18.33161146464606	C	8.10059356287881	8.54238182005145	20.74003708942741
H	7.65033151655635	11.74945573208899	18.69203579273029	C	-5.52275274159156	11.55939174484555	20.38342141757855
C	-0.68150011604857	9.67029993981125	19.07408944068634	C	0.18836451787513	21.09698991985094	20.30527156444767
C	-3.68116670151248	17.57332581592020	18.93166951679518	C	5.68159687705250	11.44572439620291	20.50685882963016
C	4.72013993616808	16.30675138131631	18.91431261879602	H	-2.75863064106019	11.53159915040584	20.21704166609719
H	3.14152067585391	10.21031301184333	19.24198321152164	H	-1.17160358765414	18.74857799700989	20.15967388372404
H	-5.18078807093397	14.01863661346642	18.90363442333584	H	4.34220224219632	13.81907894304456	20.34767252496869
H	2.34715280835374	19.35080979177778	18.85327154499897	H	-10.16567861593217	11.64289021134467	21.06019560370385
H	-1.76178877434588	9.54812955676186	19.13535798288583	H	1.75015499684456	25.41423869197342	21.32000790863572
H	-3.23070223274568	18.55612598282676	19.05576658209924	H	8.29605990347542	7.61679056285236	21.28412939597599
H	5.36019936720292	15.43902997604782	19.05584876165703	C	-8.01307394292328	11.67584572769432	20.84447014357424
C	-8.45914347008895	9.71383715121434	18.97269101252821	C	1.09959828101162	23.38135647133503	20.95450507074566
C	-0.21818139776786	24.49856184156071	18.81707203829456	C	7.02612539423751	9.35368117747495	21.03982196606723
C	8.80113897229864	10.00775471647884	19.04519232478151	H	-0.52831774067088	12.47079714748134	20.50589821487853
C	-10.90338903164422	9.81858456672573	19.30765190412827	H	-1.45511660783378	16.34335948599508	20.44186624698676
C	0.62557360315115	26.76539786764802	19.36518701526651	H	2.40024695001169	15.26537904686551	20.56561200458933
C	10.11918689256604	7.94677172762464	19.41373138187890	C	-2.64254557006711	12.42026098462502	20.83511093006650
C	-7.16630015364744	10.11345386055619	19.22146515508575	C	-0.47309041966200	18.21009108030407	20.79725278075847
C	-0.32690642195593	23.14457577583320	19.03161198106051	C	3.49421014824958	13.46401609886897	20.93041687507321
C	7.74876055761616	10.85346980873567	19.30301862756041	H	-7.90253856110965	12.44794721927752	21.60638275535872
C	1.51258606410062	8.92644752090093	19.74723876389462	H	1.63107207444443	23.00268153963243	21.82808250813952
C	-5.47904629780902	16.05414619418221	19.44911232450729	H	6.36712357478422	9.04459372256994	21.85179993193867
C	4.28662365215974	18.63536119064086	19.36990267656152	C	-1.37376367822243	12.94734835669955	20.99454573004128
C	0.11856453855048	8.74525492295264	19.72207641847744	C	-0.64065902303167	16.84694453732130	20.95557487642338
C	-4.92019107409896	17.34184151051850	19.50216640749432	C	2.39379046898962	14.29241839839808	21.05133716033014
C	5.13879066995797	17.51951232258609	19.43191274295607	C	-5.12200114667798	12.54982841633242	21.23282095317381

C	0.79262199264874	20.32549978158836	21.25382014059410	C	1.43249957589427	18.15654121140190	22.28281087055222
C	4.64269040663901	11.27531591081631	21.37198522108540	C	2.40579895818779	11.80011515444128	22.30278412358766
C	-3.76966737615891	13.02574093351550	21.43598633127187	C	-2.27606303331295	14.70072313216339	22.40755074613786
C	0.57814453219671	18.90270339530727	21.43916558028794	C	1.27403080108965	16.78457082425653	22.44356406887454
C	3.52669662622270	12.18686355961768	21.53449660340593	C	1.29638533815922	12.62635310040722	22.43130497108374
H	-5.88077521001145	13.08328268818389	21.81483035137779	H	-4.39151879335651	14.64150439233375	22.74636911183762
H	1.51480135759805	20.78300890039601	21.93856347640780	H	2.23498587745649	18.66636603436072	22.82169142484408
H	4.60104494476865	10.37110781563768	21.98861866352745	H	2.40723775790607	10.83158399122279	22.80762939289938
C	-1.17117076076213	14.11198116022847	21.76298310679429	H	-2.13189467442593	15.58213846747716	23.03293102676570
C	0.24548097276790	16.10514974825536	21.76235871204555	H	1.94957751020050	16.23185124115466	23.09777021578996
C	1.26501091225523	13.87879646963454	21.78817564679382	H	0.44582115299005	12.30114342905395	23.03111930718762
N	0.11321615374050	14.69775017897027	21.84964972504762				
C	-3.54710673425245	14.16616509107539	22.24209212761582				

(h) 3Me-HBC⊂TPACage^{6+}

H	-4.30818722778833	14.04863583464649	11.84074883266587	N	-9.90827327511365	10.27984098048528	13.12697758430565
H	1.42582725671774	18.83806183493702	12.19687975149194	N	0.15472853458908	25.52766214749239	13.28345909442685
H	2.65187110720196	11.25502257394973	12.60683022171777	N	9.14692619205804	9.20029857123097	13.36710304807874
H	-8.48600751992602	12.92576936022101	11.64478129602251	H	-11.99092667880949	10.38820129735836	12.99767142104053
H	1.93577996149467	23.17777027525750	11.68915438695035	H	1.01571718605400	27.41421251030287	13.02488094258008
H	6.08217388677601	8.67906262648141	12.11710109792435	H	10.40756604141092	7.57543113091436	12.98894549717656
H	-2.21074320180942	15.30170647729214	12.00204021663192	C	-4.97140665067713	11.76387358651758	13.28113331827392
H	1.57718789309122	16.39731837296306	12.32441227596189	C	-0.54109651193229	20.42518182348162	13.35585940192658
H	0.48371081568759	12.36710139689777	12.53766433037576	C	4.97669754382398	12.23272113957226	13.74662553395298
C	-8.54519123287390	11.99324013128127	12.20680495180382	C	-3.73333645480882	12.52240881898054	13.28867160666364
C	1.14434564037289	23.59787649296685	12.31171539189991	C	-0.41527831255616	18.98035200986722	13.35333805357227
C	6.88505715643798	9.20128171367377	12.63867627885003	C	3.65428171039791	12.82972752410076	13.72966481994482
H	-10.70333175166435	11.86466229457680	12.03252905476071	C	-1.32802987368248	14.00092530552027	13.49840557762747
H	1.84023997394707	25.63869854461078	12.06493053933344	C	-0.20972757350950	16.16298287019561	13.53559226097011
H	8.32617815209062	7.61713469981229	12.29059988413985	C	1.11457382672919	14.10087550083270	13.68390975303663
C	-3.53436869582885	13.69640289998429	12.52558973894164	N	-0.13333626011394	14.75259061253418	13.60527510673019
C	0.65887722002699	18.28973488905416	12.74671949798615	H	-4.95438616433148	10.84212005536852	13.86937324106855
C	2.54323608524901	12.22109067520444	13.10218166495367	H	-1.40340979862424	20.80367972797405	13.91119949720315
C	-2.35049269641924	14.41167555401929	12.61550011019424	H	5.74247316927406	12.82609934173133	14.25172374977219
C	0.75336000515791	16.90859028346452	12.82256465333493	C	-7.54837262258340	10.19118402195926	13.45776770707124
C	1.30784860838364	12.84563877357302	13.06545741119904	C	-0.79873145486605	23.38897725129796	13.72344458729060
C	-9.78318034121651	11.41962138040064	12.41040444252420	C	7.75968548758475	11.06744572941123	13.88434700465285
C	1.10732799675605	24.96376077157163	12.50643097838720	C	-8.80471454239602	9.66025411701921	13.62390428871656
C	8.12264225567678	8.59688648820443	12.72168099960612	C	-0.79485419601132	24.75387262266300	13.87408478022599
H	-6.16591117856510	13.06006204107114	12.09516619324940	C	8.97165276882689	10.42560435548381	13.93036029219289
H	1.17243775432068	20.97350321675210	12.21655845314458	C	-11.24949296942160	9.77386834749618	13.52128274937695
H	4.57638860801462	10.44017263895213	12.67770330287110	C	0.21155410502987	26.97303166575940	13.62539544703456
C	-7.38138250066729	11.41843795968916	12.76651630887725	C	10.42071091952794	8.47701990446169	13.61236065364990
C	0.19542038560191	22.75442118405007	12.93362170644682	C	-2.68451578586352	12.10122508306929	14.13141550939827
C	6.65210243568060	10.46062322599527	13.23924864146448	C	-1.38615150655420	18.21761905615453	14.03533248067236
C	-6.12602850539513	12.12864625609850	12.66460837046408	C	3.44582848010698	14.08010065681757	14.35046304227891
C	0.30914134510248	21.32066241157846	12.78931454434816	H	-11.34516867728432	8.73710414183260	13.17471753721316
C	5.32925636687113	11.04091887275211	13.19373893377575	H	-0.73979325582347	27.43417238332596	13.33037437110302

H	11.24772599599796	9.11904690556258	13.28323488503190	C	-7.34719957159836	12.18618131868908	16.20922308790445
H	-8.97843221994320	8.74403800035826	14.18754980479157	C	1.85225814094668	21.89723807676474	15.88014870780515
H	-1.52521313863265	25.27549202428848	14.49333626719869	C	5.65897230362228	9.16272469387449	16.37882277367672
H	9.83621673349153	10.84874284211338	14.44116621381811	H	-8.30658987026686	11.72537838161840	15.94019261238298
C	-1.51208859752274	12.83391386152282	14.25743652026019	H	1.98137294265896	22.92340789009856	15.51316130712577
C	-1.28008286201764	16.83911046542335	14.14737885750489	H	6.49266488034413	8.54054051087907	16.02972803315146
C	2.20191228050478	14.69773484513058	14.34984565270035	C	-4.27775471379471	10.25827449886731	16.72362889050422
H	-6.70940951086953	9.67107605820527	13.91592624158825	C	-1.38437308954994	20.39133961959394	16.69908633853159
H	-1.54886272175357	22.81523835153041	14.26368896563876	C	5.90549097229487	12.77432416199386	16.94278070244561
H	7.66789061795603	12.02295299998346	14.39410867068356	C	-5.13577035510119	11.39780544891958	16.64833618619446
H	-2.81121014493588	11.19869109003469	14.72737725631565	C	0.02812799809581	20.51972688925291	16.54056459482077
H	-2.22448933237151	18.72797389503101	14.50885728969001	C	5.33704744570830	11.46411725684050	16.91655305066388
H	4.28075383108104	14.56891759330072	14.85288659823381	C	-11.21267964016723	11.21631423248781	17.03409106360305
H	-0.74039272682822	12.50883450692880	14.95399980468180	C	1.78371183186913	26.50904530601860	17.04891794386697
H	-2.02657153421555	16.28168525110560	14.71229174744072	C	9.49362388785348	7.22393286346127	17.07776476887572
H	2.07185211714354	15.64996767509980	14.86184643135376	H	-5.25577424757547	13.51900852241555	16.78716823985075
C	-11.37194633724721	9.86931866317863	15.02321295973364	H	1.90117967125608	19.50909204694064	16.63427727797993
C	0.45578565377458	27.10860143936039	15.10941072857501	H	3.55636035849809	10.31821492829412	17.16656209421454
C	10.51862907481065	8.15981836697126	15.08689823336726	H	-7.44494062153014	12.65690035982964	17.20152312890509
H	-11.28851611949511	12.03868038572526	15.04719715138202	H	2.41124122646074	21.78179878637996	16.82407194336513
H	2.43878822246267	26.23371838333508	15.01693900901667	H	5.29465596230465	8.76136074189595	17.34014528704554
H	8.78723769627759	6.84933741169916	15.08036624316656	C	-4.63311541719948	12.64610206260944	16.95928521579645
H	-7.09877818547198	12.96433074717197	15.46955465550095	C	0.83639629239606	19.44017382810329	16.83442356475435
H	2.25490327063256	21.19555828860772	15.13137529051527	C	4.00551220373383	11.30359806225409	17.25200258098546
H	4.84049328002321	9.11823674077484	15.64083083331282	H	-2.29790597970760	9.56504603374270	17.09654930906203
H	-11.47198653887840	7.73316723409126	15.32884836450905	H	-3.00022930306178	19.06911615902293	17.11207898254753
H	-1.46689495922767	28.00326119836412	15.52587737949519	H	5.52991684936430	14.85133456964084	17.17566423030521
H	12.25867161656924	9.39756359731184	15.41914970483327	C	-2.96563585628347	10.42019163867398	17.13088744094084
H	-4.66044480078480	7.12865326625001	15.87131367158261	C	-1.92155981050756	19.18559104899105	17.10958486014214
H	-3.89510908137338	22.41914335517986	16.16617862036078	C	5.11178417893749	13.85528289811132	17.27074535951868
H	8.85701565819831	13.97191195458950	16.19416988145825	H	-3.63506215176299	7.65410740780614	17.24725168554685
C	-11.30258676679507	11.12582112523679	15.64858430647106	H	-3.82753641512824	21.24291475631502	17.51947700942007
C	1.64085505487249	26.59987630041315	15.66786542071684	H	7.85222893805604	14.59844244467513	17.54187343935739
C	9.54080641899340	7.35123561649896	15.69279917221651	C	-11.32889844752215	8.80453122641276	17.20097962312492
C	-11.41068310983888	8.71324942415539	15.80792356514046	C	-0.40197946544301	27.50859604187880	17.34204155161965
C	-0.54720817832959	27.59573502955715	15.95338530156897	C	11.44696482122738	8.64049608884027	17.27526685367897
C	11.48945520594457	8.77487061642227	15.88301268185596	H	-11.12520951141139	12.19678951701674	17.51000951612689
H	-3.16651092322126	8.07156761743022	15.56302881183593	H	2.68778890396955	26.06659159125131	17.47626268381070
H	-3.95604579344082	20.66496129097096	15.82017264750499	H	8.70245825980387	6.63019353909719	17.54370018123937
H	7.31799269117233	14.81733942714390	15.83832970649747	C	-3.29742555060880	12.83073899764809	17.41703472033315
O	-4.83010020991716	9.09678614501645	16.31069303422310	C	0.30914348841364	18.22011754407874	17.33467279554993
O	-2.10724882010314	21.48627895452530	16.35767155034701	C	3.18339347450049	12.40034608970357	17.63394518695287
O	7.20712399234383	12.86001956797552	16.57270497316429	C	-2.44145193327792	11.69230038899486	17.49742973309293
C	-4.01743429734914	7.93354290414714	16.25080912384229	C	-1.10352062347065	18.06872991540357	17.43762167440666
C	-3.52134171693472	21.43530219157051	16.47741773153516	C	3.74265514520422	13.71095668219777	17.62584270261377
C	7.82826734241513	14.13717097315940	16.53982285175631	C	-11.19897995315471	10.05201284253205	17.81967043167006
O	-6.39271581078052	11.14162146213833	16.21942340581052	C	0.74451521555553	26.92880655054319	17.89584188999563
O	0.46309969234310	21.71102116490648	16.07756361632998	C	10.42746459746881	7.89821402120475	17.88187538960019
O	6.16818599359027	10.47692199323905	16.51805016504703	H	-11.32813238075538	7.89431290806613	17.80582102242816

H	-1.21106180941704	27.84479551598705	17.99492263288745	C	4.24641588431163	18.71279477681417	19.23354710090127
H	12.18263219937854	9.16153638655655	17.89300300324690	C	0.05437648354172	8.76527999983877	19.72134952082583
C	-0.50012798398703	13.16751271168949	17.77849154188912	C	-5.03305105494647	17.36562762205431	19.37371528451260
C	-1.36825005291832	14.31552273053409	17.72314624373235	C	5.07568075988934	17.57451360721491	19.35209762125394
C	-0.80644887378955	15.64411902982705	17.71547972623607	H	-11.10491169162373	9.18420954531638	19.79708031225375
C	0.62318414801561	15.82150798196211	17.71849234360336	H	-0.01448621802286	27.08217500787092	19.91465258629957
C	1.49417768501377	14.67536025492260	17.78986905684529	H	11.08607766970060	8.39036011775715	19.88623885990739
C	0.93161772013730	13.35092622786164	17.82403907507056	H	2.95178034062006	7.37405649072079	19.86518547206239
C	-1.05281289935808	11.87224861927277	17.86180573153180	H	-7.74661366475641	16.51385435332274	19.33763730821659
C	-1.65102969103573	16.77327760750886	17.78051104823817	H	5.59657809864134	20.40005740447450	19.22746308125121
C	2.89123573698879	14.85085031614376	17.87974582871005	H	-11.58193406897446	10.89982058164995	19.77767063301260
C	1.78114084538189	12.23402397566893	17.94325207151259	H	1.75701445032862	26.91227749875597	19.81607210800681
C	-2.76749373222894	14.13803482845820	17.74151654833678	H	10.00677574951549	6.97150095953637	19.80183471628798
C	1.16760707758465	17.11957333736231	17.70469747420691	N	-9.53335159128439	10.56215997014800	19.56550929801527
C	1.20534566483691	10.99811843170977	18.44257523705891	N	0.69606510985693	25.12896929366230	19.57349954820643
C	-3.58420927421408	15.25617645999370	18.17893966603037	N	9.03508216391066	8.81598114676488	19.67991915459362
C	2.53316774071447	17.29811091073279	18.16033234854366	H	-0.40793603684577	7.92690116530237	20.24926838195761
C	-0.21376214150814	10.81401753426746	18.39859087203405	H	-5.56546665006447	18.17825051287750	19.87524342766046
C	-3.01304108409065	16.56623732402238	18.23532169340534	H	6.04529243734891	17.65980706355495	19.84966292147393
C	3.39067097398162	16.15952386462910	18.26351364821481	H	-4.76960288032048	11.08262108215877	19.76042837881083
H	-8.78982021308605	9.13951176520851	18.23355468774355	H	-0.48676154988567	20.49224778491563	19.55433969856805
H	-0.71422194174984	24.93304157407945	18.05000758404466	H	5.71633827001797	12.23307418949224	19.85395263122611
H	9.68051579663475	10.28938997851724	18.35122186662210	C	2.33595805583637	7.99025288308174	20.54311524126866
C	1.99382742173499	10.05057788832376	19.13948087964082	C	-7.01396543432634	15.84432212596362	19.81706098930223
C	-4.88518158633575	15.06537561683753	18.71150042404511	C	4.69141158137260	20.05361034557117	19.75405157304906
C	2.99318150289816	18.54250800260803	18.66627077269305	H	3.03217479424117	8.53716350618824	21.20001415882798
H	-6.41924404374547	9.73111764879118	18.64695405486536	H	-7.34247562320274	14.81057003951877	19.63439217477107
H	-0.97936518777343	22.49719797075797	18.35310600074699	H	3.91129400023537	20.81743681235334	19.61511967502123
H	7.74645694306053	11.76687141633860	18.76441635366089	C	-6.88484351262244	11.29832478513947	20.09839709195568
C	-0.74996991516785	9.67782572034860	19.05965236929395	C	0.45317201568543	22.37206120831821	20.00208543537238
C	-3.76144853605193	17.58746257989193	18.87996113170441	C	6.81484592358346	10.43129499058924	20.22279419764209
C	4.65040797803639	16.34100537010288	18.89675777729669	C	-9.27084566494028	11.55263175401476	20.45307105517848
H	3.05908614791182	10.24111834566428	19.25342646112625	C	1.43271487207589	24.52524019347038	20.53837448269254
H	-5.29516713235220	14.06068591616431	18.74487659929618	C	8.09545739055153	8.40124724647737	20.56547800346733
H	2.31464697961207	19.39137432167495	18.67526418792635	C	-5.50306761210820	11.65810915811967	20.32917057062625
H	-1.82871640304526	9.54163667566003	19.11747788763053	C	0.26235663995823	20.95310600990388	20.20114433817777
H	-3.30851894047282	18.56318214255612	19.04488726053141	C	5.69764028123361	11.31900565260008	20.44967342279034
H	5.28440300472370	15.47824673128534	19.08839339909713	H	-2.76274323207709	11.55713683350807	20.16569262197188
C	-8.51406437131901	9.92302407748772	18.93852885078489	H	-1.08158764054404	18.61059885564496	20.10281414292180
C	-0.15327605373653	24.39559854045416	18.81230464097892	H	4.41017156488317	13.68315207057079	20.36240835559236
C	8.89984866331683	10.01329002363345	19.05809879122346	H	-10.13263233205252	12.02606106264903	20.92209034618495
C	-10.94123666081621	10.14987897759470	19.29956522163430	H	2.09665906812588	25.16849259970426	21.11530426938881
C	0.80702024203851	26.60427273507187	19.36326581721876	H	8.25824647272461	7.42744071697788	21.02671646068041
C	10.21699369560929	7.95758263572325	19.37175099348685	C	-7.97818003381820	11.93315206582663	20.73677543565649
C	-7.20308482945087	10.26615568831247	19.18157937697517	C	1.33201558436065	23.17142278079023	20.77278096690666
C	-0.29041752344177	23.03949806681230	18.99965299344513	C	6.99362737433610	9.17792132923931	20.85585582721518
C	7.81762616822235	10.82376406997678	19.30398756926856	H	-0.49729302965386	12.40747138125283	20.49934872309253
C	1.45356464138227	8.93910464284812	19.77467051988766	H	-1.37595732598070	16.21297266078047	20.44556071583963
C	-5.63060087466060	16.08911739239848	19.27685135760846	H	2.49527977566881	15.15178139148753	20.60824985918655

H	1.74493092000920	7.30632727304129	21.17110115103771
H	-7.06871460260889	16.03971063755784	20.90198309274243
H	4.94714226158701	20.01061128131956	20.82646929323582
C	-2.61575830784882	12.42014971949804	20.81248950538512
C	-0.38018274605831	18.08093449172021	20.74440359338651
C	3.55598509049874	13.32903312067742	20.93733265399280
H	-7.82982970900157	12.73109516187709	21.46368580561213
H	1.94083035944459	22.74578008546926	21.57023219554945
H	6.27139337920667	8.79109597983743	21.57483964622643
C	-1.32970923111973	12.89549632985849	20.99894299417775
C	-0.55415984540302	16.72373026615051	20.94045456265304
C	2.46984071069213	14.17182953110965	21.07689321504317
C	-5.09261315037579	12.64656158329988	21.16712896902175
C	0.92441222343697	20.18781381671523	21.10810855111967
C	4.66506545806165	11.11324225708943	21.30783131125279
C	-3.72432987895593	13.05866570167206	21.40910457496716
C	0.69299665286358	18.77544857788623	21.34262841445375
C	3.56417760960051	12.03751925273571	21.50806634328960
H	-5.84451178780603	13.22468739768579	21.71268182877593
H	1.70757803940727	20.64114207615667	21.72309919150401
H	4.62004659103143	10.18775794764590	21.89046032145475
C	-1.09757241946237	14.03267879610556	21.79540030561722
C	0.33896382122296	15.99804903936860	21.75348512296568
C	1.33380627277347	13.76225033089923	21.80397503730856
N	0.19718530435361	14.59576960544219	21.88486745905101
C	-3.47418238742559	14.16769745425637	22.24602005184088
C	1.56172276319616	18.04724975991221	22.18322849850873
C	2.43551300810411	11.64812081410541	22.26024410512874
C	-2.18461865479341	14.64391853563997	22.44579851405617
C	1.38753527283615	16.68421262027407	22.39395997447302
C	1.34229131735437	12.49277097757233	22.41313496466594
H	-4.31133667591216	14.66825925092246	22.73771078859572
H	2.38801316542289	18.56474418517247	22.67682119512722
H	2.41451817196014	10.66169840192530	22.72894013565358
H	-2.01531812224936	15.51179929377520	23.08399410678411
H	2.07518616872229	16.14208123132033	23.04427297330989
H	0.48154522832719	12.16050398025948	22.99426466146747

(i) 3F-HBC⊂TPACage^{6+}

H	-8.48658322853753	12.93294062605949	11.72796355314100
H	2.28182451216735	23.03307086560626	11.85203462329438
H	-4.32075656437754	13.99657159529389	11.89019820026645
H	-2.19871563889961	15.21075427068708	11.97417429125439
H	6.18906853915480	8.58572449221785	12.10508332765598
H	-10.71914212041133	11.90039721793191	12.10443489144116
H	1.53535321135462	18.70521728489007	12.11098936803089
H	2.19920757994928	25.49605241848205	12.21238776957951
H	-6.17306697944715	13.04302716635727	12.18692523518667
H	1.62783804894487	16.26102616401100	12.22152565055718
H	1.40850370642277	20.84813882260474	12.26701033988464
H	8.44115154430101	7.54945853915359	12.32878336061703
C	-8.56248911669987	12.01143384119544	12.30597000738591
C	1.44166674025870	23.47042646815688	12.39345627454336
H	0.52405831937123	12.25169780669401	12.47440747340029
C	-9.80865349223474	11.45369759144584	12.50304659786203
H	2.70266848046580	11.15191548530778	12.53058480732265
C	1.41344561623398	24.83746958969645	12.58219227862764
C	6.98085094116988	9.12709192725182	12.62411995867767
H	4.65719110046348	10.34246929795255	12.61127730394528
C	-3.53355524574961	13.63594928969177	12.55483047579519
C	-2.33478611789197	14.32942332695546	12.60085052468109
C	8.22275478602939	8.53608383683382	12.73624582545856
C	0.76215872325706	18.17214224744125	12.66712022504183
C	0.82232415675451	16.78884399688006	12.73186354417389
C	0.51876401478855	21.21163735290163	12.78705178419482
C	-6.14380946871912	12.11946403592444	12.76955464445314
C	-7.41067277800546	11.43183806374507	12.88555930769323
C	0.41917014632391	22.64814796605715	12.91954246805541
C	1.35042190939012	12.73675175275391	12.99325291439621
H	10.50327196852691	7.53436142609043	13.06199982443497
C	2.59101162641263	12.11956638324973	13.02247804522750
H	-12.03233164461244	10.44250466243329	13.05651829665952
H	1.31019055131340	27.29476803392664	13.08173508260801
C	5.39922796074795	10.96022846251314	13.12306466237114
C	6.72907802803306	10.39702749174712	13.19299461375766
H	-0.46387615407748	27.34075999078688	13.24198410066372
N	-9.95453681787702	10.32724311720709	13.23675893428846
H	11.33356139987582	9.09622997170202	13.27818180831033
N	0.40104000676678	25.42159736214157	13.26248367555197
H	-11.39991691773459	8.79303379208002	13.28138675273825
C	-0.28861028267958	18.88431345406388	13.28934020434195
C	-0.37918679319272	20.33192148153963	13.30249931023450
N	9.23211613116888	9.16266401357810	13.38235434906801
C	-3.73298443247459	12.47507703138799	13.33800624928864
C	-4.98593311010171	11.74079145785946	13.37159912157054
C	-0.14875263342849	16.06145905363577	13.45341945356781
C	-1.29300040741476	13.90983115332089	13.45699339992047
N	-0.08966009679378	14.65023230192394	13.52934767754688
C	0.45284613204746	26.86465165905524	13.61272997808945
C	10.51634233503782	8.46208317132244	13.64512506859821
C	-11.30686500495284	9.83576032761319	13.61054530319088
C	-7.59986956883232	10.22125192362638	13.60003506987367
C	1.15645722605275	13.99381129912122	13.60662153064794
C	-0.63696058534923	23.30487244503153	13.60329925469690
C	3.70262486490952	12.73834938122397	13.63881260971734

C	5.03064184144981	12.15411025975845	13.65912648488792	C	1.85266231609122	21.98382956704908	15.99209781037843
C	-8.86426898670118	9.70688800438345	13.76121146961487	H	-4.64792062109155	7.13846925180800	15.96100483291799
C	-0.61980762566687	24.66958841426275	13.75372808185401	H	6.70671455764363	8.68112256286001	16.09277561024668
C	7.82172383442181	11.02843681333920	13.84058342602359	C	-3.52755262613285	21.38023385733715	16.19267676514563
H	-1.25528792336116	20.72638381663833	13.82354531441895	O	0.45897678757800	21.75624605789217	16.09296408992257
C	9.03841250285990	10.39789958627587	13.91734388605065	H	8.90177377566862	14.18191023792413	16.13204101392456
C	-1.27503018517095	18.13980924555529	13.96892080208686	H	-8.30553172651397	11.72784956022296	16.11253962658403
H	-4.97476208690577	10.82636851375398	13.97180823017105	O	-2.10981940169554	21.46789850745355	16.18975562119415
C	-1.19999416846604	16.75835808125581	14.07441476051565	C	5.83722837970392	9.29343494503904	16.36263641341245
H	-6.77144464100936	9.70029324117853	14.07636230054736	C	-3.99834859361663	7.94390069821643	16.32779372605199
H	-1.45429176809831	22.74959555928466	14.05840180760704	C	-7.34206313117036	12.18851309576275	16.36693412367876
H	5.78913090269922	12.76081884782349	14.15940070606077	O	-6.38872323162499	11.14119877396341	16.37149241975104
C	-2.66486110975446	12.04099064966241	14.14939124004445	O	6.29291505355913	10.63297106124403	16.43254813925188
C	-1.47368866879110	12.75062593777070	14.22943447105787	O	-4.81304669070509	9.10482775138161	16.41011177608038
C	3.49074033169273	13.98763345720443	14.25940902112984	C	7.87225120196168	14.32315815908853	16.48584453495133
C	2.24358574259889	14.59634326987823	14.26714106100586	O	7.27609644190583	13.03378441303944	16.50703114033180
H	7.71659800709340	11.99404027149918	14.32975370192347	C	0.02716539559088	20.55800913735419	16.53500327845456
H	-9.05471084851985	8.80323408005246	14.33986376375825	C	-1.39071611592069	20.39822692525065	16.60374910662926
H	-1.39789657163724	25.20788348806898	14.29560102984149	C	-5.12302571584183	11.40192283544808	16.76610640239374
H	9.89096876258922	10.83905521804114	14.43304220494657	H	1.91507365458080	19.58919136336506	16.74021727823450
H	-2.09958419276985	18.66580554213290	14.44854947287374	C	5.43063993744664	11.60200892372083	16.80535262402570
H	-1.95519791819113	16.21628877090303	14.64284900121473	C	-4.25595170711342	10.26648405107353	16.81175269959011
H	-2.78987558020715	11.14703918021675	14.75854621024670	C	5.97563064852478	12.92155308745739	16.86689278009594
H	4.32570346914656	14.48274696601812	14.75521002456106	C	0.84161506679267	19.49810946147959	16.88018493798084
H	2.11210108391053	15.54930754900214	14.77675647724997	H	3.66345033177971	10.42724047124347	16.93118571011556
H	-0.68579625571395	12.41358867753618	14.90184772007744	H	-5.25078811859091	13.52339304046663	16.92087141559426
C	10.63323123230314	8.21106491761202	15.12985009965958	H	-3.00246836253535	19.05019788796441	16.96256541968260
C	0.57853817341433	26.99435433586598	15.11186501080627	H	2.35239875600884	21.87004304935430	16.96880001906289
H	8.95059311762642	6.84042318672289	15.18666074888823	C	-1.92651710059241	19.18973091050266	17.00921038820405
H	-11.34330456828180	12.12842053506010	15.09507223000502	C	4.09013782549588	11.41375480145309	17.08205556873028
C	-11.46978082250992	9.96093496628162	15.10650131567324	C	-4.61795462157508	12.65229685250946	17.06572695279693
H	2.55877495452838	26.10909044960857	15.17176205908695	H	-3.91435994558554	21.20694198823190	17.21080230689589
H	2.32406156153453	21.30471137286739	15.26301391006194	C	9.64785883973335	7.32877894221144	17.16376316233864
H	-1.36882104279023	27.89384371049644	15.37916695600295	C	-11.35153819351437	11.34189193051651	17.09755158459340
H	12.32803435051340	9.52452227751373	15.39902460874998	C	1.75465318554328	26.39837356788456	17.14790949076516
H	-3.88429602320571	20.57914101403776	15.52527856972416	H	-2.26350089079765	9.58168512749659	17.11741614704237
H	-11.62376639884835	7.83315683247938	15.44751559002030	H	5.55581837695554	14.98725037292643	17.15412335535092
H	5.05536951655120	9.16554952638494	15.59595568336522	C	-2.93228520823149	10.43437687510162	17.17745680097791
H	-7.10059006671186	12.96002685417422	15.61810560497018	C	5.15665122102218	13.97960595326526	17.21173766102847
H	1.97610235464959	23.01934179912929	15.65086365868010	C	-0.44432702296442	27.40477258937001	17.27199869970617
H	-3.16439205728058	8.08628525603261	15.62045086667825	C	11.54656859105474	8.82622331488708	17.29082608412478
H	-3.89931063542550	22.34414657089777	15.82279214953358	C	-11.52237775002660	8.93633183777881	17.30347612453015
C	9.68649187989999	7.39549755386604	15.77439455689034	H	5.44785553579173	8.94540022256382	17.33469252452261
C	-11.39670683477604	11.22714930870298	15.71153852385755	H	-3.59286627224948	7.66320050172869	17.31468794041588
C	1.71543470405215	26.48121362066685	15.75938722155513	C	0.30997931216196	18.26456160259334	17.34086845952424
H	7.34402562283314	14.99963740420102	15.79445878328243	H	-7.42501137765342	12.66595527300862	17.35733172866636
C	-0.48469194385890	27.48548617447283	15.87564898161753	C	-1.10306996292198	18.09339956509959	17.38841754775752
C	11.58243675422738	8.89683108689573	15.89374800562992	C	-3.26671132506400	12.84182658599726	17.47080464523988
C	-11.55896712497931	8.82077922002797	15.91025794913596	C	3.24068650055011	12.48185049562373	17.48282646722144

H	7.89176582181903	14.77332429962467	17.49297984467323	C	8.99908842339252	10.21720508966706	19.14036558848995
C	-2.40103919313297	11.70960276078737	17.52235341929465	H	-1.70540011068954	9.57695591090433	19.12019089683112
C	3.78410373303107	13.79839183497772	17.53893774006144	H	5.25473963444137	15.49168622771132	19.15321549542966
H	-11.25871312787828	12.32910907877676	17.55825400399834	C	-7.42381989315913	10.33144448302311	19.31009005362801
H	8.88079640111395	6.72653120438958	17.65852276686630	C	7.86053462744829	10.96527234018613	19.31665966216195
H	2.62401277369651	25.95772589065836	17.64360101978287	C	0.61672729082582	26.52529189196755	19.38645052106741
C	-0.78954546865550	15.67446440996780	17.68079311362118	C	10.35037599792977	8.19007936999205	19.42147319221167
C	0.63731823136754	15.86194742930037	17.68561568724740	C	-11.16274866203527	10.31107011115944	19.38803469824489
C	-1.33797146052273	14.34121501693721	17.70557653912110	C	-5.47179971457013	16.10239145887551	19.43957719908651
C	1.16655016678989	17.16472951263983	17.71056904201297	C	1.57845017304426	8.92782399996790	19.43892638390999
C	0.97337321746510	13.39334019951228	17.72803207699118	C	-4.94711144078963	17.39848651797348	19.47384359598817
C	-1.64210693556818	16.79712292984937	17.73745582183530	C	4.12019112209130	18.64431141923676	19.47513085961370
C	-0.45830585392799	13.19997799301570	17.72997559388390	C	0.19619819000315	8.73488288935776	19.53445007486751
C	1.52018077316996	14.72392701395781	17.72745202389284	N	0.51376915330496	25.05275848771030	19.60758147262145
C	-2.73348869866050	14.15190126985471	17.76731898126626	H	-0.65632475706045	20.41712572788593	19.59960741140813
C	1.84020230490210	12.28400904341463	17.78697112302424	C	4.98787301963005	17.55216705383472	19.58565521438941
H	-1.29807799939084	27.74835398267926	17.86135359028485	N	9.11349837833295	8.97932983121846	19.68265114171784
C	2.91399523759025	14.91290052943898	17.83047944233851	N	-9.74805653037756	10.68709046894503	19.67244567665528
C	-1.00035583881073	11.90128920473031	17.83101827089351	H	5.69851899703779	12.29263908417173	19.80372990505058
H	12.26485116960840	9.39928489656176	17.88255122809750	H	10.21330968798969	7.21562326459982	19.90350257142412
C	10.55688791836418	8.07457712245458	17.93296272902687	H	-11.79054474877817	11.08844193513107	19.83872182764780
C	0.65733249448075	26.82770629987537	17.91261162523922	H	-0.25061331924492	26.99686121784426	19.86858531207114
C	-11.38736341369428	10.19230937895299	17.90395108910377	H	-4.98629917503383	11.11566521172275	19.84847162787129
H	-11.56041024508122	8.03742168429322	17.92376257521411	H	11.18735153133453	8.70926692495593	19.90692616993449
H	-0.88419935202947	24.84912029895793	18.07418432524506	H	1.52606186380866	26.85481952166142	19.90287729388601
C	-2.99328702372720	16.58817765535682	18.22498332499151	H	-11.36398646026283	9.35989195278925	19.89920324783003
C	-3.54389678420609	15.26590889739287	18.22629868261621	F	2.41161810038759	8.03207056578889	20.00481381801685
C	2.51448713178922	17.33840243184851	18.21369697286281	H	-5.48036756857901	18.19241540774303	19.99885685932218
C	1.29123001398141	11.02666262876916	18.25783376252573	F	-6.64327122844187	15.85813286236581	20.06162309746869
C	3.38666829836549	16.20692724260551	18.29438314818169	C	0.28775389733847	22.29683706455307	20.03621958362922
C	-0.12829673153077	10.84085460967777	18.30607294039122	C	6.78034014826321	10.46857668073262	20.08599743632451
H	-1.13847755203155	22.40986168342445	18.38216326149373	H	-0.20652640080413	7.87353285548502	20.07052332801201
H	-9.02859862761865	9.21692544294494	18.37960899042340	F	4.43921153625497	19.80002180890370	20.09088065991968
H	9.84533553730297	10.57635664696772	18.55647176212734	H	-1.23075041543869	18.53304528487586	20.13884168812393
H	-6.64689372297570	9.77151399199299	18.79077061643117	H	-2.98398728236333	11.56165081836349	20.16283884576499
C	-4.80266070687555	15.05745283912861	18.84398436045202	H	5.91217010633852	17.64247085974658	20.15867559813693
C	2.12184810763814	10.03977827905406	18.84041014668134	C	-7.08984550844433	11.37663720918639	20.20540723702101
C	-0.32598323706307	24.31453551819196	18.84082298383101	C	0.10359679364677	20.87632617018490	20.23483150718853
C	-3.72046641381532	17.62125187770765	18.87401840037274	H	4.28725935991536	13.65486959412324	20.24339464289709
C	2.91865040730283	18.55814466233964	18.81150034277178	C	5.63343161140970	11.32130805852101	20.29544494152478
H	3.19640253723275	10.17720297113484	18.90475024169685	C	8.09270584599434	8.45519303398079	20.40377181977358
H	2.25436924993332	19.41714269305141	18.84551829143190	H	-0.69335962209435	12.36871769042224	20.42786023333273
H	-5.22705384353787	14.06297205159198	18.93831580522824	C	-5.69905491522831	11.70231478916278	20.43254844634778
H	7.80686075151991	11.94785713571943	18.85046912055815	H	-1.50826185996457	16.13056649674262	20.46015452108007
H	-3.28348011647813	18.61352455054834	18.96407219105400	C	6.93073223165843	9.16670777952290	20.62038859332698
C	-0.63277404996496	9.69361532641894	18.97705388592329	C	-9.46988180800743	11.69309292010952	20.53703281014890
C	-0.45532024887124	22.95813400295923	19.02940092808128	C	1.24785448150451	24.45609570845779	20.57828735551260
C	4.61071081912070	16.35522281383449	19.00042255645396	H	2.38492840507422	15.11461996741017	20.58198887929792
C	-8.74000644080762	10.01079829562588	19.06743194873688	H	8.24455447473560	7.45202670065324	20.80097864301036

C	-0.51566117670713	17.99993220885182	20.76219067579459	H	1.75998475474132	22.67917399045554	21.61575969467814
C	3.43612909474730	13.27249487582470	20.80391413159333	C	1.22066382426881	13.66913045387355	21.70885288357544
C	1.15616237617177	23.10109506684087	20.81251561134294	H	1.58592061983252	20.55887769377703	21.71961351577155
C	-8.17059300479191	12.05329076591712	20.81989819323197	C	0.22998920893355	15.90959116559147	21.73582112420065
C	-2.79515908213596	12.39776686566752	20.83472592915858	C	-1.21091407446361	13.95380181708634	21.80572034514987
C	-0.67921658439022	16.63954906726142	20.94506860448606	H	-5.97708016888493	13.24445172584152	21.86023030837107
C	-1.49518347077622	12.84876319710104	20.98124960105314	N	0.09283168784583	14.50368318316475	21.85093488283849
H	-10.32463505713554	12.19919085987403	20.98413727436598	C	2.31085466916575	11.52159354556174	22.02427008955551
C	2.35544952122804	14.11125564663144	20.99727539716442	C	1.45030824366347	17.95982152742634	22.16772869667470
C	4.54526985058370	11.03788601415426	21.05814168711337	C	1.22683900135831	12.36389088351341	22.24024717832215
H	6.14668900802099	8.69760748669242	21.21495986235180	C	-3.56363577981365	14.10795057423229	22.36263160317792
C	0.78624753972850	20.10884096500659	21.12373386569970	C	1.28543972182362	16.59317212863474	22.36660743247743
H	1.90120852426570	25.10585860803179	21.16005767197519	H	2.28710671157976	10.50846462675644	22.43182499917404
C	-5.25157975050259	12.65959946970836	21.28661443838827	C	-2.25857398624447	14.55737419713423	22.52389777470871
C	3.44129123074131	11.94907275266288	21.29568765509901	H	2.28401210381869	18.47577150476445	22.65025286968722
C	0.56374753680539	18.69291390006968	21.35050577698548	H	0.37211018417241	12.00372113032094	22.81317896913790
H	4.46061265377037	10.05808049135911	21.53773495985433	H	-4.37162369874171	14.60486884943475	22.90413434900003
C	-3.86676036893677	13.03310672518910	21.49949117929466	H	1.98640309651364	16.04705361944227	22.99902465087682
H	-8.00906875086226	12.87765560578753	21.51429534390640	H	-2.04828942576974	15.40132608566414	23.18201664056416
